# Detection of selection signatures in Piemontese and Marchigiana cattle, two breeds with similar production aptitudes but different selection histories

**DOI:** 10.1186/s12711-015-0128-2

**Published:** 2015-06-23

**Authors:** Silvia Sorbolini, Gabriele Marras, Giustino Gaspa, Corrado Dimauro, Massimo Cellesi, Alessio Valentini, Nicolò PP Macciotta

**Affiliations:** Dipartimento di Agraria, Sezione di Scienze Zootecniche Università degli Studi di Sassari, 07100 Sassari, Italy; Dipartimento per l’Innovazione dei Sistemi Biologici Agroalimentari e Forestali DIBAF, Università della Tuscia, Viterbo, Italy

## Abstract

**Background:**

Domestication and selection are processes that alter the pattern of within- and between-population genetic variability. They can be investigated at the genomic level by tracing the so-called selection signatures. Recently, sequence polymorphisms at the genome-wide level have been investigated in a wide range of animals. A common approach to detect selection signatures is to compare breeds that have been selected for different breeding goals (i.e. dairy and beef cattle). However, genetic variations in different breeds with similar production aptitudes and similar phenotypes can be related to differences in their selection history.

**Methods:**

In this study, we investigated selection signatures between two Italian beef cattle breeds, Piemontese and Marchigiana, using genotyping data that was obtained with the Illumina BovineSNP50 BeadChip. The comparison was based on the fixation index (F_st_), combined with a locally weighted scatterplot smoothing (LOWESS) regression and a control chart approach. In addition, analyses of F_st_ were carried out to confirm candidate genes. In particular, data were processed using the varLD method, which compares the regional variation of linkage disequilibrium between populations.

**Results:**

Genome scans confirmed the presence of selective sweeps in the genomic regions that harbour candidate genes that are known to affect productive traits in cattle such as *DGAT1, ABCG2, CAPN3, MSTN* and *FTO*. In addition, several new putative candidate genes (for example *ALAS1*, *ABCB8*, *ACADS* and *SOD1*) were detected.

**Conclusions:**

This study provided evidence on the different selection histories of two cattle breeds and the usefulness of genomic scans to detect selective sweeps even in cattle breeds that are bred for similar production aptitudes.

**Electronic supplementary material:**

The online version of this article (doi:10.1186/s12711-015-0128-2) contains supplementary material, which is available to authorized users.

## Background

After domestication, natural and artificial selection have led to different animal strains. During the industrial revolution (200 to 250 years ago), these animal strains were artificially clustered into breeds based on their phenotypic characteristics and the environmental conditions in which they are raised. Animals have been selected for traits that are important for various human communities [1, 2]. Domestication and subsequent natural and artificial selection have changed the frequency of mutations that affect phenotypic traits [3]. Understanding how and where selection has shaped the patterns of genetic variation remains one of the most challenging topics in genetics [3]. Thanks to their great phenotypic variability, domestic animals offer the opportunity to explore genotype-phenotype relationships and represent excellent models for studies on evolutionary biology [4–6]. According to the theory of genetic hitch-hiking [7], when the favourable allele of a gene spreads in a population, the sequences that are upstream and downstream of this gene also undergo an increase in frequency until fixation [8]. The integration of disciplines such as quantitative genetics and population genetics has made the study of species’ variability easier and more reliable [5]. For example, association and hitch-hiking mapping allow the identification of quantitative trait nucleotides (QTN) that are responsible for differences in economically important traits [9].

The recent advances in genetics and statistical methodologies contribute to the characterization of biological diversity, animal domestication, and breed development [4]. In particular, the availability of high-density single nucleotide polymorphism (SNP) panels provides an exciting opportunity to identify genomic regions under selection [10]. The abundance of SNPs throughout the genome makes these genetic makers particularly suitable for the detection of genomic regions where a reduction in heterozygosity (selective sweep) occurred [11]. In mammals, several methods based on the measurement of differences in allele frequency, and on linkage disequilibrium (LD) patterns and haplotype structures have been used to examine selective sweeps or patterns of diversity [12–16]. In population genetics, the most commonly used statistics to detect signatures of selection are the calculation of the fixation index (F_st_) [17], composite log likelihood (CLL) [18] and extended haplotype homozygosity (EHH) [19]. Recently, in humans, intra- and inter-population genetic diversity was investigated by comparing continuous stretches of diploid DNA sequences that are identical on each strand [20] and that are called runs of homozygosity (ROH) [21].These methods can help to identify genomic regions that have undergone selection (natural or artificial) and to detect associations between traits of economic interest and genes present in these regions. In cattle, most studies have compared breeds with different production aptitudes, for example dairy and beef breeds [18, 22, 23], in order to detect signatures of selective breeding [22, 24, 25]. As expected, these comparisons highlighted genes that have a huge effect on phenotypes (i.e. *DGAT1* or *ABCG2* for dairy and *MSTN* for beef cattle, respectively). However, there is a wide range of bovine breeds with different selection histories some of which have the same production aptitudes. For example, some dairy breeds have been selected mainly to improve milk yield, whereas other breeds have been privileged for milk composition or functional traits. Therefore, studies that compare breeds with similar production aptitudes [13, 26–29] can be considered highly informative to investigate their genetic variability for breeding purposes. Here, we studied the genetic differences between two Italian beef cattle breeds, Piemontese and Marchigiana. These two populations exhibit similar morphological and productive traits, but have different origins and selection histories. We analyzed the genetic variation between these two breeds that were genotyped with the Illumina BovineSNP50 BeadChip assay (http://www.illumina.com) by comparing the SNP allele frequencies. We used smoothed fixation indices (F_st_) [30] and their interpretation followed the approach of Pintus et al. [23]. In order to confirm the results obtained with the LOWESS/control chart procedure, we performed an analysis with the varLD software that measures the genetic variability between populations by comparing the differences in regional LD patterns [31].

## Methods

### Samples, genotyping and data editing

A total of 364 Piemontese and 410 Marchigiana bulls were sampled for this study. Animals were genotyped using the Illumina Infinium Bovine BeadChip that includes 54 001 SNPs (http://www.illumina.com). SNPs that were not located on the 29 autosomes of the *Bos taurus* UMD 3.1/bosTau6 build of the bovine genome assembly were excluded. Quality controls removed SNPs that were monomorphic in both breeds and that had more than 2.5% missing data or a minor allele frequency less than 1%. Missing data were replaced with the most frequent allele at that specific locus for each breed. After quality control, 43 009 SNPs were retained for the analysis.

### Detection of relevant signals using the LOWESS/control chart procedure

First, allele frequencies and observed and expected heterozygosities were calculated separately for each breed. Then, total allele frequencies at each locus, *f*_*p*_ and *f*_*q*_, were calculated by considering all animals as a single population as follows:

$$ {f}_p=\frac{\left[{f}_{Pm}\left(2{\mathrm{n}}_{Pm}\right)+{f}_{Ma}\left(2{\mathrm{n}}_{Ma}\right)\right]}{2\left({n}_{Pm}+{\mathrm{n}}_{Ma}\right)}, $$

where *f*_*Pm*_ and *f*_*Ma*_ and *n*_*Pm*_ and *n*_*Ma*_ are allele frequencies and number of individuals in the Piemontese and Marchigiana breeds, respectively.

$$ {f}_q=1-{f}_p. $$

Expected heterozygosity in the populations (*H*_*s*_) and overall heterozygosity (*H*_*t*_) were calculated. Finally, F_st_ was calculated according to Weir and Cockerham [30] as:

$$ {F}_{st}=\frac{\left({H}_t-{H}_s\right)}{H_t}. $$

F_st_ computation generates F_st_ data patterns along the chromosome that are usually highly variable and thus difficult to interpret.

In this study, in order to smooth F_st_ data patterns and to simplify graphical presentations, values were fitted with a locally weighted scatterplot smoothing (LOWESS) regression [32], separately for each autosome, using the PROC LOESS procedure in SAS 9.2 (SAS/STAT® Software version 9.2, SAS Institute, Inc., Cary, NC, USA) as suggested by Pintus et al. [23]. The number of local regressions varied between chromosomes due to differences in chromosome length. The data interval included in the analysis was defined by the smoothing parameter *S* used for the LOWESS regression [33]. We applied the same smoothing parameter as applied in [23] that corresponds to an interval of 20 SNPs for each separate regression. Additional file 1: Table S1 contains the different smoothing parameters that were used in the analysis for each chromosome.

Since F_st_ values that deviate from the average pattern can be considered as signatures of selection, LOWESS-smoothed data were analysed using a control chart approach. Control charts are graphically represented as a flow of data between two control limits. In this study, LOWESS-smoothed F_st_ values were plotted against their position along the chromosome and the limits of the control chart were set to three standard deviations from the mean F_st_ value. LOWESS-smoothed F_st_ values that exceeded these limits were flagged as outlier signals.

### Detection of relevant signals using the varLD software

In order to confirm the results obtained with the LOWESS/control chart procedure, the varLD method was applied to our dataset according to Teo et al. [34]. We chose this method because it is based on assumptions that differ from those of the LOWESS/control chart approach and it has already been used to detect selection signatures in cattle [35]. In particular, it is based on LD and compares patterns of LD across populations. Data editing parameters (minor allele frequency (MAF) and % of missing data) and haplotype length (number of SNPs) for the VarLD method were set equal to those of the LOWESS procedure.

### Annotation and functional analysis

Annotated genes in the genomic regions that corresponded to peaks that were above the upper limit of the control chart were identified from the UCSC Genome Browser Gateway (http://genome.ucsc.edu./) and National Centre for Biotechnology Information (NCBI) (www.ncbi.nlm.nih.gov) databases. Intervals of 0.25 Mb upstream and downstream of each significant SNP were considered. Gene-specific functional analyses were performed by GeneCards (www.genecards.org) and NCBI databases consultation. In addition, functional interactions between proteins that were encoded by some of the candidate genes were investigated using the database STRING 9.0 of functional protein association networks (http://string-db.org/) [36]. Finally, to investigate the biological function of each annotated gene (and related proteins) contained in the significant genomic regions, an accurate literature search was conducted. Gene names and symbols were derived from the HUGO Gene nomenclature database (www.genenames.org).

## Results

### Detection of selection signatures using control chart and varLD approaches

**Fig. 1:**
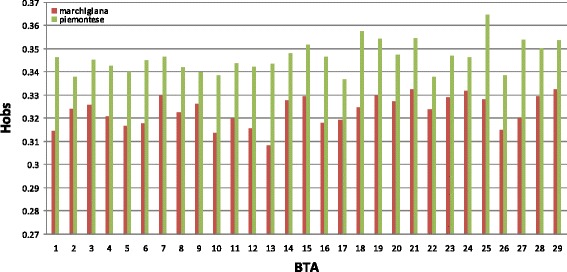
Comparison of average heterozygosity (H_obs_) per chromosome (BTA) between the two breeds. Green = Piemontese and red = Marchigiana

The average genome-wide observed heterozygosity was lower for the Marchigiana breed (0.327) than for the Piemontese breed (0.346). The largest and smallest differences between the two breeds were observed on BTA25 (0.033) (BTA for *Bos taurus* chromosome) and BTA2 (0.009), respectively (Fig. 1). The overall genetic differentiation between the two breeds was weak (mean F_st_ = 0.0285 ± 0.004 SD). This value suggests that 2.85% of the genetic variation observed in the sample is explained by population differences, whereas the remaining (97.15%) is due to individual differences within the population.

**Fig. 2:**
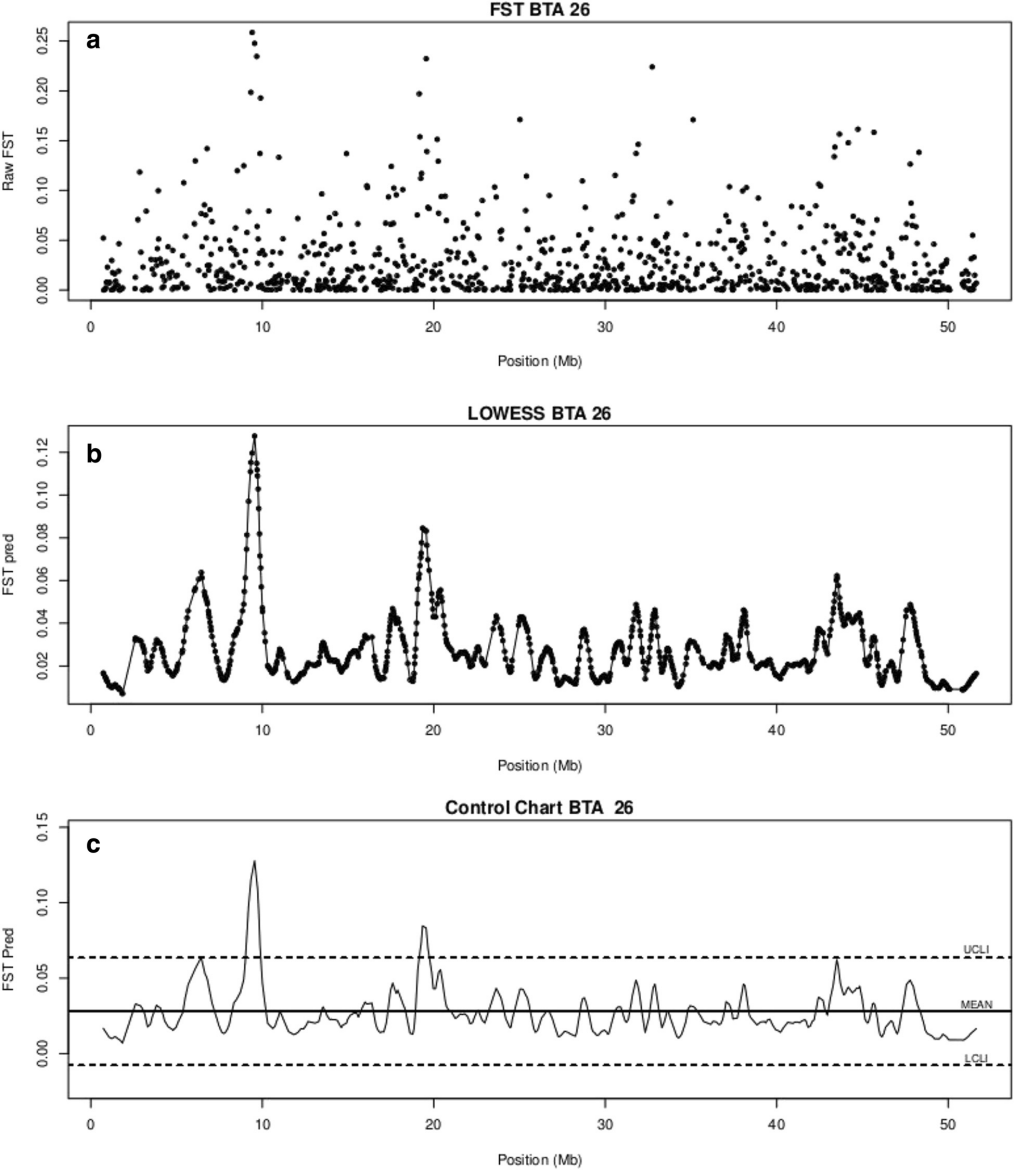
**a** Pattern of raw F_st_ data calculated for SNPs located on BTA 26. **b** Predicted F_st_ values for SNPs located on BTA 26 using the LOWESS regression with the smoothing parameter set at 0.022. **c** Control chart of predicted F_st_ values for BTA 26. Solid line = mean, dotted lines are upper (UCLI) and lower (LCLI) control limits. These control limits are three standard deviations apart from the mean value. The borderline peak is at about 7 Mb

**Fig. 3:**
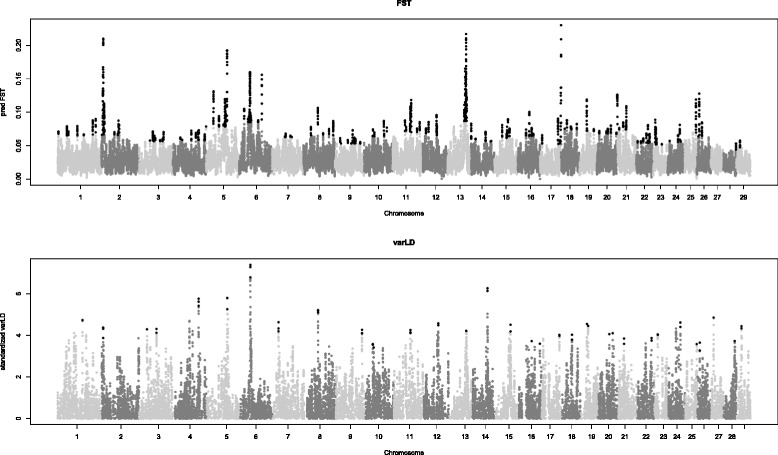
Comparison between genome-wide F_st_ and varLD analysis for the two breeds. Manhattan plots demonstrate the presence of significant signals in the same regions on several BTA chromosomes between genome-wide F_st_ and varLD analyses. Black dots represent significant signals with whole-genome significance thresholds set at three standard deviations apart from the mean value

The LOWESS/control chart analysis detected 138 outliers in the whole genome. Additional file 2: Figure S1 shows that the largest number of significant peaks (*n* = 10) was found on BTA6 whereas on BTA25, 28 and 29, only one peak was detected. No significant peak was observed on BTA27. Moreover, several intriguing peaks on BTA2, 5, 8, 9, 12, 14, 23, 26 and 29 were detected [See Additional file 2: Figure S1] but because they did not exceed the upper limit of the control chart they were considered as borderline. Figure 2 shows an example of a borderline peak on BTA26 at about 7 Mb. The varLD method (Fig. 3) detected 67 significant outlier SNPs at the genome-wide level whereas less than half of these were found by the LOWESS/control chart approach. The maximum number of significant SNPs observed with varLD was on BTA1 (*n* = 4) followed by BTA2 to 11 with three, BTA12 to 24 and BTA26 and 29 with two and BTA25, 27 and 28 with one significant SNP. A total of 933 and 189 annotated genes, derived from the bovine genome assembly (*Bos taurus* UMD 3.1/bosTau6) UCSC Genome Browser Gateway and NCBI databases, were detected in the genomic regions that surrounded peaks that exceeded the upper limit of the control chart [See Additional file 3: Table S2] and varLD method, respectively [See Additional file 4: Table S3].

### Identification of genes that are known to affect bovine production traits using control chart and varLD approaches

The reliability of the LOWESS/control chart analysis was confirmed by the detection of outlier signals that were located in the genomic regions that contain genes known to affect production traits in cattle. Among these genes, *MSTN* on BTA2, *ABCG2* on BTA6, *DGAT1* on BTA14 and *FTO* on BTA18 should be noted (Table 1). Analyses of the genome-wide Manhattan plots of F_st_ and LD showed that, overall, the results obtained with the varLD and the LOWESS/control chart approaches were comparable (Fig. 3). Overlapping outlier signals were detected on eight autosomes by both methods. For example, both procedures detected significant outlier signals on BTA2 and 6 [See Additional file 5: Figure S2] in regions that contain annotated genes known to affect bovine production traits (Table 1). Furthermore, significant signals that were detected by both methods were identified at the same positions on BTA4, 13, 17, 19, 25 and 26. Obviously, both methods identified the same annotated genes in these regions (Table 2). Moreover, eight chromosomes (BTA5, 9, 11, 12, 15, 18, 21 and 23) showed peaks at positions that did not correspond exactly in terms of base pairs, but were located within the same autosomal region as several annotated genes known to affect bovine production traits [See Additional file 5: Figure S2]. For 13 chromosomes, no common outlier signals were detected with both methods. Finally, regarding BTA27, no significant signal was detected using the smoothed F_st_, whereas one significant SNP at 8.4 Mb was observed with the regional LD variation method but there was no annotated gene in the 0.5 Mb region that surrounded that SNP (see Methods section). Moreover, BTA1, 8, 14 and 28 exhibited significant signals [See Additional file 5: Figure S2] in regions for which no annotated gene was found.

**Table 1:** Genes known to affect bovine traits identified by the control chart approach

BTA	Position (bp)	Gene symbol and name
2	6213566-6220196	*MSTN myostatin*
6	37959536-38030586	*ABCG2 ATP-binding cassette, subfamily G (white), member 2*
10	37829007-37885645	*CAPN3 calpain 3*
13	66863225-66872531	*GHRH growth hormone releasing hormone*
14	1795425-1804838	*DGAT1 diacylglycerol O-acyltransferase 1*
18	22118201-22541539	*FTO fat mass and obesity associated*

**Table 2:** List of common candidate genes that were detected by both the control chart and varLD methods

BTA	Position (Mb)	Gene symbol
2	5.6 to 5.8	*NABP1, INPP1*
4	92.2 to 92.3	*GRM8, ZNF800, MIR592*
6	37.9 to 38.1	*HERC6, PYURF, NAP1L5, HERC5, FAM13A, PPM1K, ABCG2, PKD2, IBPS, MEPE*
13	63.2 to 63.6	*SNTA1, E2F1, ZNF341, CHMP4B, PXMP4, NECAB3*
17	64.8 to 65.0	*SIRT4, RPLP0, RAB35, PLA2G1B, ALDH2, COX6A1, GATC, ACADS, POP5, RNF10, MAPKAPK5, TRIAP1, SRSF9,DYNLL1, MLEC,UNC119B, CABP1*
18	35.7 to 35.8	*CDH3*
19	28.4	*PER1, HSE, ALOX15B, GUCY2D, PFAS, RANGRF, RPL26, KCNAB3, TMEM, CYB5D1, TRAPCC1, VAMP2, AURKB, ARHGEF15, ODF4*
25	41.0 to 41.1	*CARD11, GNA12*
26	9.5 to 9.8	*PAPSS2, ATAD1, RNSL*

### Detection of putative candidate genes using control chart and varLD methods

Using the LOWESS/control chart approach at the whole-genome level led to the identification of several candidate genes that are involved in numerous biological processes, i.e. lipid and carbohydrate metabolisms (*ACAD11, ACADVL, ADIG, DGAT2, ALDOB, HADH, GAA* and *GPT*)*,* reproduction (*DNAH2, AMHR2, ABCD3, SPEM1, ZAR1* and *SHBG*), cartilage/bone morphogenesis (*LECT2, FLNB, CRTAC1, GDF5, RARG, UQCC, TGFB* and *GLOI*) and biology of the muscle (*PVALB, MYL9, FBXO32* and *CHN2*) (Table 3). Several apoptosis regulatory genes (*APAF1, CARD11, CARD14* and *WDR92*) (Table 3) and several genes that are involved in immune functions and encode proteins that are active in the immune and acute inflammatory responses (*DEFB, CXCL12, PROC* and *PROCR*) (Table 3) were detected. Three signatures of positive selection were identified in regions that contained genes that play a role in heme biosynthesis and transport i.e. *NRF1* on BTA4 at 94 Mb*, ABCB8, ABCF2* and *SMARCD3* on BTA4 at 114 Mb, and *ALAS1* on BTA22 at 49 Mb. [See Additional file 3: Table S3]. Two genes that are involved in the response to oxidative stress (*SOD1* on BTA1 and *VNN2* on BTA9) were detected. Moreover, four different gene families i.e. *ALOX* and *MYH* on BTA19, *HB* on BTA15, and *KRT* on BTA5 were also highlighted. Finally, many genes associated with neurological development and behavioural disorders were pinpointed (*CACNG2, CALN1, ACCN3, EFNB3, DLGAP1* and A*TP1B2*). A complete list of the genes identified by the control chart approach for all 29 bovine autosomes is in Table 3. Using the varLD approach, many candidate genes were detected, among which *APOL3* on BTA5 (between 74 974 805 and 74 986 756 bp) and *LCAT* on BTA18 were the most interesting.

**Table 3:** List of candidate genes identified based on control chart outliers values

BTA	Gene symbol	Gene name	Biological function
1	*ACAD11*	*acyl-CoA dehydrogenase family, member 11*	lipid metabolism
	*CHST2*	*carbohydrate (N-acetylglucosamine-6-O) sulfotransferase 2*	cartilage morphogenesis
	*SOD1*	*superoxide dismutase 1, soluble*	oxidative stress
2	*PROC*	*protein C (inactivator of coagulation factors Va and VIIIa)*	immune response
3	*ABCD3*	*ATP-binding cassette, sub-family D (ALD), member 3*	lipid metabolism
4	*ABCB8*	*ATP-binding cassette, sub-family B (MDR/TAP), member 8*	transport of heme
	*ABCF2*	*ATP-binding cassette, sub-family F (GCN20), member 2*	transport of heme
	*CHN2*	*chimerin 2*	smooth muscle cell proliferation
	*CHPF2*	*chondroitin polymerizing factor 2*	cartilage biosynthesis
	*TWIST1*	*twist family bHLH transcription factor 1*	bone and muscle development
	*NRF1*	*nuclear respiratory factor 1*	heme biosynthesis/transport
	*ACCN3*	*acid-sensing (proton-gated) ion channel 3*	Sensory neuron physiology pain induced by acidosis
	*SMARCD3*	*SWI/SNF related, matrix associated, actin dependent regulator of chromatin, subfamily d, member 3*	heme biosynthesis/transport
5	*CACNG2*	*calcium channel, voltage-dependent, gamma subunit 2*	behavioural disorders
	*CALCOCO1*	*calcium binding and coiled-coil domain 1*	cell growth
	*CHST11*	*carbohydrate (chondroitin 4) sulfotransferase 11*	cartilage biosynthesis
	*RARG*	*retinoic acid receptor, gamma*	skeletal development
	*PVALB*	*parvalbumin*	muscle relaxation
	*APAF1*	*apoptotic peptidase activating factor 1*	apoptosis
	*SOAT2*	*sterol O-acyltransferase 2*	cholesterol metabolism
	*KRTs*	*keratins*	epithelia development
	*AMHR2*	*Anti-Mullerian hormone receptor, type II*	male sex differentiation
6	*GBA3*	*glucosidase, beta, acid 3 (gene/pseudogene)*	flavonoids metabolism
	*HADH*	*hydroxyacyl-CoA dehydrogenase*	lipid metabolism
	*ZAR1*	*zygote arrest 1*	reproduction
7	*LECT2*	*leukocyte cell-derived chemotaxin 2*	cartilage/bone differentiation
	*TGFBI*	*transforming growth factor, beta-induced, 68kDa*	cartilage/bone development
8	*ALDOB*	*aldolase B, fructose-bisphosphate*	carbohydrates metabolism
	*LOXL2*	*lysyl oxidase-like 2*	biogenesis of connective
9	*CTGF*	*connective tissue growth factor*	connective morphogenesis
	*VNN2*	*vanin 2*	stress oxidative response
11	*PROKR1*	*prokineticin receptor 1*	Ca^+2^ mobilization
	*WDR92*	*WD repeat domain 92*	apoptosis modulator
13	*UQCC*	*ubiquinol-cytochrome c reductase complex assembly factor 1*	skeletal development
	*MYL9*	*myosin, light chain 9, regulatory*	muscle metabolism
	*DEFBs*	*defensin beta*	immune response
	*GDF5*	*growth differentiation factor 5*	skeletal development
	*ACSS2*	*acyl-CoA synthetase short-chain family member 2*	lipid metabolism
	*ADIG*	*adipogenin*	adipocyte development
	*PROCR*	*protein C receptor, endothelial*	immune response
	*BPIFB2 BPIF 3 BPIF6*	*BPI fold containing family B, members 2, 3 and 6*	innate immune response
14	*FBXO32*	*F-box protein 32*	muscular diseases
	*GPT*	*glutamic-pyruvate transaminase (alanine aminotransferase)*	liver gluconeogenesis
15	*DGAT2*	*diacylglycerol O-acyltransferase 2*	lipid metabolism
	*TUB*	*tubby bipartite transcription factor*	obesity
	*HBs*	*haemoglobins*	oxygen binding/transport
16	*ACBD3*	*acyl-CoA binding domain containing 3*	reproduction
	*DHRS3*	*dehydrogenase/reductase (SDR family) member 3*	steroids metabolism
	*PLOD1*	*procollagen-lysine, 2-oxoglutarate 5-dioxygenase 1*	connective synthesis
17	*ACADS*	*acyl-CoA dehydrogenase, C-2 to C-3 short chain*	lipid metabolism
18	*ACSF3*	*acyl-CoA synthase family member 3*	lipid metabolism
	*MVD*	*mevalonate (diphospho) decarboxylase*	cholesterol biosynthesis
19	*SPEM1*	*spermatid maturation 1*	reproduction
	*SHBG*	*sex hormone-binding globulin*	steroids metabolism
	*DNAH2*	*dynein, axonemal, heavy chain2*	sperm motility
	*GAA*	*glucosidase, alpha; acid*	carbohydrates metabolism
	*ATP1B2*	*ATPase, Na+/K+ transporting, beta 2 polypeptide*	osmoregulation
	*ACADVL*	*acyl-CoA dehydrogenase, very long chain*	lipid metabolism
	*ALOXs*	*arachidonate-lipoxygenases*	lipid metabolism
	*MYHs*	*myosin, heavy chain skeletal muscle*	muscle biology
	*CARD14*	*caspase recruitment domain family, member 14*	apoptosis
	*EFNB3*	*ephrin B3*	brain development
	*ATP1B2*	*ATPase, Na/K transporting beta 2 polypeptide*	neurite outgrowth
22	*PDHB*	*pyruvate dehydrogenase (lipoamide) beta*	tricarboxylic acid cycle
	*FLNB*	*filamin B, beta*	cartilage/bone morphogenesis
	*TLR9*	*toll-like receptor 9*	immune response
	*OXTR*	*oxytocin receptor*	parturition
	*CAV3*	*caveolin 3*	muscle biology
	*ALAS1*	*aminolevulinate, delta-, synthase 1*	biosynthesis of heme
	*ACOX2*	*acyl-CoA oxidase 2, branched chain*	lipid metabolism
23	*MLN*	*motilin*	control of peristalsis
	*GLO1*	*glyoxalase*	osteoclastogenesis
24	*DLGAP1*	*disc, large (Drosophila) homolog-associated protein 1*	postsynaptic scaffold in neuronal cell
25	*CARD11*	*caspase recruitment domain family, member 11*	apoptosis
	*CALN1*	*calneuron 1*	memory and learning
26	*LOXL4*	*lysyl oxidase-like 4*	biogenesis of connective tissue
	*CRTAC1*	*cartilage acidic protein 1*	cartilage production
28	*CXCL12*	*chemokine (C-X-C motif) ligand 12*	immune response

Moreover, several members of the *LT/LBP* gene family were identified by both methods on BTA13 with seven isoforms detected by varLD (*BPIFA2A, BPIFA2C, BPIFA2B, BPIFA3, BPIFA1, BPIFB1* and *BPIFB5*) and three by control chart (*BPIFB2, BPIFB6* and *BPIFB3*).

## Discussion

The two bovine breeds, Piemontese and Marchigiana, that were included in this study share similar phenotypic and production characteristics, but have different origins and breeding histories [1, 37]. The Piemontese breed is raised in Northern Italy and derives mainly from *Bos brachyceros* [38]. In the past, it was considered as a triple aptitude breed (draught, milk and meat) and, over time, its ability to produce meat has improved while maintaining a high level of milk production. The Marchigiana breed is raised in Central Italy and its origin can be traced backed to *Bos primigenius* [37]. Initially, it was considered as a dual-purpose breed (draught and meat) but because of its high capacity for muscle growth, it has become specialized as a beef breed. It was recognized as a separate breed at the beginning of the last century when crosses with Chianina bulls were stopped.

In our study, the Marchigiana breed showed a slightly lower level of chromosome heterozygosity than the Piemontese breed (Fig. 1), which confirms the findings reported by Bozzi et al. [39] who analyzed the genetic diversity of several beef cattle breeds and found that the Marchigiana breed had the lowest level of genetic diversity. This is probably due to the breeding policy conducted by farmers, but also to the small size of the breed. The genetic distances between the Piemontese and Marchigiana breeds that we report here are compatible with data from the literature. In fact, several studies showed that cattle breeds with similar production aptitudes display some genetic variability [40–45]. In our case, although the Piemontese and Marchigiana breeds share similar phenotypic and production characteristics, the genetic architectures of the traits differ. Indeed, we identified genes that are usually found when breeds with divergent artificial selection are compared (*DGAT1* on BTA14 and *ABCG2* on BTA6) [22, 46], but also polymorphisms in genes that control phenotypes that are specific to each breed (*MSTN* and *FTO*) (Table 1).

Another interesting finding of our study on these two breeds is the identification of selection signatures in genomic regions that are known to harbour candidate genes for dairy traits, such as *DGAT1* and *ABCG2*. However, some authors suggested that these genes may also have a role in beef cattle breeds [47–49]. Different causative polymorphisms may explain the detection of *DGAT1* and *ABCG2*. Regarding *DGAT1,* allele K is fixed in the Piemontese breed whereas allele A is most probably present in the Marchigiana breed [50] since in the past it was crossed with the Chianina breed that possesses this allele. Finally, Ron et al. [51] showed that the polymorphism of the *ABCG2* gene differs among beef cattle breeds.

The detection of *MSTN* was rather unexpected in this comparison between the Piemontese and Marchigiana breeds. Two possible explanations are: (1) muscular hypertrophy in each of these breeds is caused by different mutations in the *MSTN* gene, i.e. a G > T transversion that introduces an early stop codon in the third exon of the gene [52] is present in the Marchigiana breed whereas a G > A transition in the same exon [53] is found in the Piemontese breed; or (2) the mutation responsible for muscle hypertrophy is fixed in the Piemontese breed [54] while its frequency is low in the Marchigiana breed [52] because of different selection strategies applied in each breed. The two candidate genes, *UQCC* and *GDF5* detected on BTA13 contain polymorphisms that are associated with stature in humans [55] and with body size determination in European *Bos taurus* cattle [56] (Table 4). In general, Piemontese bulls are smaller (height = 135 cm, and weight = 850 kg) (www.anaborapi.it) than many other beef breeds [57], including the Marchigiana breed (height = 140 cm, and weight = 1.200 kg) (www.anabic.it). Moreover, the *GHRH* gene, which is responsible for the release of growth hormone, is located in the same region of BTA13 (Table 1), which is consistent with the identification of a QTL (quantitative trait locus) in this chromosomal region in a genome-wide association study on several beef cattle breeds [58].

**Table 4:** Genes detected in this work and previously reported by other authors for beef cattle

Gene symbol	Author
*NCAPG*	[79]
*LAP3, LCORL*	[13]
*LCT*	[80]
*ASGR1, DGAT2, HNF1A, SOAT2, TGFB1, GNLY, POP5, MYH8*	[67, 81]
*COL3A1, MYH1*	[82]
*ATP5L, ACAD11, ACY1, ACSS2, ALDOB, ACADVL*	[83]
*ASNSD1, INPP1, ORMDL1, OSGEPL1, PMS1*	[84]
*SAMHD1, TLR9*	[6]
*UQCC, GDF5*	[56]
*MCM6, DARS, UBXN4*	[85]

A primary goal for livestock industry is to produce high-quality meat for human consumption. Thus, it is necessary to know and understand which factors influence the transformation of muscle to meat, which depends mainly on the intrinsic properties of the myofibers (type, composition and size), enzymatic proteolytic activities (cathepsin and calpain-calpastatin system) [59, 60] but also on structural features such as connective tissue and intramuscular fat deposition [61]. It is generally recognized that the process of meat tenderization is the result of biochemical reactions that involve both proteolytic and apoptotic pathways [60, 61]. In our study, we identified several genes that are involved in the positive regulation of cellular apoptosis in skeletal muscle such as *APAF1, CARD11, CARD14* and *WDR92*. Numerous genes that have a role in lipid metabolism (*ACAD11, ABCD3, HADH*, *ACSS2, DGAT2* and *ACADS*), cholesterol and steroids synthesis (*SOAT2, DHRS3, SHBG* and *MVD*), and in the biology of adipose tissue (*ADIG* and *TUB*) were detected. One possible explanation may be related to the distinctive metabolism of adipose tissue in the Piemontese breed compared to other beef breeds. A comparative analysis of young bulls from various European breeds (including Marchigiana) showed that the Piemontese breed had the lowest scores for carcass traits such as fatness and fat percentage [62]. Another comparison between Piemontese, Red Angus and Gelbvieh breeds revealed that the Piemontese breed also had the lowest score for fat thickness [63]. Moreover, the cholesterol level of Piemontese meat is lower than that of other cattle breeds (Piemontese Breed Consortium www.coalvi.it) or other livestock species (Piemontese Association of the United States).

**Fig. 4:**
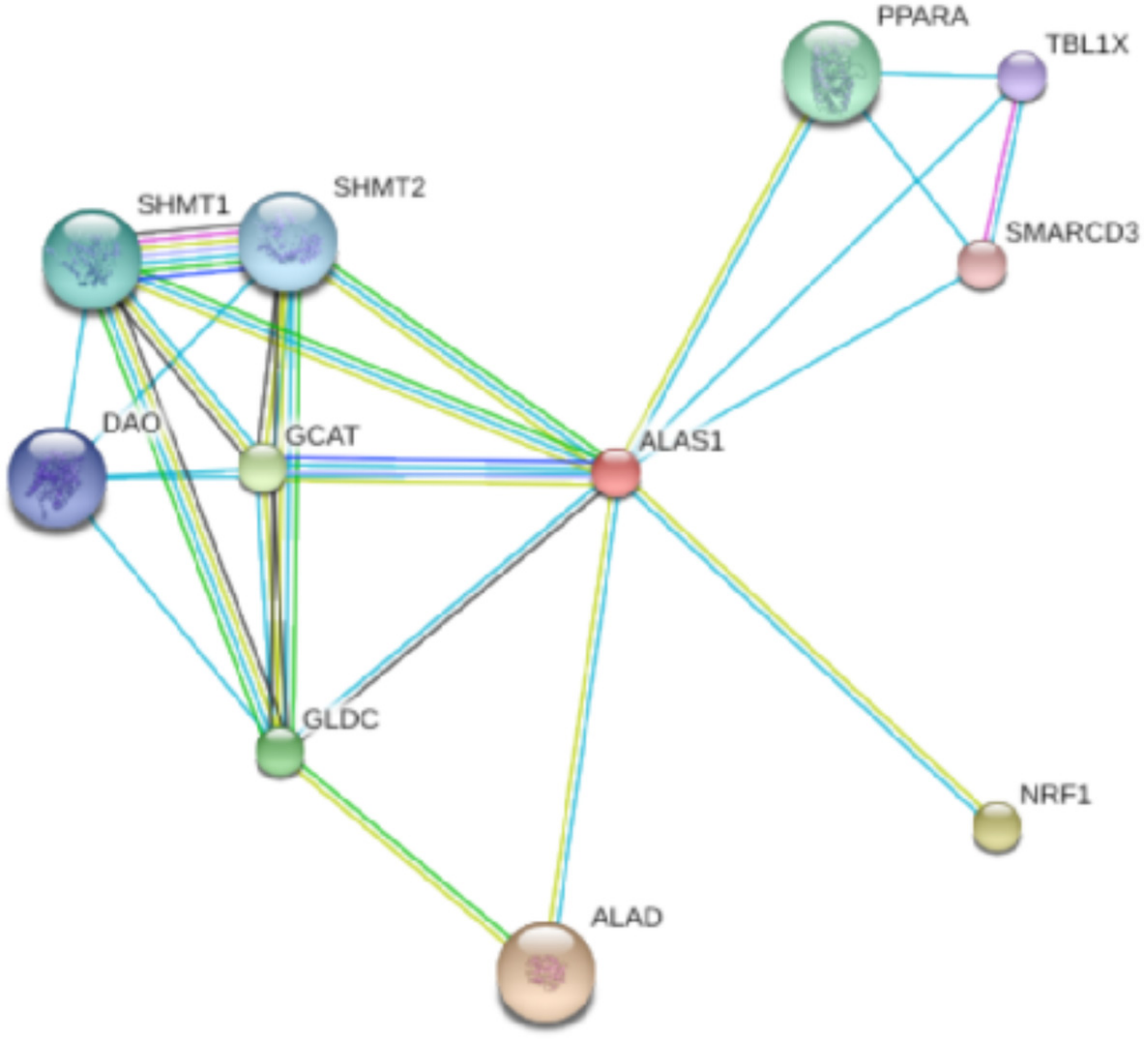
Protein network of bovine ALAS1 according to STRING 9.0 action view. Nodes are proteins; lines indicate interactions between proteins with: pink lines for post-translational, yellow lines for expression, black lines for reaction, blue lines for binding and light blue lines for phenotype. Protein interactions include direct (physical) and indirect (functional) associations derived from different sources (genomic context, high through-put experiments, conserved coexpression, previous knowledge). 0.91 and 0.89 are the confidence values for the products of *NRF1* and *SMARCD3*, respectively

The Marchigiana breed is considered as a hardy breed with excellent adaptability to pasture in harsh environments (www.agraria.org). Genes that are involved in the triggering and regulation of innate immune responses such as *chemokines* (*CXCL12*) [64], *defensins* (*DEFB119*, *122*, *122A*) and *toll-like receptors* (*TLR9*) were detected in our study, which is consistent with the high level of resistance to diseases and endoparasites of this breed. Among these genes, *TLR9* was previously reported by Ramey et al. [6] (Table 3) and a comparative analysis of 16 European breeds showed that three *TLR* genes contained fixed polymorphisms in the Marchigiana breed whereas only rare alleles were found at the same SNPs in the Piemontese breed [65]. Meat quality can be modulated by genes that are involved in acute inflammatory response processes [66]. Cytokines are a large family of soluble molecules (that also include chemokines) that regulate the inflammatory response. Recently, it was shown that genes that control immune and acute stress responses also play a role in the determination of beef tenderness [61, 67, 68]. A comparison between different types of beef cattle revealed that genes that encode immune response proteins are deregulated in situations of stress such as diseases, which suggests that they play an important role in muscle metabolism and influence final beef tenderness [67, 69]. Meat tenderness is measured by the force required to cut through a piece of meat i.e. the greater is the force required, the tougher is the meat. This is known as the Warner-Bratzler shear force test, which is the most popular method to measure the tenderness of the meat [70, 71]. A comparison of the tenderness of the *longissimus dorsii* muscle from Chianina, Piemontese, Marchigiana, Limousine and Charolais bulls showed that the Warner-Bratzler shear force values were lowest for the Piemontese samples [72]. Most interesting was the identification of genes that play a role in the biology of erythrocytes. Hemoglobin and myoglobin are the two main heme proteins that are responsible for oxygen binding and transport [69]. In this study, six genes (*NRF1*, *SMARCD3*, *ALAS1*, *HB*, *ABCF2* and *ABCB8*) involved in the metabolism of heme were identified (Table 3). *ALAS1* is a housekeeping gene that encodes a mitochondrial enzyme that catalyzes heme biosynthesis. Fig. 4 shows the relationships between bovine *ALAS1* and other proteins that were determined by data integration in the STRING 9.0 database. The highest confidence values (0.91 and 0.89) were found between *ALAS1* and *NRF1* and between *ALAS1* and *SMARCD3*, respectively, which indicate a direct interaction. This is in agreement with Chambaz and co-workers [73] who reported greater heme/iron content in Piemontese meat than in the meat of other European beef cattle breeds. In addition to the genes that control heme content, or modulate the response to various stressors or determine the structure and composition of myofibers, particular attention should be addressed to those that influence oxidative stress. Oxidative stress is considered as a metabolic disturbance that affects the health status but also the quality of the final animal products [74]. Overall, information on the link between oxidative stress and meat quality is scarce and conflicting and, for cattle, there is no direct evidence that the genetic background has an influence on oxidative stress and meat characteristics [75]. Oxidative damage to tissues is prevented by various factors such as non enzymatic antioxidant molecules (vitamins, polyphenols and thiols) and enzymes that are incorporated within the cell membranes [76] with superoxide dismutase, catalase and glutathione peroxidase being the most important antioxidative enzymes [74]. In our study, selective sweeps were highlighted in the chromosomal region that contains the *SOD1* gene.

The presence of smoothed F_st_ peaks that reached but did not exceed the upper limit of the control chart (borderline peaks) on several autosomes [See Additional file 2: Figure S1] suggests recent selection. In general, selection acts in genomic regions that have a functional importance, thus, identification of genes that have undergone recent selection or that are currently under selection is relevant [77]. An incomplete selective sweep appears in a population when the frequency of a favourable allele of a gene is increasing but has not reached fixation yet. In addition, Schwarzenbacher et al. [5] and Ramey et al. [6] identified selective sweeps, which, although they had not reached fixation, represented potentially interesting regions for cattle. Our comparison between the LOWESS/control chart and the varLD approaches to detect selection signatures provided concordant results for 16 of the 29 bovine autosomes. Such incomplete agreement is rather frequent in studies on the detection of selection signatures [78]. The LOWESS/control chart method detected more significant markers than the varLD method. In addition, when both methods detected the same chromosomal regions and candidate genes, as for *MSTN* on BTA2 and *ABCG2* on BTA6, some of the significant markers did not coincide. The reasons for these discrepancies are probably due to differences between the two methods, in particular in the metrics used. On the one hand, the LOWESS/control chart approach is based on the analysis of differences in allele frequencies at a single locus, although the LOWESS smoothing corrects for local variation. On the other hand, the varLD method relies on the difference in correlation structures among markers located in the same window between two populations and the SNP that is flagged as the most significant is the SNP that is located nearest to the centre of the window [31]. Therefore, for the same genomic region, the LOWESS/control chart approach can detect a larger number of significant markers than the varLD method. For example, for the region that contains *MSTN* on BTA2, the LOWESS/control chart approach detected 10 markers (between 4.9 and 6.6 Mb), whereas only three significant SNPs (between 5.6 and 5.8 Mb) were identified by the varLD method. Another discrepancy between the two methods was the different positions of some detected peaks, which may be due to how signals are averaged or smoothed and/or to the rationale used to assess significant markers.

## Conclusions

Phenotypic variability is the basis of most comparative studies conducted in all living beings. In this study, we showed that, even when phenotypic diversity is not sufficiently large to be detected, investigating the polymorphisms that are present in the regions of the genome that are involved in breeding traits can be very useful in terms of genetic improvement. Our results highlight interesting genomic differences between two cattle breeds that share the same production aptitudes. These variations were located in regions that contain both genes known to affect production traits (in both beef and dairy cattle) and new candidate genes. In many cases, F_st_ revealed clear differences but borderline values also flagged regions where selection is currently acting. We detected genes that are involved in different metabolic pathways. This finding confirms the great complexity of the mechanisms that underlie quantitative traits, which can show genetic variation even among breeds that are phenotypically similar. With the aim of increasing both the quality and quantity of meat in bovine breeds, it would be interesting to analyze in detail the genes that we identified (such as *SOD1, LOXL2, CAV3, ACADS, CXCL12, MYL9, MVD, TLR9* and *ALAS1*) in order to include them in selection programs. Therefore, to increase breed performance and reveal how selection shapes the genome, it is essential to dissect the genetic architecture of each population.

## Additional files

Additional file 1: Table S1. List of chromosomal specific smoothing parameters *S*. Table S1 provides the LOWESS smoothing parameters that were calculated for each chromosome.

Additional file 2: Figure S1. Patterns of raw F_st_ data, predicted F_st_ values and control chart of predicted F_st_ values on BTA1 to 29. Description: The plots represent the pattern of raw F_st_ data calculated for SNPs located on each chromosome (BTA), the predicted F_st_ values for SNPs located on each BTA using the LOWESS regression with chromosomal specific smoothing parameter and the control chart of predicted F_st_ values for each BTA. Upper control limit (UCLI) and lower control limit (LCLI) are three standard deviations apart from the mean value.

Additional file 3: Table S2. List of 933 genes annotated in cattle detected using the control chart method. Description: This list includes all the bovine annotated genes derived from *Bos taurus* UMD 3.1/bosTau6 assembly that are present in the 0.5 Mb interval (0.25 Mb upstream and downstream) considered for each significant SNP using the control chart method.

Additional file 4: Table S3. List of 189 genes annotated in cattle detected using varLD. Description: This list includes all the bovine annotated genes derived from *Bos taurus* UMD 3.1/bosTau6 assembly that are present in the 0.5 Mb interval (0.25 Mb upstream and downstream) considered for each significant SNP using the varLD approach.

Additional file 5: Figure S2. Plots obtained with the F_st_ method *vs* the varLD method. Description: Chromosome-wide plots obtained in the F_st_ and varLD analyses for BTA1 to 29. Solid lines represent the threshold set at three standard deviations apart from the mean value.
