# Supplementary material for: Detection of selection signatures in Piemontese and Marchigiana cattle, two breeds with similar production aptitudes but different selection histories
Source: Genet Sel Evol. 2015 Jun 23;47(1):52. doi: 10.1186/s12711-015-0128-2 (PMC4476081; doi:10.1186/s12711-015-0128-2)
Supplement: Supplementary file 1 — List of chromosomal specific smoothing parameters S. Table S1 provides the LOWESS smoothing parameters that were calculated for each chromosome. [file 12711_2015_128_MOESM1_ESM.pdf]

BTA	Length (bp)	SNP number	Smoothing parameter S	FST
1	158161585	2806	0,00713	0,43503
2	136531159	2300	0,00870	0,47124
3	121403393	2151	0,00930	0,27933
4	120641946	2097	0,00954	0,29346
5	119729902	1770	0,01130	0,46225
6	119396337	2149	0,00931	0,44806
7	112610067	1878	0,01065	0,44509
8	113367096	1995	0,01003	0,32658
9	105351459	1692	0,01182	0,24532
10	104215086	1803	0,01109	0,32622
11	107043330	1886	0,01060	0,33668
12	91091598	1377	0,01452	0,28642
13	84148909	1491	0,01341	0,54780
14	84616190	1448	0,01381	0,32498
15	85049720	1417	0,01411	0,30955
16	80924801	1317	0,01519	0,31732
17	74998349	1358	0,01473	0,28150
18	65978584	1127	0,01775	0,40787
19	64007021	1152	0,01736	0,34630
20	71793734	1338	0,01495	0,27074
21	70608408	1172	0,01706	0,37591
22	61378199	1098	0,01821	0,28489
23	52128894	910	0,02198	0,26731
24	62643699	1082	0,01848	0,27364
25	42851121	818	0,02445	0,27863
26	51638650	890	0,02247	0,25875
27	45368987	808	0,02475	0,18559
28	46194755	797	0,02509	0,17805
29	51124356	882	0,02268	0,19822
