# Supplementary material for: Detection of selection signatures in Piemontese and Marchigiana cattle, two breeds with similar production aptitudes but different selection histories
Source: Genet Sel Evol. 2015 Jun 23;47(1):52. doi: 10.1186/s12711-015-0128-2 (PMC4476081; doi:10.1186/s12711-015-0128-2)
Supplement: Supplementary file 2 — Patterns of raw Fst data, predicted Fst values and control chart of predicted Fst values on BTA1 to 29. Description: The plots represent the pattern of raw Fst data calculated for SNPs located on each chromosome (BTA), the predicted Fst values for SNPs located on each BTA using the LOWESS regression with chromosomal specific smoothing parameter and the control chart of predicted Fst values for each BTA. Upper control limit (UCLI) and lower control limit (LCLI) are three standard deviations apart from the mean value. [file 12711_2015_128_MOESM2_ESM.pdf]

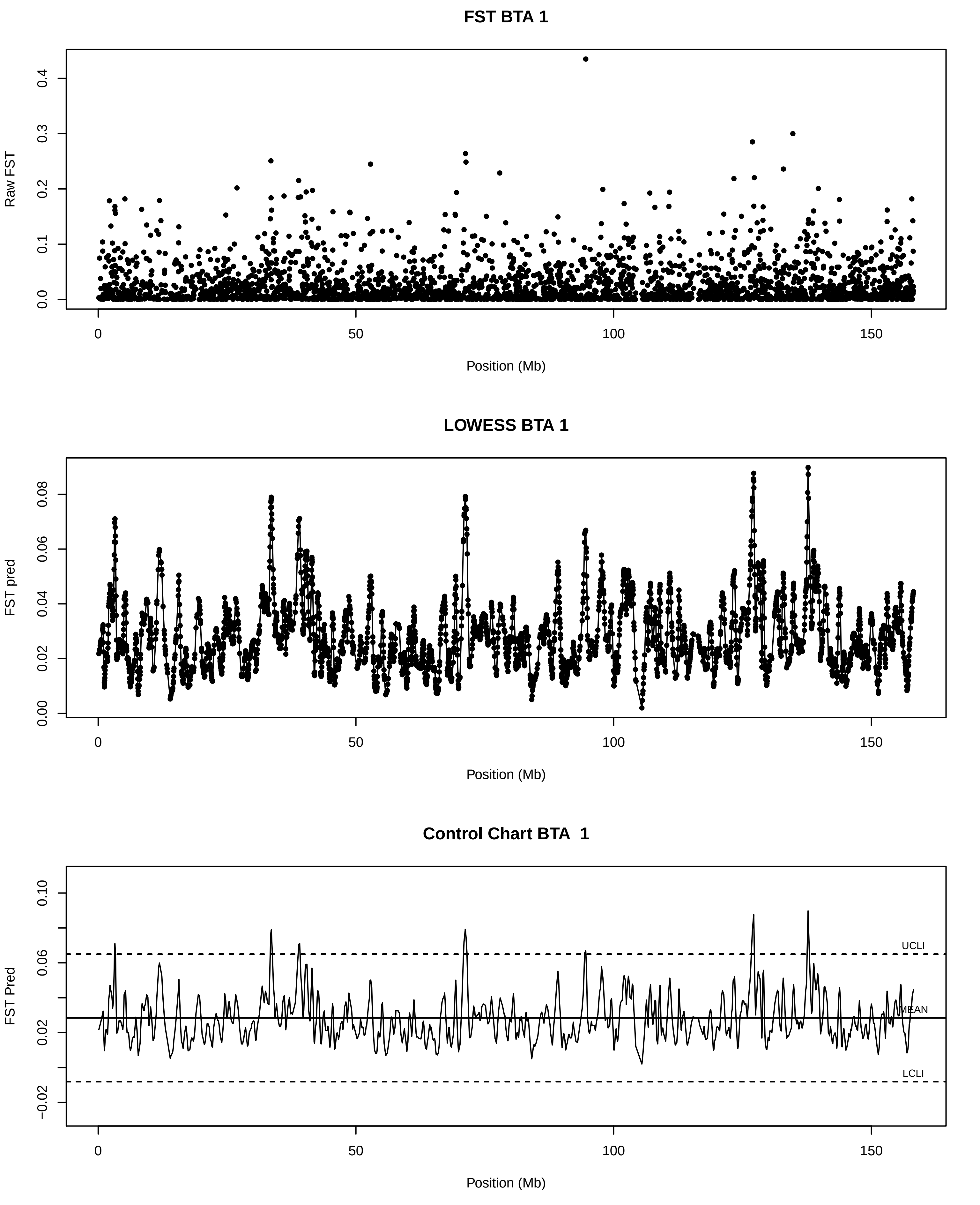
FST BTA 1
Raw FST
0.0
0.1
0.2
0.3
0.4
0
50
100
150
Position (Mb)
LOWESS BTA 1
FST pred
0.00
0.02
0.04
0.06
0.08
0
50
100
150
Position (Mb)
Control Chart BTA 1
FST Pred
−0.02
0.02
0.06
0.10
UCLI
MEAN
LCLI
0
50
100
150
Position (Mb)

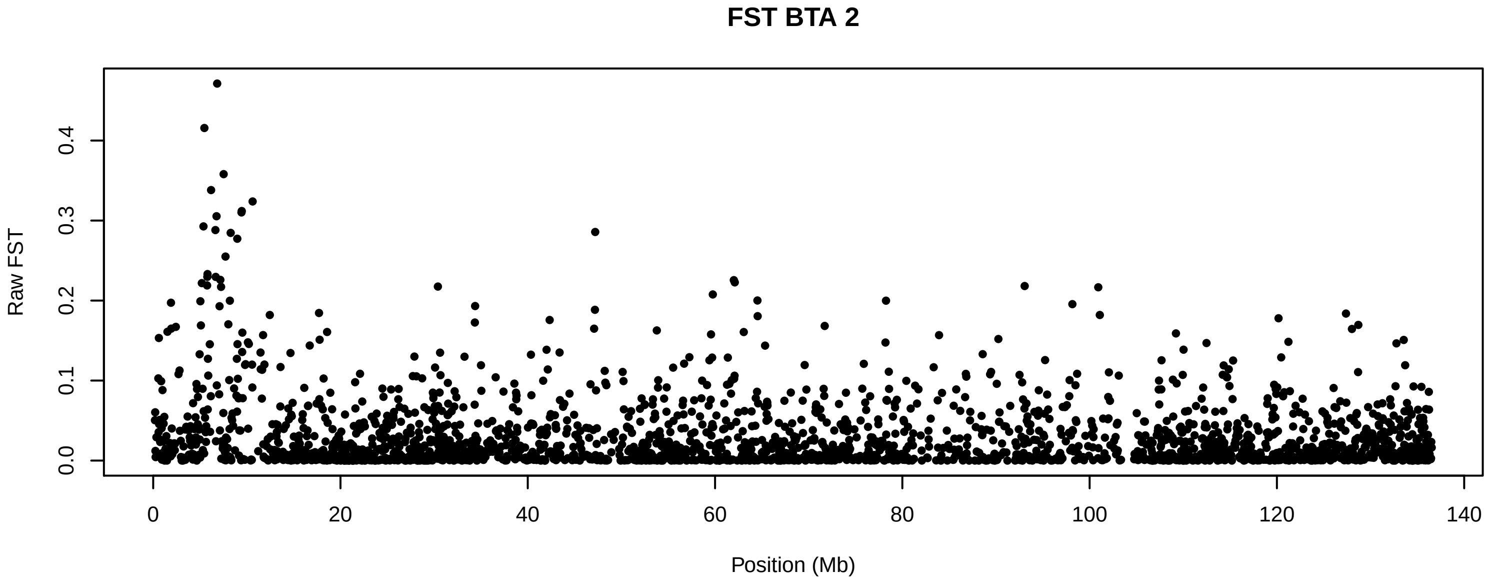
FST BTA 2
Raw FST
Position (Mb)
0.0
0.1
0.2
0.3
0.4
0
20
40
60
80
100
120
140

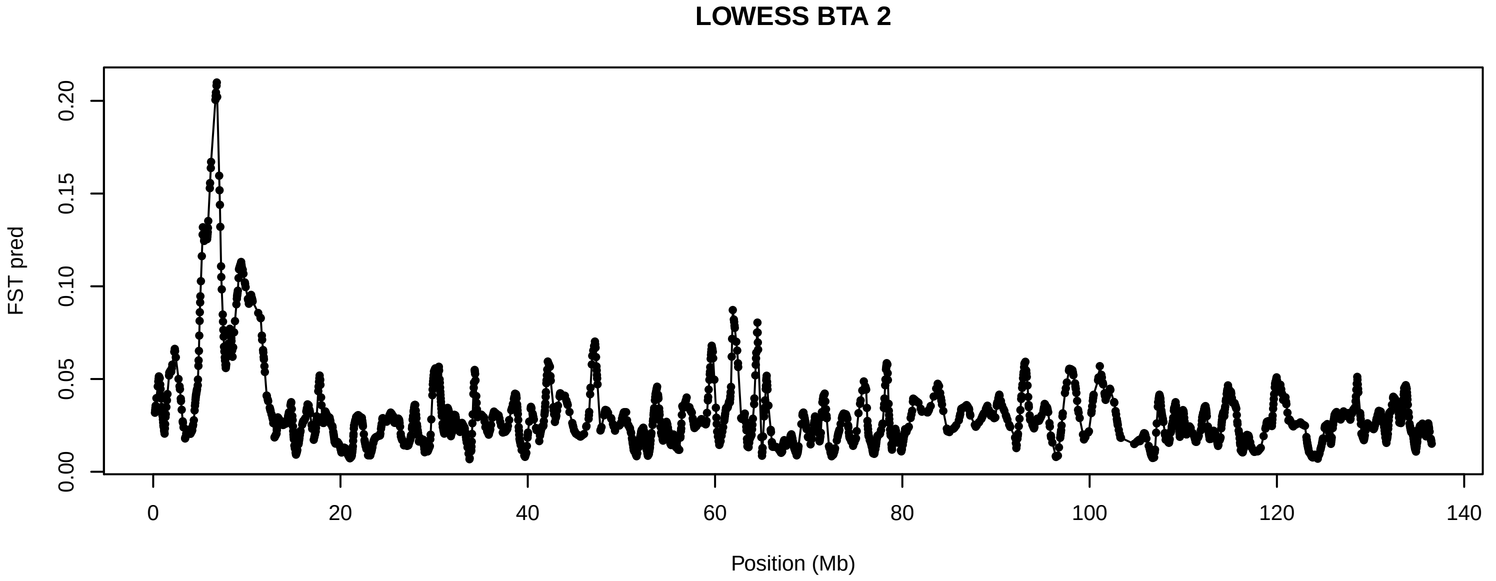
LOWESS BTA 2
FST pred
Position (Mb)
0.00
0.05
0.10
0.15
0.20
0
20
40
60
80
100
120
140

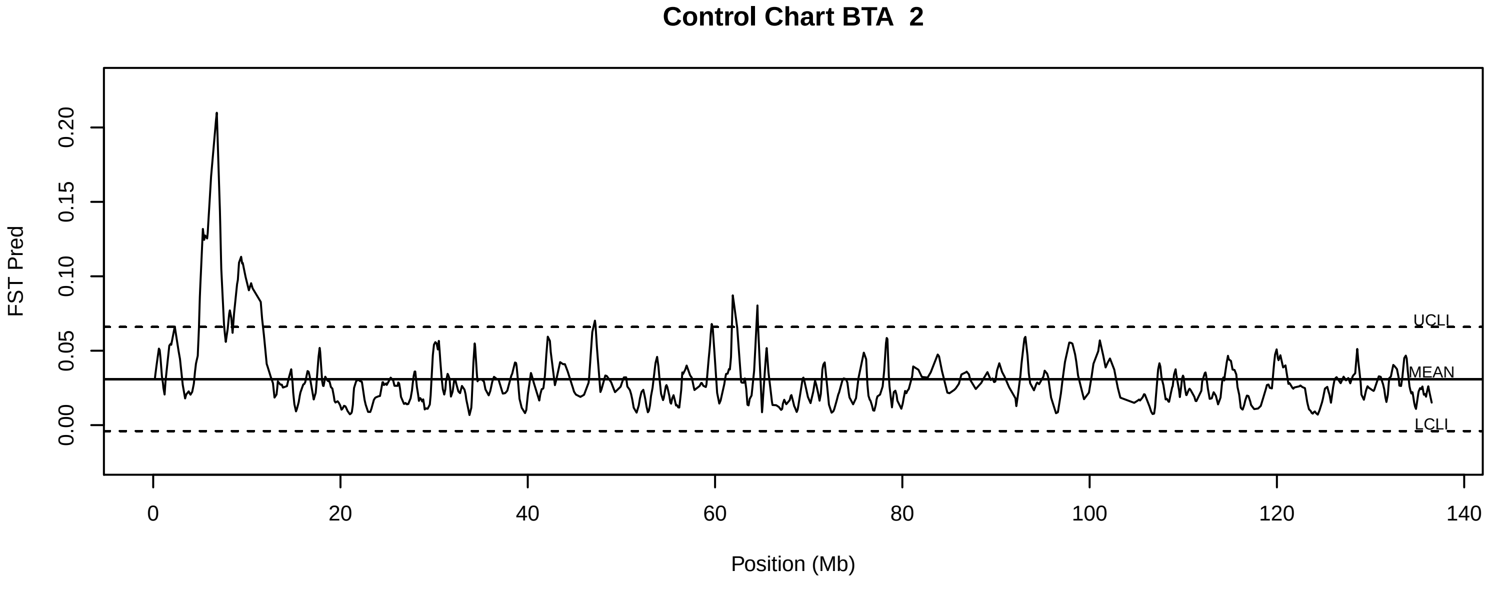
Control Chart BTA 2
FST Pred
Position (Mb)
UCLL
MEAN
LCLL
0.00
0.05
0.10
0.15
0.20
0
20
40
60
80
100
120
140

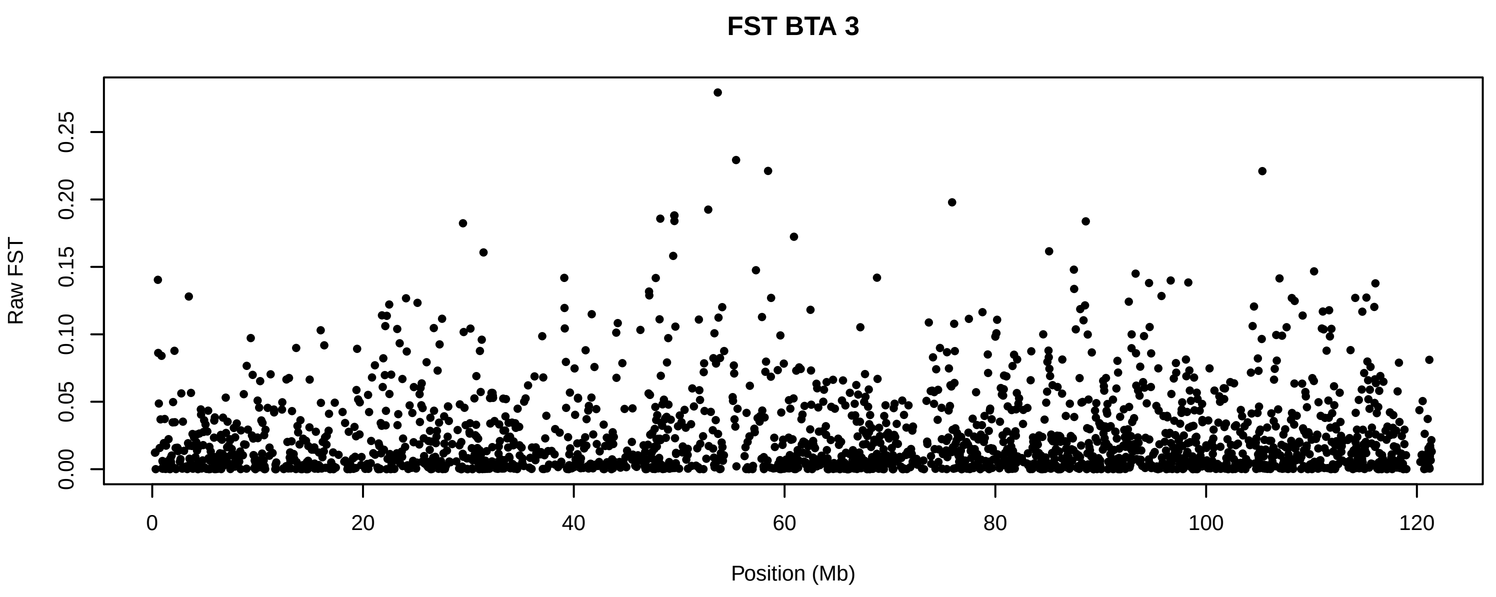
FST BTA 3
Raw FST
0.00
0.05
0.10
0.15
0.20
0.25
0
20
40
60
80
100
120
Position (Mb)

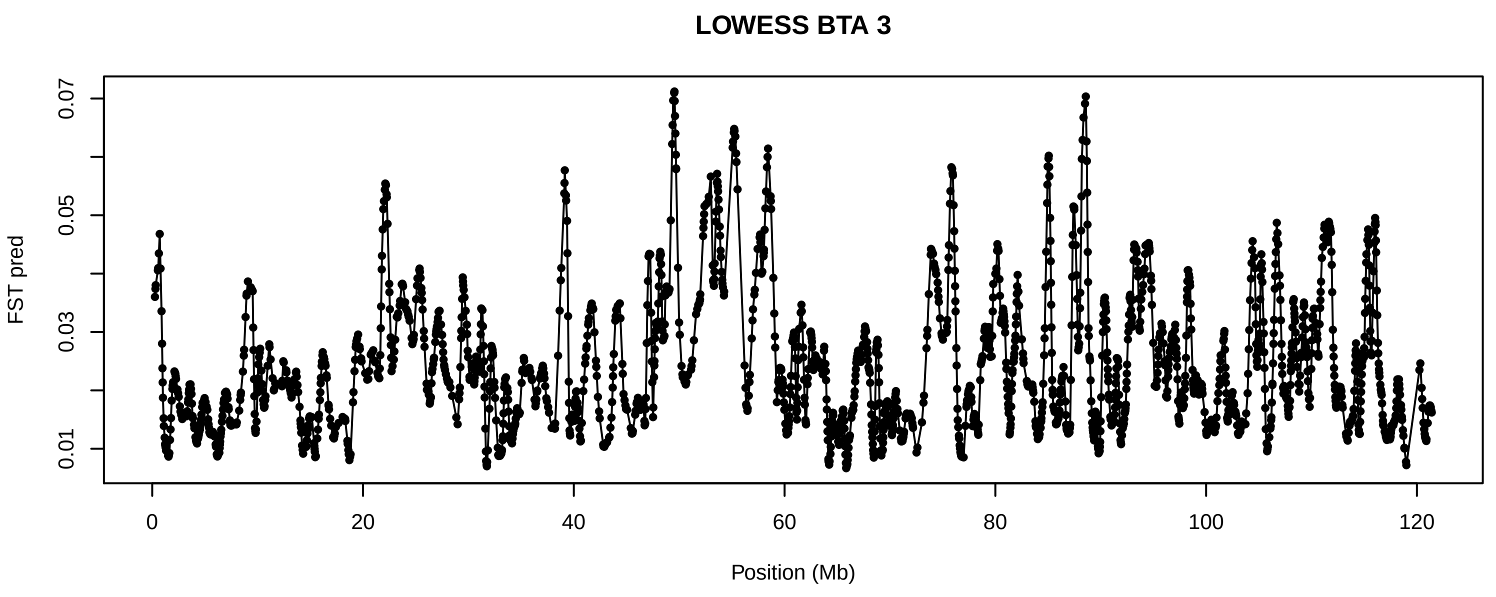
LOWESS BTA 3
FST pred
0.01
0.03
0.05
0.07
0
20
40
60
80
100
120
Position (Mb)

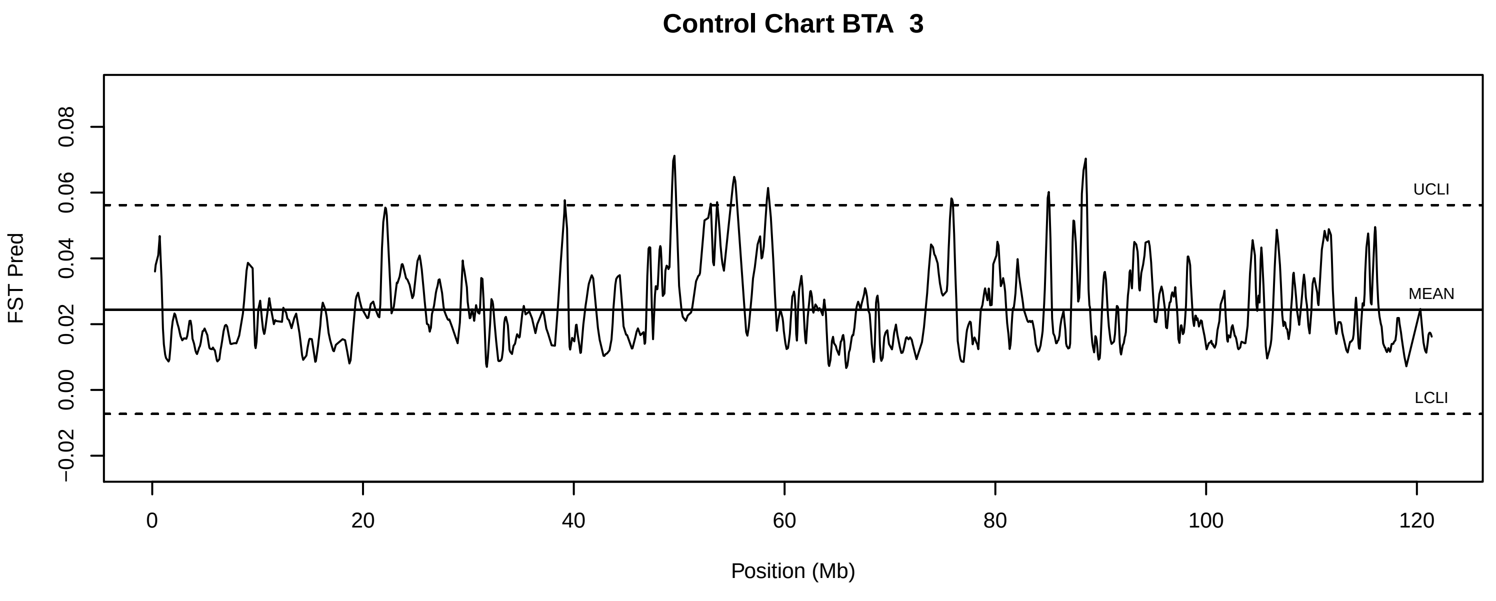
Control Chart BTA 3
FST Pred
−0.02
0.00
0.02
0.04
0.06
0.08
UCLI
MEAN
LCLI
0
20
40
60
80
100
120
Position (Mb)

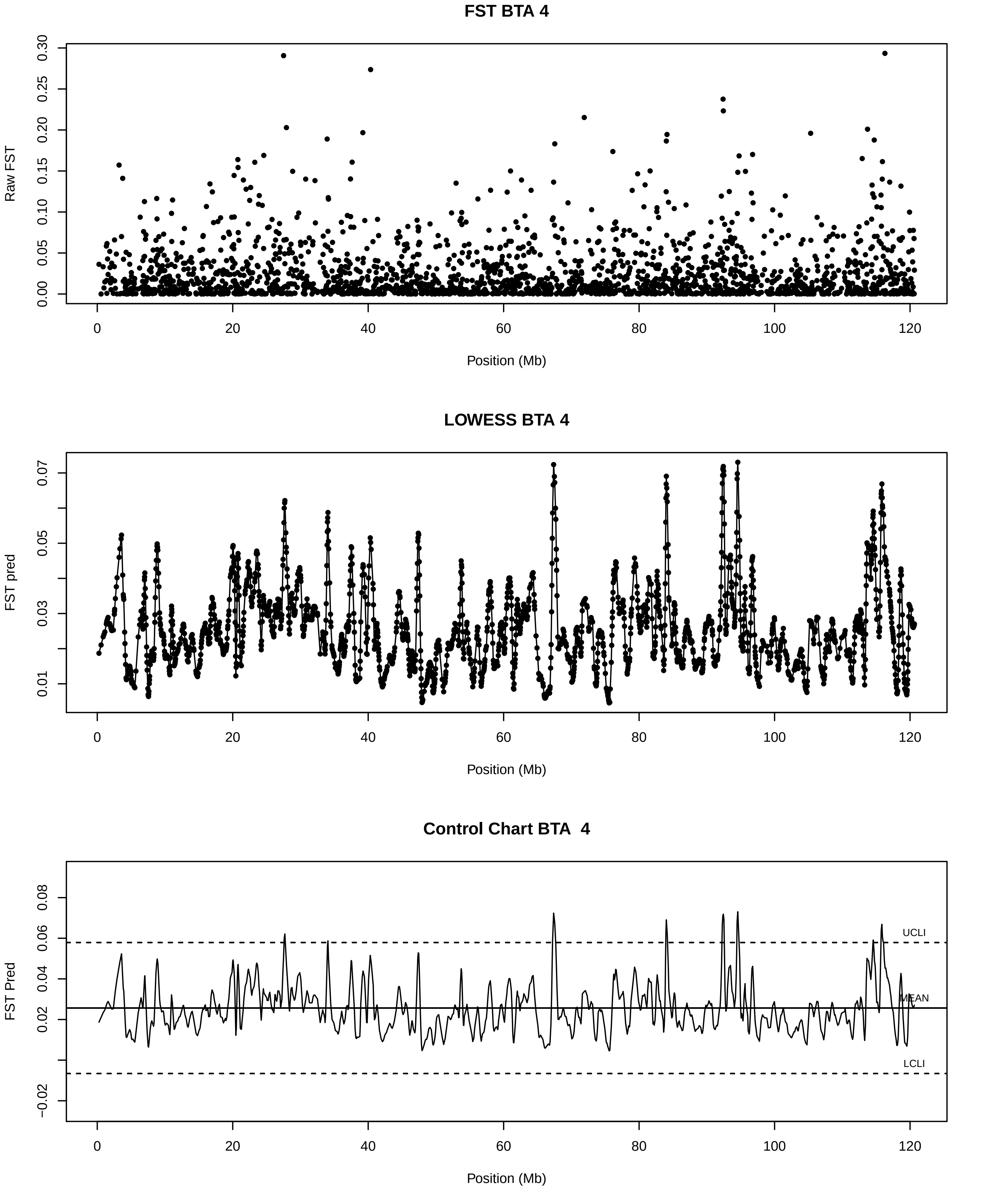
FST BTA 4
Raw FST
0.00
0.05
0.10
0.15
0.20
0.25
0.30
0
20
40
60
80
100
120
Position (Mb)
LOWESS BTA 4
FST pred
0.01
0.03
0.05
0.07
Position (Mb)
Control Chart BTA 4
FST Pred
−0.02
0.02
0.04
0.06
0.08
UCLI
MEAN
LCLI
Position (Mb)

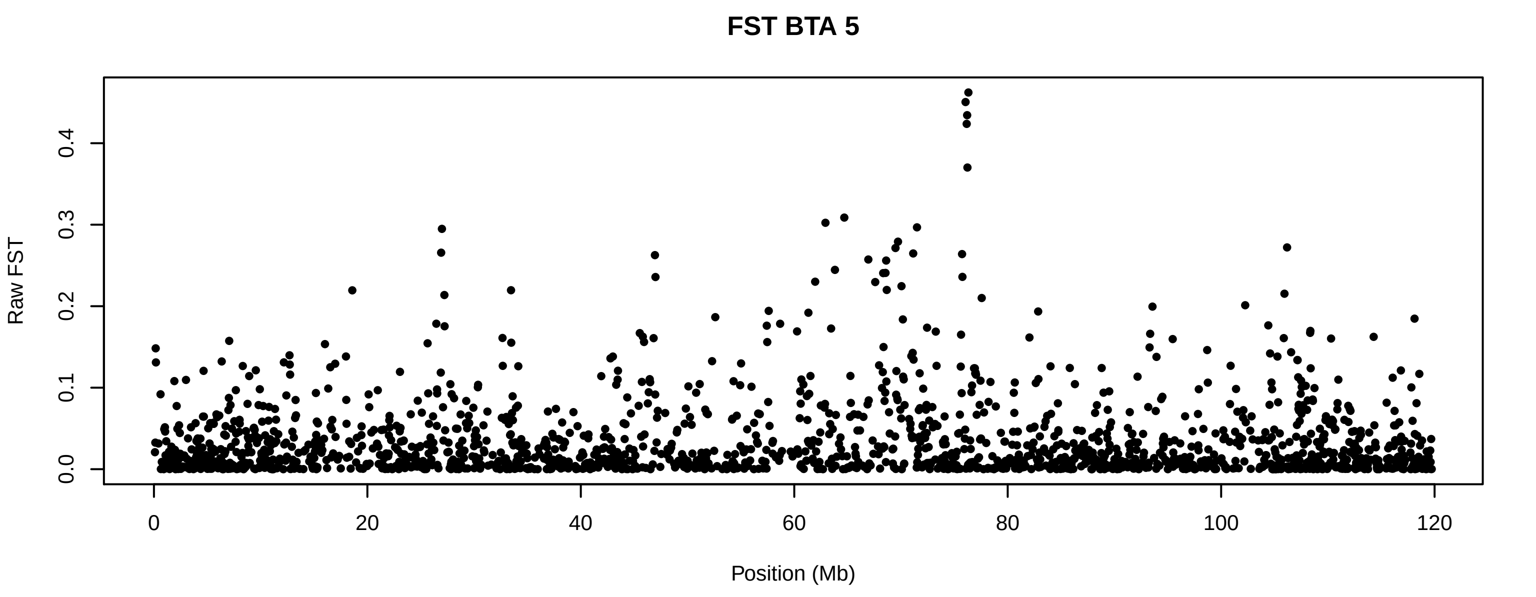
FST BTA 5
Raw FST
0.0
0.1
0.2
0.3
0.4
0
20
40
60
80
100
120
Position (Mb)

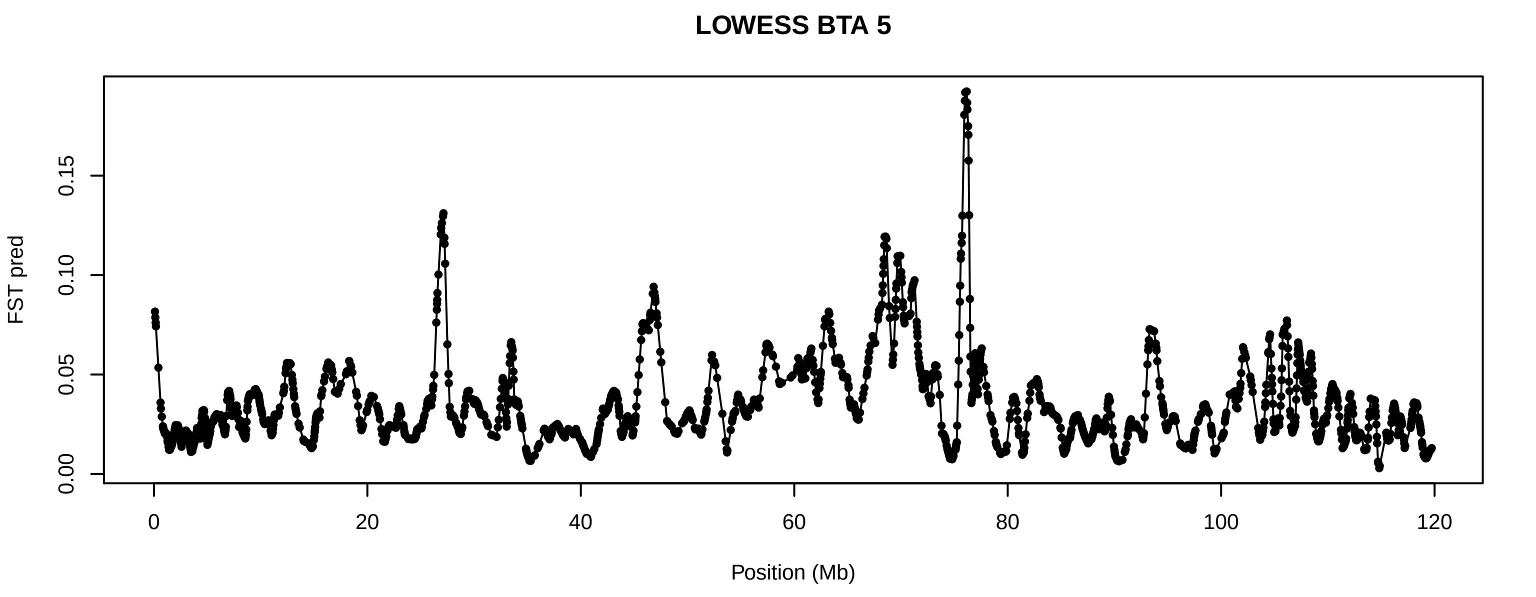
LOWESS BTA 5
FST pred
0.00
0.05
0.10
0.15
0
20
40
60
80
100
120
Position (Mb)

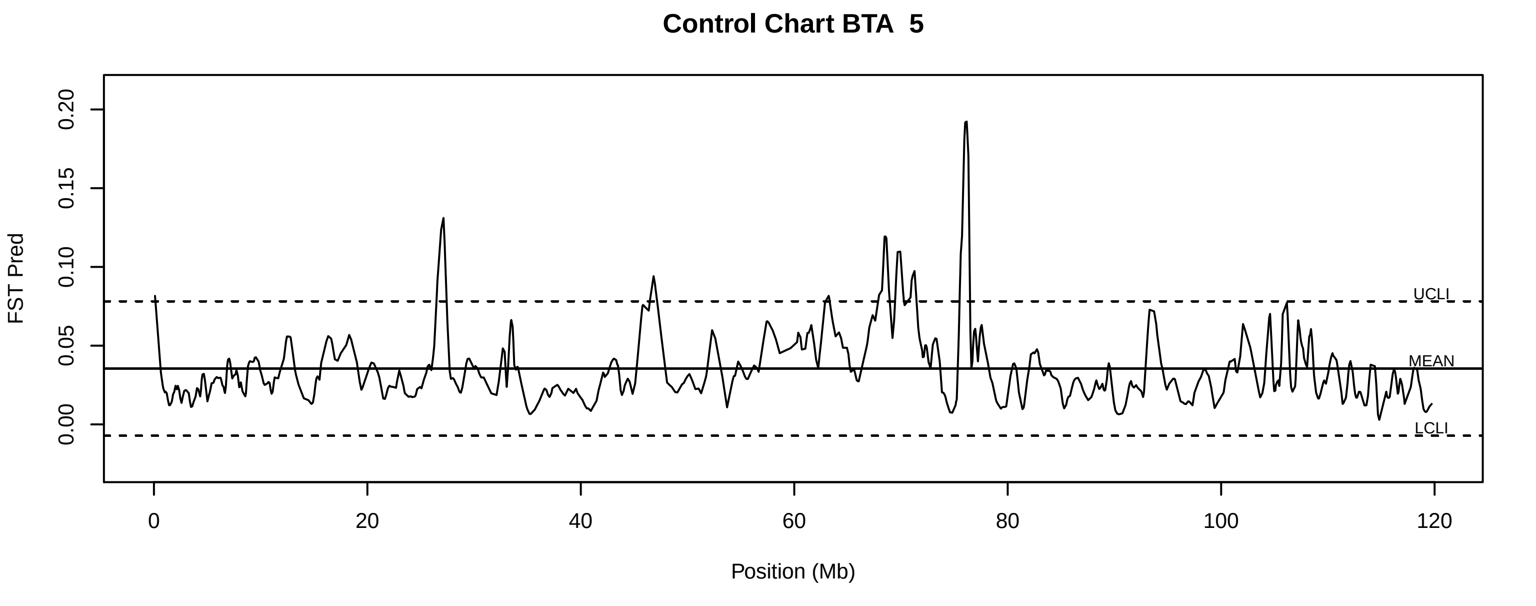
Control Chart BTA 5
FST Pred
0.00
0.05
0.10
0.15
0.20
UCLI
MEAN
LCLI
0
20
40
60
80
100
120
Position (Mb)

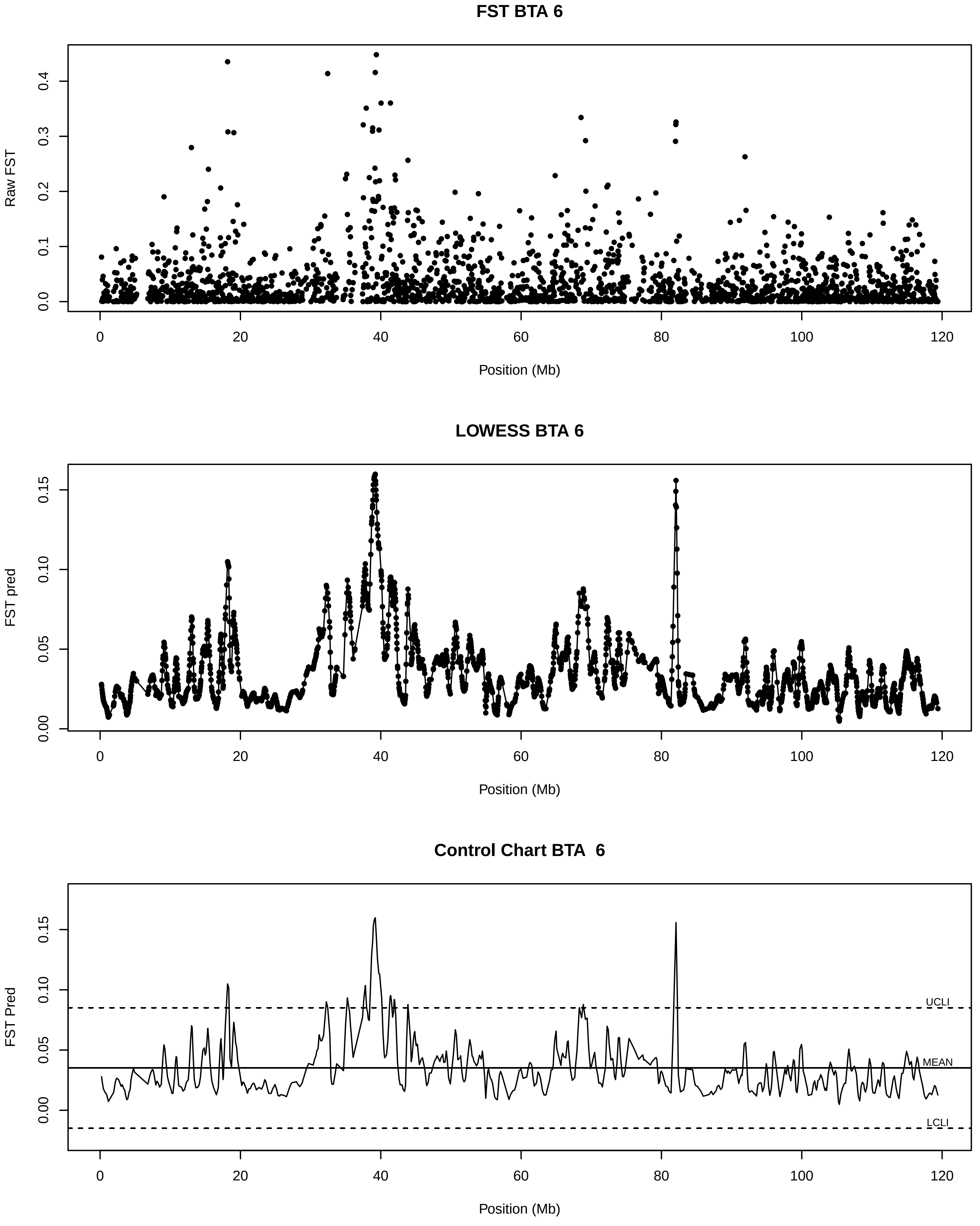
FST BTA 6
Raw FST
0.0
0.1
0.2
0.3
0.4
0
20
40
60
80
100
120
Position (Mb)
LOWESS BTA 6
FST pred
0.00
0.05
0.10
0.15
Position (Mb)
Control Chart BTA 6
FST Pred
UCLI
MEAN
LCLI
Position (Mb)

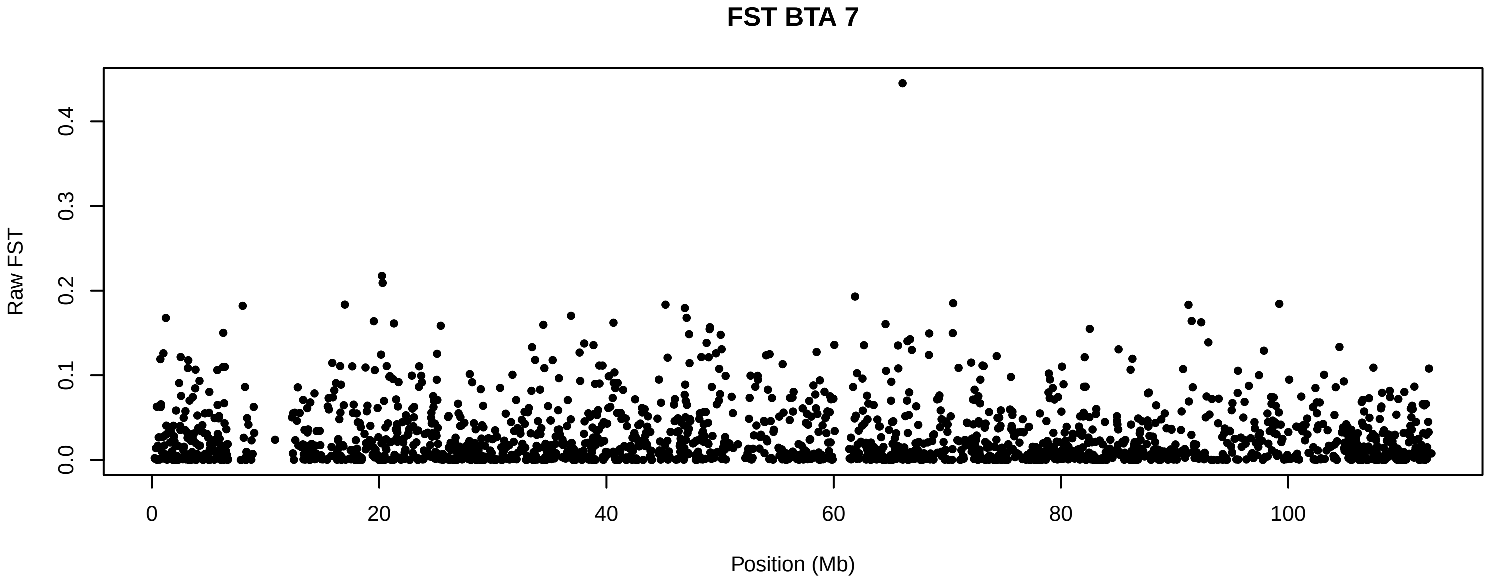
FST BTA 7
Raw FST
0.0
0.1
0.2
0.3
0.4
0
20
40
60
80
100
Position (Mb)

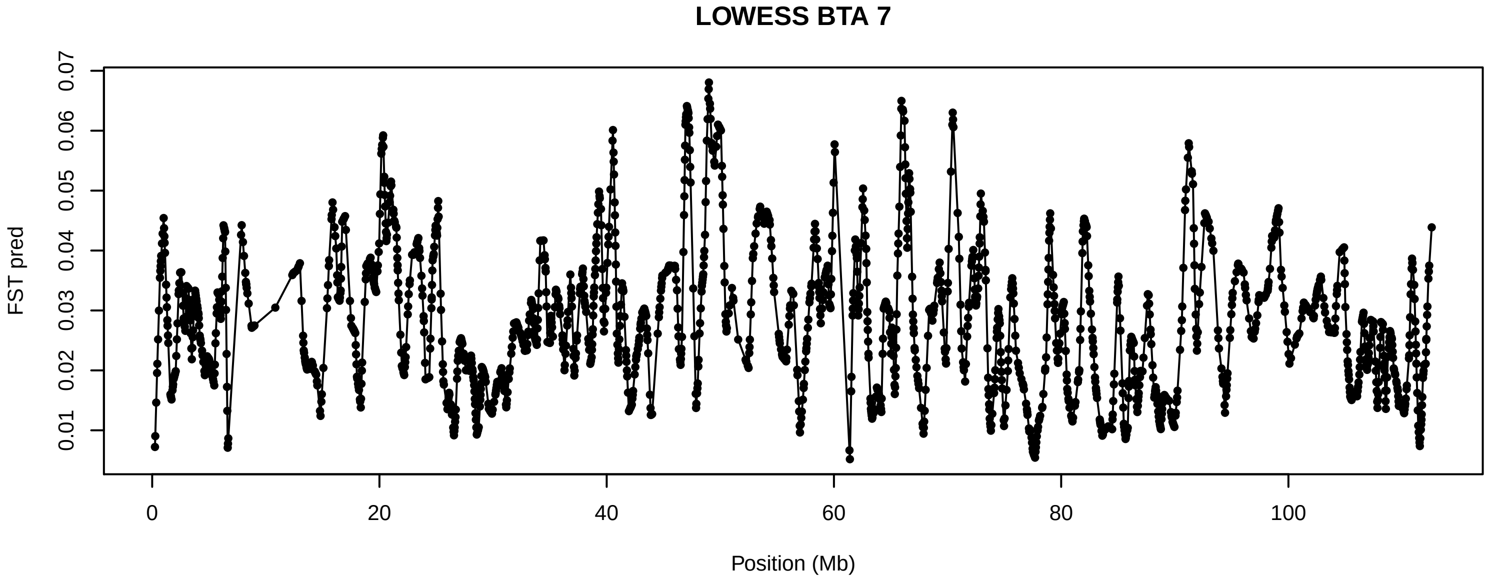
LOWESS BTA 7
FST pred
0.01
0.02
0.03
0.04
0.05
0.06
0.07
0
20
40
60
80
100
Position (Mb)

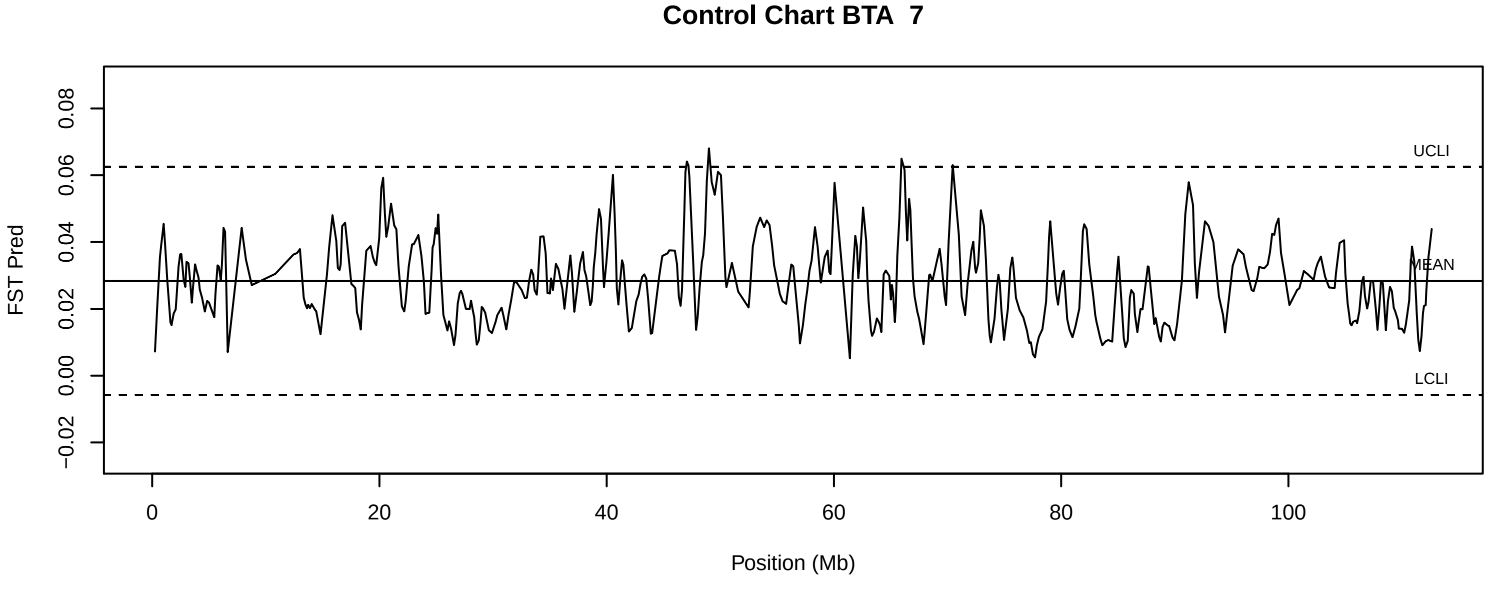
Control Chart BTA 7
FST Pred
−0.02
0.00
0.02
0.04
0.06
0.08
UCLI
MEAN
LCLI
0
20
40
60
80
100
Position (Mb)

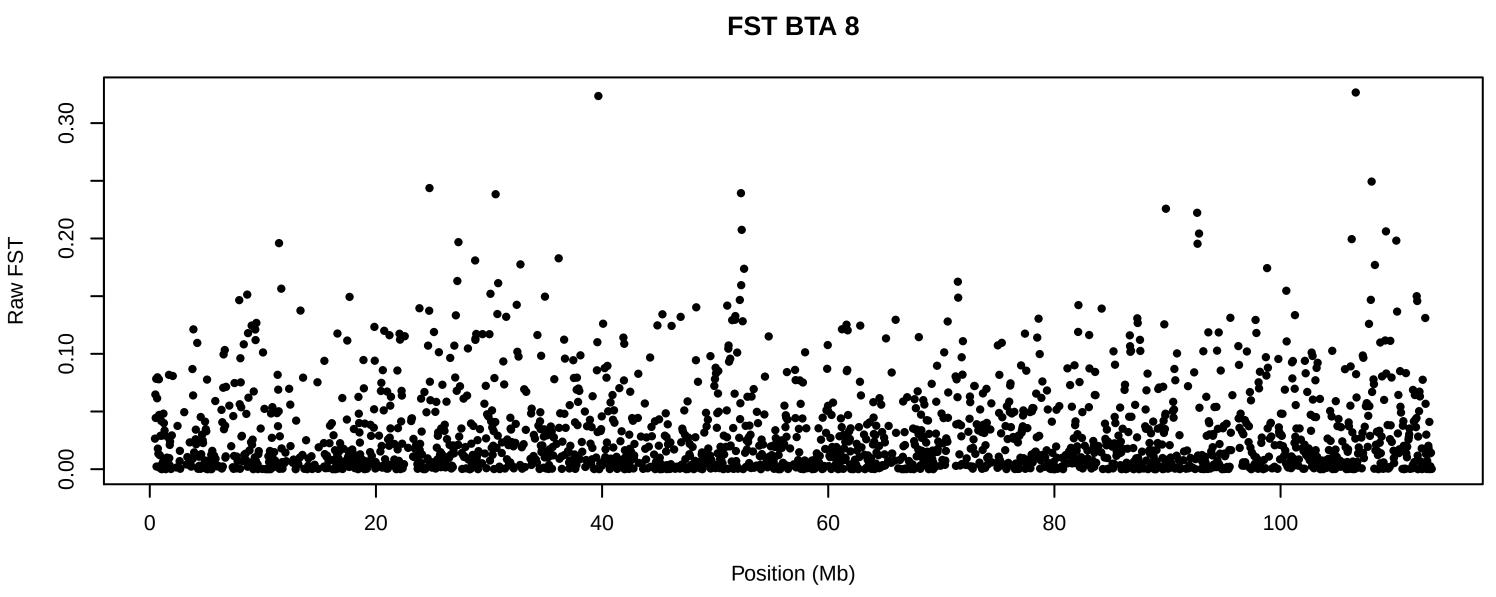
FST BTA 8
Raw FST
0.00
0.10
0.20
0.30
0
20
40
60
80
100
Position (Mb)

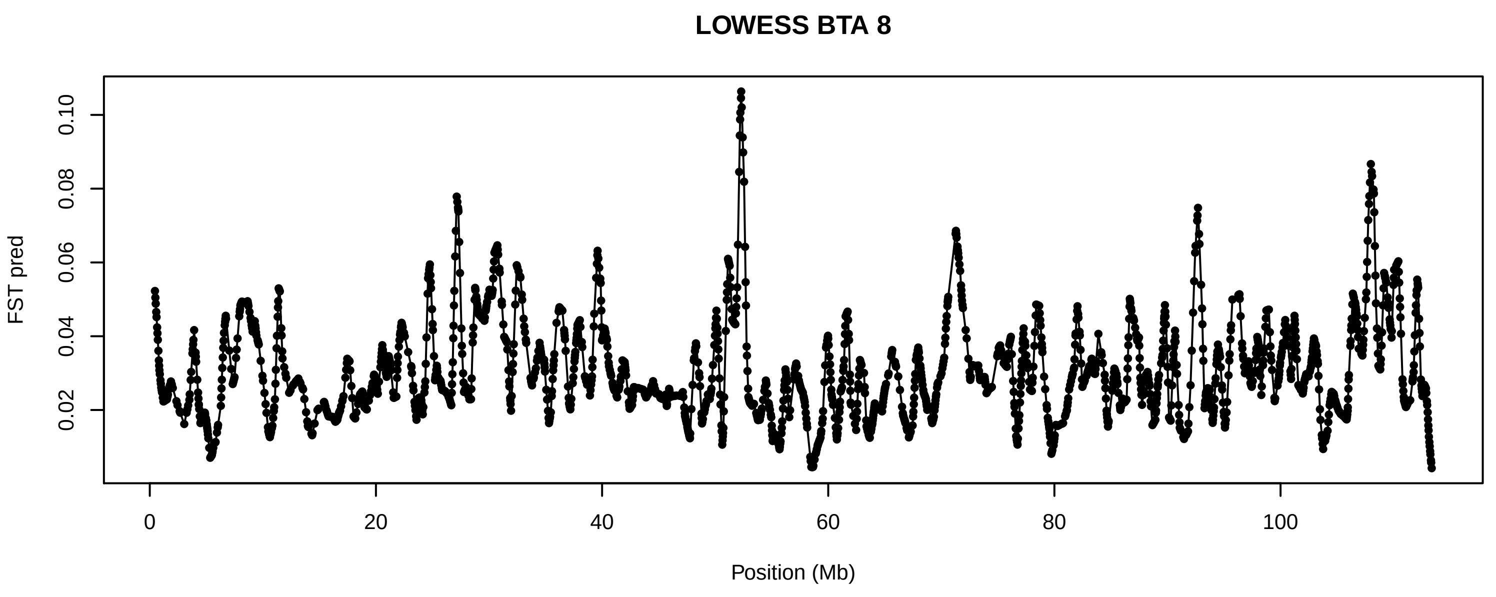
LOWESS BTA 8
FST pred
0.02
0.04
0.06
0.08
0.10
0
20
40
60
80
100
Position (Mb)

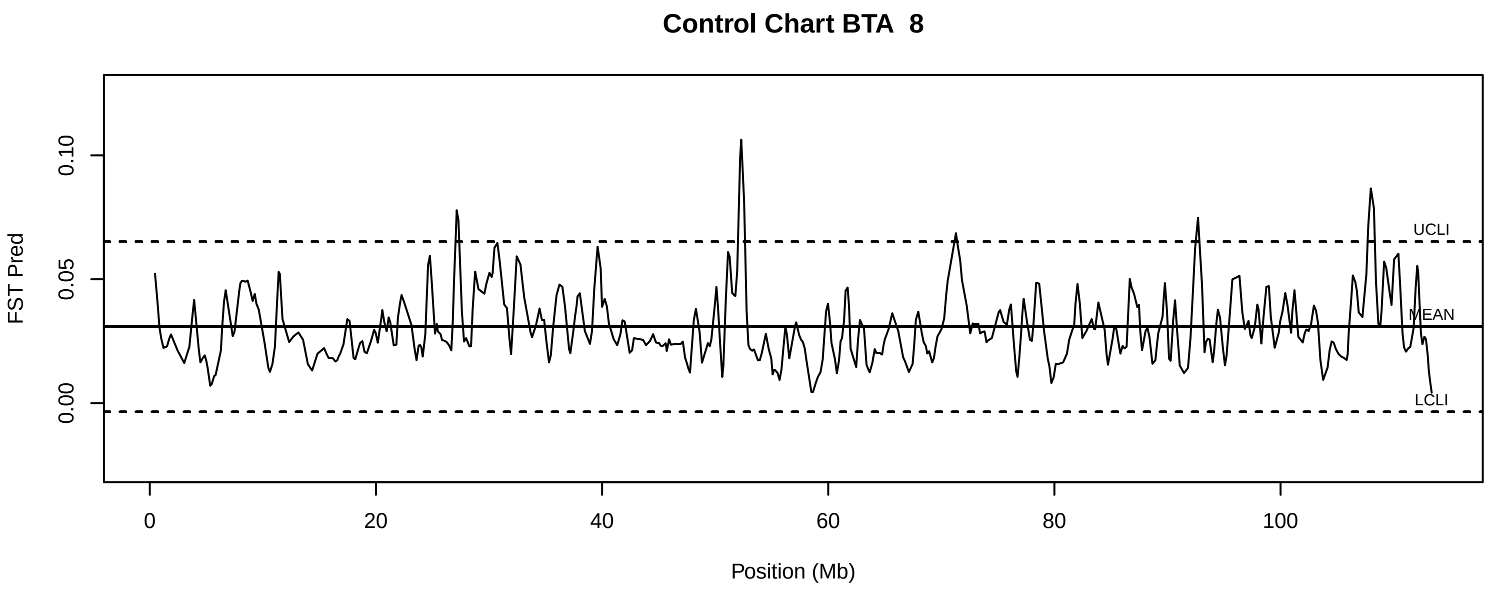
Control Chart BTA 8
FST Pred
0.00
0.05
0.10
UCLI
MEAN
LCLI
0
20
40
60
80
100
Position (Mb)

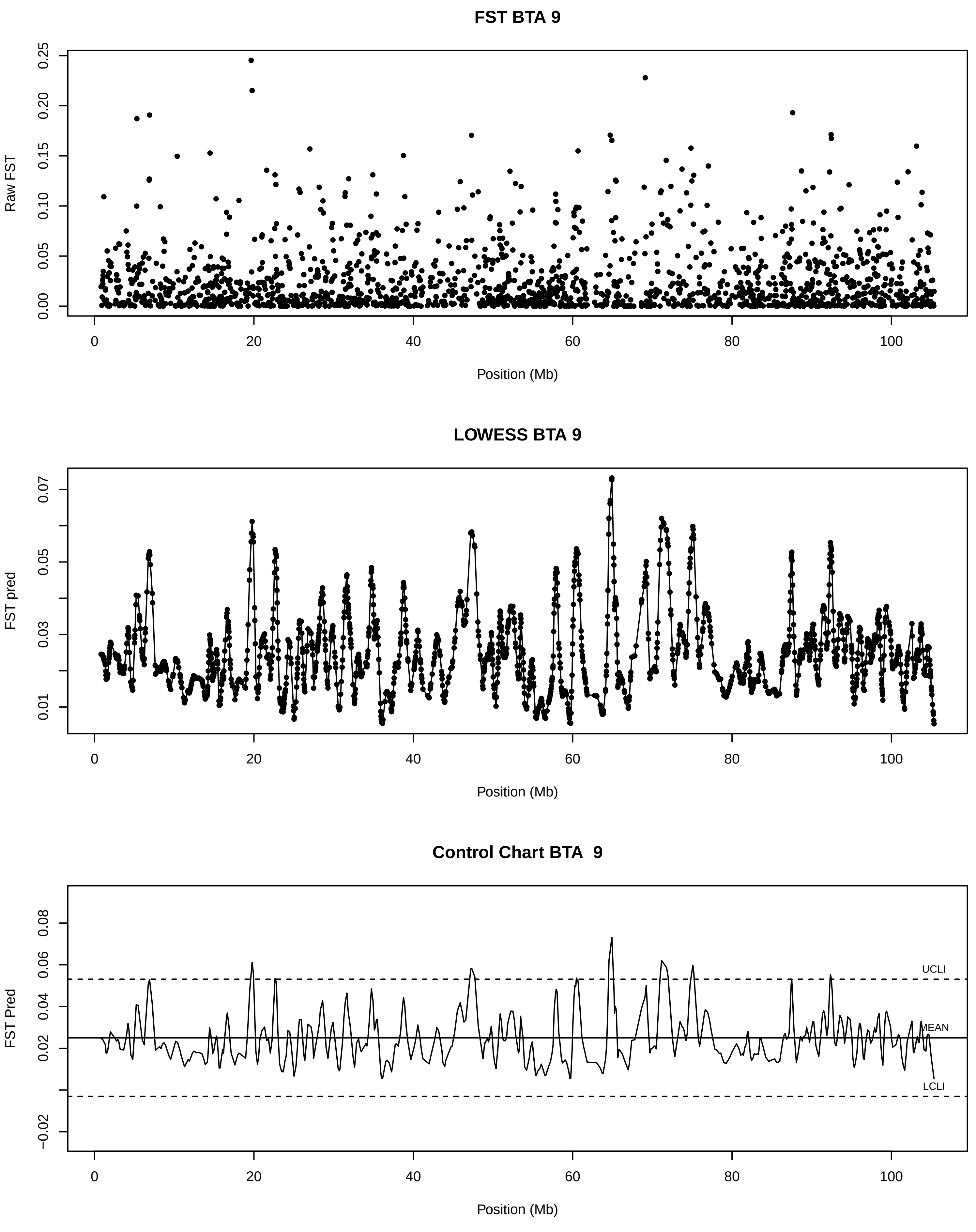
FST BTA 9
Raw FST
Position (Mb)
LOWESS BTA 9
FST pred
Position (Mb)
Control Chart BTA 9
FST Pred
UCLI
MEAN
LCLI
Position (Mb)

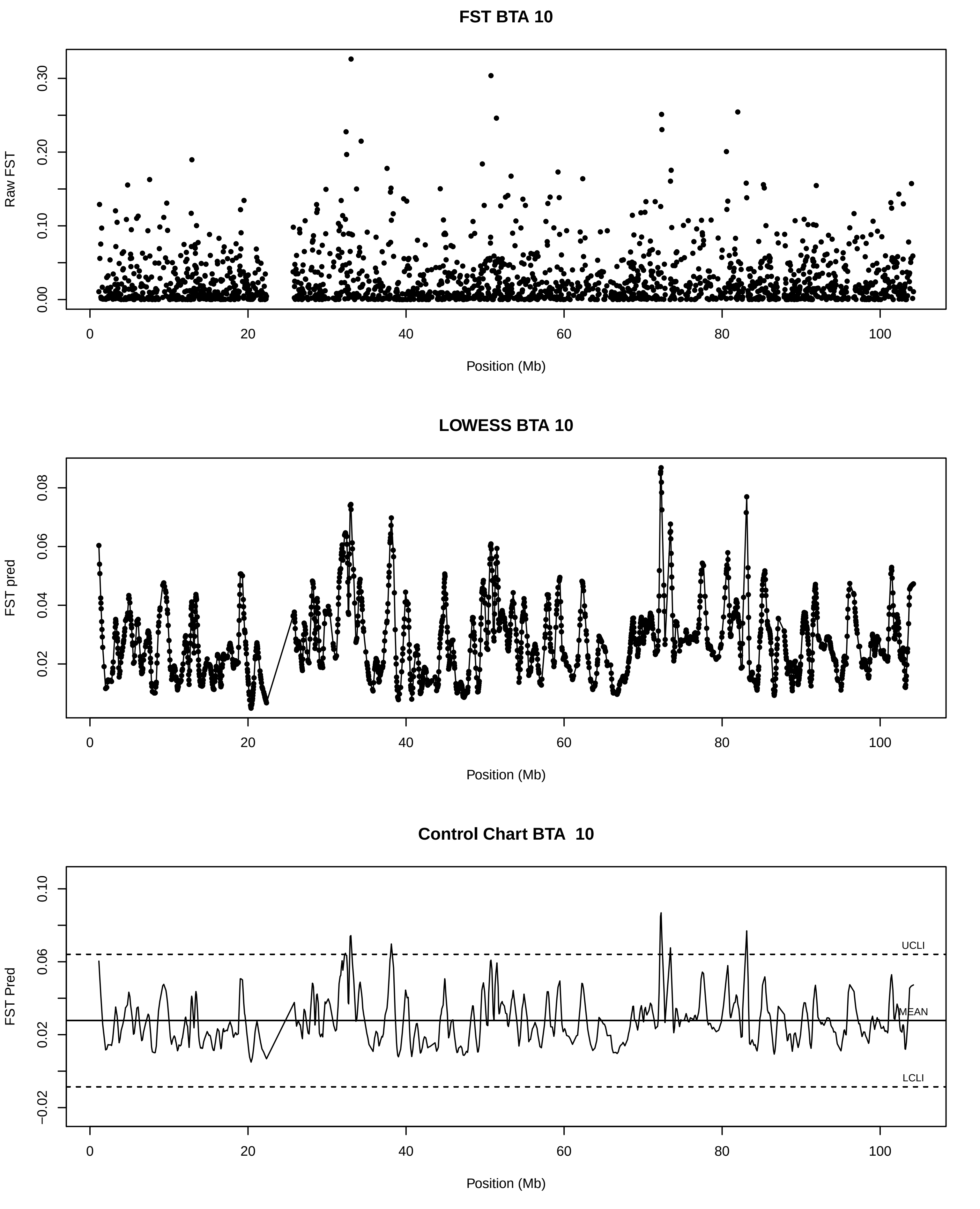
FST BTA 10
Raw FST
0.00
0.10
0.20
0.30
Position (Mb)
0
20
40
60
80
100
LOWESS BTA 10
FST pred
0.02
0.04
0.06
0.08
Position (Mb)
Control Chart BTA 10
FST Pred
−0.02
0.02
0.06
0.10
UCLI
MEAN
LCLI
Position (Mb)

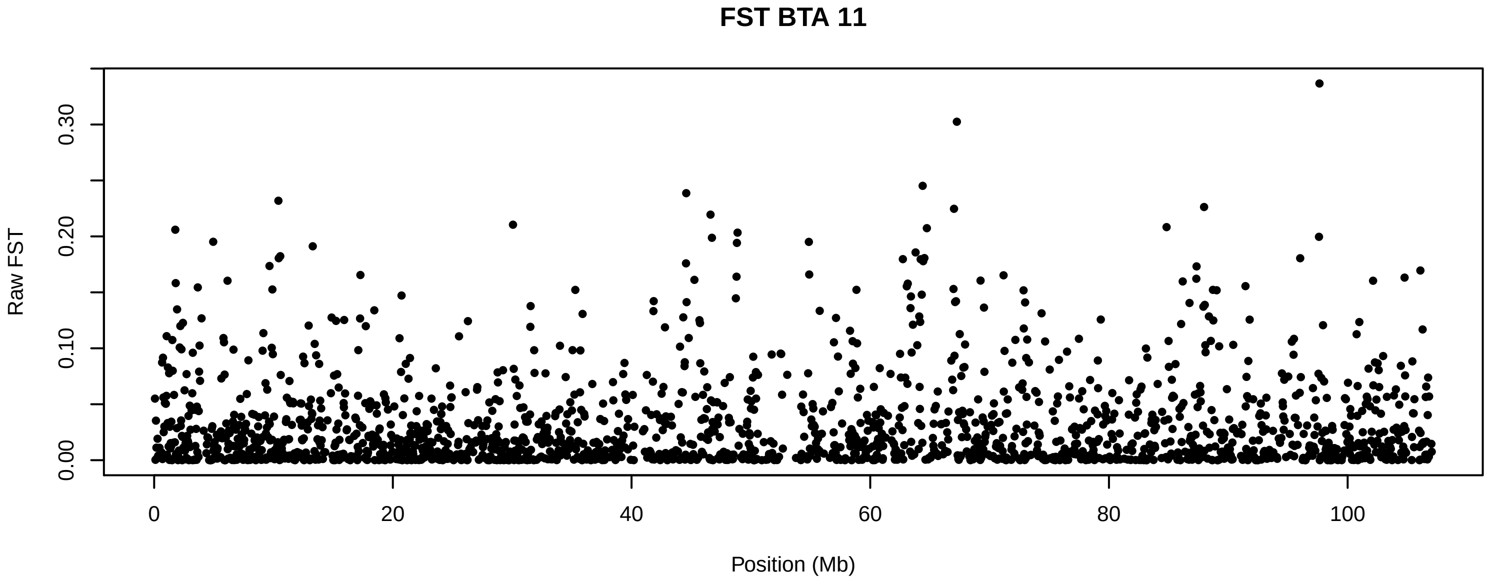
FST BTA 11
Raw FST
0.00
0.10
0.20
0.30
0
20
40
60
80
100
Position (Mb)

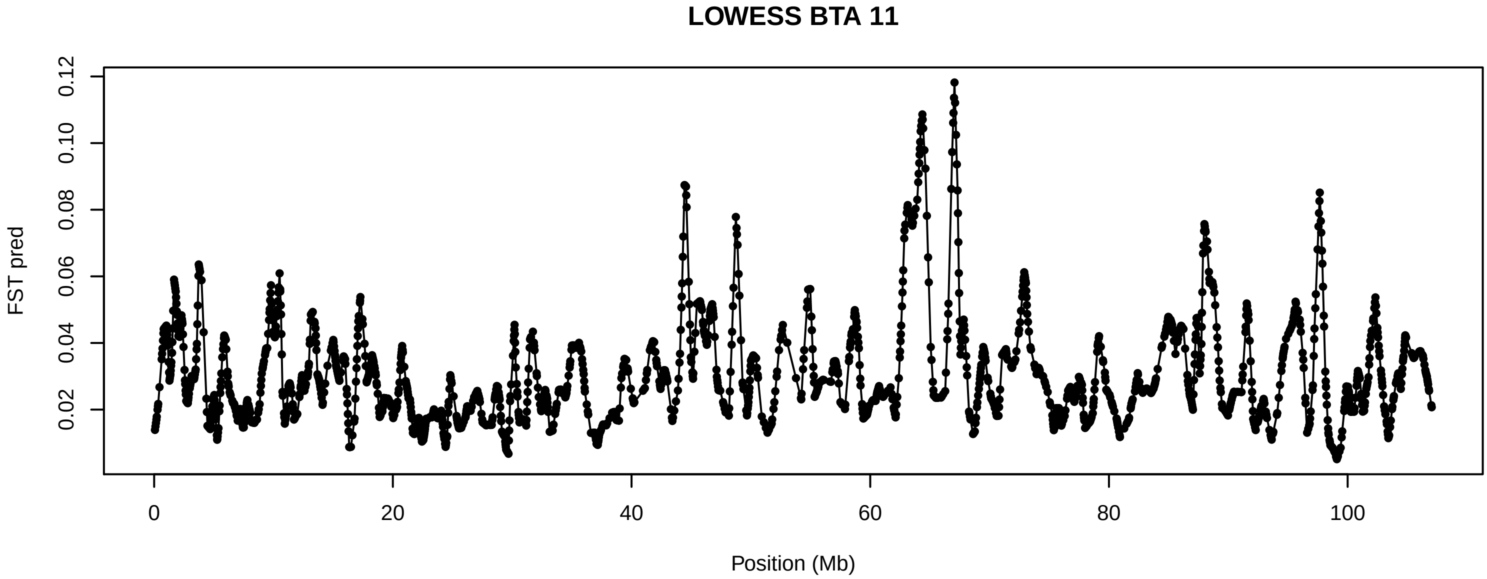
LOWESS BTA 11
FST pred
0.02
0.04
0.06
0.08
0.10
0.12
0
20
40
60
80
100
Position (Mb)

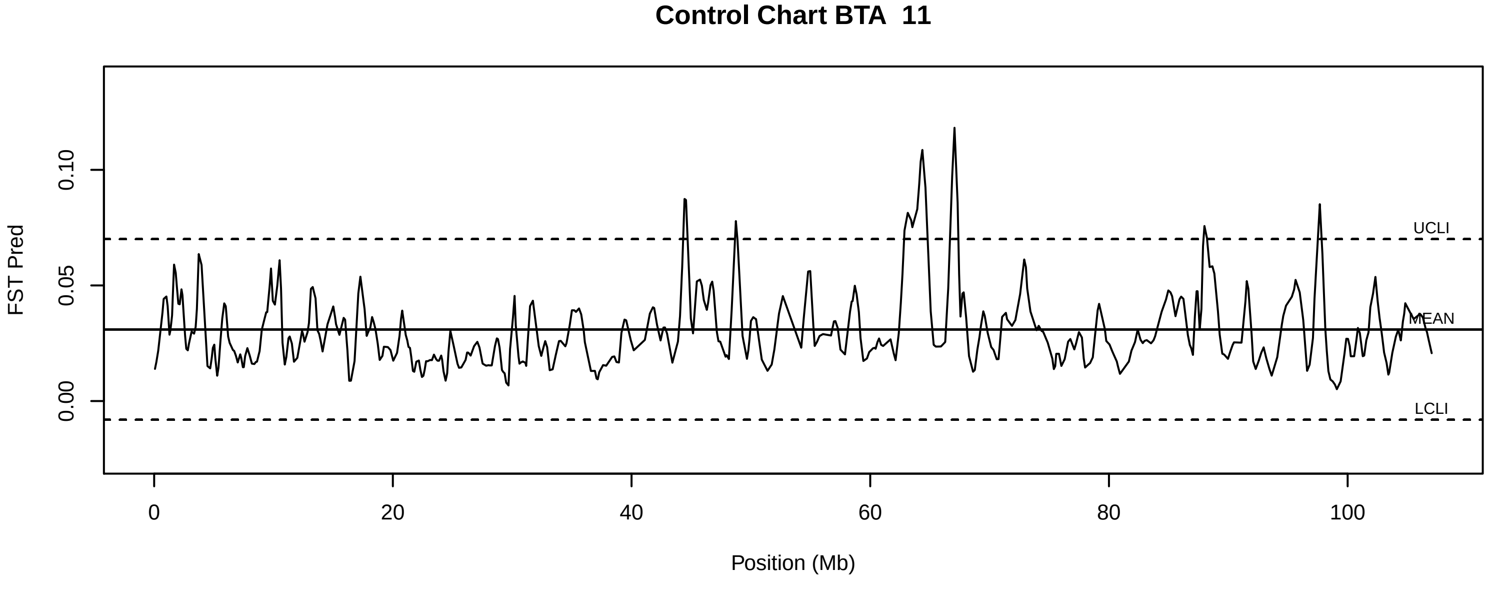
Control Chart BTA 11
FST Pred
0.00
0.05
0.10
UCLI
MEAN
LCLI
0
20
40
60
80
100
Position (Mb)

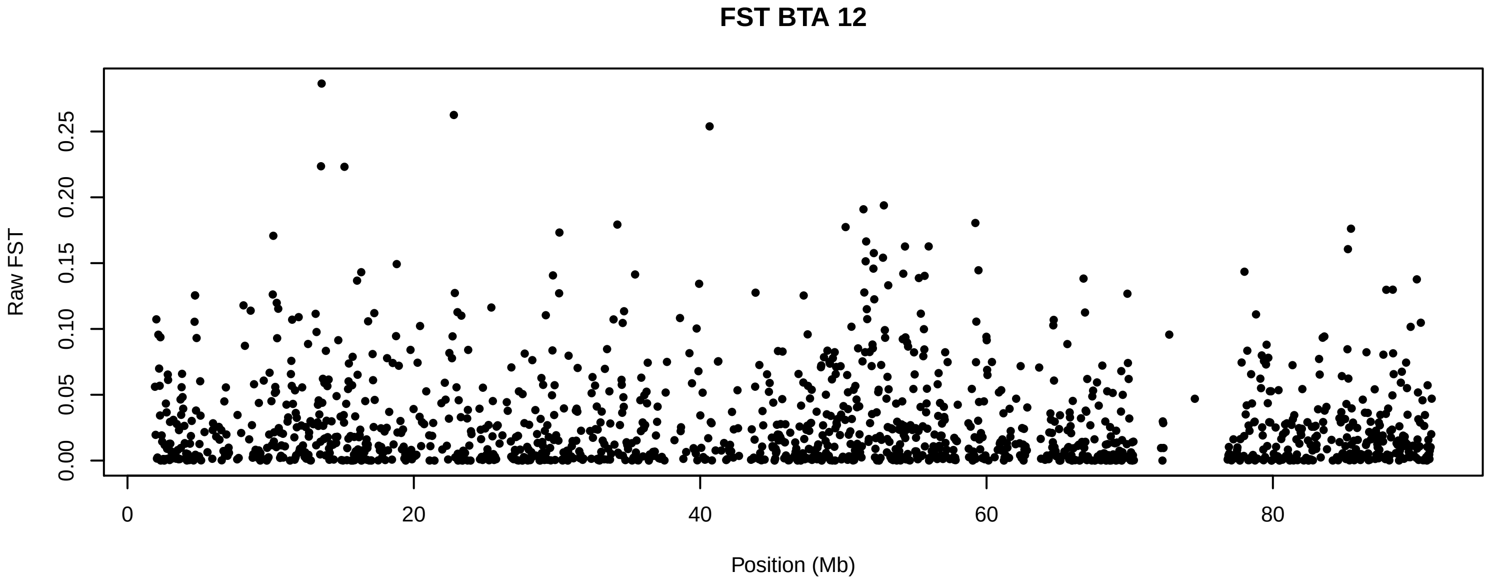

FST BTA 12
Raw FST
0.00
0.05
0.10
0.15
0.20
0.25
0
20
40
60
80
Position (Mb)

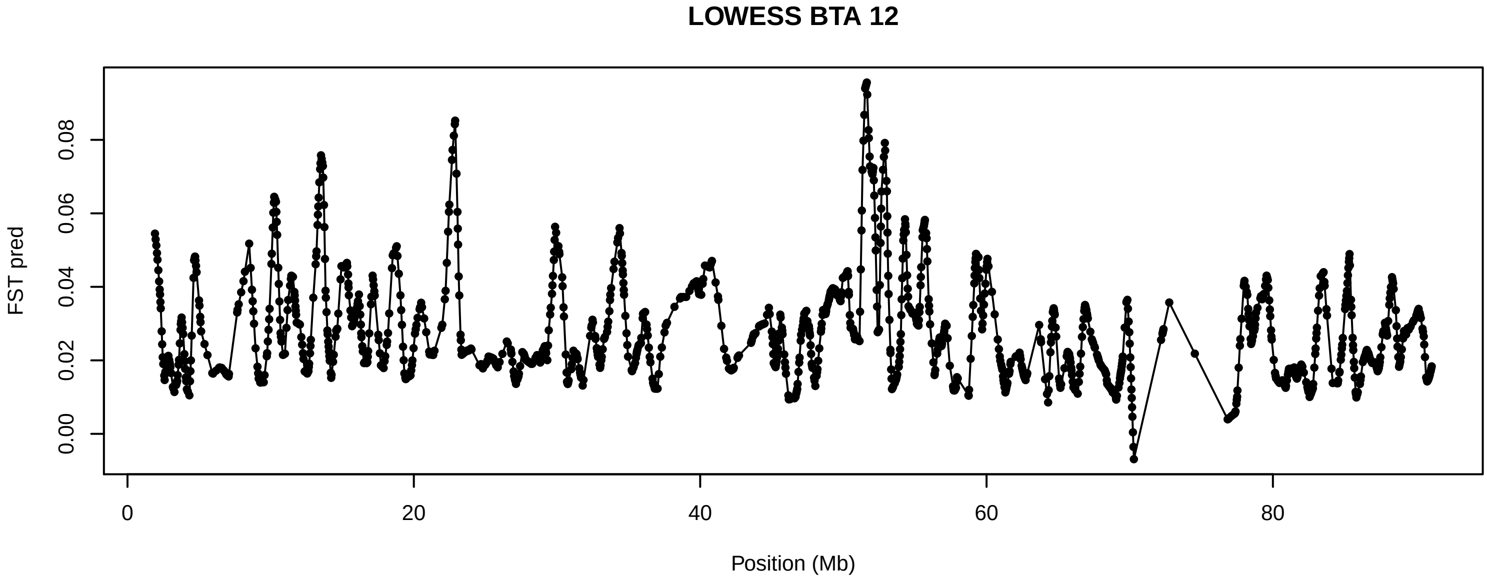

LOWESS BTA 12
FST pred
0.00
0.02
0.04
0.06
0.08
0
20
40
60
80
Position (Mb)

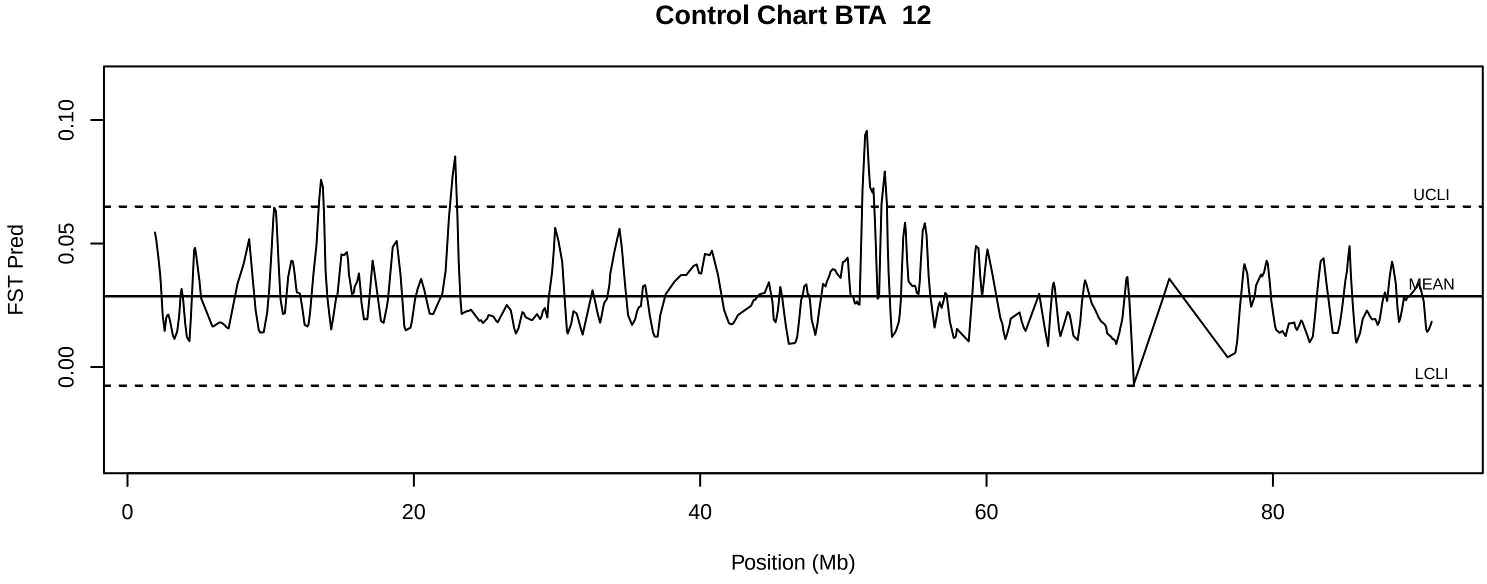

Control Chart BTA 12
FST Pred
0.00
0.05
0.10
UCLI
MEAN
LCLI
0
20
40
60
80
Position (Mb)

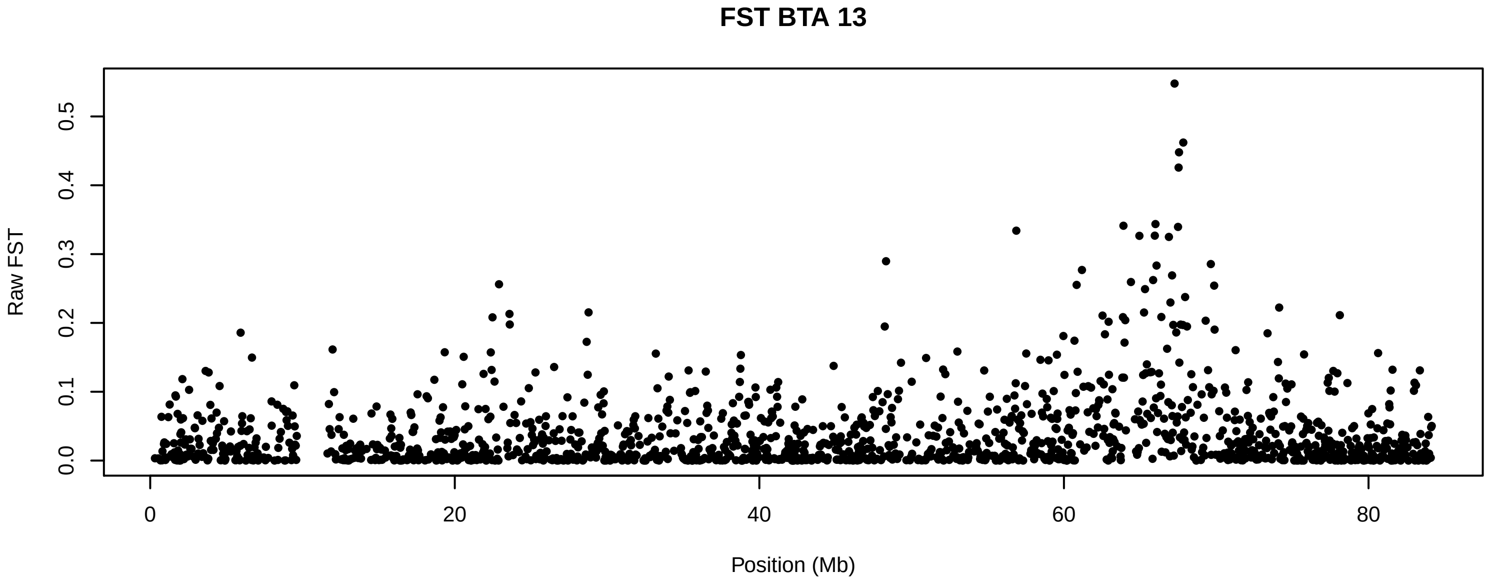
FST BTA 13
Raw FST
0.0
0.1
0.2
0.3
0.4
0.5
0
20
40
60
80
Position (Mb)

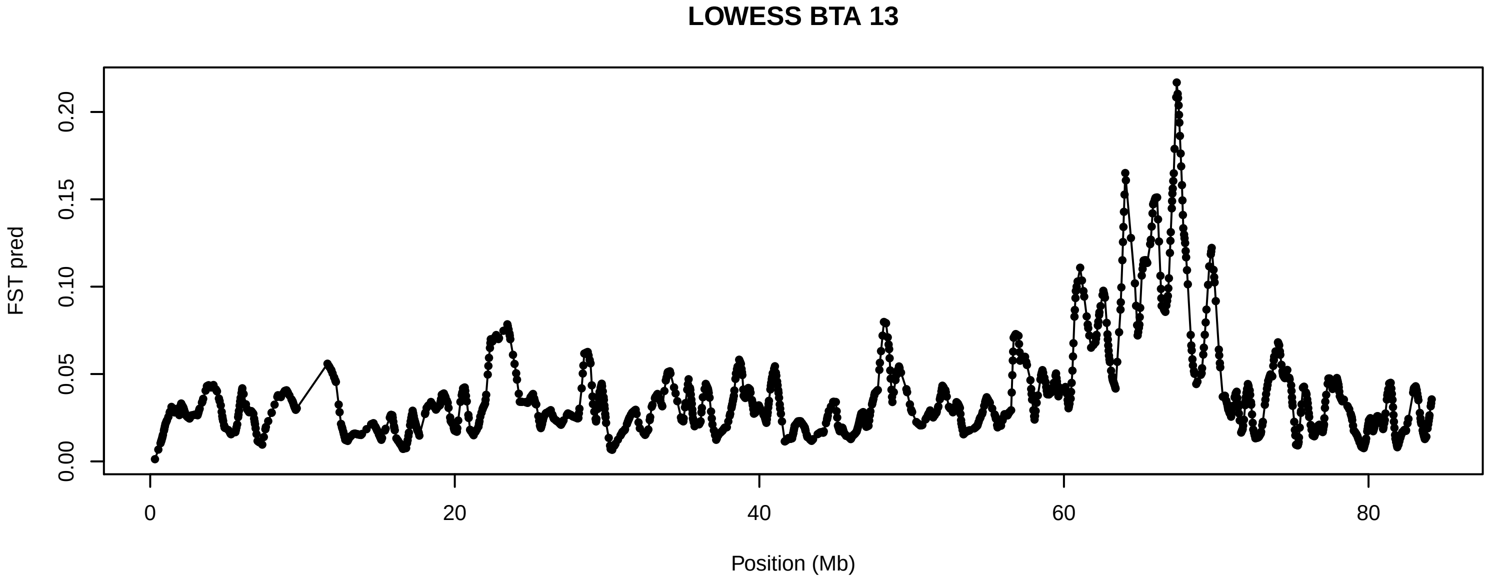
LOWESS BTA 13
FST pred
0.00
0.05
0.10
0.15
0.20
0
20
40
60
80
Position (Mb)

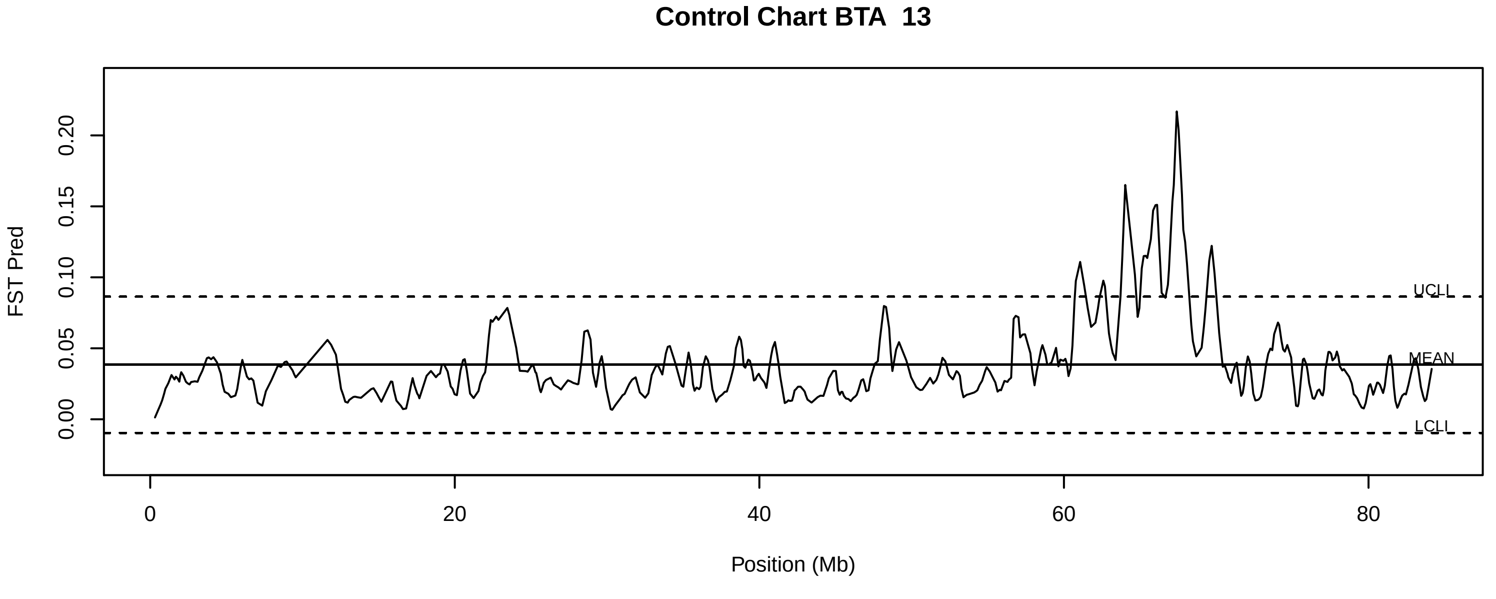
Control Chart BTA 13
FST Pred
0.00
0.05
0.10
0.15
0.20
UCLL
MEAN
LCLL
0
20
40
60
80
Position (Mb)

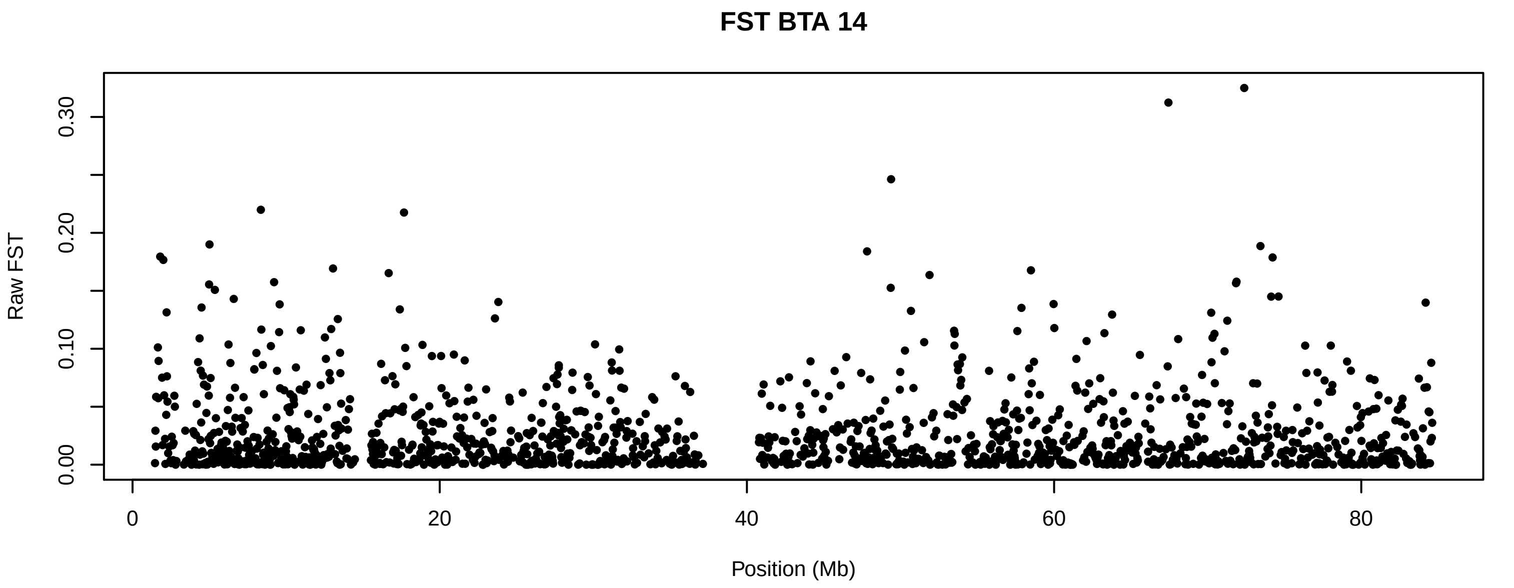
FST BTA 14
Raw FST
0.00
0.10
0.20
0.30
0
20
40
60
80
Position (Mb)

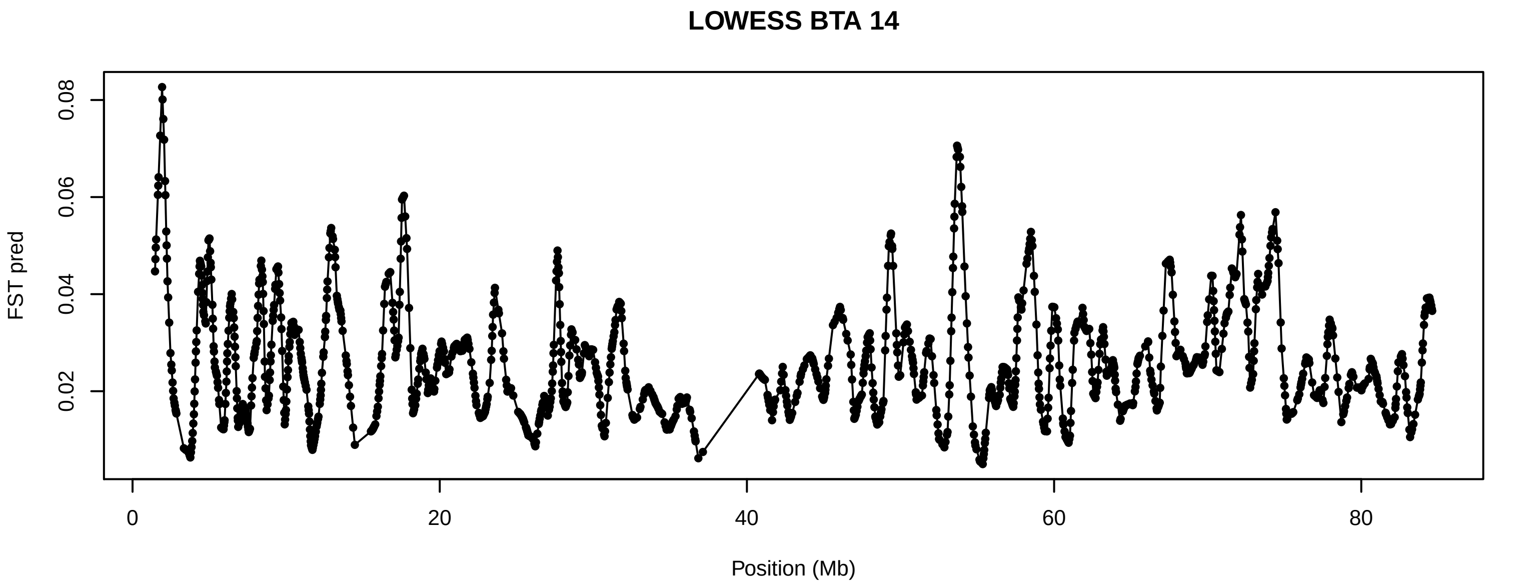
LOWESS BTA 14
FST pred
0.02
0.04
0.06
0.08
0
20
40
60
80
Position (Mb)

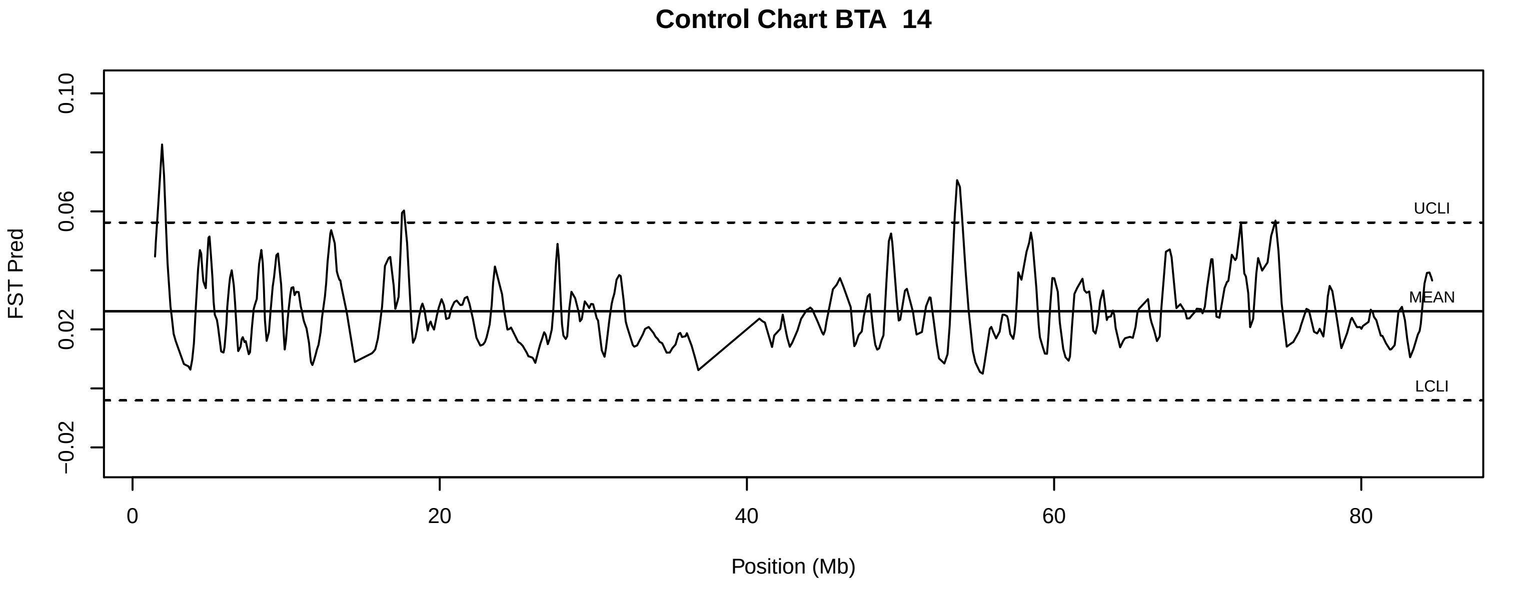
Control Chart BTA 14
FST Pred
−0.02
0.02
0.06
0.10
UCLI
MEAN
LCLI
0
20
40
60
80
Position (Mb)

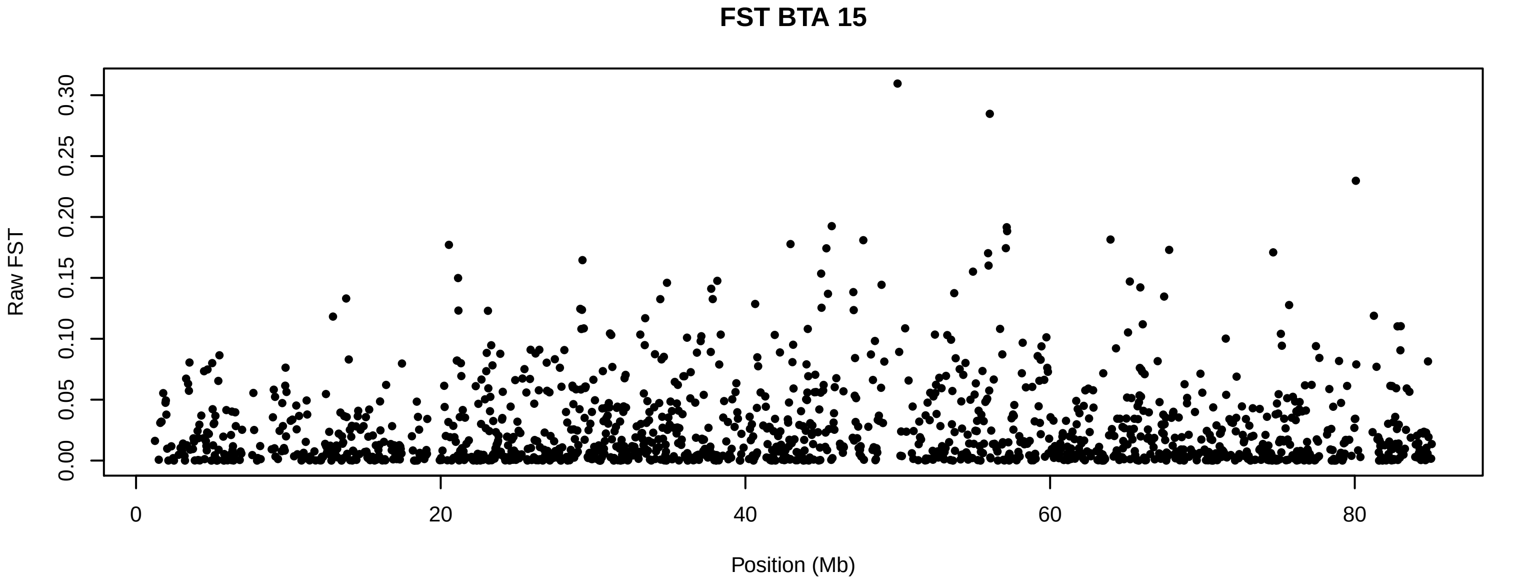
FST BTA 15
Raw FST
0.00
0.05
0.10
0.15
0.20
0.25
0.30
0
20
40
60
80
Position (Mb)

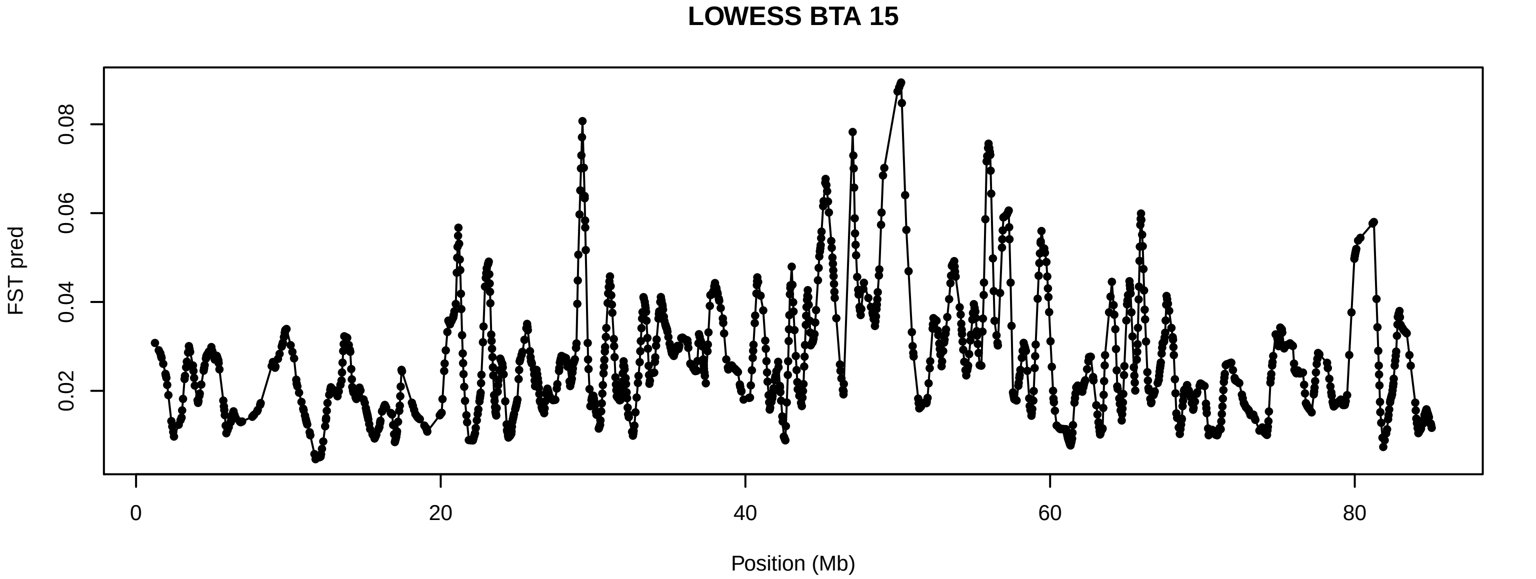
LOWESS BTA 15
FST pred
0.02
0.04
0.06
0.08
0
20
40
60
80
Position (Mb)

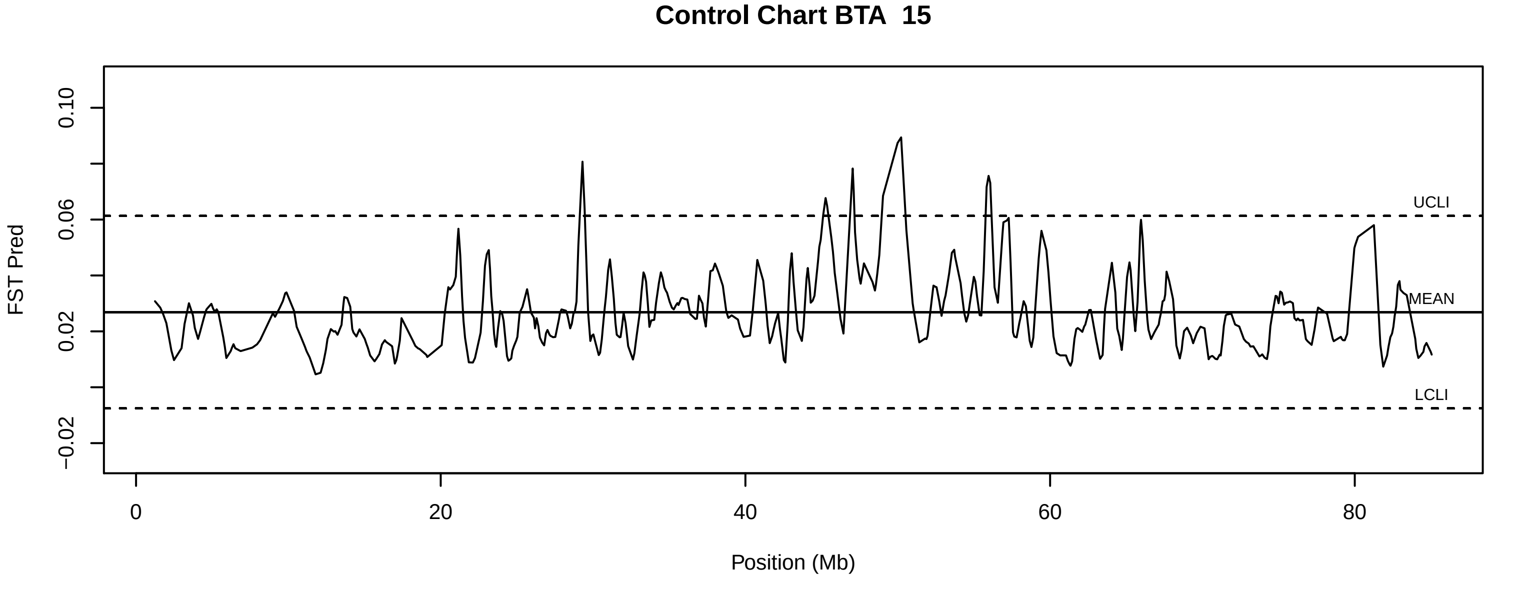
Control Chart BTA 15
FST Pred
−0.02
0.02
0.06
0.10
UCLI
MEAN
LCLI
0
20
40
60
80
Position (Mb)

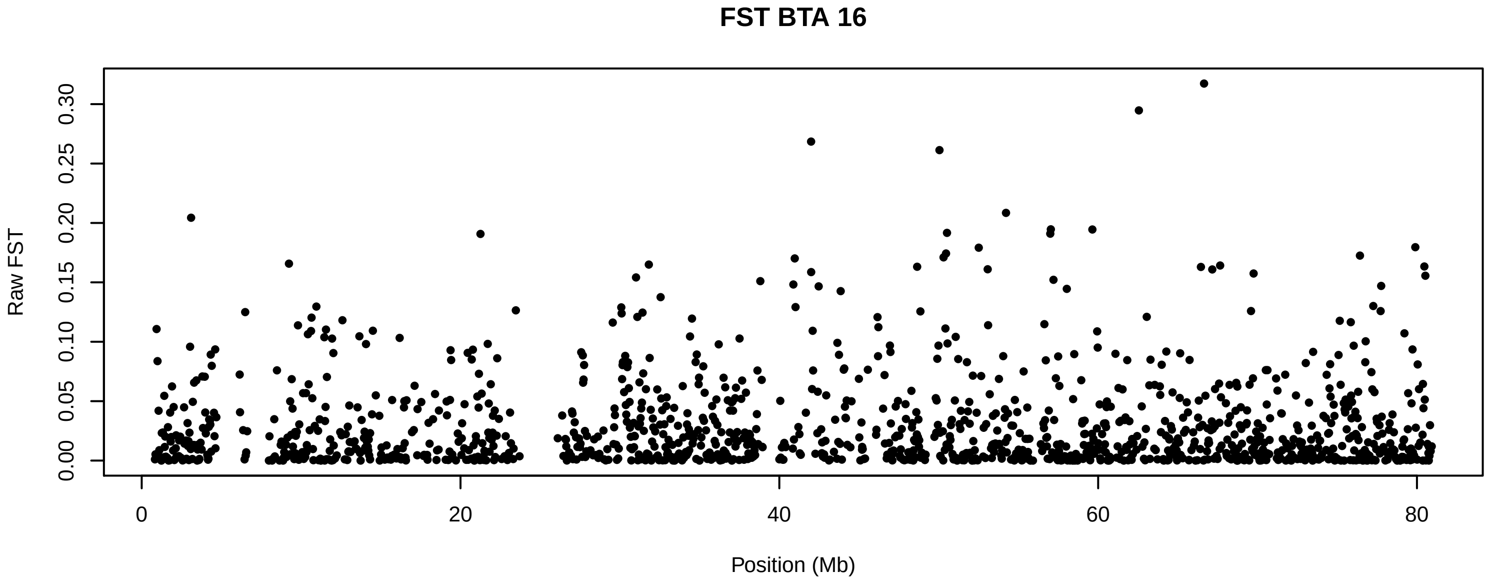

FST BTA 16
Raw FST
0.00
0.05
0.10
0.15
0.20
0.25
0.30
0
20
40
60
80
Position (Mb)

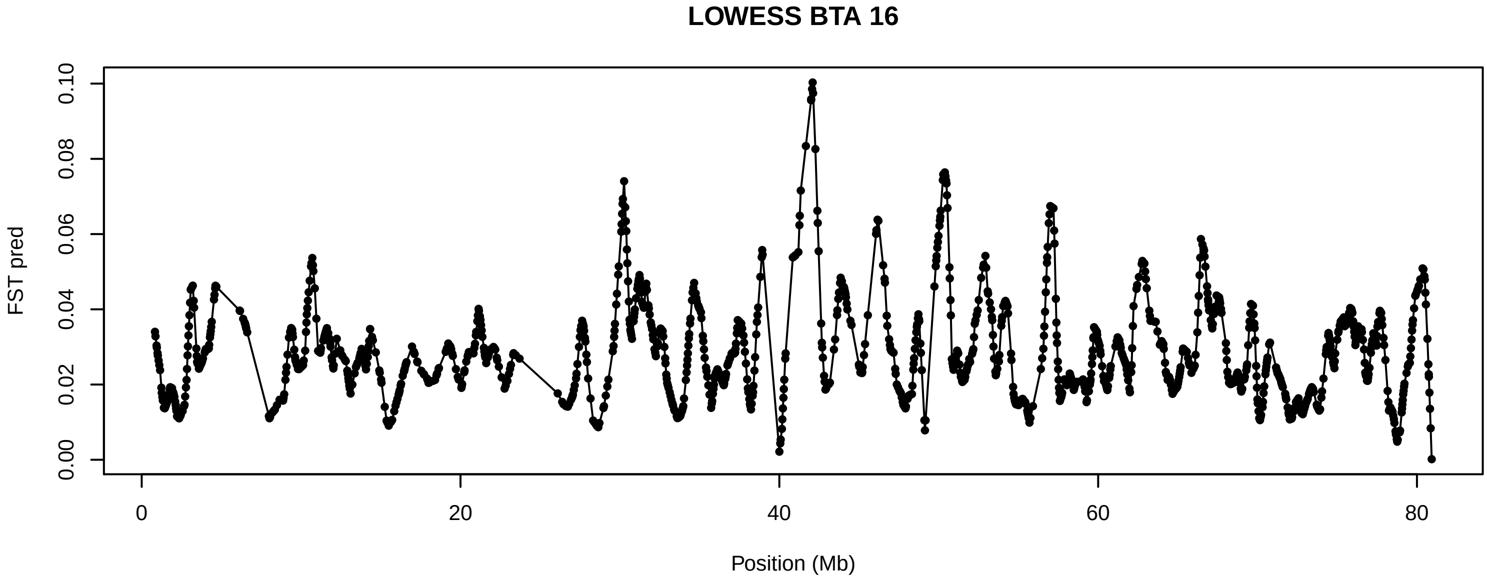

LOWESS BTA 16
FST pred
0.00
0.02
0.04
0.06
0.08
0.10
0
20
40
60
80
Position (Mb)

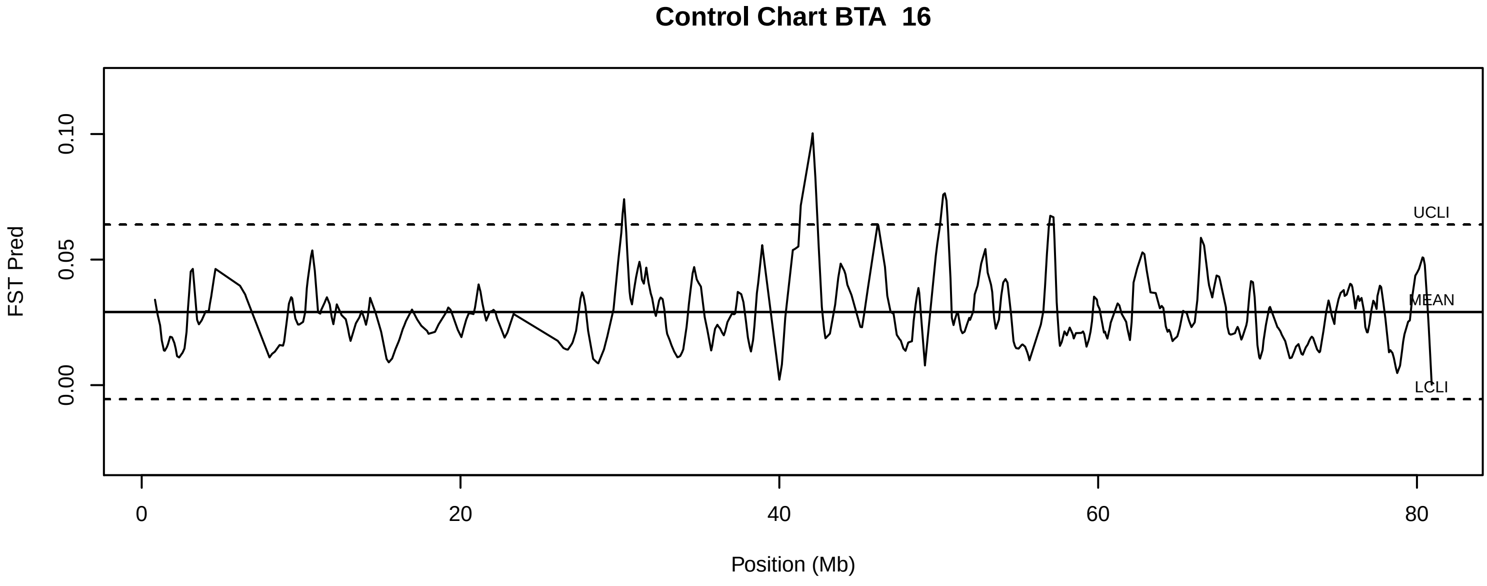

Control Chart BTA 16
FST Pred
0.00
0.05
0.10
UCLI
MEAN
LCLI
0
20
40
60
80
Position (Mb)

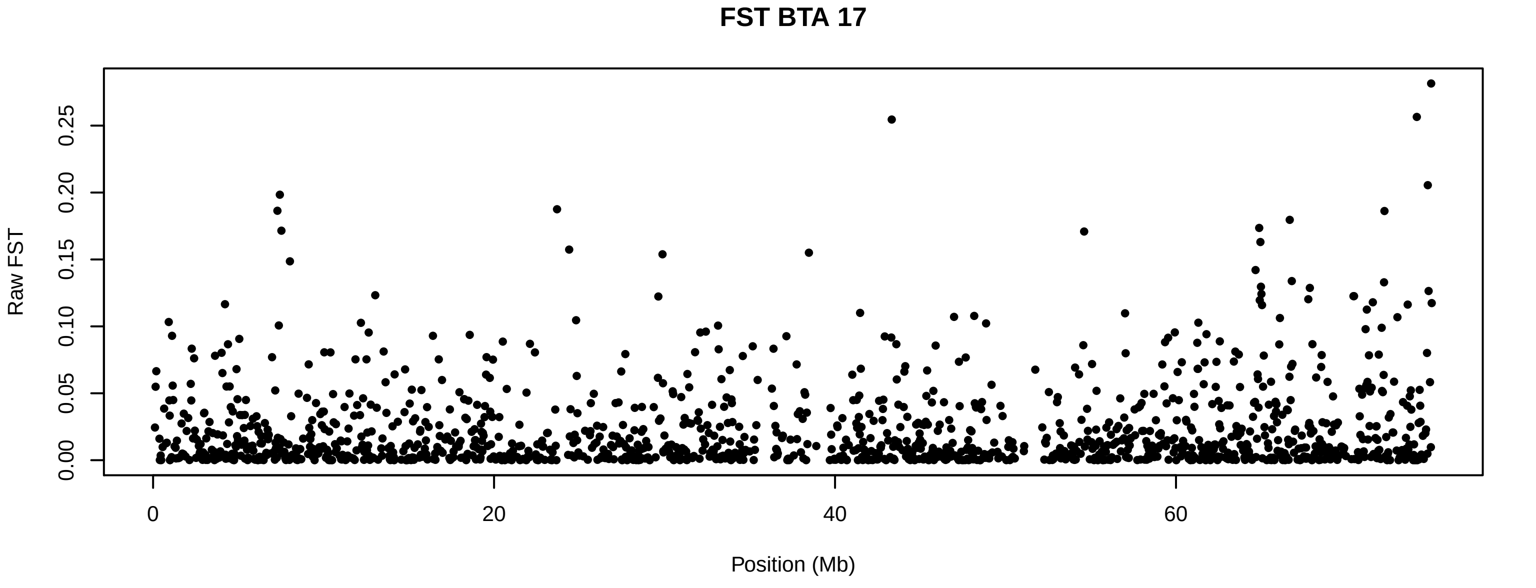
FST BTA 17
Raw FST
0.00
0.05
0.10
0.15
0.20
0.25
0
20
40
60
Position (Mb)

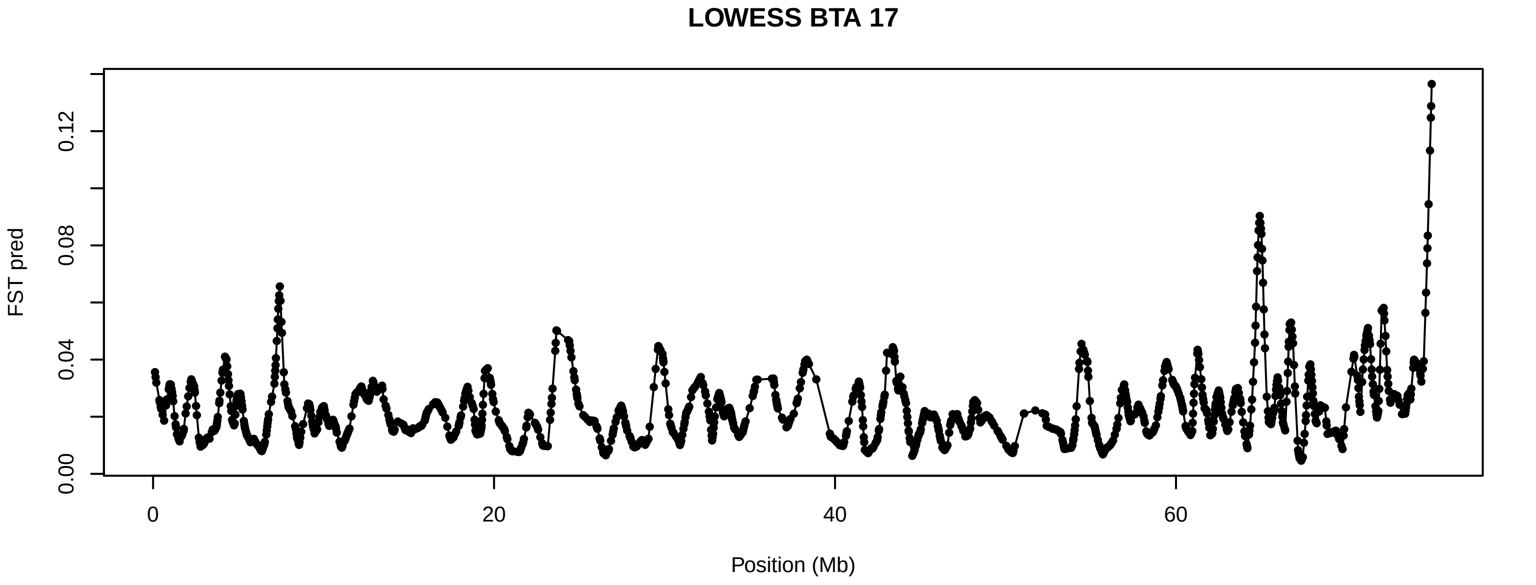
LOWESS BTA 17
FST pred
0.00
0.04
0.08
0.12
0
20
40
60
Position (Mb)

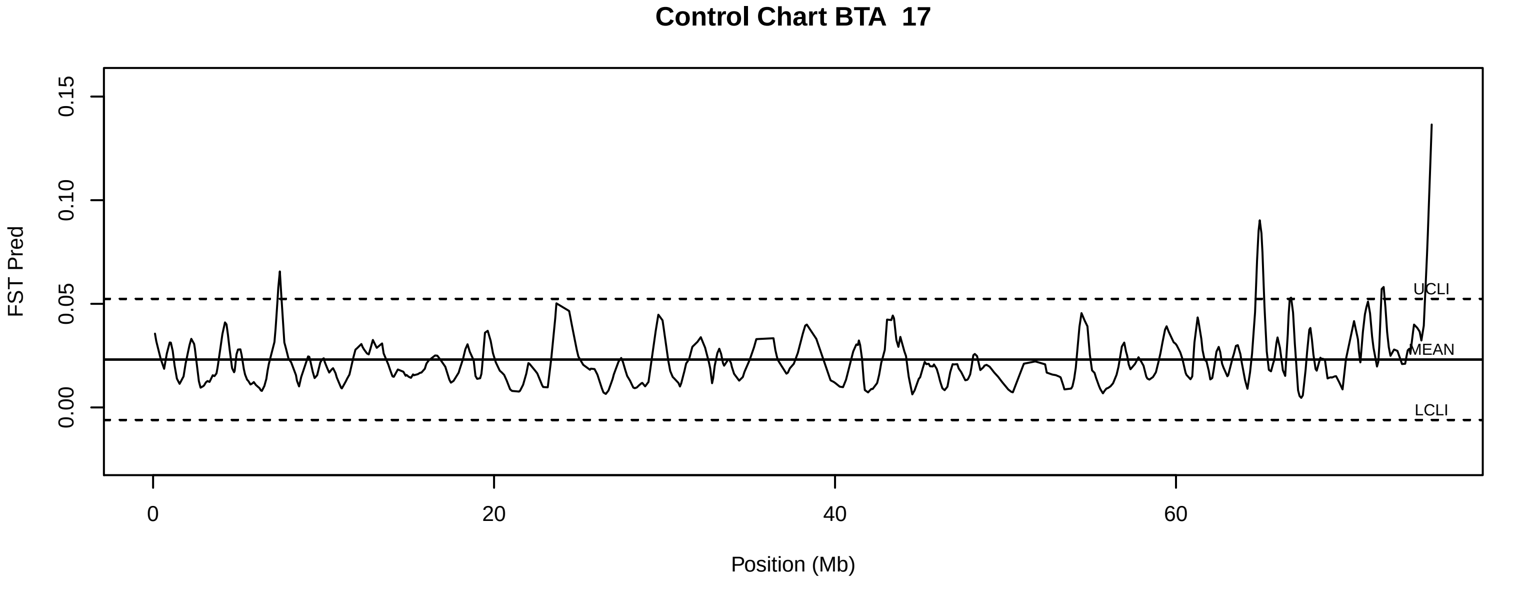
Control Chart BTA 17
FST Pred
0.00
0.05
0.10
0.15
UCLI
MEAN
LCLI
0
20
40
60
Position (Mb)

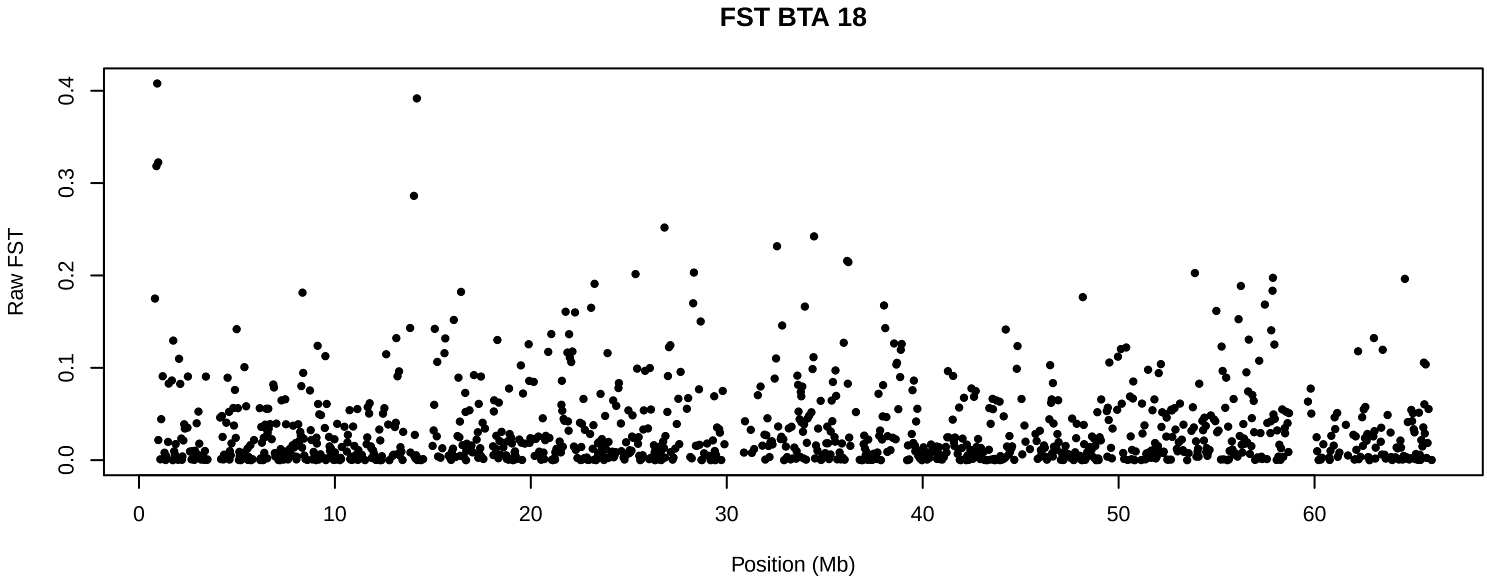
FST BTA 18
Raw FST
0.0
0.1
0.2
0.3
0.4
0
10
20
30
40
50
60
Position (Mb)

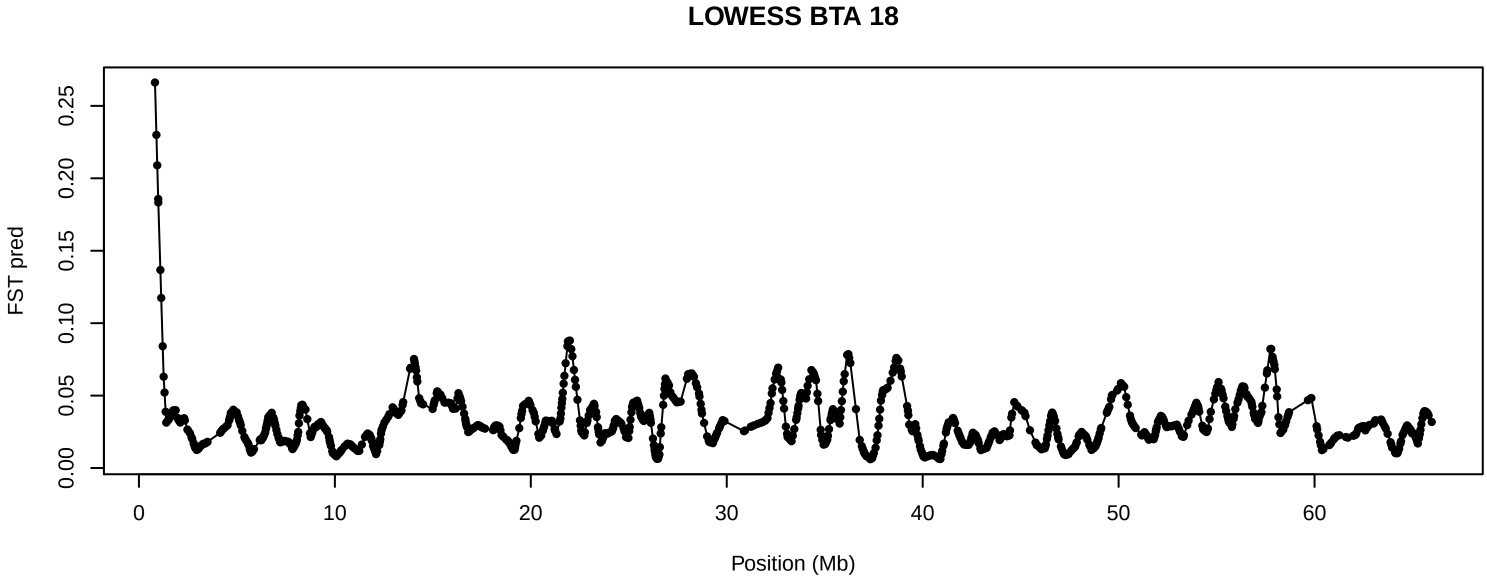
LOWESS BTA 18
FST pred
0.00
0.05
0.10
0.15
0.20
0.25
0
10
20
30
40
50
60
Position (Mb)

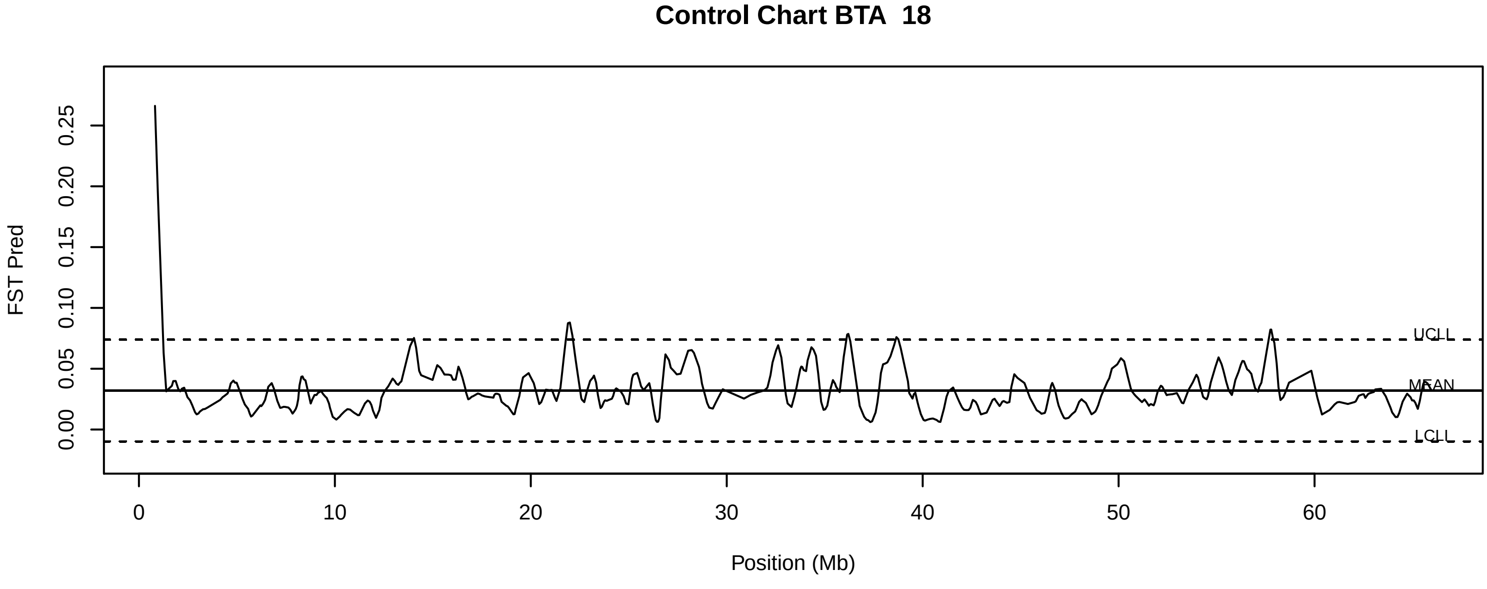
Control Chart BTA 18
FST Pred
0.00
0.05
0.10
0.15
0.20
0.25
UCLL
MEAN
LCLL
0
10
20
30
40
50
60
Position (Mb)

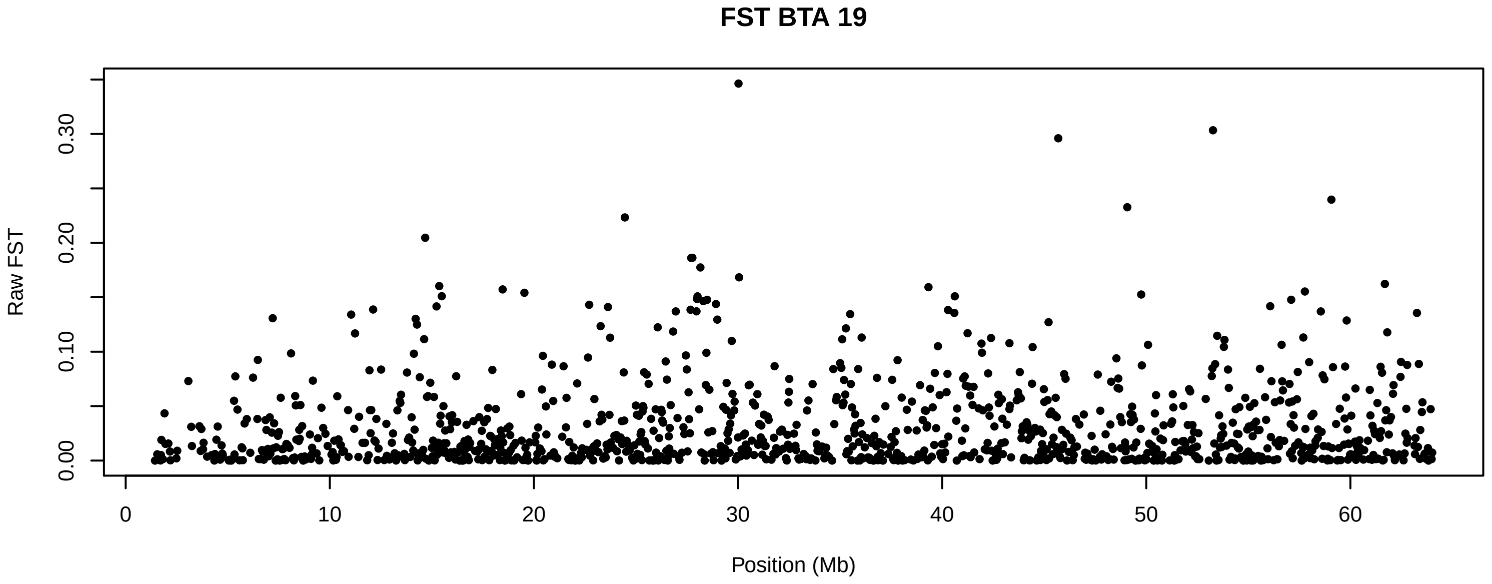
FST BTA 19
Raw FST
0.00
0.10
0.20
0.30
0
10
20
30
40
50
60
Position (Mb)

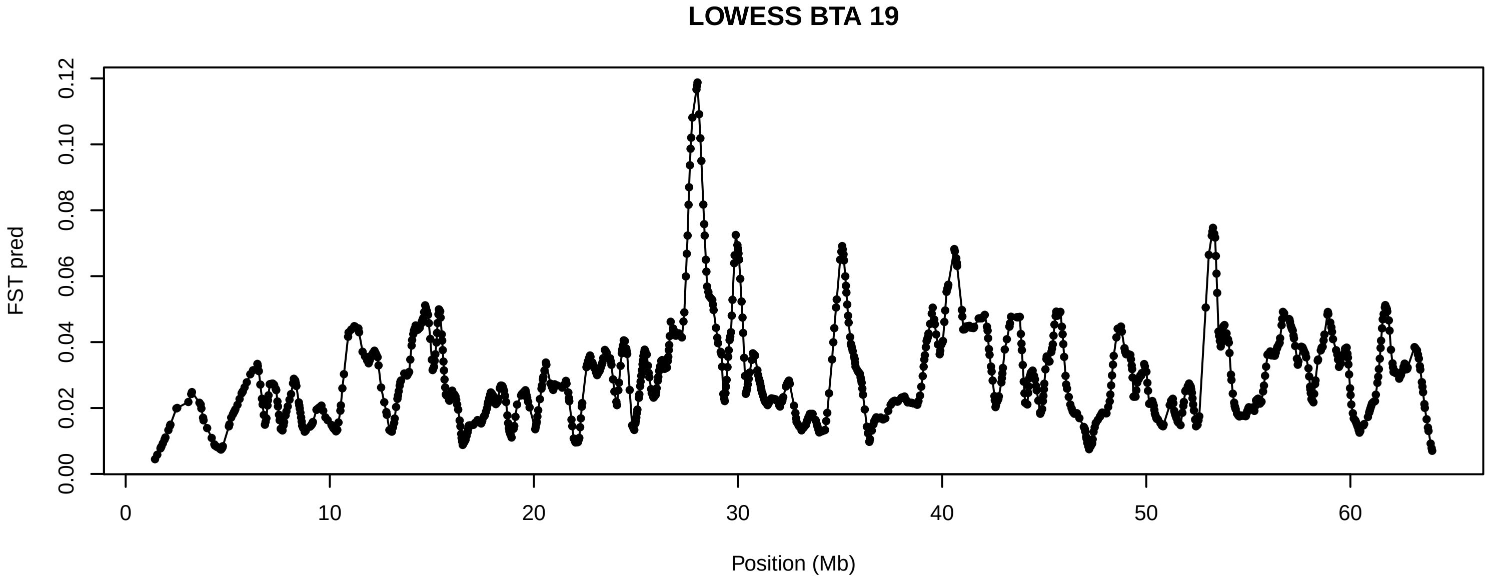
LOWESS BTA 19
FST pred
0.00
0.02
0.04
0.06
0.08
0.10
0.12
0
10
20
30
40
50
60
Position (Mb)

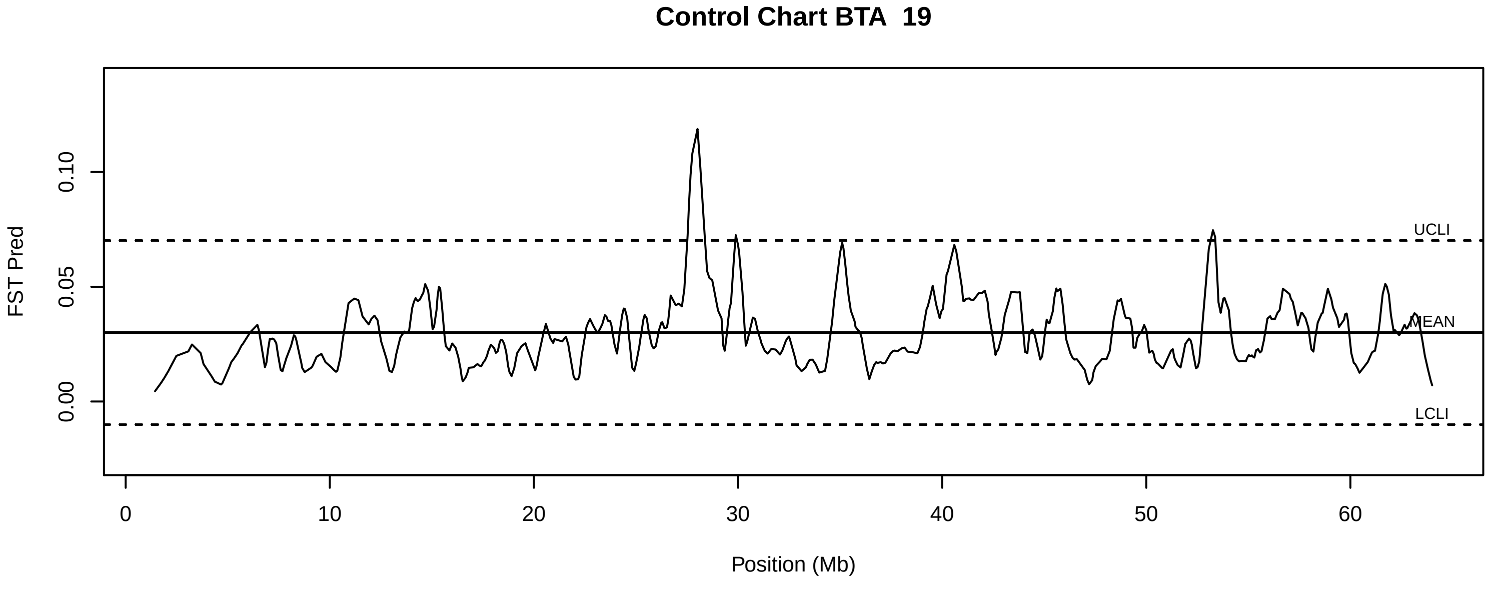
Control Chart BTA 19
FST Pred
0.00
0.05
0.10
UCLI
MEAN
LCLI
0
10
20
30
40
50
60
Position (Mb)

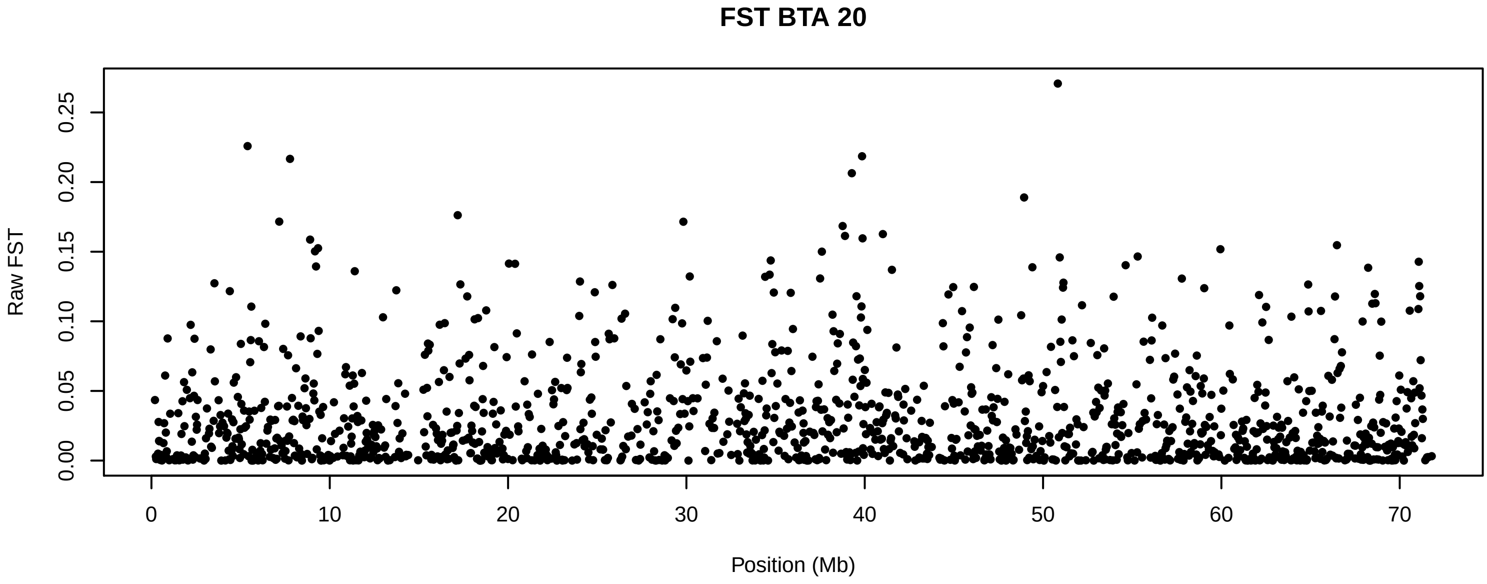
FST BTA 20
Raw FST
0.00
0.05
0.10
0.15
0.20
0.25
0
10
20
30
40
50
60
70
Position (Mb)

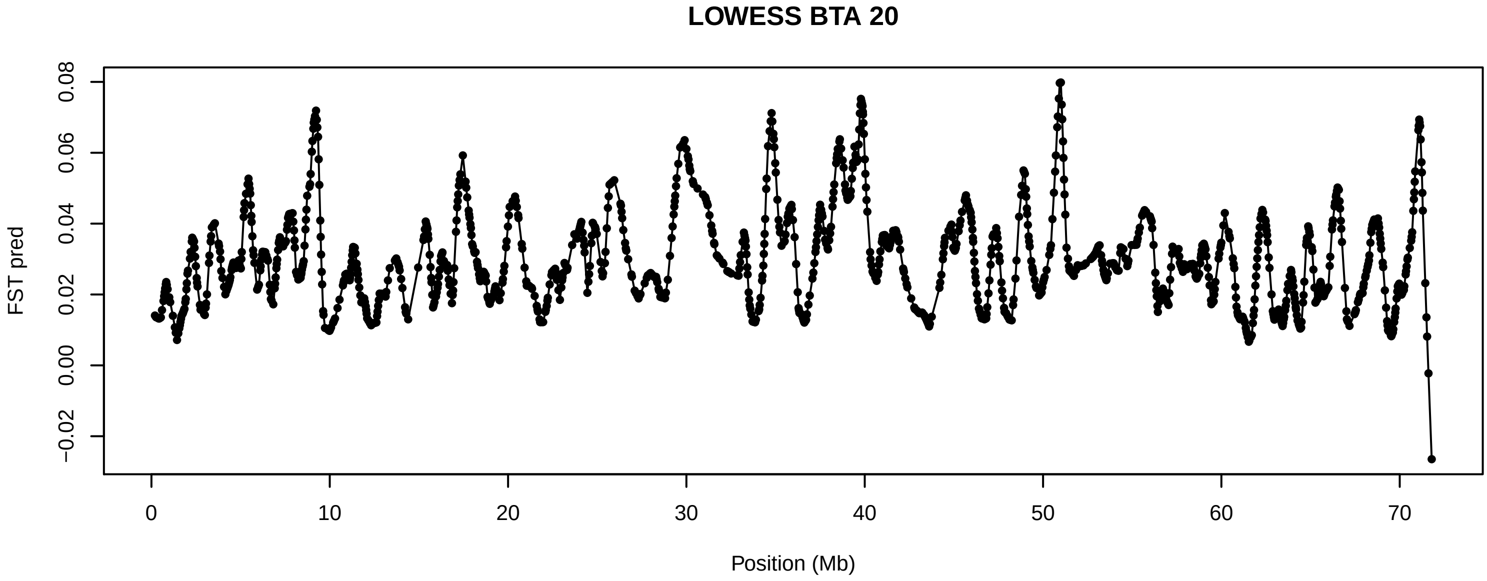
LOWESS BTA 20
FST pred
−0.02
0.00
0.02
0.04
0.06
0.08
0
10
20
30
40
50
60
70
Position (Mb)

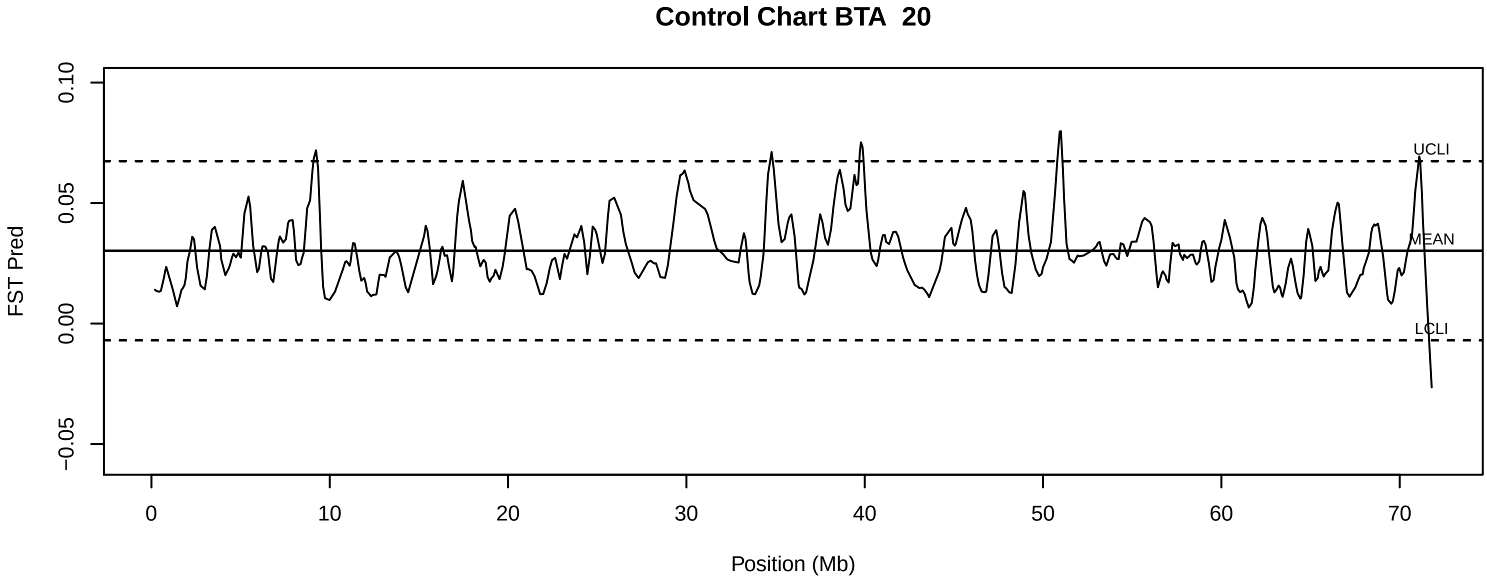
Control Chart BTA 20
FST Pred
−0.05
0.00
0.05
0.10
UCLI
MEAN
LCLI
0
10
20
30
40
50
60
70
Position (Mb)

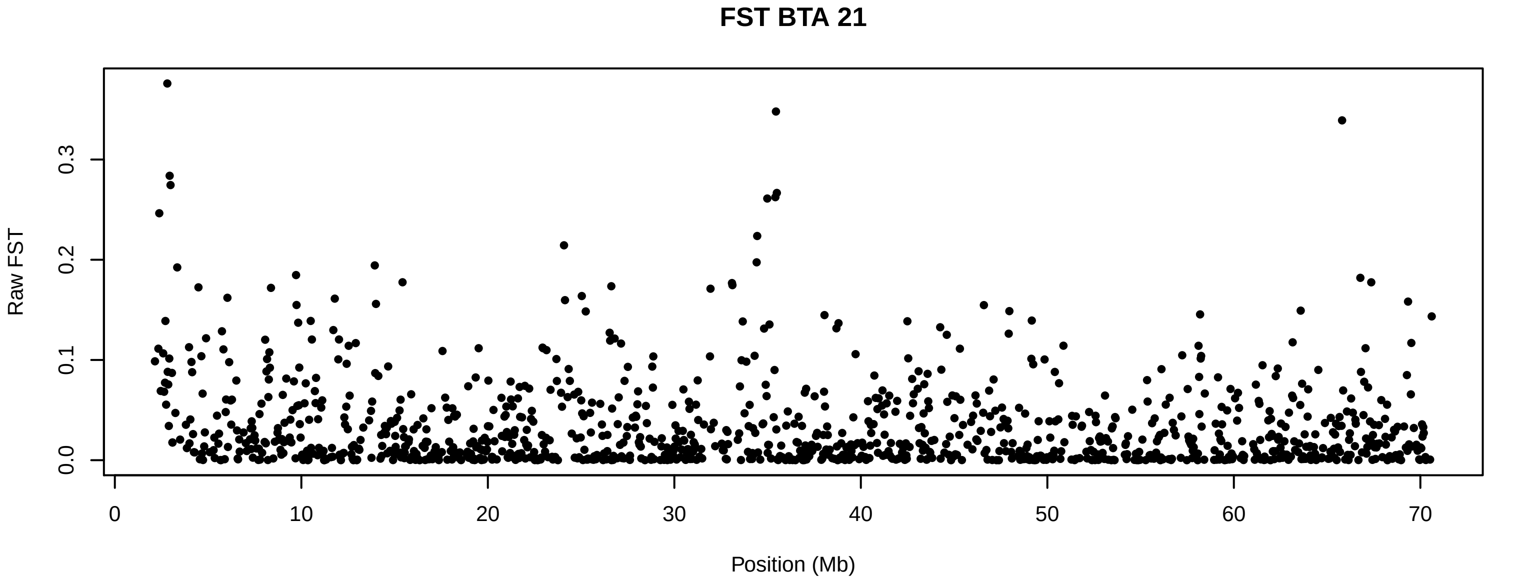
FST BTA 21
Raw FST
0.0
0.1
0.2
0.3
0 10 20 30 40 50 60 70
Position (Mb)

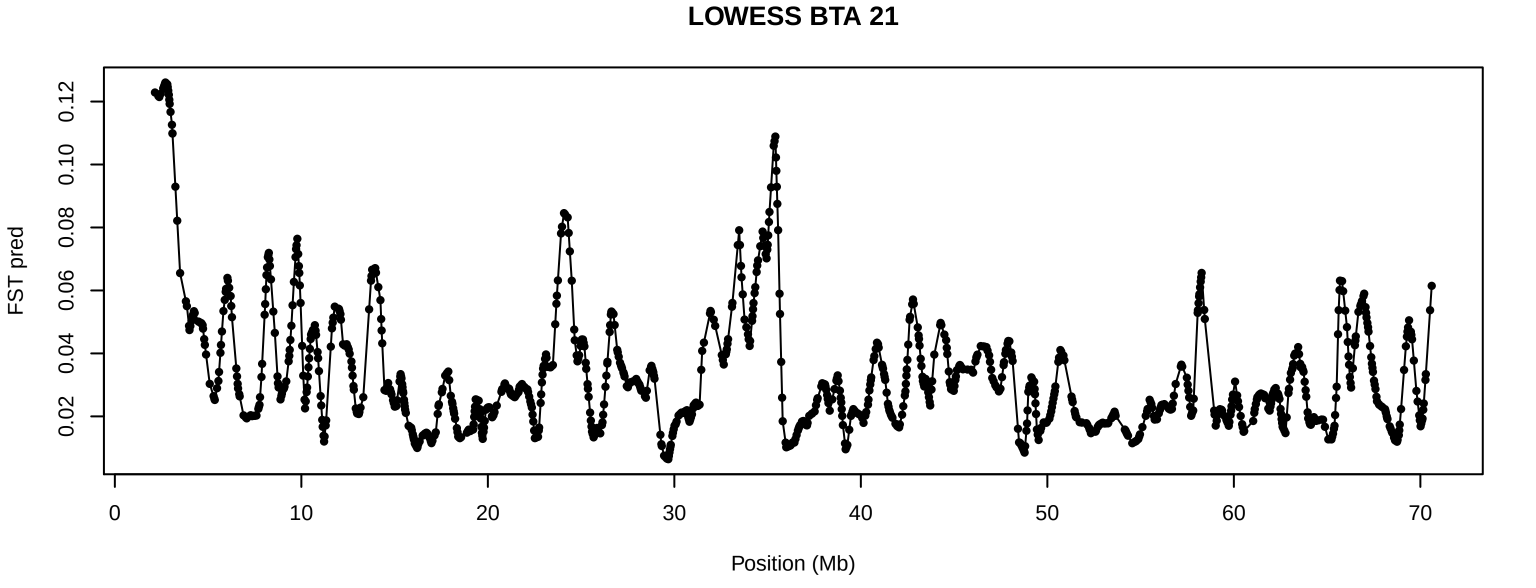
LOWESS BTA 21
FST pred
0.02
0.04
0.06
0.08
0.10
0.12
0 10 20 30 40 50 60 70
Position (Mb)

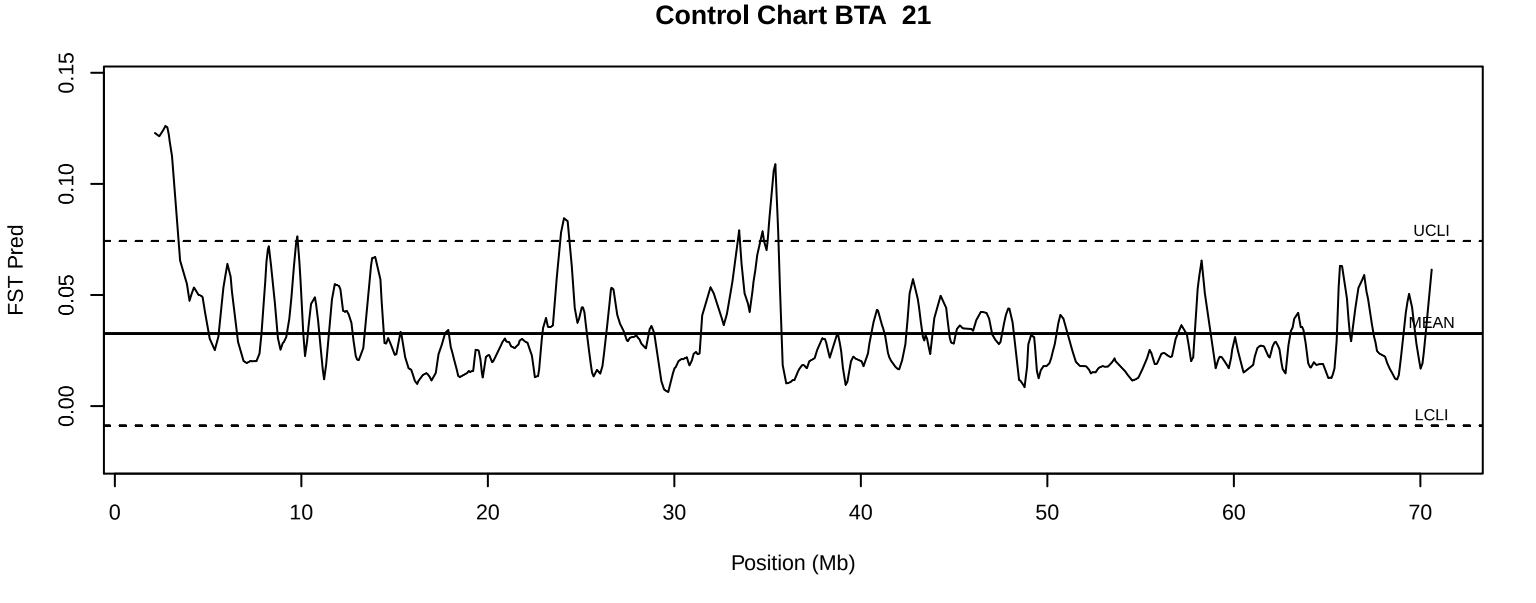
Control Chart BTA 21
FST Pred
0.00
0.05
0.10
0.15
UCLI
MEAN
LCLI
0 10 20 30 40 50 60 70
Position (Mb)

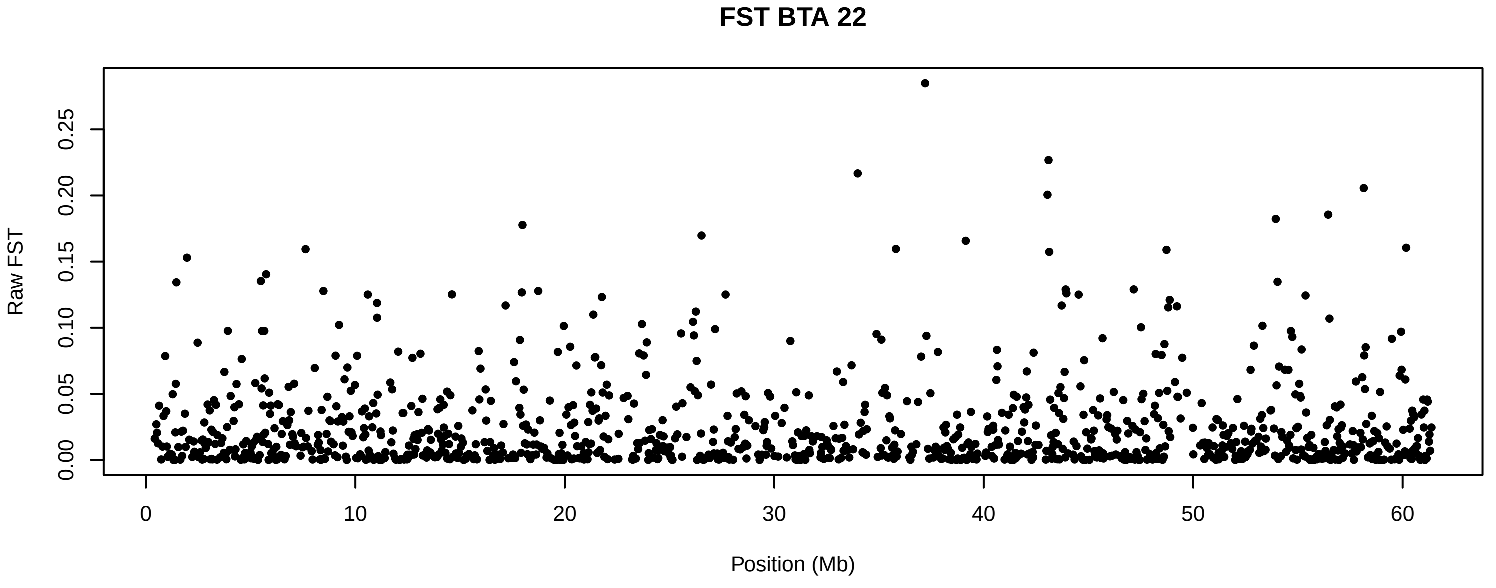
FST BTA 22
Raw FST
0.00
0.05
0.10
0.15
0.20
0.25
0
10
20
30
40
50
60
Position (Mb)

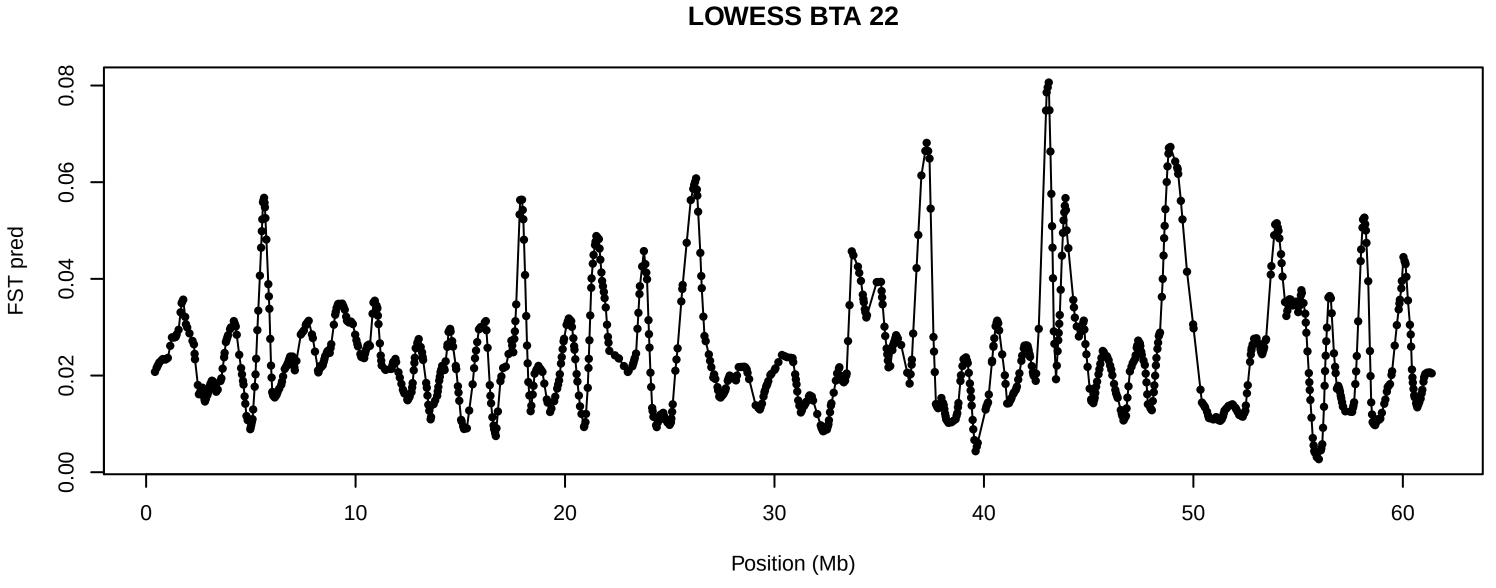
LOWESS BTA 22
FST pred
0.00
0.02
0.04
0.06
0.08
0
10
20
30
40
50
60
Position (Mb)

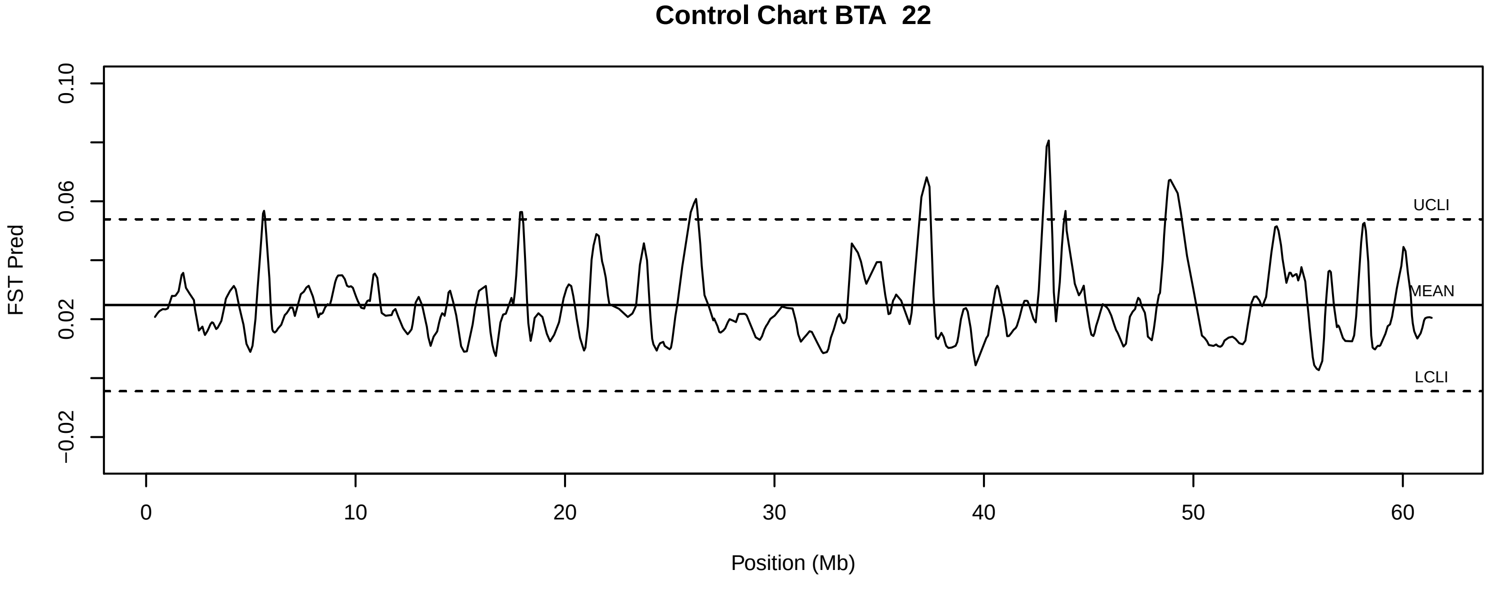
Control Chart BTA 22
FST Pred
−0.02
0.02
0.06
0.10
UCLI
MEAN
LCLI
0
10
20
30
40
50
60
Position (Mb)

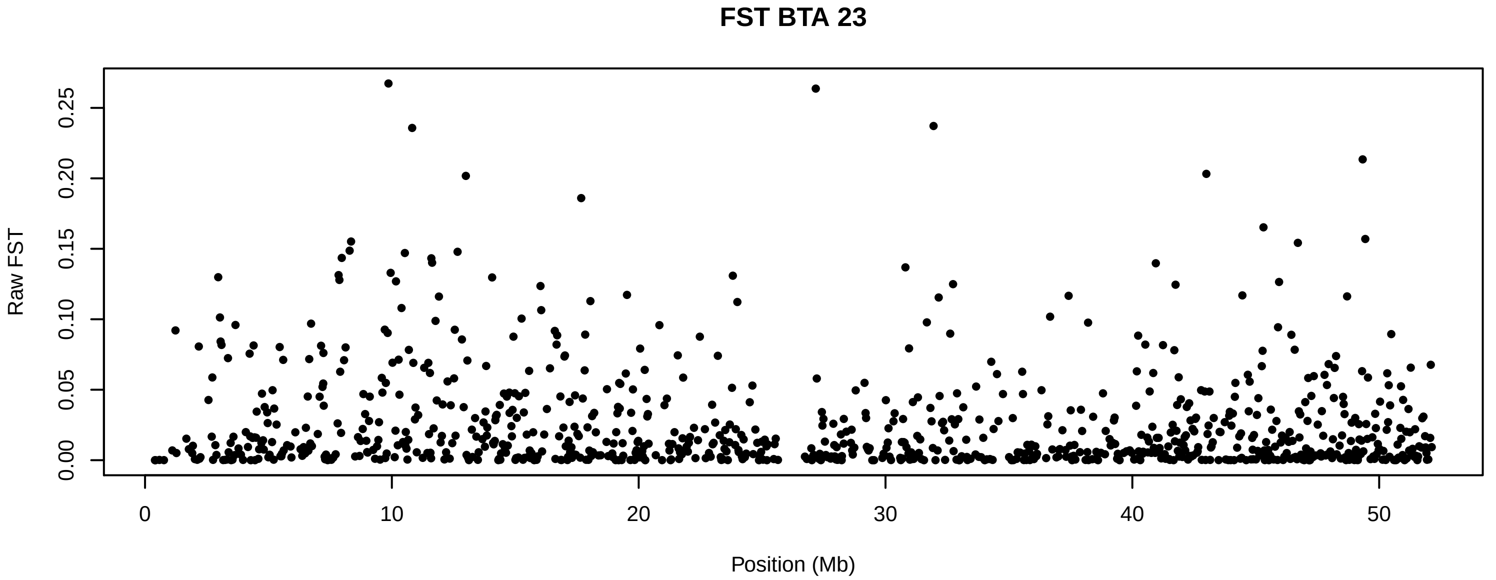
FST BTA 23
Raw FST
0.00
0.05
0.10
0.15
0.20
0.25
0
10
20
30
40
50
Position (Mb)

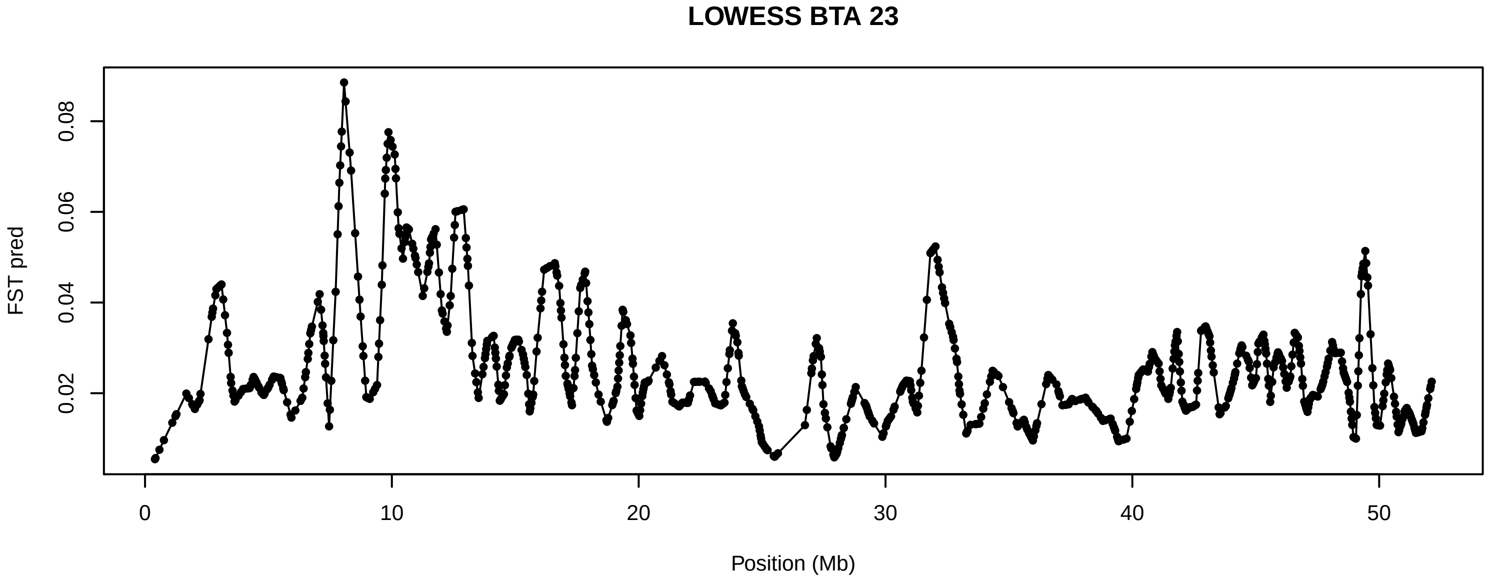
LOWESS BTA 23
FST pred
0.02
0.04
0.06
0.08
0
10
20
30
40
50
Position (Mb)

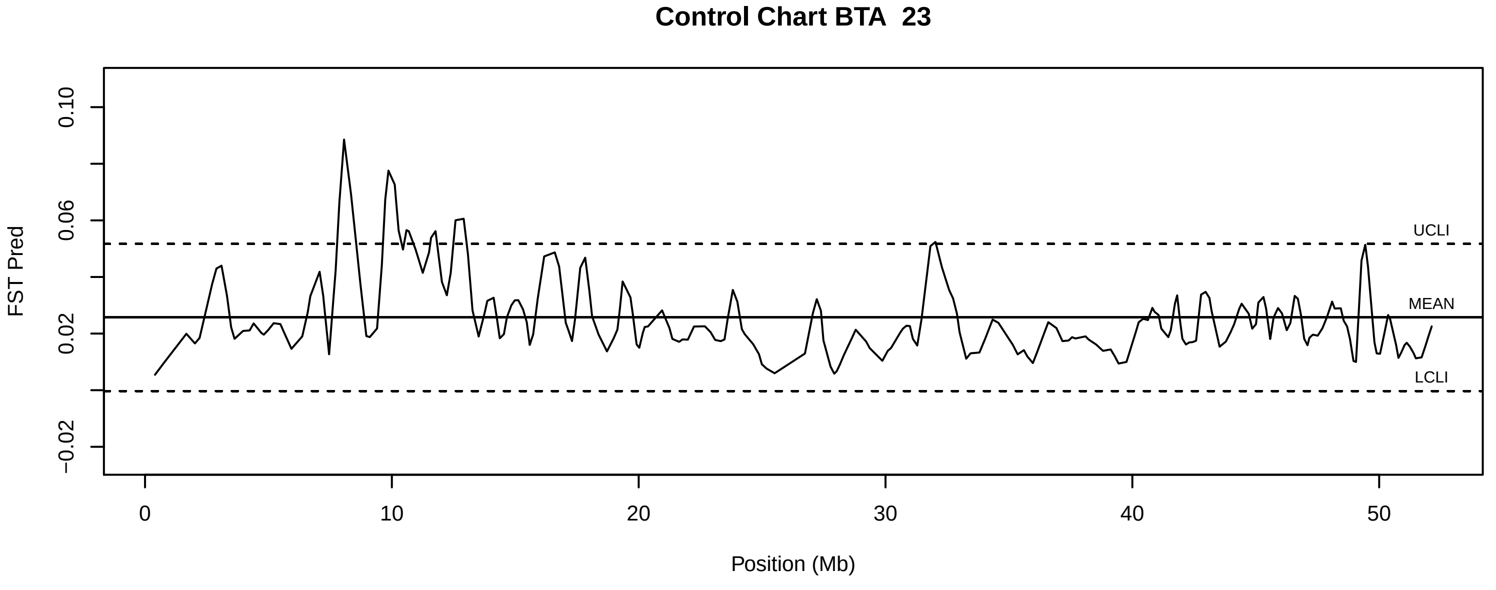
Control Chart BTA 23
FST Pred
−0.02
0.02
0.06
0.10
UCLI
MEAN
LCLI
0
10
20
30
40
50
Position (Mb)

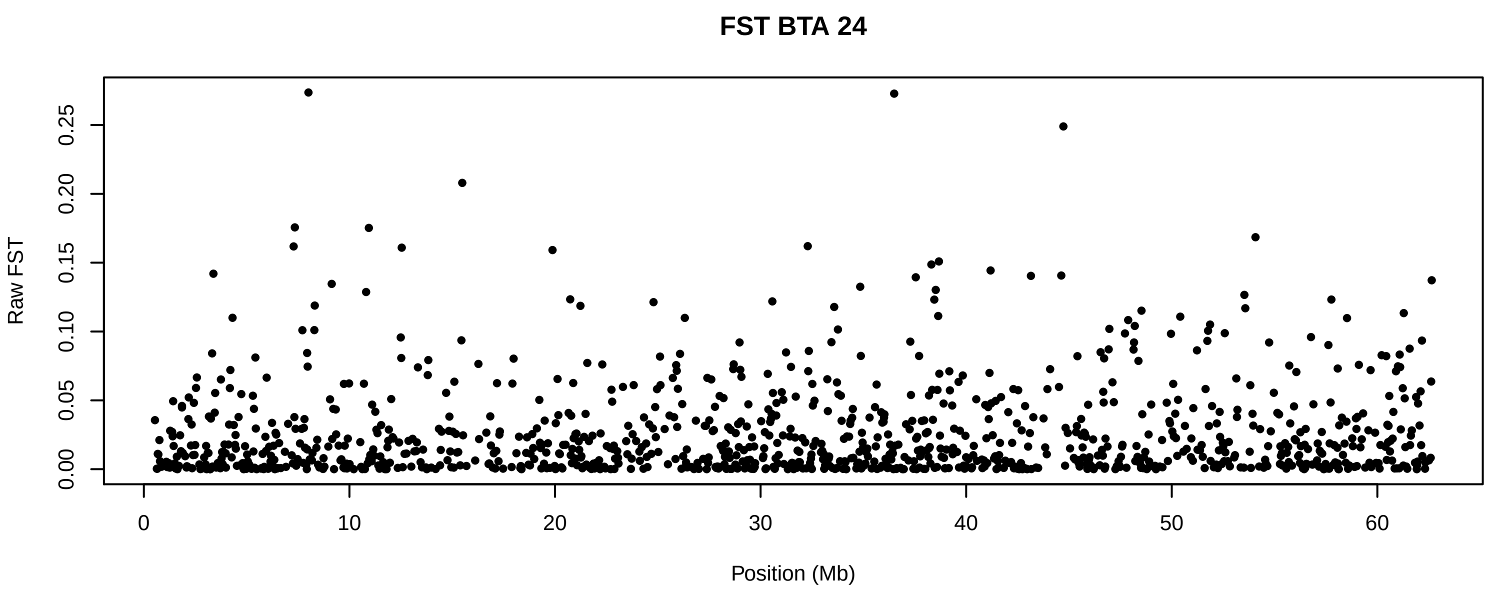

FST BTA 24
Raw FST
0.00
0.05
0.10
0.15
0.20
0.25
0
10
20
30
40
50
60
Position (Mb)

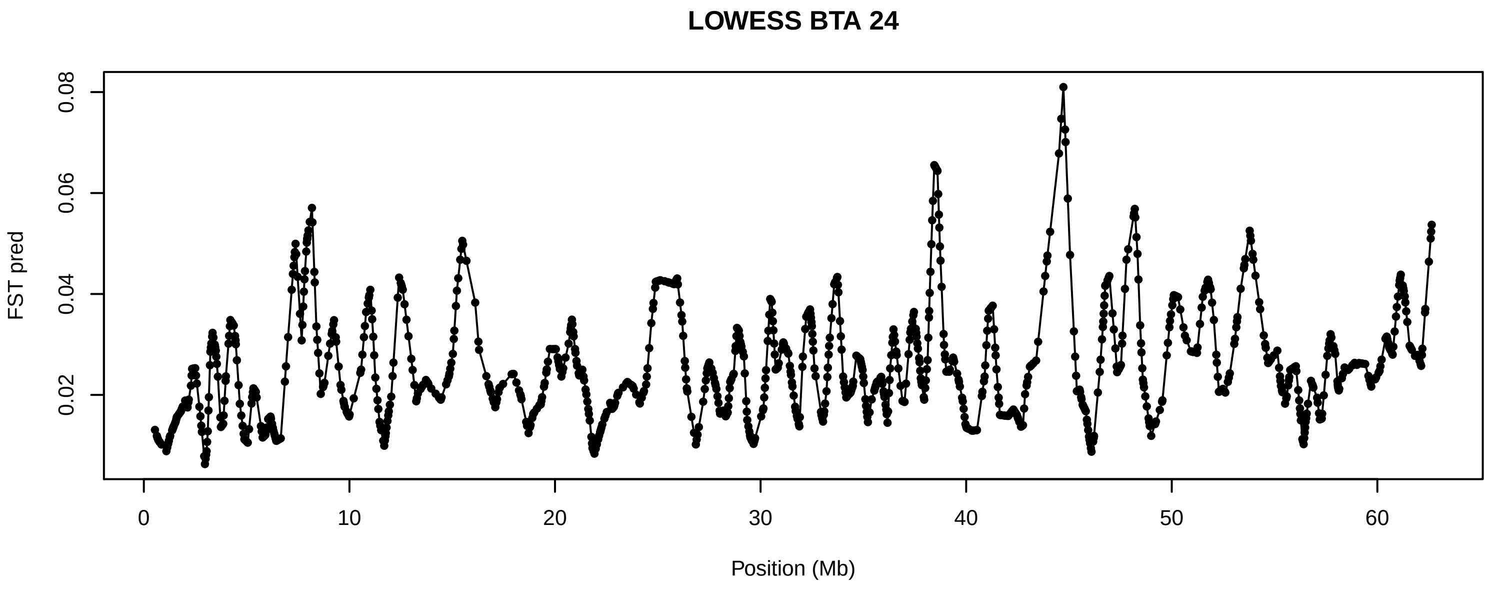

LOWESS BTA 24
FST pred
0.02
0.04
0.06
0.08
0
10
20
30
40
50
60
Position (Mb)

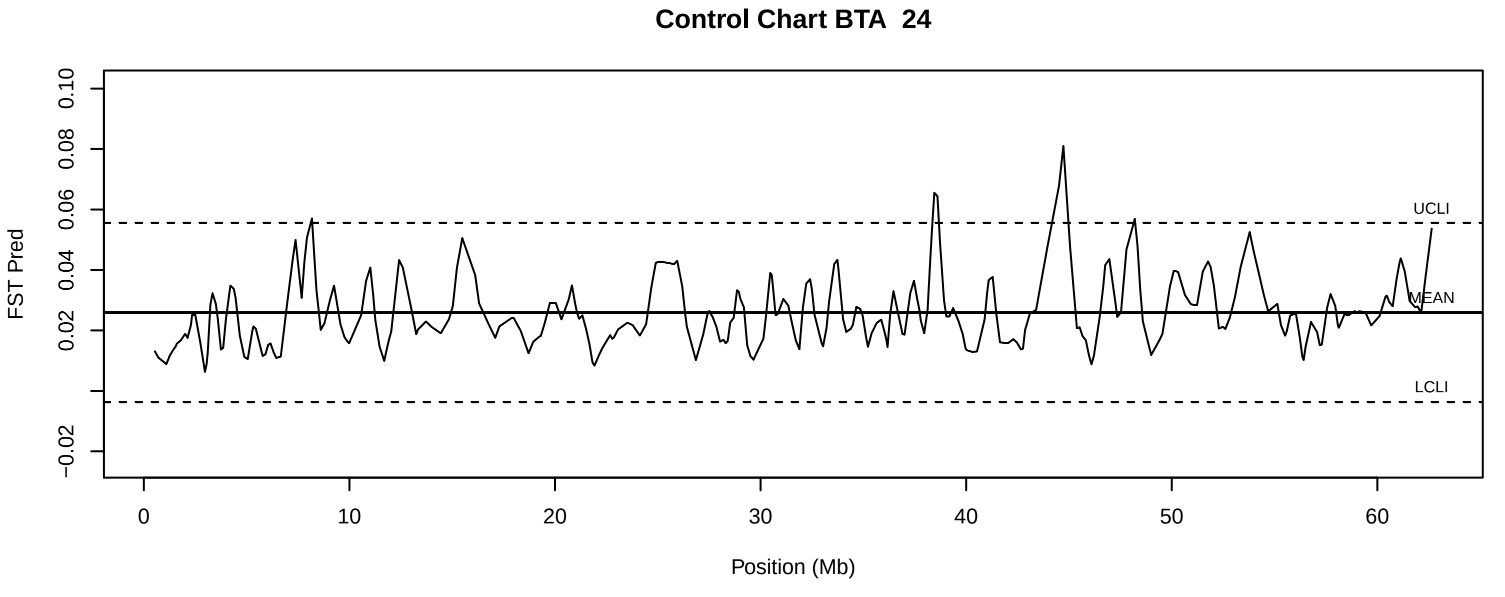

Control Chart BTA 24
FST Pred
−0.02
0.02
0.04
0.06
0.08
0.10
UCLI
MEAN
LCLI
0
10
20
30
40
50
60
Position (Mb)

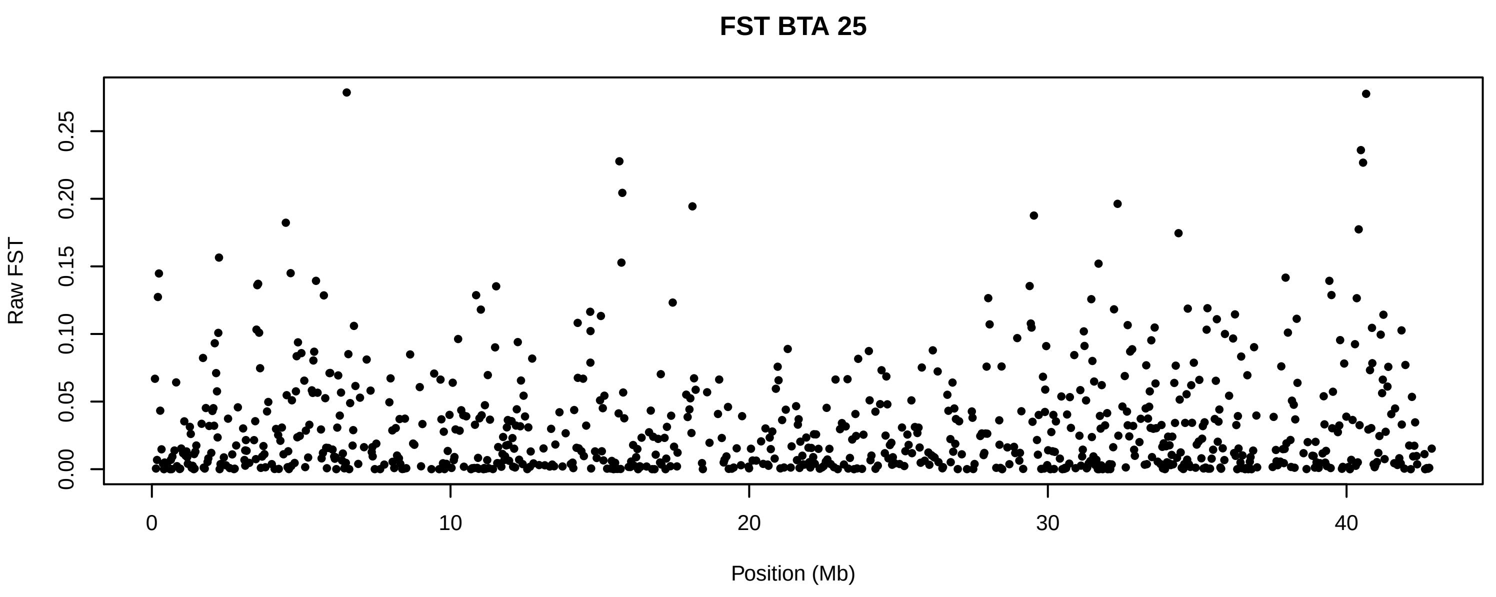
FST BTA 25
Raw FST
0.00
0.05
0.10
0.15
0.20
0.25
0
10
20
30
40
Position (Mb)

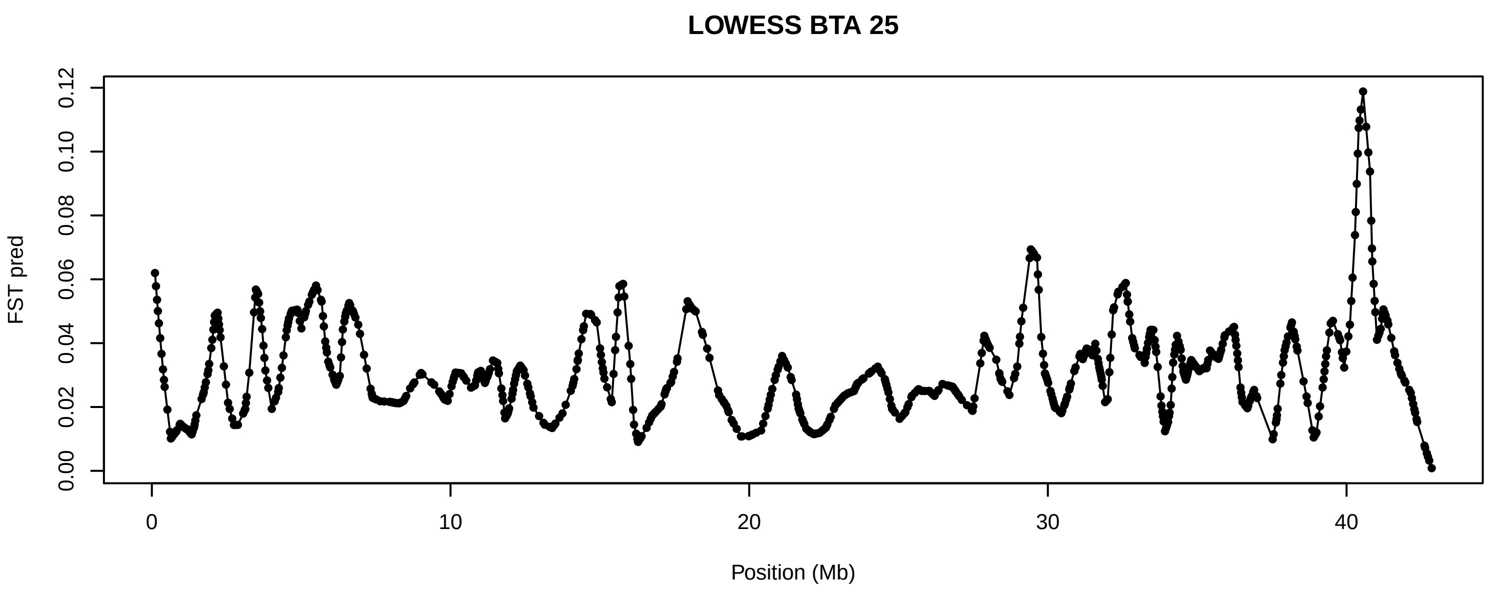
LOWESS BTA 25
FST pred
0.00
0.02
0.04
0.06
0.08
0.10
0.12
0
10
20
30
40
Position (Mb)

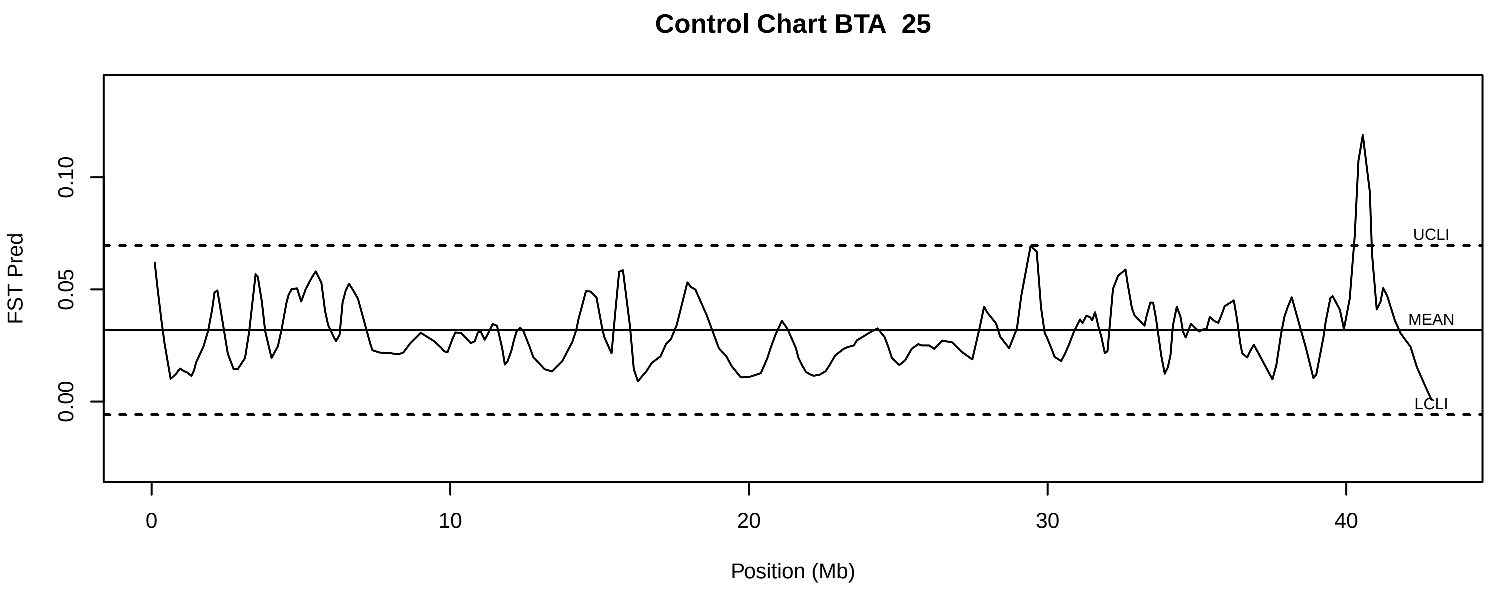
Control Chart BTA 25
FST Pred
0.00
0.05
0.10
UCLI
MEAN
LCLI
0
10
20
30
40
Position (Mb)

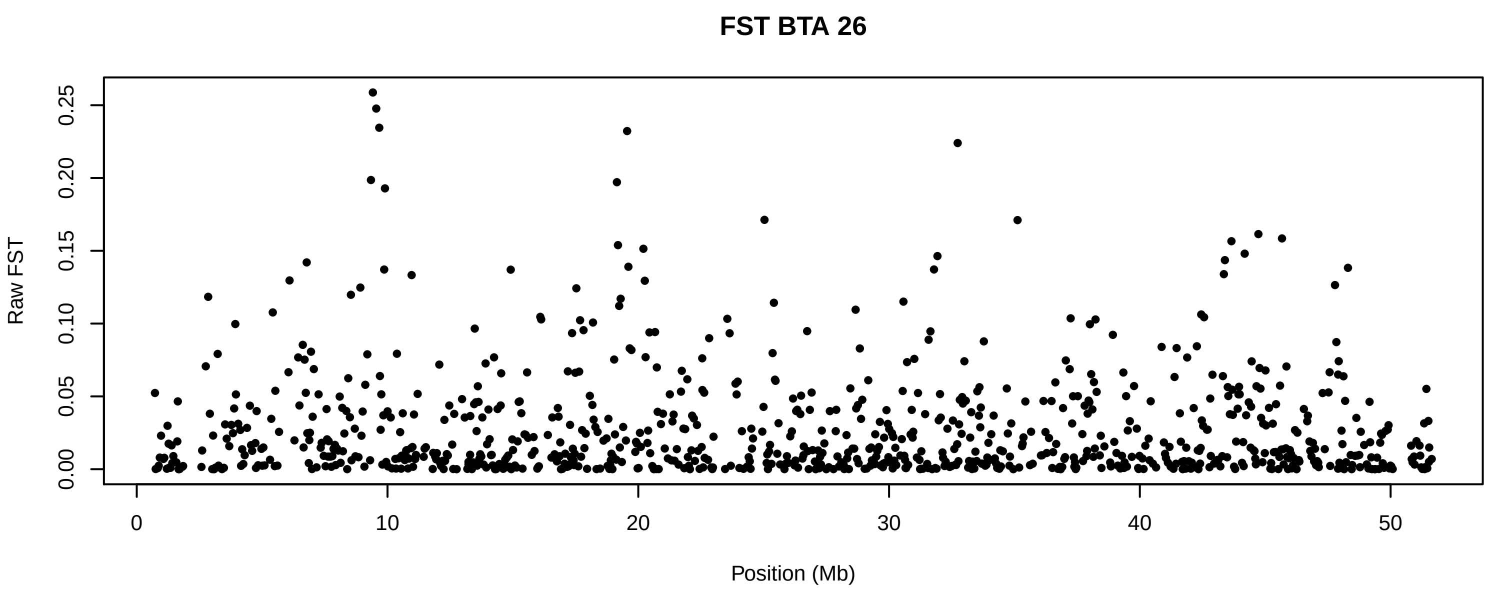
FST BTA 26
Raw FST
Position (Mb)

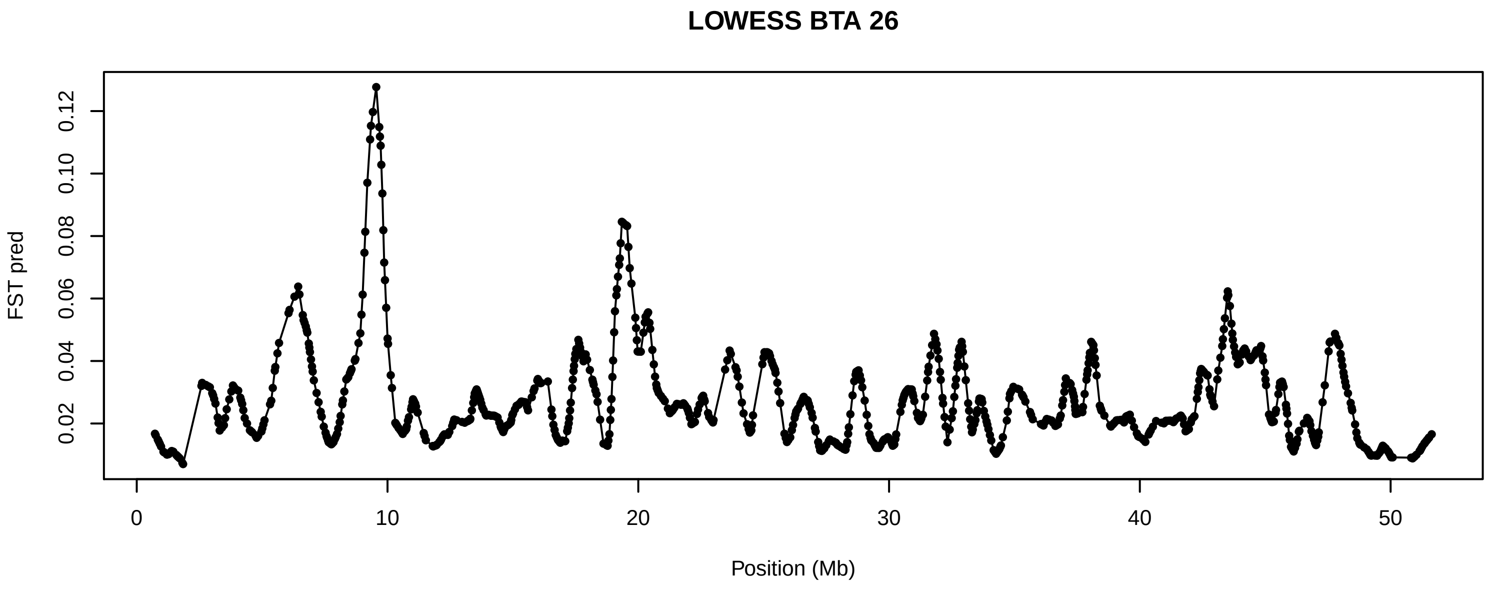
LOWESS BTA 26
FST pred
Position (Mb)

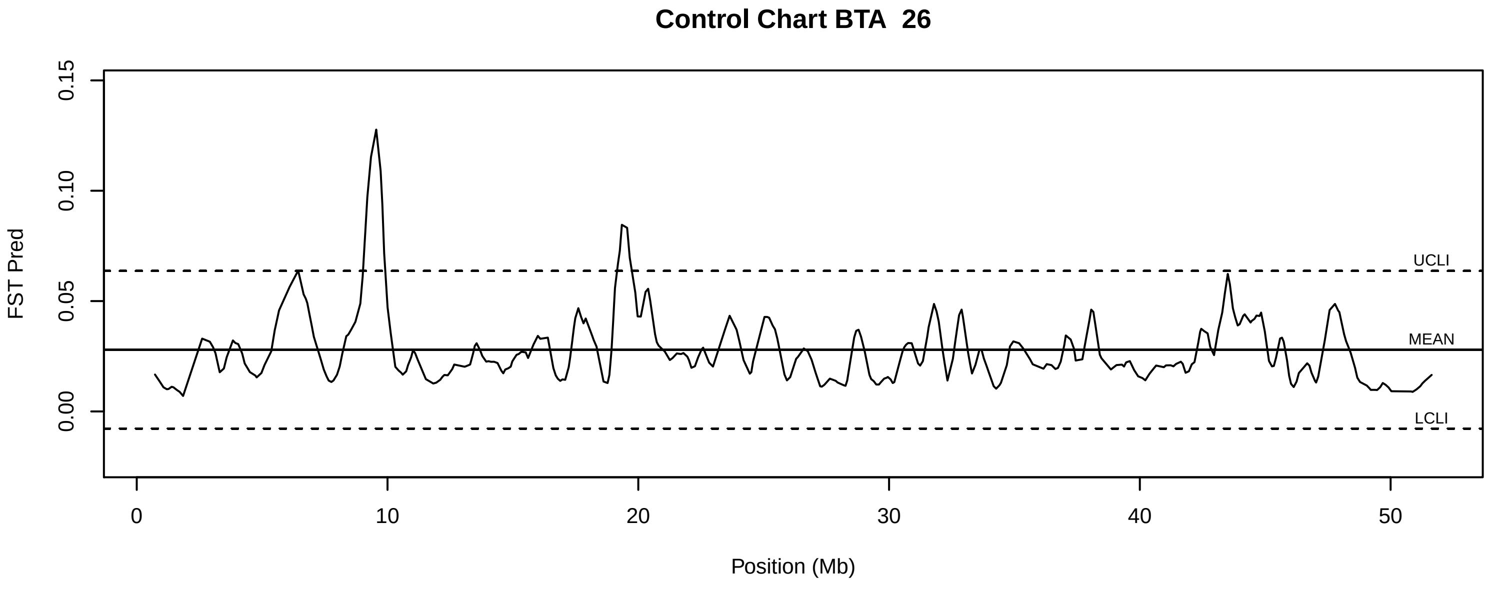
Control Chart BTA 26
FST Pred
UCLI
MEAN
LCLI
Position (Mb)

FST BTA 27

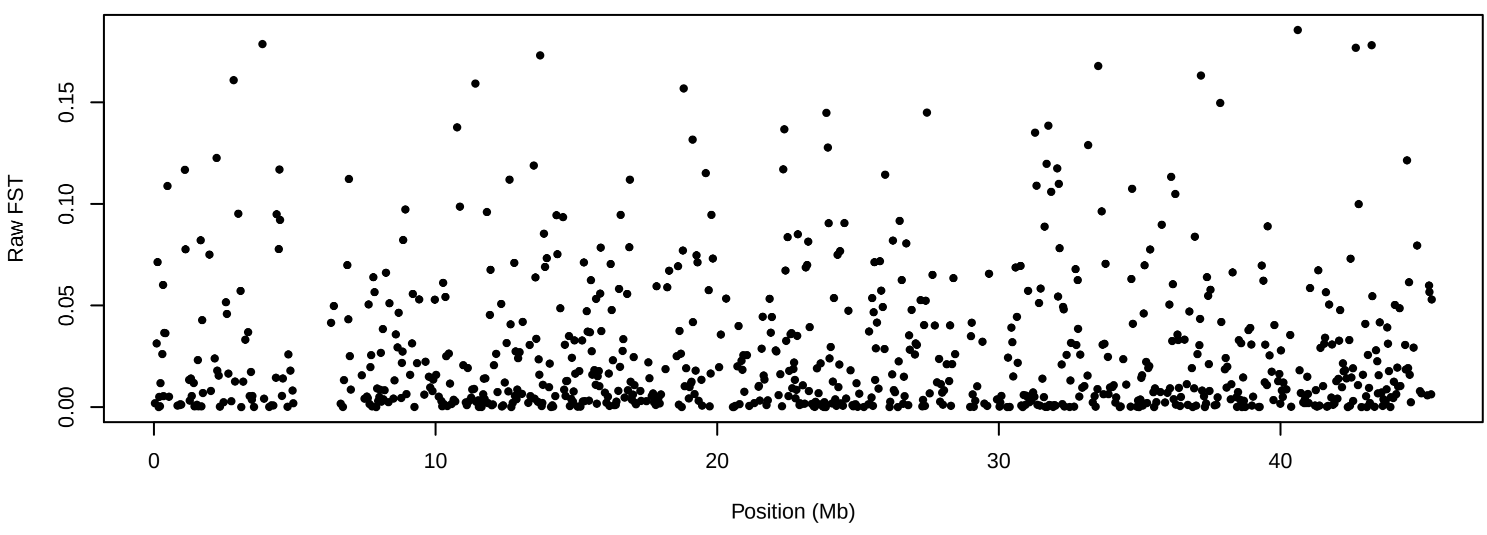

LOWESS BTA 27

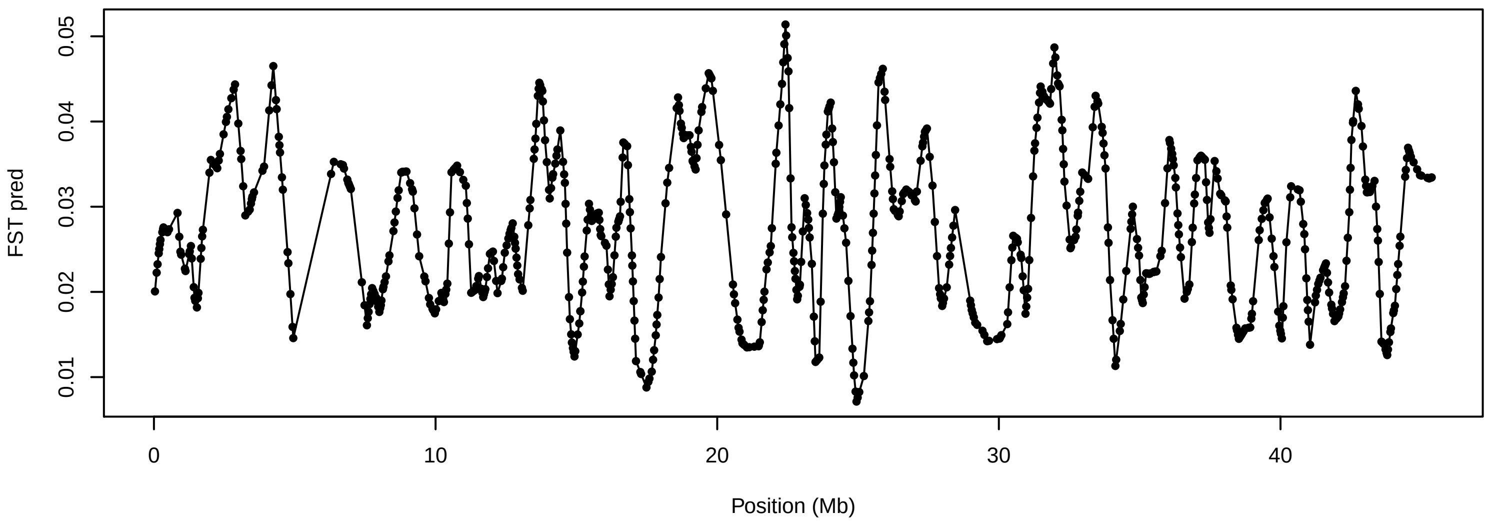

Control Chart BTA 27

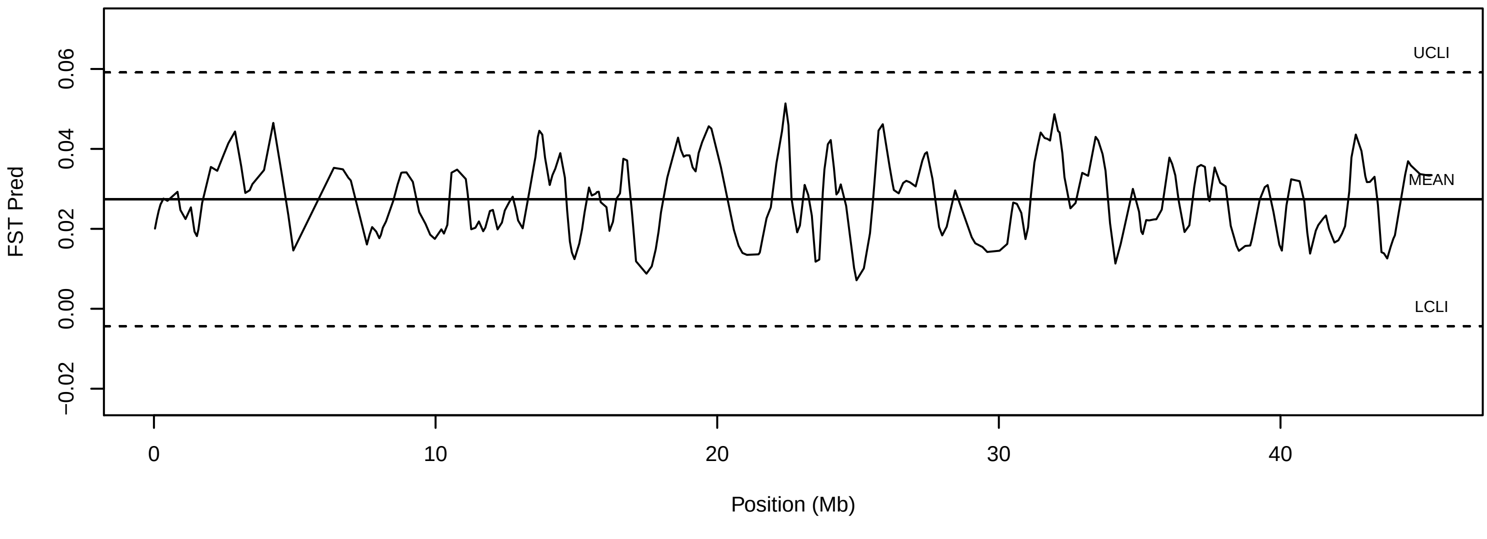

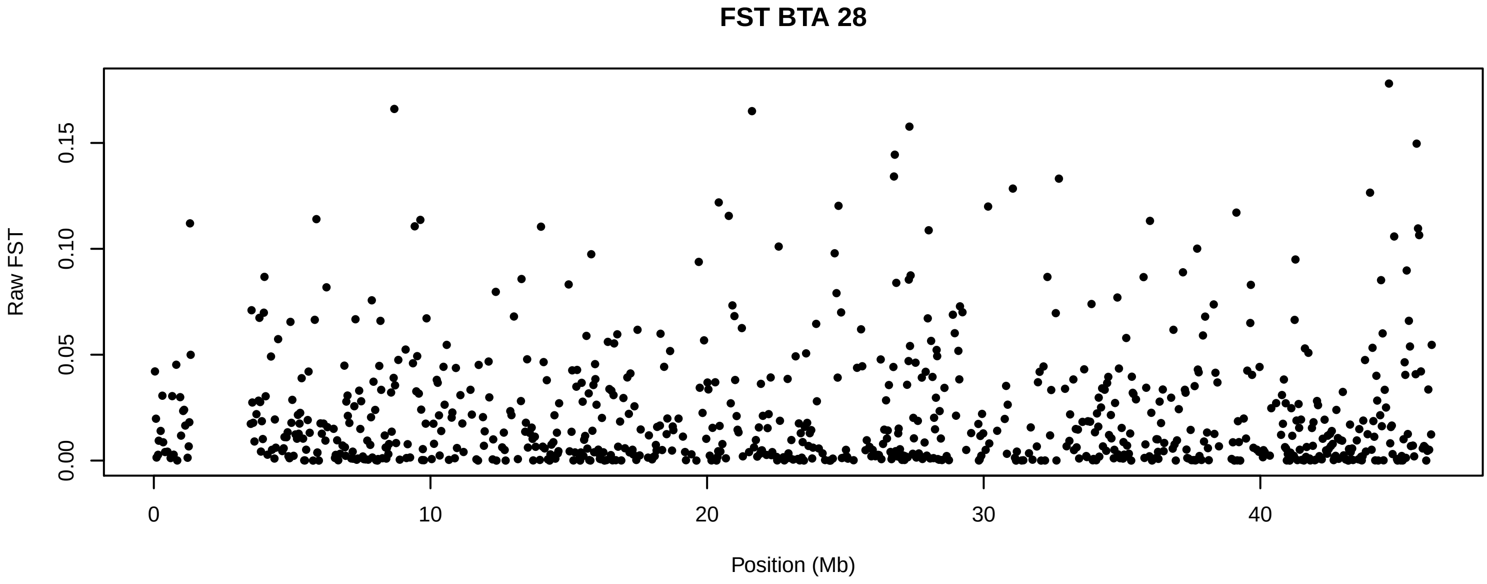
FST BTA 28
Raw FST
0.00
0.05
0.10
0.15
0
10
20
30
40
Position (Mb)

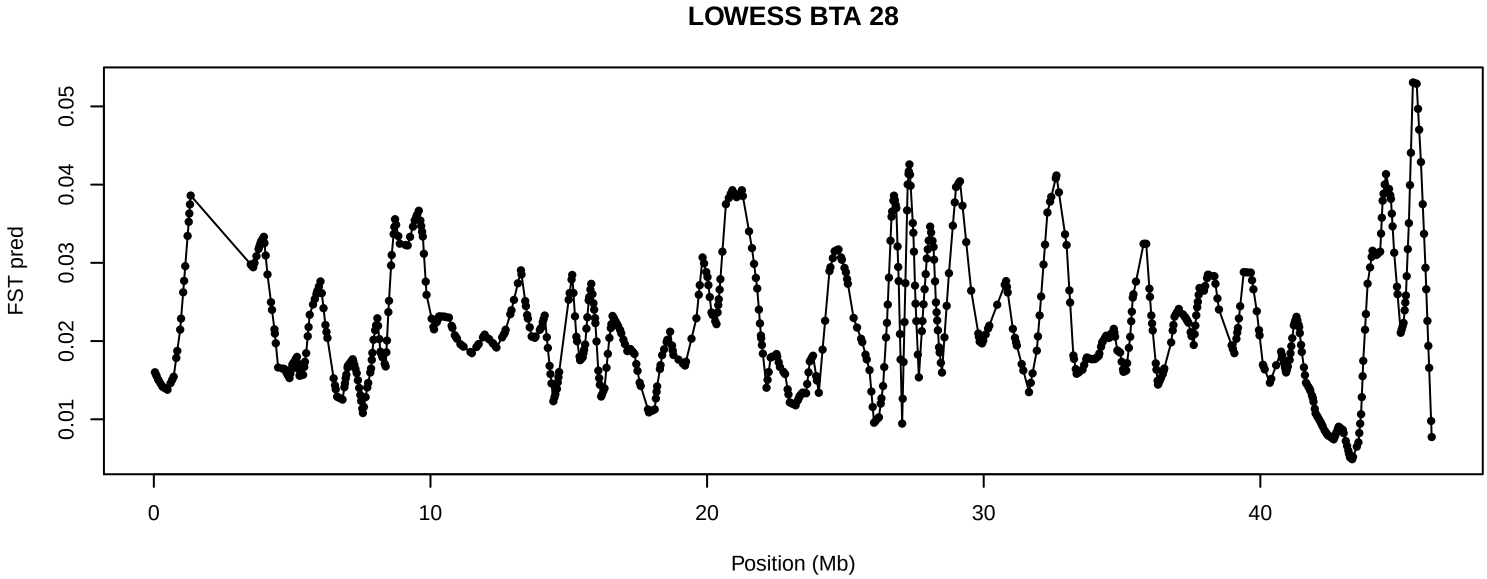
LOWESS BTA 28
FST pred
0.01
0.02
0.03
0.04
0.05
0
10
20
30
40
Position (Mb)

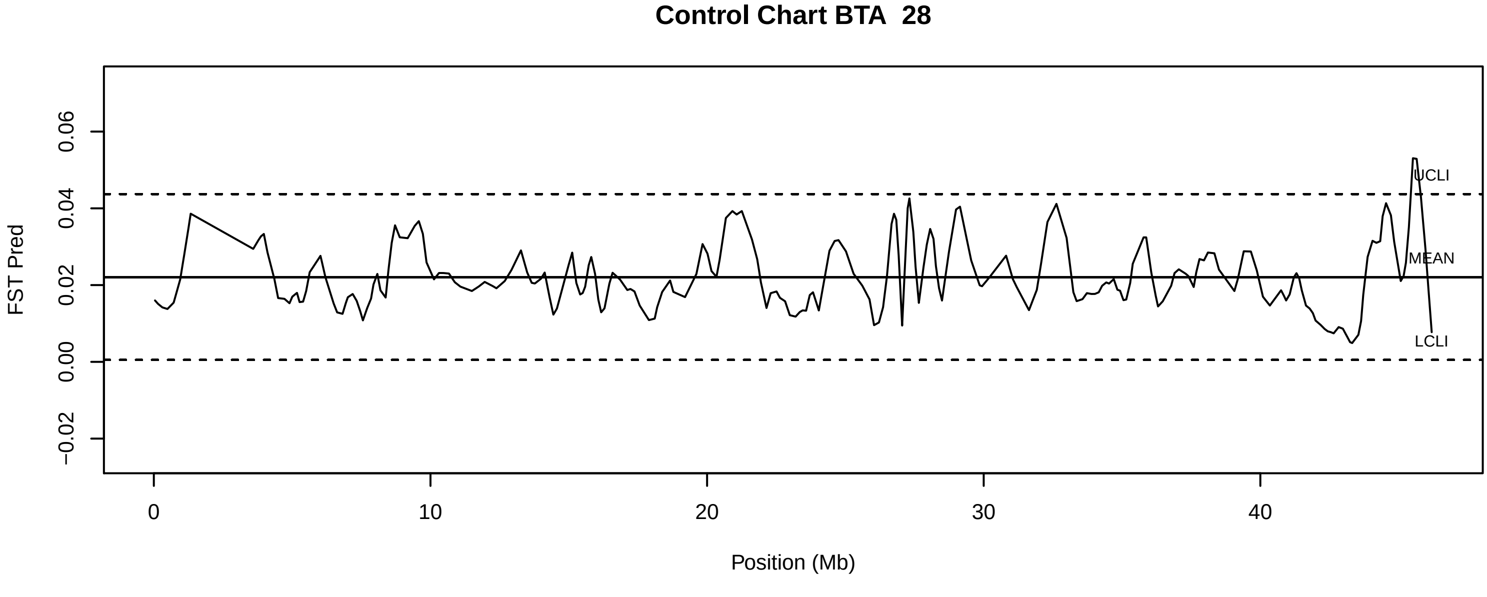
Control Chart BTA 28
FST Pred
−0.02
0.00
0.02
0.04
0.06
UCLI
MEAN
LCLI
0
10
20
30
40
Position (Mb)

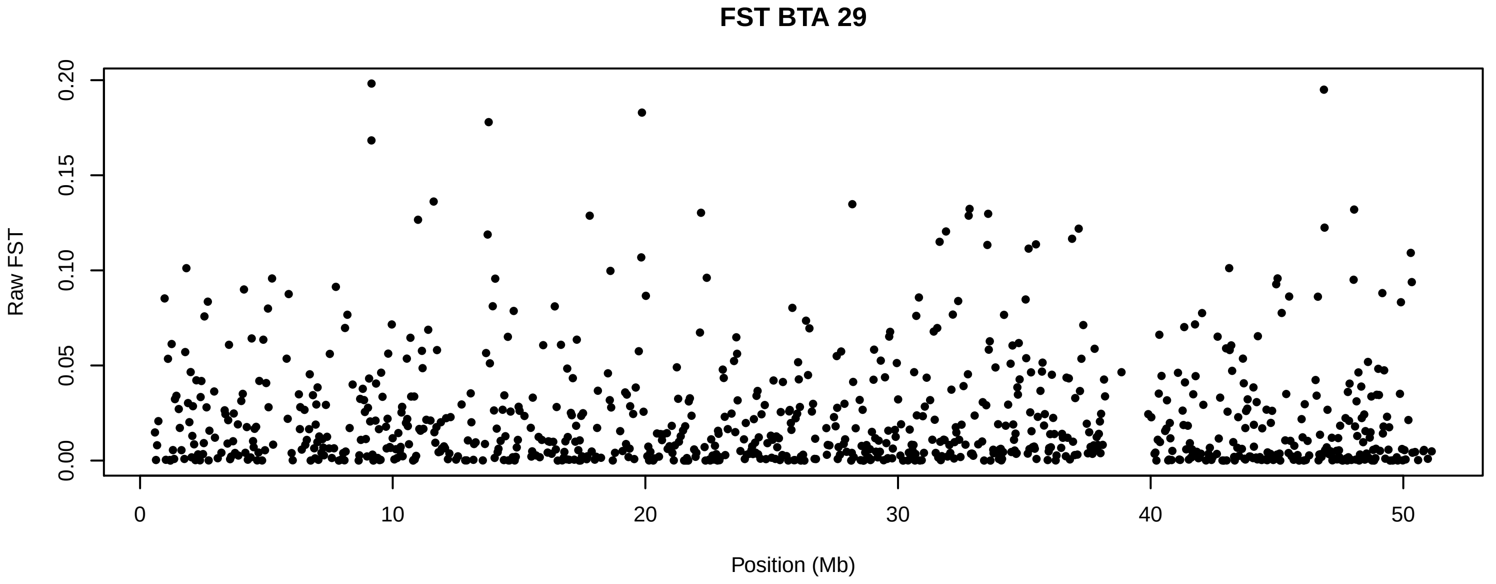
FST BTA 29
Raw FST
0.00
0.05
0.10
0.15
0.20
0
10
20
30
40
50
Position (Mb)

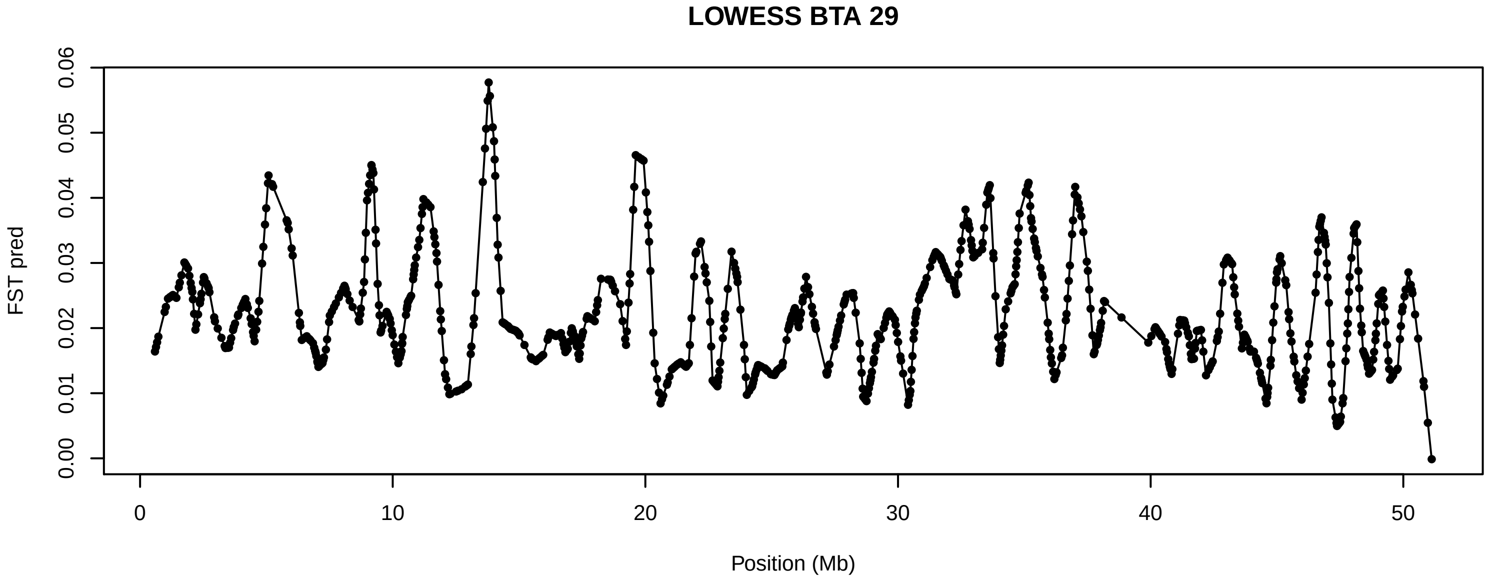
LOWESS BTA 29
FST pred
0.00
0.01
0.02
0.03
0.04
0.05
0.06
0
10
20
30
40
50
Position (Mb)

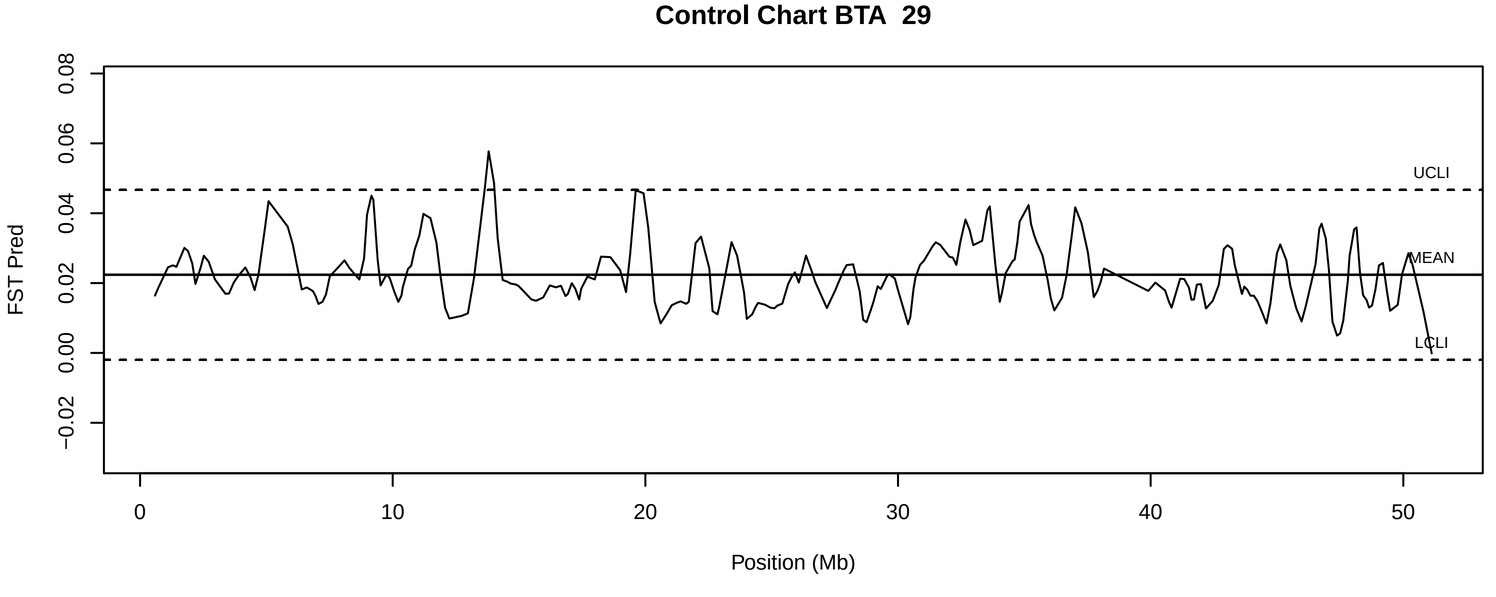
Control Chart BTA 29
FST Pred
−0.02
0.00
0.02
0.04
0.06
0.08
UCLI
MEAN
LCLI
0
10
20
30
40
50
Position (Mb)
