# Supplementary material for: Detection of selection signatures in Piemontese and Marchigiana cattle, two breeds with similar production aptitudes but different selection histories
Source: Genet Sel Evol. 2015 Jun 23;47(1):52. doi: 10.1186/s12711-015-0128-2 (PMC4476081; doi:10.1186/s12711-015-0128-2)
Supplement: Supplementary file 3 — List of 933 genes annotated in cattle detected using the control chart method. Description: This list includes all the bovine annotated genes derived from Bos taurus UMD 3.1/bosTau6 assembly that are present in the 0.5 Mb interval (0.25 Mb upstream and downstream) considered for each significant SNP using the control chart method. [file 12711_2015_128_MOESM3_ESM.pdf]

gene name	start bp	end bp	BTA
SOD1	3.113.947	3.122.613	1
RPL35A	70.788.694	70.792.140	1
FYTTD1	70.954.510	70.984.201	1
IQCG	70.792.453	70.838.107	1
KIAA0226	70.996.321	71.044.883	1
TNK2	71.207.393	71.230.157	1
TFRC	71.259.941	71.287.252	1
ZDHHC19	71.396.753	71.409.483	1
PCYT1A	71.431.932	71.484.412	1
SLC51A	71.416.989	71.434.990	1
TCTEX1D2	71.489.283	71.516.789	1
UBXN7	71.542.375	71.594.698	1
C1H3orf43	71.654.671	71.661.825	1
RNF168	71.629.569	71.653.254	1
FBXO45	71.710.337	71.721.212	1
WDR53	71.697.096	71.709.973	1
NRROS	71.765.080	71.791.876	1
NLGN1	93.626.046	94.742.072	1
CHST2	126.870.791	126.874.541	1
PAQR9	127.041.079	127.043.159	1
SLC9A9	126.040.088	126.704.343	1
PCOLCE2	127.132.776	127.229.654	1
TRPC1	127.257.294	127.318.223	1
PLS1	127.323.031	127.416.647	1
LOC511034	137.110.870	137.499.189	1
NPHP3	137.902.567	137.957.297	1
UBA5	137.960.487	137.993.315	1
ACAD11	137.993.896	138.112.991	1
ACKR4	138.069.155	138.071.195	1
WDR33	4.608.386	4.717.754	2
LIMS2	4.780.175	4.819.192	2
GPR17	4.805.764	4.811.802	2
PROC	5.034.438	5.044.966	2
ERCC3	5.181.036	5.211.359	2
MIR2350	5.398.826	5.398.902	2
BIN1	5.350.653	5.407.857	2
NAB1	5.567.015	5.603.683	2
INPP1	5.867.811	5.903.148	2
C2H2orf88	6.038.401	6.113.008	2
MSTN	6.213.565	6.220.196	2
PMS1	6.395.003	6.511.175	2
SLC40A1	6.716.590	6.740.329	2
WDR75	6.875.263	6.916.093	2
OSGEPL1	6.531.463	6.542.370	2
ORMDL1	6.511.309	6.523.391	2

ASNSD1	6.605.442	6.616.484	2
MIR2917	7.214.195	7.214.275	2
COL3A1	7.317.286	7.356.904	2
GULP1	7.830.306	8.068.230	2
TFPI	8.786.392	8.884.005	2
CALCRL	8.901.431	9.030.990	2
ZSWIM2	9.502.290	9.523.394	2
FAM171B	9.563.952	9.590.363	2
ITGAV	9.651.630	9.760.100	2
MIR2351	9.700.673	9.700.749	2
ZC3H15	9.849.223	9.870.250	2
KIF5C	47.315.196	47.472.004	2
MIR2354	47.459.009	47.459.084	2
HNMT	59.376.268	59.417.330	2
UBXN4	61.919.638	61.949.692	2
MIR128-1	62.007.751	62.007.833	2
MCM6	61.821.929	61.856.346	2
CXCR4	61.582.124	61.585.838	2
DARS	61.722.451	61.787.888	2
LCT	61.867.079	61.915.814	2
RAB3GAP1	62.457.395	62.568.130	2
ABCD3	49.136.316	49.225.714	3
F3	49.110.155	49.121.034	3
ARHGAP29	49.392.813	49.474.286	3
ABCA4	49.541.294	49.686.388	3
DNTTIP2	49.770.191	49.781.520	3
GCLM	49.741.374	49.759.101	3
MIR760	49.797.089	49.797.206	3
BCAR3	49.850.608	50.069.718	3
ZNF326	53.384.336	53.419.678	3
LRRC8C	53.717.829	53.810.701	3
MIR2285K-5	53.564.232	53.564.313	3
LRRC8D	53.475.692	53.606.716	3
CCBL2	55.093.504	55.153.829	3
PKN2	55.231.809	55.365.736	3
GTF2B	55.179.368	55.210.379	3
CYR61	58.678.776	58.681.686	3
LRRC7	75.355.802	75.744.768	3
NFIA	84.749.653	85.167.847	3
MYSM1	87.924.697	87.966.272	3
OMA1	88.054.069	88.119.290	3
TWIST1	27.853.326	27.855.320	4
GRM3	33.769.687	34.021.297	4
CHN2	67.063.891	67.105.085	4
PRR15	67.039.118	67.040.699	4
ZNF800	92.423.261	92.444.888	4

GRM8	91.470.675	92.316.363	4
MIR592	92.126.803	92.126.899	4
GCC1	92.617.108	92.620.507	4
ARF5	92.624.396	92.627.378	4
FSCN3	92.629.381	92.637.487	4
SND1	92.679.168	93.108.508	4
NRF1	94.264.902	94.359.401	4
MIR96	94.411.192	94.411.295	4
UBE2H	94.467.615	94.565.768	4
ZC3HC1	94.634.226	94.650.468	4
KLHDC10	94.676.715	94.737.327	4
MIR183	94.411.430	94.411.500	4
MIR182	94.406.699	94.406.810	4
SSMEM1	94.800.227	94.809.913	4
MIR671	114.614.901	114.615.019	4
TMUB1	114.455.521	114.457.946	4
CHPF2	114.610.855	114.615.301	4
CDK5	114.429.162	114.433.469	4
SLC4A2	114.438.014	114.450.607	4
NOS3	114.375.966	114.394.483	4
FASTK	114.450.704	114.455.203	4
ABCB8	114.404.771	114.422.140	4
KCNH2	114.329.662	114.357.980	4
MIR6525	114.451.860	114.451.945	4
ABCF2	114.587.368	114.602.056	4
SMARCD3	114.615.442	114.621.742	4
ACCN3	114.424.077	114.428.038	4
WDR86	114.730.381	114.755.250	4
NUB1	114.689.766	114.725.191	4
RHEB	114.803.156	114.855.282	4
XRCC2	115.796.820	115.821.949	4
AMHR2	26.753.259	26.759.487	5
ATF7	26.580.497	26.671.702	5
PCBP2	26.702.494	26.721.581	5
PRR13	26.730.146	26.733.390	5
NPFF	26.679.263	26.680.063	5
CALCOCO1	26.489.543	26.503.713	5
MAP3K12	26.695.547	26.701.886	5
TARBP2	26.681.521	26.686.845	5
ATP5G2	26.535.456	26.539.249	5
SP1	26.769.553	26.804.655	5
SP7	26.848.176	26.855.851	5
AAAS	26.862.073	26.878.095	5
ESPL1	26.891.700	26.913.470	5
PFDN5	26.886.090	26.890.186	5
C5H12orf10	26.878.372	26.885.790	5

IGFBP6	27.044.007	27.047.853	5
SPRYD3	27.054.220	27.068.013	5
ITGB7	26.958.460	26.973.298	5
SOAT2	27.032.005	27.042.825	5
MFSD5	26.919.412	26.921.681	5
RARG	26.935.717	26.956.344	5
EIF4B	27.091.607	27.115.819	5
KRT18	27.171.924	27.175.575	5
KRT8_AA	27.213.668	27.221.171	5
KRT79	27.271.879	27.282.428	5
KRT78	27.258.341	27.266.308	5
KRT4	27.291.349	27.298.264	5
KRT73	27.443.920	27.454.390	5
KRT72	27.464.195	27.476.582	5
KRT74	27.487.719	27.496.237	5
KRT71	27.505.152	27.513.513	5
CAND1	46.719.645	46.753.948	5
TMPO	63.027.193	63.052.822	5
ANKS1B	63.229.075	63.658.499	5
SLC25A3	63.086.352	63.092.547	5
APAF1	63.125.176	63.207.285	5
IKBIP	63.097.772	63.121.576	5
GLT8D2	67.994.624	68.036.627	5
TDG	67.973.221	67.994.057	5
HSP90B1	67.940.791	67.959.532	5
STAB2	67.612.322	67.784.873	5
C5H12orf73	67.960.358	67.967.214	5
NFYB	68.109.726	68.127.878	5
MIR2284Z-2	68.142.724	68.569.714	5
HCFC2	68.052.160	68.097.172	5
MIR2435	68.192.523	68.192.593	5
EID3	68.253.019	68.254.500	5
TXNRD1	68.239.610	68.302.679	5
CHST11	68.385.644	68.645.774	5
SLC41A2	68.700.509	68.842.780	5
ALDH1L2	68.880.983	68.941.773	5
C5H12orf45	68.866.723	68.876.106	5
APPL2	69.039.868	69.091.742	5
C5H12orf75	69.161.771	69.206.621	5
NUAK1	69.816.582	69.892.512	5
TCP11L2	70.013.666	70.054.235	5
POLR3B	70.062.607	70.178.439	5
RFX4	70.323.197	70.395.056	5
CRY1	70.604.519	70.701.030	5
MTERFD3	70.596.404	70.602.003	5
BTBD11	70.923.455	71.257.874	5

TMEM263	70.573.523	70.593.134	5
PWP1	71.280.814	71.300.028	5
RTCB	71.366.682	71.388.046	5
BPIFC	71.391.549	71.435.614	5
FBXO7	71.448.943	71.469.171	5
SYN3	71.475.668	71.926.718	5
CSF2RB	75.724.619	75.745.819	5
EIF3D	75.299.354	75.313.533	5
FOXRED2	75.279.134	75.292.528	5
NCF4	75.641.216	75.659.771	5
CACNG2	75.343.290	75.464.712	5
PVALB	75.550.902	75.568.048	5
IFT27_AA	75.509.445	75.532.844	5
TEX33	75.790.908	75.808.769	5
MPST_AA	75.817.921	75.827.377	5
MPST	75.817.996	75.827.377	5
TST	75.810.520	75.817.101	5
KCTD17	75.855.302	75.866.757	5
C1QTNF6	75.992.617	75.999.893	5
RAC2	76.033.362	76.050.188	5
CYTH4	76.080.337	76.109.479	5
MIR1835	76.049.714	76.049.777	5
MFNG	76.266.276	76.282.792	5
USP18	76.388.592	76.404.941	5
ALG10	76.497.225	76.503.464	5
SYT10	76.692.597	76.808.969	5
RPL34	17.827.883	17.831.767	6
OSTC	17.789.595	17.801.277	6
LEF1	18.335.030	18.450.774	6
HADH	18.459.378	18.502.809	6
CYP2U1	18.512.362	18.533.033	6
SGMS2	18.547.605	18.568.780	6
ATOH1	32.136.523	32.138.712	6
CCSER1	35.100.698	35.938.394	6
PIGY	37.677.051	37.679.907	6
HERC3	37.478.605	37.624.902	6
HERC6	37.736.137	37.793.279	6
PYURF	37.677.051	37.679.907	6
NAP1L5	37.509.212	37.511.141	6
HERC5	37.683.705	37.728.602	6
FAM13A	37.355.567	37.457.493	6
PPM1K	37.876.469	37.898.525	6
ABCG2	37.959.535	38.030.586	6
PKD2	38.040.870	38.099.549	6
SPP1	38.120.577	38.127.541	6
LAP3	38.574.589	38.600.027	6

IBSP	38.309.554	38.323.303	6
MED28	38.603.033	38.608.841	6
MEPE	38.279.590	38.293.661	6
NCAPG	38.765.968	38.812.055	6
DCAF16_AA	38.754.414	38.755.059	6
LCORL	38.840.863	38.992.112	6
SLIT2	41.236.269	41.642.320	6
MIR218-1	41.545.028	41.545.138	6
PACRGL	41.685.690	41.707.024	6
KCNIP4	41.707.927	41.837.538	6
GBA3	43.730.499	43.863.281	6
NIPAL1	68.364.431	68.391.572	6
NFXL1	68.238.617	68.299.616	6
CNGA1	68.316.336	68.354.288	6
TXK	68.404.659	68.449.203	6
FRYL	68.827.869	68.974.140	6
SLAIN2	68.695.101	68.766.469	6
SLC10A4	68.813.853	68.819.851	6
ZAR1	68.820.786	68.824.912	6
OCIAD1	69.144.343	69.167.282	6
TECRL	81.511.553	81.653.990	6
C7H5orf15	47.231.924	47.244.897	7
VDAC1	47.247.742	47.273.458	7
TCF7	47.352.222	47.384.999	7
SKP1	47.389.341	47.401.412	7
PPP2CA	47.425.979	47.450.747	7
SMAD5	49.155.482	49.217.780	7
FBXL21	48.966.619	48.985.226	7
TGFBI	49.062.891	49.095.978	7
LECT2	48.987.177	48.998.964	7
SLC25A48	48.858.233	48.904.409	7
TRPC7	49.250.060	49.400.471	7
TIMD4	70.297.935	70.348.692	7
HAVCR1	70.213.697	70.224.457	7
CNTLN	26.938.855	27.268.300	8
SH3GL2	26.631.847	26.859.970	8
CHMP7	71.228.580	71.251.803	8
TNFRSF10D	71.111.761	71.212.279	8
TNFRSF10D_AA	70.908.188	71.192.762	8
ENTPD4	71.430.480	71.457.015	8
LOXL2	71.278.581	71.390.893	8
SLC25A37_AA	71.542.980	71.584.026	8
SLC25A37	71.587.510	71.590.578	8
LPPR1	92.346.241	92.674.692	8
TMEM246	92.824.738	92.865.294	8
ZNF189	92.751.542	92.762.917	8

MRPL50	92.744.047	92.751.166	8
ALDOB	92.773.690	92.789.092	8
RNF20	92.911.254	92.935.750	8
GRIN3A	92.944.702	93.145.524	8
PPP3R2	92.968.723	92.969.626	8
TRIM32	107.645.158	107.656.814	8
SH3BGRL2	19.480.888	19.554.836	9
ELOVL4	19.842.900	19.881.703	9
TTK	19.944.418	19.990.959	9
BCKDHB	19.963.213	20.335.161	9
MAP3K7	60.555.708	60.620.134	9
SNX14	64.765.557	64.849.621	9
NT5E	64.859.549	64.871.772	9
NT5E_AA	64.879.263	64.929.945	9
CTGF	70.873.216	70.876.448	9
ENPP1	70.734.236	70.804.299	9
STX7	71.381.756	71.455.585	9
TAAR6	71.504.220	71.505.259	9
RPS12	71.974.859	71.978.201	9
VNN2	71.884.620	71.913.826	9
VNN1	71.833.313	71.851.906	9
TAAR2	71.780.662	71.781.583	9
PDE7B	75.034.592	75.191.800	9
BCLAF1	75.262.827	75.291.455	9
MTFR2	75.236.006	75.254.523	9
MAP7	75.364.389	75.519.232	9
CNKSR3	92.451.952	92.525.551	9
OPRM1	92.152.081	92.209.694	9
C10H15orf41	32.328.991	32.570.684	10
MEIS2	32.643.641	32.868.269	10
TTBK2	38.159.316	38.248.606	10
HAUS2	37.995.375	38.008.617	10
SNAP23	37.946.250	37.986.851	10
LRRC57	37.989.930	37.995.318	10
CAPN3	37.829.006	37.885.645	10
UBR1	38.300.764	38.447.565	10
CCDC175	72.027.459	72.101.711	10
JKAMP	72.005.614	72.027.624	10
L3HYPDH	71.992.842	72.005.431	10
DHRS7	72.617.790	72.641.194	10
KRT8	73.627.763	73.629.541	10
MNAT1	73.153.516	73.363.532	10
TRMT5	73.369.415	73.380.029	10
PRKCH	73.694.667	73.927.122	10
MAP3K9	82.728.772	82.806.769	10
LIMS1	44.577.632	44.658.623	11

RANBP2	44.509.054	44.570.848	11
SEPT10	44.150.750	44.188.823	11
GCC2	44.669.019	44.702.360	11
SULT1C4_AA	44.724.158	44.731.845	11
SULT1C3	44.768.841	44.785.035	11
ATOH8	48.954.368	48.996.567	11
PTCD3	48.598.059	48.629.215	11
REEP1	48.389.167	48.525.011	11
MRPL35	48.529.513	48.535.995	11
ST3GAL5	48.848.623	48.905.318	11
POLR1A	48.629.651	48.713.876	11
IMMT	48.544.000	48.586.544	11
GNLY	49.060.225	49.062.860	11
LGALSL	62.812.718	62.820.736	11
SLC1A4	63.337.654	63.367.593	11
ACTR2	63.552.927	63.591.981	11
RAB1A	63.429.722	63.458.565	11
MEIS1	64.770.130	64.915.360	11
CNRIP1	66.679.941	66.704.192	11
PNO1	66.566.346	66.575.632	11
WDR92	66.536.902	66.565.908	11
FBXO48	66.837.189	66.838.229	11
PLEK	66.762.700	66.788.487	11
APLF	66.840.543	66.933.745	11
PPP3R1	66.582.635	66.642.276	11
PROKR1	66.986.759	66.998.537	11
ARHGAP25	67.066.106	67.158.251	11
BMP10	67.187.148	67.193.866	11
GKN1	67.283.596	67.289.445	11
ANTXR1	67.334.563	67.590.531	11
ITGB1BP1	87.994.385	88.010.373	11
CPSF3	87.960.692	87.994.179	11
YWHAQ	87.842.279	87.873.675	11
IAH1	87.942.469	87.953.717	11
MIR2298	88.328.995	88.329.064	11
RALGPS1	97.674.641	97.975.506	11
ZBTB43	97.582.041	97.608.142	11
ANGPTL2	97.836.903	97.859.547	11
GARNL3	98.028.248	98.138.113	11
DNAJC15	13.183.733	13.266.310	12
COG6	22.718.819	22.773.621	12
LHFP	22.817.677	23.026.883	12
MIR2285AB	51.127.955	51.128.014	12
MYCBP2	52.496.958	52.769.208	12
CLN5	52.453.835	52.461.657	12
FBXL3	52.466.036	52.477.380	12

SCEL	53.032.462	53.156.625	12
MIR2284S	53.250.954	53.251.022	12
SRXN1	60.944.182	60.951.049	13
ANGPT4	60.729.757	60.773.779	13
SLC52A3	60.829.862	60.845.763	13
FAM110A	60.798.591	60.812.321	13
PSMF1	60.505.461	60.544.959	13
TCF15	60.991.999	60.997.980	13
CSNK2A1	61.049.592	61.107.276	13
TBC1D20	61.123.466	61.142.448	13
RBCK1	61.147.246	61.164.402	13
NRSN2	61.211.660	61.218.035	13
TRIB3	61.176.217	61.187.210	13
ZCCHC3	61.254.415	61.257.121	13
DEFB119	61.523.657	61.533.444	13
DEFB122	61.572.836	61.577.455	13
DEFB122A	61.562.052	61.566.096	13
DEFB119_AA	61.531.963	61.533.379	13
TM9SF4	62.165.597	62.218.185	13
KIF3B	62.290.757	62.331.054	13
POFUT1	62.237.056	62.258.544	13
COMMD7	62.647.730	62.675.887	13
POFUT1_AA	62.237.056	62.258.544	13
DNMT3B	62.700.410	62.721.124	13
MAPRE1	62.728.052	62.755.964	13
SUN5	62.822.736	62.843.246	13
BPIFB2	62.850.751	62.869.092	13
BPIFB6	62.877.502	62.892.488	13
BPIFB3	62.901.451	62.918.251	13
CHMP4B	63.817.039	63.849.783	13
E2F1	63.704.816	63.714.008	13
ZNF341	63.750.179	63.790.156	13
SNTA1	63.408.785	63.490.256	13
RALY	63.953.595	64.043.571	13
PXMP4	63.726.541	63.743.492	13
NECAB3	63.684.234	63.703.230	13
EIF2S2	64.050.710	64.069.300	13
ASIP	64.213.311	64.239.962	13
AHCY	64.258.557	64.274.274	13
ITCH	64.357.861	64.462.220	13
MAP1LC3A	64.497.253	64.498.905	13
DYNLRB1	64.466.197	64.483.930	13
ACSS2	64.794.727	64.841.520	13
GGT7	64.737.633	64.791.116	13
GSS	64.842.137	64.867.355	13
MIR499	64.900.479	64.900.582	13

TRPC4AP	64.912.845	64.981.566	13
EIF6	65.206.236	65.212.123	13
EDEM2	64.999.240	65.028.233	13
UQCC	65.233.031	65.327.796	13
PROCR	65.052.809	65.106.553	13
GDF5	65.340.131	65.343.889	13
ERGIC3	65.435.598	65.448.951	13
CPNE1	65.507.238	65.543.149	13
SPAG4	65.499.317	65.504.023	13
NFS1	65.545.939	65.563.035	13
ROMO1	65.563.082	65.564.614	13
RBM12	65.532.003	65.543.175	13
RBM39	65.566.348	65.592.976	13
PHF20	65.616.356	65.741.829	13
SCAND1	65.744.267	65.745.100	13
AAR2	66.018.135	66.038.296	13
DLGAP4	66.263.334	66.292.990	13
MYL9	66.306.259	66.314.230	13
C13H20orf24	66.362.561	66.369.979	13
TGIF2	66.337.750	66.352.681	13
NDRG3	66.398.145	66.594.149	13
SLA2	66.368.688	66.395.515	13
SAMHD1	66.432.395	66.534.338	13
TLDC2	66.433.852	66.442.685	13
RBL1	66.567.168	66.730.589	13
DSN1	66.602.328	66.618.267	13
RPN2	66.799.464	66.853.781	13
MANBAL	66.899.245	66.927.386	13
GHRH	66.863.224	66.872.531	13
SRC	66.985.877	67.004.791	13
NNAT_AA	67.118.489	67.120.881	13
NNAT	67.118.489	67.120.881	13
BLCAP	67.114.711	67.125.197	13
CTNNBL1	67.257.418	67.431.112	13
VSTM2L	67.460.323	67.498.849	13
TTI1	67.528.733	67.559.052	13
RPRD1B	67.570.197	67.628.071	13
TGM2	67.663.134	67.697.607	13
BPI	67.835.163	67.861.379	13
LBP	67.875.397	67.910.095	13
ADIG	68.119.516	68.129.193	13
PPP1R16B	68.258.626	68.366.080	13
FAM83D	68.368.928	68.391.902	13
LRRC24	1.604.104	1.609.477	14
FOXH1	1.654.700	1.656.256	14
VPS28	1.693.642	1.698.621	14

CPSF1	1.728.206	1.742.668	14
MROH1	1.844.663	1.894.424	14
HSF1	1.806.080	1.825.793	14
MFSD3	1.620.565	1.622.643	14
MIR1839	1.883.845	1.883.917	14
SCRT1	1.782.900	1.790.463	14
SLC39A4	1.719.731	1.724.221	14
KIFC2	1.656.360	1.663.803	14
C14H8orf33	1.487.365	1.489.409	14
LRRC14	1.610.261	1.613.725	14
CYHR1_AA	1.664.422	1.665.518	14
DGAT1	1.795.424	1.804.838	14
RECQL4	1.614.026	1.620.509	14
ARHGAP39	1.563.865	1.600.378	14
PPP1R16A	1.628.813	1.633.988	14
CYHR1	1.674.620	1.677.519	14
MIR2308	1.566.932	1.567.001	14
RPL8	1.505.029	1.507.626	14
COMMD5	1.531.490	1.533.526	14
C14H8orf82	1.602.473	1.604.999	14
ADCK5	1.742.710	1.756.301	14
TONSL	1.681.493	1.692.499	14
ZNF34	1.491.980	1.497.904	14
GPR172A	1.763.992	1.766.621	14
GPT	1.623.901	1.626.907	14
MAF1	1.921.782	1.924.816	14
SHARPIN	1.925.025	1.929.354	14
GPAA1	1.942.671	1.945.910	14
CYC1	1.930.182	1.932.580	14
GRINA	2.018.558	2.021.709	14
OPLAH	1.957.553	1.966.647	14
EXOSC4	1.947.197	1.949.074	14
PUF60	2.164.998	2.177.242	14
NRBP2	2.152.611	2.159.658	14
MIR2309	2.071.850	2.071.925	14
MAPK15	2.235.032	2.240.765	14
PYCRL	2.301.586	2.309.099	14
TSTA3	2.288.554	2.293.395	14
CCDC166	2.253.176	2.254.950	14
MIR193A-2	2.272.016	2.272.069	14
GSDMD	2.341.281	2.347.798	14
NAPRT1	2.327.869	2.331.019	14
EEF1D	2.313.945	2.326.721	14
EEF1D_AA	2.313.945	2.326.721	14
ANXA13	17.734.174	17.750.055	14
TMEM65	17.254.350	17.299.175	14

FBXO32	17.919.804	29.162.110	14
KIAA1429	72.100.483	72.164.631	14
RAD54B	72.176.934	72.295.285	14
ESRP1	71.945.028	72.003.342	14
GEM	72.386.750	72.399.661	14
GEM_AA	72.385.635	72.399.661	14
RUNX1T1	74.642.805	74.733.199	14
UPK2	29.207.862	30.075.285	15
SCN2B	29.291.351	29.303.255	15
MPZL2	29.378.621	29.389.165	15
MPZL3	29.345.978	29.376.614	15
SCN4B	29.257.443	29.277.309	15
AMICA1	29.313.739	29.344.913	15
IL10RA	29.090.960	29.102.604	15
FXYD6	28.926.758	28.963.808	15
FXYD2	28.909.314	28.916.825	15
TMPRSS4	29.193.490	29.233.496	15
CD3E	29.441.566	29.452.181	15
UBE4A	29.476.821	29.516.720	15
CD3D	29.456.445	29.460.605	15
CD3G	29.462.565	29.474.058	15
ATP5L	29.521.981	29.530.113	15
LOC784741	29.622.642	29.623.547	15
RIC3	44.962.851	45.024.903	15
TUB	45.033.762	45.055.126	15
LMO1	44.880.075	44.917.825	15
LOC783210	45.393.385	45.394.330	15
LOC506989	46.902.642	46.904.086	15
MIR2316	47.272.309	47.272.384	15
RRP8	47.082.235	47.085.798	15
FXC1	47.185.420	47.186.320	15
TRIM3	47.193.827	47.219.562	15
ILK	47.074.655	47.082.065	15
SMPD1	47.267.700	47.272.165	15
MRPL17	47.004.018	47.005.494	15
ARFIP2	47.186.493	47.191.611	15
APBB1	47.245.397	47.267.568	15
TAF10	47.073.271	47.074.687	15
HPX	47.226.086	47.235.666	15
TPP1	47.065.386	47.070.704	15
PRKCDBP	47.329.715	47.331.336	15
CCKBR	47.384.223	47.395.047	15
HBE4	49.007.000	49.008.555	15
HBE1	48.997.630	48.999.487	15
HBE2	49.049.051	49.050.463	15
HBG	49.073.120	49.074.735	15

HBB	49.022.977	49.024.620	15
LOC788610	49.073.059	49.074.738	15
LOC785144	49.922.807	49.923.752	15
LOC788703	50.649.457	50.650.392	15
UVRAG	55.992.136	56.317.353	15
MARK1	55.850.536	55.853.227	15
MOGAT2	55.892.937	55.904.776	15
DGAT2	55.940.756	55.973.229	15
MOGAT2_AA	55.810.203	55.816.942	15
WNT11	56.384.699	56.404.335	15
ACBD3	29.911.423	29.949.907	16
C16H1orf95	30.304.539	30.360.118	16
PARP1	30.119.201	30.160.574	16
FASLG	41.027.787	41.036.682	16
TNFSF18	41.541.494	41.552.708	16
TNFSF4	41.689.562	41.709.076	16
DHRS3_AA	42.039.113	42.040.601	16
VPS13D	42.079.661	42.349.474	16
DHRS3	41.984.484	42.002.572	16
TNFRSF1B	42.376.250	42.409.768	16
MFN2_AA	42.560.146	42.587.221	16
MFN2	42.560.146	42.587.221	16
MIIP	42.537.918	42.548.110	16
PLOD1	42.594.655	42.625.798	16
AJAP1	49.832.131	49.912.373	16
C16H1orf174	50.472.097	50.478.063	16
DFFB	50.480.285	50.494.637	16
MIR2320	50.502.446	50.502.521	16
LRRC47	50.540.188	50.551.323	16
SMIM1	50.553.081	50.556.851	16
WRAP73	50.674.471	50.686.445	16
TPRG1L	50.687.997	50.690.792	16
MIR551A	50.741.092	50.741.186	16
ZBTB37	56.696.965	56.706.787	16
RABGAP1L	56.941.659	57.039.136	16
SERPINC1	56.723.666	56.734.280	16
GPR52	57.209.959	57.214.300	16
MSI1	64.914.953	64.937.019	17
ADAM1B	64.491.185	64.494.524	17
SIRT4	64.875.356	64.891.607	17
ALDH2	64.551.610	64.577.901	17
MAPKAPK5	64.505.160	64.538.589	17
RPLP0	64.809.253	64.813.277	17
TMEM116	64.443.982	64.462.559	17
PLA2G1B	64.899.558	64.902.628	17
RAB35	64.724.241	64.742.928	17

PLA2G1B_AA	64.910.438	64.913.109	17
COX6A1	64.995.247	64.997.122	17
GATC	65.000.549	65.007.059	17
TRIAP1	64.998.264	65.000.579	17
SRSF9	65.008.116	65.014.401	17
DYNLL1	65.042.595	65.045.026	17
RNF10	65.067.804	65.101.420	17
POP5	65.102.633	65.104.915	17
CABP1	65.154.258	65.170.682	17
MLEC	65.184.867	65.194.880	17
UNC119B	65.206.693	65.216.028	17
ACADS	65.220.604	65.237.484	17
SART3_AA	66.666.990	66.684.125	17
FICD	66.628.035	66.631.575	17
ISCU	66.685.451	66.691.556	17
CORO1C	66.467.520	66.542.217	17
CMKLR1	66.827.050	66.828.139	17
TMEM119	66.586.627	66.593.119	17
SART3	66.608.715	66.625.585	17
SELPLG	66.557.142	66.573.395	17
LOC533308	66.685.503	66.691.252	17
SELPLG_AA	66.557.142	66.573.395	17
MIR6521	66.961.890	66.961.978	17
DRG1	72.273.493	72.288.389	17
OSBP2	71.771.702	71.894.872	17
LIMK2	72.142.467	72.195.910	17
INPP5J	72.076.122	72.086.055	17
RNF185	72.108.602	72.138.746	17
SMTN	72.041.476	72.064.314	17
MORC2	71.920.844	71.941.603	17
PATZ1	72.229.522	72.247.004	17
PIK3IP1	72.198.071	72.208.758	17
SELM	72.064.475	72.068.000	17
PISD	72.425.904	72.453.284	17
SMPD4	74.397.214	74.412.808	17
UFD1L	74.717.682	74.730.989	17
DGCR2	74.581.118	74.598.155	17
CLDN5	74.749.249	74.750.524	17
LOC787548	74.392.828	74.397.072	17
PRODH	74.054.343	74.456.079	17
GNB1L	74.852.180	74.880.572	17
CDC45	74.732.074	74.747.037	17
C17H22orf39	74.711.449	74.715.833	17
KLHL22	74.506.170	74.520.231	17
TSSK1B	74.607.592	74.609.032	17
SLC25A1	74.633.531	74.635.904	17

MED15	74.425.041	74.490.537	17
DGCR14	74.613.478	74.620.081	17
SEPT5	74.807.362	74.811.262	17
ZNF74	74.559.800	74.564.762	17
TXNRD2	74.887.065	74.912.977	17
COMT	74.924.017	74.929.409	17
MIR185	74.967.065	74.967.144	17
ARVCF	74.929.356	74.933.307	17
TANGO2	74.969.198	74.976.402	17
MIR1306	74.987.968	74.988.026	17
DGCR8	74.987.853	75.002.266	17
TANGO2_AA	74.981.685	74.983.351	17
RANBP1	75.007.339	75.009.962	17
RANBP1_AA	75.037.054	75.038.117	17
UQCRFS1	1.021.247	1.026.029	18
VAC14	1.449.429	1.525.768	18
CYBA	13.931.106	13.938.043	18
CTU2	13.977.384	13.984.731	18
TRAPPC2L	14.079.803	14.083.636	18
APRT	14.057.696	14.060.472	18
GALNS	14.061.155	14.077.096	18
RNF166	13.969.300	13.977.633	18
MIR2327	14.003.562	14.003.632	18
MVD	13.938.825	13.945.489	18
ACSF3	14.254.105	14.292.739	18
SNAI3	13.958.994	13.964.622	18
CHD9	21.634.591	21.859.834	18
FTO	22.118.200	22.541.539	18
RBL2	21.879.670	21.925.831	18
AKTIP	21.925.485	21.935.039	18
CDH1	36.221.700	36.243.315	18
CDH3	36.095.672	36.140.923	18
HAS3	36.449.382	36.455.306	18
CHTF8	36.458.138	36.469.860	18
CIRH1A	36.469.930	36.500.064	18
ETFB	57.795.755	57.808.323	18
NKG7	57.812.521	57.815.252	18
SIGLEC10	57.835.265	57.842.961	18
LIM2	57.819.478	57.826.623	18
CLDND2	57.809.112	57.810.395	18
IGLON5	57.762.990	57.777.429	18
CTU1	57.517.495	57.524.958	18
LOC100138951	57.574.116	57.583.637	18
MIR99B	58.014.867	58.014.937	18
MIRLET7E	58.015.035	58.015.114	18
MIR125A	58.015.534	58.015.620	18

ZNF613	58.100.733	58.141.898	18
CUEDC2	27.388.015	27.389.051	19
ASGR1	27.529.096	27.532.634	19
ASGR2	27.490.116	27.500.094	19
PLSCR3	27.706.378	27.710.913	19
MIR2338	27.298.366	27.298.439	19
TMEM102	27.747.546	27.749.622	19
ACADVL	27.568.222	27.573.378	19
RNASEK	27.436.502	27.438.228	19
TMEM256	27.714.468	27.715.619	19
CLDN7	27.602.592	27.604.817	19
DVL2	27.573.454	27.581.405	19
KCTD11	27.685.378	27.688.137	19
YBX2	27.622.772	27.628.768	19
PHF23	27.582.169	27.585.939	19
NLGN2	27.727.931	27.733.393	19
ZBTB4	27.767.023	27.773.342	19
FGF11	27.751.728	27.755.716	19
C19H17orf49	27.438.489	27.441.265	19
GPS2	27.653.443	27.656.067	19
MIR195	27.441.342	27.441.429	19
DLG4	27.543.282	27.565.410	19
ALOX12E	27.361.426	27.370.258	19
MIR497	27.441.651	27.441.763	19
SLC16A13	27.455.596	27.459.089	19
SLC2A4	27.616.612	27.622.206	19
NEURL4	27.656.327	27.668.154	19
ALOX15	27.331.076	27.339.653	19
ALOX12	27.421.124	27.434.258	19
EIF5A	27.648.381	27.653.258	19
CTDNEP1	27.589.892	27.596.031	19
SPEM1	27.733.849	27.735.192	19
MIR324	27.571.340	27.571.429	19
GABARAP	27.586.883	27.588.815	19
CHRNB1	27.757.182	27.766.382	19
ACAP1	27.673.003	27.684.649	19
BCL6B	27.447.121	27.451.108	19
POLR2A	27.786.958	27.815.341	19
AMAC1L3	27.784.051	27.785.626	19
TNFSF12	27.893.174	27.901.018	19
TNFSF12_AA	27.893.254	27.900.538	19
EIF4A1	27.915.412	27.921.421	19
CD68	27.921.926	27.923.976	19
SENP3	27.906.585	27.914.530	19
TNFSF13	27.901.944	27.904.808	19
MPDU1	27.925.700	27.929.096	19

SAT2	27.953.737	27.955.396	19
FXR2	27.932.222	27.940.210	19
SOX15	27.929.103	27.930.911	19
SHBG	27.955.572	27.960.099	19
WRAP53	27.998.929	28.010.745	19
ATP1B2	27.974.166	27.978.470	19
TP53	27.985.493	27.997.841	19
EFNB3	28.012.503	28.018.573	19
KCNAB3	28.214.103	28.220.774	19
TMEM88	28.156.791	28.157.771	19
CYB5D1	28.159.799	28.161.241	19
DNAH2	28.027.091	28.134.582	19
TRAPPC1	28.221.788	28.223.283	19
ALOX12B	28.329.830	28.341.331	19
GUCY2D	28.274.098	28.288.581	19
ALOX15B	28.306.790	28.315.605	19
HES7	28.375.016	28.377.490	19
PER1	28.390.015	28.399.748	19
C19H17orf59	28.443.495	28.445.326	19
TMEM107	28.432.874	28.435.112	19
VAMP2_AA	28.408.574	28.410.802	19
AURKB	28.447.632	28.452.478	19
SLC25A35	28.521.122	28.524.591	19
ARHGEF15	28.541.732	28.550.394	19
RANGRF	28.519.718	28.521.136	19
PFAS	28.498.628	28.511.705	19
ODF4	28.565.346	28.570.383	19
RPL26	28.590.311	28.595.225	19
GAS7	29.670.740	29.876.650	19
RCVRN	29.653.155	29.661.163	19
MYH2	30.137.766	30.165.109	19
MYH1	30.110.727	30.134.757	19
MYH8	30.031.026	30.061.053	19
CBX8	53.360.163	53.362.249	19
SLC26A11	53.023.435	53.041.077	19
SGSH	53.041.241	53.048.385	19
EIF4A3	53.085.462	53.097.208	19
CBX2	53.371.116	53.377.419	19
GAA	53.100.964	53.113.264	19
TBC1D16	53.181.378	53.246.057	19
CARD14	53.048.428	53.069.156	19
RBFOX3	53.623.086	53.953.039	19
MAP1B	9.325.945	9.419.062	20
PTCD2	9.179.366	9.211.163	20
ZNF366	9.075.830	9.094.324	20
MRPS27	9.211.236	9.315.275	20

DAB2	35.018.907	35.079.162	20
C1QTNF3	39.760.049	39.792.325	20
ADAMTS12	39.913.084	40.304.028	20
CDH12	51.282.706	51.624.351	20
LPCAT1	71.144.852	71.167.619	20
NDUFS6	70.986.111	70.991.386	20
CLPTM1L	71.235.490	71.247.878	20
TERT	71.263.286	71.279.911	20
IRX4	70.929.127	70.933.738	20
SLC6A18	71.293.256	71.299.686	20
UBE3A	2.346.808	2.410.193	21
FAM103A1	23.705.959	23.711.316	21
C21H15orf40	23.721.982	23.728.268	21
HMG20A	33.083.548	33.160.048	21
CSPG4	33.574.739	33.610.470	21
IMP3	33.646.396	33.647.527	21
SNX33	33.625.398	33.633.910	21
ODF3L1	33.559.733	33.563.692	21
PTPN9	33.697.443	33.766.710	21
SNUPN	33.666.093	33.683.248	21
SEMA7A	34.626.180	34.651.483	21
STRA6	34.878.676	34.901.785	21
ISLR	34.903.758	34.906.883	21
CYP11A1_AA	34.724.305	34.734.990	21
ARID3B	34.476.932	34.523.170	21
UBL7	34.601.364	34.614.703	21
PML	34.981.852	35.025.090	21
STOML1	35.027.785	35.036.053	21
GZMB	35.135.770	35.137.852	21
LOXL1	35.058.002	35.081.938	21
STXBP6	35.415.157	35.655.955	21
GADL1	5.258.461	5.452.369	22
CAV3	17.832.936	17.846.997	22
OXTR	17.817.625	17.827.292	22
SRGAP3	17.372.629	17.613.438	22
LMCD1	17.961.209	18.018.234	22
RAD18	17.627.572	17.735.889	22
CHL1	26.101.270	26.322.519	22
ADAMTS9	36.876.903	37.038.527	22
PSMD6	37.538.100	37.556.710	22
FAM107A	43.354.327	43.359.047	22
ACOX2	43.379.503	43.410.316	22
KCTD6	43.413.161	43.416.190	22
PDHB	43.461.213	43.466.627	22
PXK	43.466.981	43.548.273	22
MIR2370	43.741.295	43.741.374	22

ABHD6	43.569.313	43.617.625	22
DNASE1L3	43.639.143	43.659.285	22
FLNB	43.674.719	43.815.706	22
ARF4	44.150.603	44.169.288	22
NEK4	48.680.449	48.708.295	22
STAB1	48.884.237	48.909.562	22
GLT8D1	48.712.945	48.725.193	22
SFMBT1	48.499.293	48.548.478	22
TMEM110	48.556.151	48.607.238	22
MUSTN1	48.611.897	48.613.044	22
SPCS1	48.710.802	48.712.454	22
ITIH1	48.655.026	48.669.271	22
ITIH3	48.633.829	48.647.777	22
ITIH4	48.615.396	48.630.388	22
TNNC1	48.988.983	48.991.878	22
BAP1	49.032.897	49.041.534	22
GLYCTK	49.142.817	49.146.185	22
MIR135A-1	49.142.027	49.142.117	22
ALAS1	49.242.703	49.256.766	22
TWF2	49.218.178	49.227.368	22
POC1A	49.282.959	49.359.379	22
TLR9	49.229.609	49.233.939	22
DUSP7	49.379.909	49.386.856	22
WDR82	49.182.444	49.201.057	22
MIRLET7G	49.189.339	49.189.422	22
RPL29	49.499.797	49.501.973	22
ABHD14B	49.516.823	49.520.470	22
PCBP4	49.521.551	49.531.508	22
ACY1	49.502.957	49.507.814	22
LOC100125949	49.603.515	49.607.502	22
RRP9	49.544.957	49.552.848	22
GPR62	49.531.461	49.533.582	22
IQCF5	49.566.541	49.568.436	22
IQCF2	49.579.350	49.580.862	22
IQCF1	49.557.991	49.560.690	22
MLN	7.865.123	7.872.669	23
LEMD2	7.841.999	7.858.371	23
MNF1	7.758.139	7.783.973	23
UQCC2	7.796.306	7.820.842	23
LOC516410	7.662.063	7.667.198	23
SYNGAP1	7.537.951	7.567.019	23
BAK1	7.655.891	7.658.885	23
ITPR3	7.687.260	7.756.803	23
ZBTB9	7.568.875	7.571.700	23
LOC100126544	8.269.051	8.271.772	23
NUDT3	8.297.775	8.414.740	23

HMGA1	8.258.749	8.268.920	23
RPS10	8.434.568	8.441.520	23
PACSIN1	8.469.768	8.534.471	23
SPDEF	8.537.366	8.554.696	23
C23H6orf106	8.591.961	8.688.924	23
SNRPC	8.726.520	8.737.237	23
CLPS	9.723.790	9.726.250	23
SLC26A8	9.853.164	9.942.504	23
MAPK14	9.944.806	10.023.622	23
LHFPL5	9.733.330	9.742.174	23
FKBP5	9.521.253	9.637.802	23
MAPK13	10.036.686	10.045.047	23
BRPF3	10.099.946	10.132.302	23
PNPLA1	10.166.998	10.223.697	23
KCTD20	10.355.457	10.391.460	23
STK38	10.395.673	10.436.810	23
SRSF3	10.481.199	10.487.676	23
RPS4Y1	10.528.436	10.544.184	23
CPNE5	10.631.429	10.734.955	23
CDKN1A	10.560.498	10.568.782	23
PPIL1	10.748.953	10.769.296	23
PI16	10.836.789	10.848.749	23
MTCH1	10.854.190	10.873.802	23
FGD2	10.892.082	10.918.349	23
PIM1	11.050.889	11.055.827	23
TMEM217	11.109.500	11.132.859	23
ZFAND3	11.712.801	12.040.964	23
CMTR1	11.303.505	11.356.262	23
MDGA1	11.530.877	11.594.184	23
CCDC167	11.358.156	11.373.691	23
BTBD9	12.046.516	12.462.083	23
GLO1	12.483.467	12.509.232	23
MIR2377	13.082.432	13.082.501	23
KCNK5	13.055.369	13.095.621	23
SAYSD1	12.964.405	12.971.106	23
KCNK17	13.160.207	13.172.869	23
SLC17A2	31.721.013	31.739.272	23
SLC17A3	31.741.803	31.778.649	23
LRRC16A	32.029.121	32.339.087	23
TRIM38	31.693.731	31.708.621	23
SCGN	31.951.990	32.004.354	23
DLGAP1	37.994.545	38.293.883	24
SMAD2	47.971.044	48.022.060	24
MIR2390	40.643.968	40.644.040	25
CARD11	40.960.557	41.068.748	25
GNA12	41.099.258	41.171.209	25

MBL2	6.344.784	6.348.912	26
SGMS1	8.734.534	9.058.815	26
MINPP1	9.170.250	9.206.942	26
PAPSS2	9.292.115	9.402.158	26
ATAD1	9.410.908	9.451.790	26
RNLS	9.932.076	10.211.164	26
LOXL4	19.201.156	19.223.480	26
MIR1287	19.342.068	19.342.159	26
LOXL4_AA	19.219.703	19.222.115	26
PYROXD2	19.334.007	19.359.393	26
CRTAC1	18.882.721	19.035.009	26
CRTAC1_AA	18.882.155	19.035.009	26
HPS1	19.361.241	19.386.569	26
CXCL12	45.410.675	45.418.794	28

outlier
3.197.378
70.974.318
70.974.318
70.974.318
70.974.318
70.974.318
71.070.958
71.229.187
71.229.187
71.229.187
71.263.427
71.349.186
71.431.023
71.431.023
71.504.767
71.504.767
71.544.028
94.367.295
126.815.881
126.815.881
126.815.881
126.898.336
127.112.983
127.112.983
137.567.928
137.718.422
137.718.422
137.821.564
137.821.564
4.921.527
4.921.527
4.921.527
4.921.527
4.958.110
5.193.223
5.193.223
5.370.008
5.640.288
5.802.738
6.047.203
6.151.365
6.645.590
6.645.590
6.645.590
6.645.590

6.645.590
7.046.478
7.086.105
8.000.159
8.538.934
8.736.092
9.311.143
9.392.865
9.427.890
9.454.144
9.633.324
47.084.459
47.238.139
59.614.350
61.822.538
61.822.538
61.822.538
61.822.538
61.822.538
61.822.538
62.265.739
49.335.664
49.335.664
49.335.664
49.335.664
49.531.950
49.531.950
49.551.407
49.604.937
53.598.182
53.598.182
53.598.182
53.598.182
55.074.485
55.074.485
55.074.485
58.434.544
75.826.743
84.966.080
88.206.911
88.206.911
27.604.132
34.059.453
67.222.038
67.222.038
92.235.197

92.235.197
92.235.197
92.394.150
92.394.150
92.394.150
92.436.001
94.427.889
94.427.889
94.427.889
94.427.889
94.427.889
94.427.889
94.427.889
94.569.814
114.540.598
114.540.598
114.540.598
114.540.598
114.540.598
114.540.598
114.540.598
114.540.598
114.540.598
114.540.598
114.540.598
114.540.598
114.540.598
114.540.598
114.540.598
114.565.961
115.713.661
26.507.844
26.507.844
26.507.844
26.507.844
26.507.844
26.507.844
26.507.844
26.507.844
26.507.844
26.526.934
26.661.043
26.661.043
26.661.043
26.661.043
26.661.043

26.876.852
26.876.852
26.876.852
26.876.852
26.876.852
26.876.852
26.876.852
26.986.116
26.986.116
27.086.960
27.086.960
27.086.960
27.214.378
27.214.378
27.287.454
27.287.454
46.497.410
63.226.065
63.226.065
63.226.065
63.226.065
63.226.065
67.902.804
67.902.804
67.902.804
67.902.804
67.902.804
67.902.804
67.902.804
67.902.804
67.948.150
68.039.989
68.039.989
68.220.538
68.461.765
68.632.534
68.632.534
68.880.383
68.946.279
69.571.158
69.935.577
69.935.577
70.075.232
70.762.660
70.762.660
70.762.660

70.762.660
71.045.870
71.144.630
71.144.630
71.272.297
71.272.297
75.518.275
75.518.275
75.518.275
75.518.275
75.518.275
75.518.275
75.518.275
75.548.821
75.599.879
75.599.879
75.599.879
75.627.333
75.757.551
75.933.509
75.933.509
75.933.509
76.019.053
76.157.488
76.286.670
76.457.030
18.033.876
18.033.876
18.168.879
18.213.934
18.286.930
18.307.093
32.241.952
35.147.153
37.501.365
37.501.365
37.501.365
37.501.365
37.501.365
37.501.365
37.501.365
37.653.391
37.742.740
37.801.349
37.925.393
38.464.203

38.464.203
38.464.203
38.464.203
38.576.012
38.576.012
38.627.070
41.253.845
41.316.161
41.443.081
41.474.060
43.872.606
68.291.351
68.291.351
68.291.351
68.291.351
68.808.196
68.808.196
68.808.196
68.808.196
68.904.846
81.767.374
47.002.161
47.002.161
47.136.122
47.162.335
47.192.742
48.947.232
48.947.232
48.947.232
48.947.232
48.947.232
49.084.339
70.453.894
70.453.894
27.067.009
27.067.009
71.263.375
71.263.375
71.263.375
71.263.375
71.263.375
71.293.524
71.352.779
92.627.675
92.627.675
92.627.675

92.627.675
92.627.675
92.700.263
92.700.263
92.792.703
107.707.691
19.649.248
19.649.248
19.702.063
19.774.304
60.465.587
64.529.140
64.690.186
64.690.186
70.959.203
70.959.203
71.162.153
71.389.287
71.736.363
71.736.363
71.736.363
71.736.363
74.848.962
75.080.790
75.080.790
75.157.517
92.350.052
92.350.052
32.260.224
32.930.297
38.048.013
38.048.013
38.048.013
38.048.013
38.048.013
38.097.257
72.202.330
72.202.330
72.202.330
72.380.552
73.417.064
73.417.064
73.417.064
73.466.092
83.050.810
44.335.688

44.335.688
44.335.688
44.439.698
44.478.837
44.531.959
48.739.496
48.739.496
48.739.496
48.739.496
48.739.496
48.739.496
48.739.496
48.827.893
62.847.615
63.114.545
63.337.021
63.337.021
64.537.797
66.789.317
66.789.317
66.789.317
66.789.317
66.789.317
66.789.317
66.789.317
66.789.317
66.864.873
66.946.746
67.059.602
67.108.521
87.972.079
87.972.079
87.972.079
87.972.079
88.096.474
97.556.999
97.556.999
97.600.391
97.789.423
13.400.067
22.664.255
22.664.255
51.337.744
52.663.318
52.663.318
52.663.318

52.828.641
53.009.331
60.714.542
60.714.542
60.714.542
60.714.542
60.714.542
60.756.573
60.834.492
60.896.947
61.065.155
61.065.155
61.065.155
61.065.155
61.279.037
61.329.689
61.329.689
61.329.689
62.404.218
62.404.218
62.404.218
62.404.218
62.404.218
62.534.909
62.534.909
62.586.212
62.618.708
62.643.096
62.692.209
63.715.266
63.715.266
63.715.266
63.715.266
63.715.266
63.715.266
63.715.266
63.836.808
63.978.193
64.026.359
64.399.733
64.399.733
64.399.733
64.660.314
64.660.314
64.660.314
64.660.314

64.737.978
65.006.713
65.006.713
65.006.713
65.006.713
65.108.908
65.201.054
65.259.649
65.259.649
65.324.047
65.324.047
65.324.047
65.324.047
65.369.103
65.661.023
65.817.864
66.049.830
66.080.035
66.118.164
66.118.164
66.179.152
66.179.152
66.240.806
66.240.806
66.336.246
66.370.867
66.572.178
66.727.403
66.727.403
66.774.859
66.892.611
66.892.611
66.892.611
67.015.369
67.212.324
67.375.037
67.375.037
67.467.905
67.616.199
67.663.982
67.888.763
68.022.725
68.133.617
1.651.311
1.651.311
1.651.311

1.651.311
1.651.311
1.651.311
1.651.311
1.651.311
1.651.311
1.651.311
1.651.311
1.651.311
1.651.311
1.651.311
1.651.311
1.651.311
1.651.311
1.651.311
1.651.311
1.651.311
1.651.311
1.651.311
1.651.311
1.651.311
1.651.311
1.651.311
1.651.311
1.651.311
1.675.278
1.675.278
1.696.470
1.696.470
1.801.116
1.801.116
1.801.116
1.923.292
1.923.292
1.923.292
2.002.873
2.054.457
2.054.457
2.054.457
2.054.457
2.117.455
2.117.455
2.117.455
2.117.455
17.544.926
17.544.926

17.674.401
72.166.145
72.166.145
72.166.145
72.166.145
72.166.145
74.414.864
29.162.110
29.162.110
29.162.110
29.162.110
29.162.110
29.162.110
29.162.110
29.162.110
29.162.110
29.162.110
29.206.191
29.233.683
29.233.683
29.233.683
29.305.818
29.392.924
45.104.876
45.104.876
45.104.876
45.241.208
47.041.396
47.041.396
47.041.396
47.041.396
47.041.396
47.041.396
47.041.396
47.041.396
47.041.396
47.041.396
47.041.396
47.041.396
47.041.396
47.085.423
47.135.215
49.031.887
49.031.887
49.031.887
49.031.887

49.031.887
49.031.887
49.987.359
50.485.308
55.832.867
55.832.867
55.832.867
55.832.867
55.832.867
56.135.629
30.137.377
30.137.377
30.137.377
41.283.716
41.345.308
41.664.706
41.992.472
41.992.472
41.992.472
42.262.470
42.386.711
42.386.711
42.386.711
42.386.711
50.095.343
50.256.549
50.256.549
50.256.549
50.302.273
50.383.731
50.458.174
50.458.174
50.517.509
56.952.563
56.952.563
56.952.563
56.996.623
64.696.267
64.696.267
64.696.267
64.696.267
64.696.267
64.696.267
64.696.267
64.696.267
64.696.267

64.696.267
64.754.159
64.754.159
64.754.159
64.784.269
64.811.174
64.843.236
64.879.624
64.912.981
64.950.742
64.982.245
64.982.245
66.671.839
66.671.839
66.671.839
66.671.839
66.671.839
66.671.839
66.671.839
66.671.839
66.671.839
66.671.839
66.725.091
72.063.677
72.063.677
72.063.677
72.063.677
72.063.677
72.063.677
72.063.677
72.063.677
72.063.677
72.063.677
72.180.821
74.627.267
74.627.267
74.627.267
74.627.267
74.627.267
74.627.267
74.627.267
74.627.267
74.627.267
74.627.267
74.627.267
74.627.267

74.627.267
74.627.267
74.627.267
74.627.267
74.666.629
74.723.634
74.723.634
74.723.634
74.723.634
74.748.408
74.748.408
74.748.408
74.769.219
74.817.054
0.818901
1.212.743
14.038.121
14.038.121
14.038.121
14.038.121
14.038.121
14.038.121
14.038.121
14.038.121
14.038.121
14.038.121
21.869.056
21.869.056
21.869.056
21.869.056
36.146.356
36.146.356
36.207.061
36.250.231
36.250.231
57.745.169
57.745.169
57.745.169
57.745.169
57.745.169
57.745.169
57.745.169
57.745.169
57.768.387
57.768.387
57.768.387

57.855.715
27.529.791
27.529.791
27.529.791
27.529.791
27.529.791
27.529.791
27.529.791
27.529.791
27.529.791
27.529.791
27.529.791
27.529.791
27.529.791
27.529.791
27.529.791
27.529.791
27.529.791
27.529.791
27.529.791
27.529.791
27.529.791
27.529.791
27.529.791
27.529.791
27.529.791
27.529.791
27.529.791
27.529.791
27.529.791
27.529.791
27.529.791
27.529.791
27.529.791
27.529.791
27.529.791
27.529.791
27.576.066
27.576.066
27.643.677
27.643.677
27.674.629
27.674.629
27.674.629
27.674.629
27.706.098

27.706.098
27.706.098
27.706.098
27.706.098
27.763.447
27.763.447
27.763.447
27.763.447
27.965.234
27.965.234
27.965.234
27.965.234
27.992.667
28.095.023
28.095.023
28.095.023
28.155.641
28.155.641
28.204.745
28.204.745
28.204.745
28.204.745
28.299.528
28.299.528
28.299.528
28.299.528
28.342.107
28.342.107
29.890.136
29.890.136
29.890.136
29.890.136
29.890.136
53.208.781
53.208.781
53.208.781
53.208.781
53.208.781
53.208.781
53.208.781
53.208.781
53.373.533
9.104.042
9.104.042
9.104.042
9.104.042

34.777.599
39.727.100
39.727.100
51.044.020
71.067.704
71.067.704
71.067.704
71.067.704
71.067.704
71.067.704
2.151.256
23.923.849
23.923.849
33.401.904
33.401.904
33.401.904
33.401.904
33.401.904
33.475.697
33.475.697
34.730.593
34.730.593
34.730.593
34.730.593
34.730.593
34.730.593
34.766.249
35.003.844
35.003.844
35.003.844
35.184.852
5.579.646
17.857.858
17.857.858
17.857.858
17.857.858
17.857.858
26.002.227
37.013.886
37.337.158
43.127.183
43.175.304
43.175.304
43.218.799
43.218.799
43.862.404

43.862.404
43.862.404
43.862.404
43.914.790
48.663.639
48.663.639
48.663.639
48.663.639
48.663.639
48.663.639
48.663.639
48.663.639
48.663.639
48.663.639
48.764.434
48.810.254
48.904.665
48.904.665
49.131.324
49.131.324
49.131.324
49.131.324
49.131.324
49.131.324
49.131.324
49.251.271
49.273.889
49.273.889
49.273.889
49.402.072
49.402.072
49.402.072
49.402.072
49.402.072
49.402.072
7.802.541
7.802.541
7.802.541
7.802.541
7.802.541
7.802.541
7.802.541
7.802.541
7.802.541
8.063.504
8.063.504

8.063.504
8.289.524
8.289.524
8.289.524
8.347.642
8.513.266
9.712.645
9.712.645
9.712.645
9.712.645
9.712.645
9.790.753
9.861.848
9.951.185
10.115.560
10.146.619
10.240.332
10.306.869
10.391.419
10.391.419
10.526.857
10.593.430
10.631.079
10.665.897
10.823.037
10.870.036
11.563.415
11.563.415
11.563.415
11.563.415
11.818.761
12.519.690
12.838.486
12.838.486
12.838.486
12.997.137
31.946.349
31.946.349
31.946.349
31.946.349
31.946.349
38.383.252
48.164.849
40.406.561
40.730.047
40.851.475

6.437.290
9.078.964
9.078.964
9.078.964
9.195.089
9.698.553
19.189.998
19.189.998
19.189.998
19.189.998
19.189.998
19.189.998
19.189.998
45.442.995
