# Supplementary material for: Detection of selection signatures in Piemontese and Marchigiana cattle, two breeds with similar production aptitudes but different selection histories
Source: Genet Sel Evol. 2015 Jun 23;47(1):52. doi: 10.1186/s12711-015-0128-2 (PMC4476081; doi:10.1186/s12711-015-0128-2)
Supplement: Supplementary file 4 — List of 189 genes annotated in cattle detected using varLD. Description: This list includes all the bovine annotated genes derived from Bos taurus UMD 3.1/bosTau6 assembly that are present in the 0.5 Mb interval (0.25 Mb upstream and downstream) considered for each significant SNP using the varLD approach. [file 12711_2015_128_MOESM4_ESM.pdf]

Gene name	start bp	end bp	BTA
NAB1	5567015	5603683	2
INPP1	5867811	5903148	2
C2H2orf88	6038401	6113008	2
NGF	28124614	28125504	3
TSPAN2	28364795	28449607	3
TSHB	28419562	28424798	3
ZNF800	92423261	92444888	4
GRM8	91470675	92316363	4
MIR592	92126803	92126899	4
RBFOX2	74255691	74544965	5
LOC515697	74673522	74688915	5
RBFOX2	74255691	74544965	5
LOC515697	74673522	74688915	5
LOC510193	74935712	74959664	5
APOL3_AA	74974806	74986756	5
PKD2	38040870	38099549	6
SPP1	38120577	38127541	6
HERC6	37736137	37793279	6
PPM1K	37876469	37898525	6
ABCG2	37959535	38030586	6
HERC5	37683705	37728602	6
IBSP	38309554	38323303	6
MEPE	38279590	38293661	6
FEM1A	20565298	20568880	7
ARRDC5	20473897	20486896	7
MIR7-3	20584156	20584238	7
TNFAIP8L1	20697556	20716655	7
UBXN6	20879419	20891487	7
HDGFRP2	20843765	20861798	7
TICAM1	20547954	20550264	7
FSD1	20979097	20990894	7
CHAF1A	20893519	20918558	7
PLIN5	20816180	20827918	7
PLIN3	20503791	20524337	7
LRG1	20812033	20814992	7
C7H19orf10	20683773	20695706	7
STAP2	20966850	20978543	7
SH3GL1	20923761	20951051	7
SHD	21002489	21010652	7
CCDC94	21017251	21031250	7
TNFAIP8L1	20697556	20716655	7
UBXN6	20879419	20891487	7
HDGFRP2	20843765	20861798	7
SIRT6	21079143	21087378	7
CREB3L3	21088128	21099619	7
EBI3	21033473	21040668	7
PIAS4	21204179	21227471	7

ZBTB7A	21179395	21196337	7
MAP2K2	21132388	21153987	7
LDHAL6B	95367660	95368977	9
TMEM242	95353541	95394421	9
MIR2481	95289914	95289978	9
ZDHHC14	95439712	95744856	9
CHRM5	28577030	28578629	10
EMC7	28548985	28564465	10
CCT4	60397413	60410035	11
XPO1	60068220	60109922	11
COMMD1	60427664	60598439	11
B3GNT2	60654633	60687732	11
CCT4	60397413	60410035	11
RNF219	54102933	54147844	12
CDK5RAP1	63368940	63403771	13
BPIFA2C	63086221	63095440	13
BPIFA2B	63165305	63175676	13
BPIFA3	63227592	63239140	13
BPIFA1	63247710	63254775	13
BPIFA2A	63041966	63053506	13
SNTA1	63408785	63490256	13
BPIFB5	63311350	63329709	13
BPIFB1	63284372	63305522	13
CHMP4B	63817039	63849783	13
E2F1	63704816	63714008	13
ZNF341	63750179	63790156	13
PXMP4	63726541	63743492	13
NECAB3	63684234	63703230	13
ARRB1	55255744	55329451	15
XRRA1	54931231	54968552	15
CHRDL2	54785593	54819151	15
NEU3	55029089	55043623	15
SLCO2B1	55151084	55203574	15
RPS3	55370366	55375312	15
MIR326	55314008	55314103	15
PARK7	46249162	46265920	16
ERRFI1	46201191	46215456	16
VAMP3	46455175	46464710	16
TNFRSF9	46288365	46306440	16
C16H1orf74	75421210	75423469	16
LAMB3	75567713	75610921	16
G0S2	75539495	75540404	16
IRF6	75401220	75417467	16
MIR205	75799965	75800034	16
TRAF3IP3	75426163	75446749	16
CAMK1G	75611810	75642910	16
HSD11B1	75463611	75508770	16
DIEXF	75346249	75373188	16

RNF10	65067804	65101420	17
SRSF9	65008116	65014401	17
MSI1	64914953	64937019	17
COX6A1	64995247	64997122	17
SIRT4	64875356	64891607	17
RPLP0	64809253	64813277	17
DYNLL1	65042595	65045026	17
PLA2G1B	64899558	64902628	17
GATC	65000549	65007059	17
RAB35	64724241	64742928	17
TRIAP1	64998264	65000579	17
MLEC	65184867	65194880	17
CABP1	65154258	65170682	17
UNC119B	65206693	65216028	17
ACADS	65220604	65237484	17
PSMB10	35540354	35542906	18
SLC7A6OS	35795186	35803175	18
DUS2L	35602602	35634597	18
SMPD3	35847628	35872312	18
DDX28	35600675	35602509	18
PLA2G15	35747166	35760966	18
LCAT	35544365	35547611	18
SLC7A6	35763842	35794542	18
SLC12A4	35547821	35569646	18
ESRP2	35734986	35741010	18
NFATC3	35640708	35732176	18
PSKH1	35510858	35535918	18
PRMT7	35803281	35844344	18
PAFAH1B1	24112442	24162110	19
METTL16	23961073	24023406	19
C19H17orf59	28443495	28445326	19
KCNAB3	28214103	28220774	19
TMEM107	28432874	28435112	19
ALOX12B	28329830	28341331	19
SLC25A35	28521122	28524591	19
GUCY2D	28274098	28288581	19
ARHGEF15	28541732	28550394	19
MYH10	28680826	28800880	19
RANGRF	28519718	28521136	19
TRAPPC1	28221788	28223283	19
VAMP2_AA	28408574	28410802	19
PFAS	28498628	28511705	19
ALOX15B	28306790	28315605	19
ODF4	28565346	28570383	19
HES7	28375016	28377490	19
PER1	28390015	28399748	19
NDEL1	28637768	28673102	19
RPL26	28590311	28595225	19

AURKB	28447632	28452478	19
NPR3	40965737	41042094	20
SUB1	41122022	41143914	20
CDH18	53409875	54014026	20
MIR2361	53415662	53415738	20
NMB	22741474	22744781	21
WDR73	22735035	22740454	21
SEC11A	22754372	22787431	21
SLC28A1	22949799	23048041	21
ZNF592	22822828	22871700	21
MIR2364	22859904	22859978	21
PDE8A	23119314	23262117	21
KLHL18	52871072	52922306	22
NRADD	53073617	53077203	22
CCDC12	53110762	53147115	22
PTPN23	52818929	52844833	22
NGP	52858271	52862343	22
PRSS45	53296645	53304315	22
PRSS42	53236357	53240657	22
KIF9	52925930	52959210	22
MYL3	53202765	53208553	22
PTH1R	53161299	53224114	22
TMIE	53345153	53350541	22
ALS2CL	53358572	53380721	22
MOCS1	13832464	13866950	23
LRFN2	14309127	14351087	23
CHMP1B	43149700	43151533	24
GNAL	43111843	43163945	24
CIDEA	43217423	43235018	24
CEP76	43500035	43515274	24
TUBB6	43249499	43250810	24
AFG3L2	43252924	43271906	24
PSMG2	43515582	43525531	24
PTPN2	43539720	43607744	24
IMPA2	43189672	43209175	24
SEH1L	43639268	43659103	24
GNA12	41099258	41171209	25
CARD11	40960557	41068748	25
BRAT1	41206700	41216986	25
PAPSS2	9292115	9402158	26
ATAD1	9410908	9451790	26
RNLS	9932076	10211164	26
CLNS1A	18444586	18465090	29
AQP11	18477127	18488713	29
PAK1	18684327	18780241	29
MIR2285K-4	18714778	18908690	29
