# Supplementary material for: Detection of selection signatures in Piemontese and Marchigiana cattle, two breeds with similar production aptitudes but different selection histories
Source: Genet Sel Evol. 2015 Jun 23;47(1):52. doi: 10.1186/s12711-015-0128-2 (PMC4476081; doi:10.1186/s12711-015-0128-2)
Supplement: Supplementary file 5 — Plots obtained with the Fst method vs the varLD method. Description: Chromosome-wide plots obtained in the Fst and varLD analyses for BTA1 to 29. Solid lines represent the threshold set at three standard deviations apart from the mean value. [file 12711_2015_128_MOESM5_ESM.pdf]

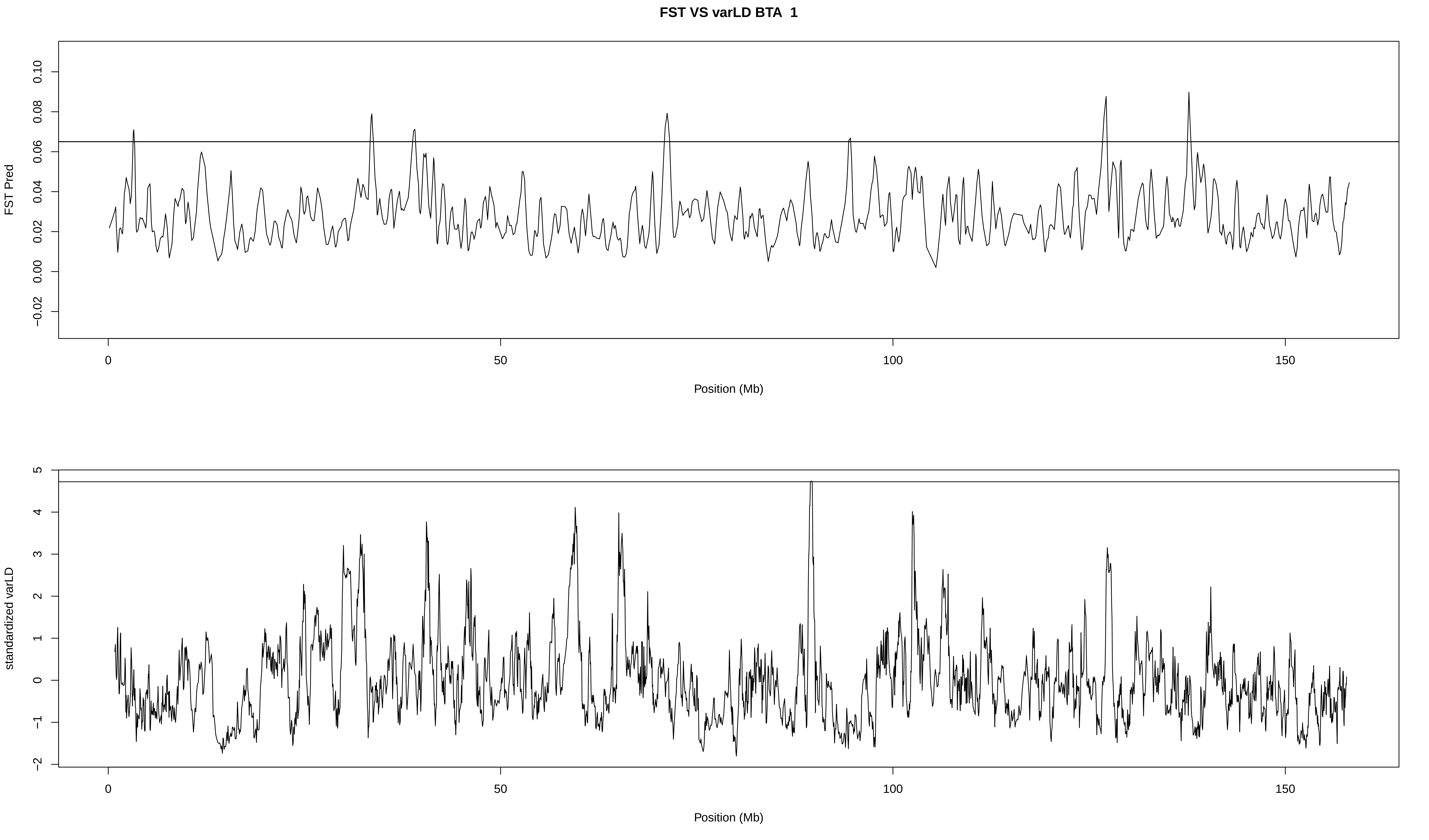
FST VS varLD BTA 1
FST Pred
−0.02
0.00
0.02
0.04
0.06
0.08
0.10
0
50
100
150
Position (Mb)
standardized varLD
−2
−1
0
1
2
3
4
5
0
50
100
150
Position (Mb)

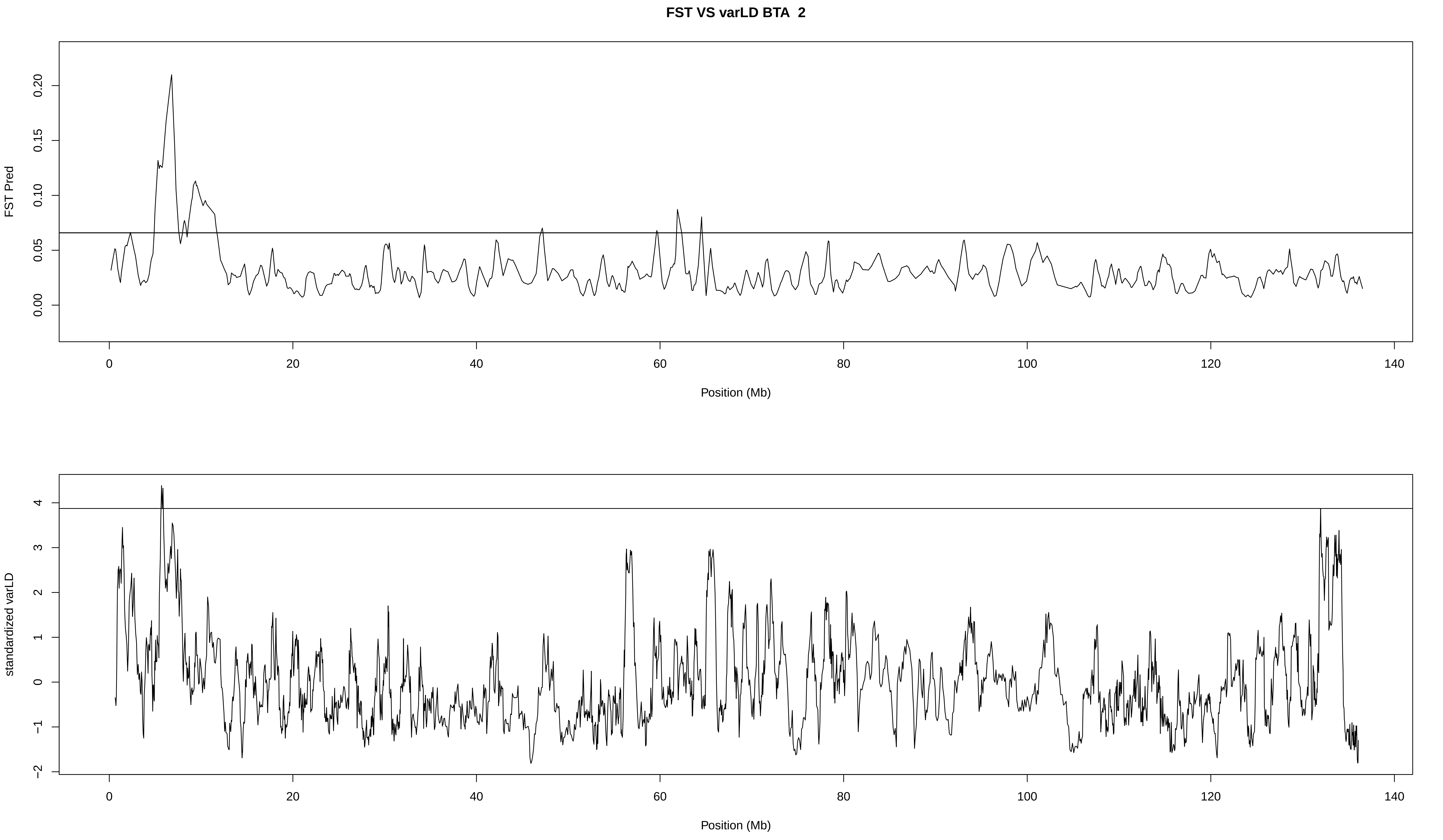
FST VS varLD BTA 2
FST Pred
0.00
0.05
0.10
0.15
0.20
0
20
40
60
80
100
120
140
Position (Mb)
standardized varLD
−2
−1
0
1
2
3
4
0
20
40
60
80
100
120
140
Position (Mb)

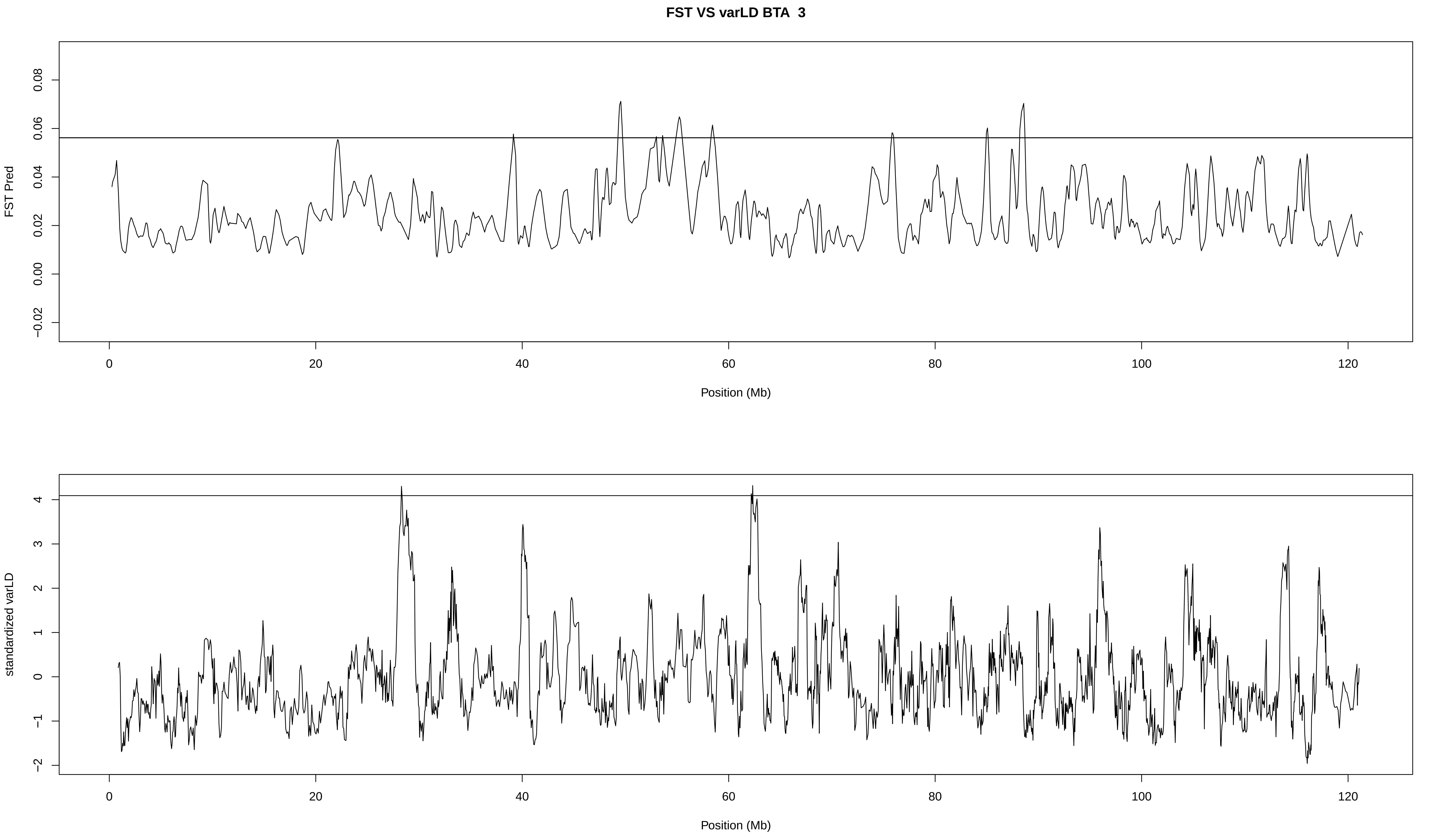

FST VS varLD BTA 3
FST Pred
0.08
0.06
0.04
0.02
0.00
−0.02
0
20
40
60
80
100
120
Position (Mb)
standardized varLD
4
3
2
1
0
−1
−2
0
20
40
60
80
100
120
Position (Mb)

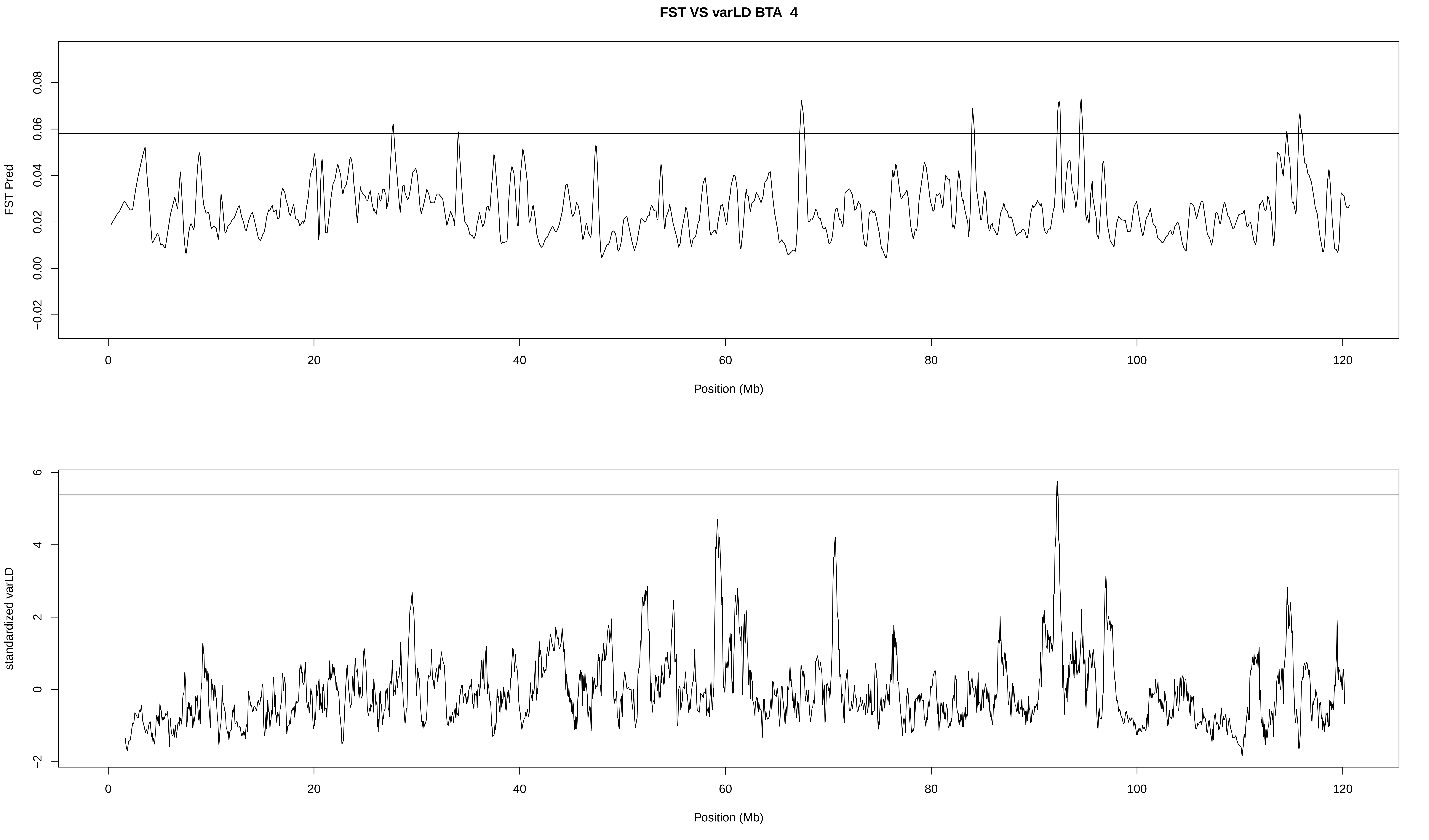
FST VS varLD BTA 4
FST Pred
0.08
0.06
0.04
0.02
0.00
−0.02
0
20
40
60
80
100
120
Position (Mb)
standardized varLD
6
4
2
0
−2
0
20
40
60
80
100
120
Position (Mb)

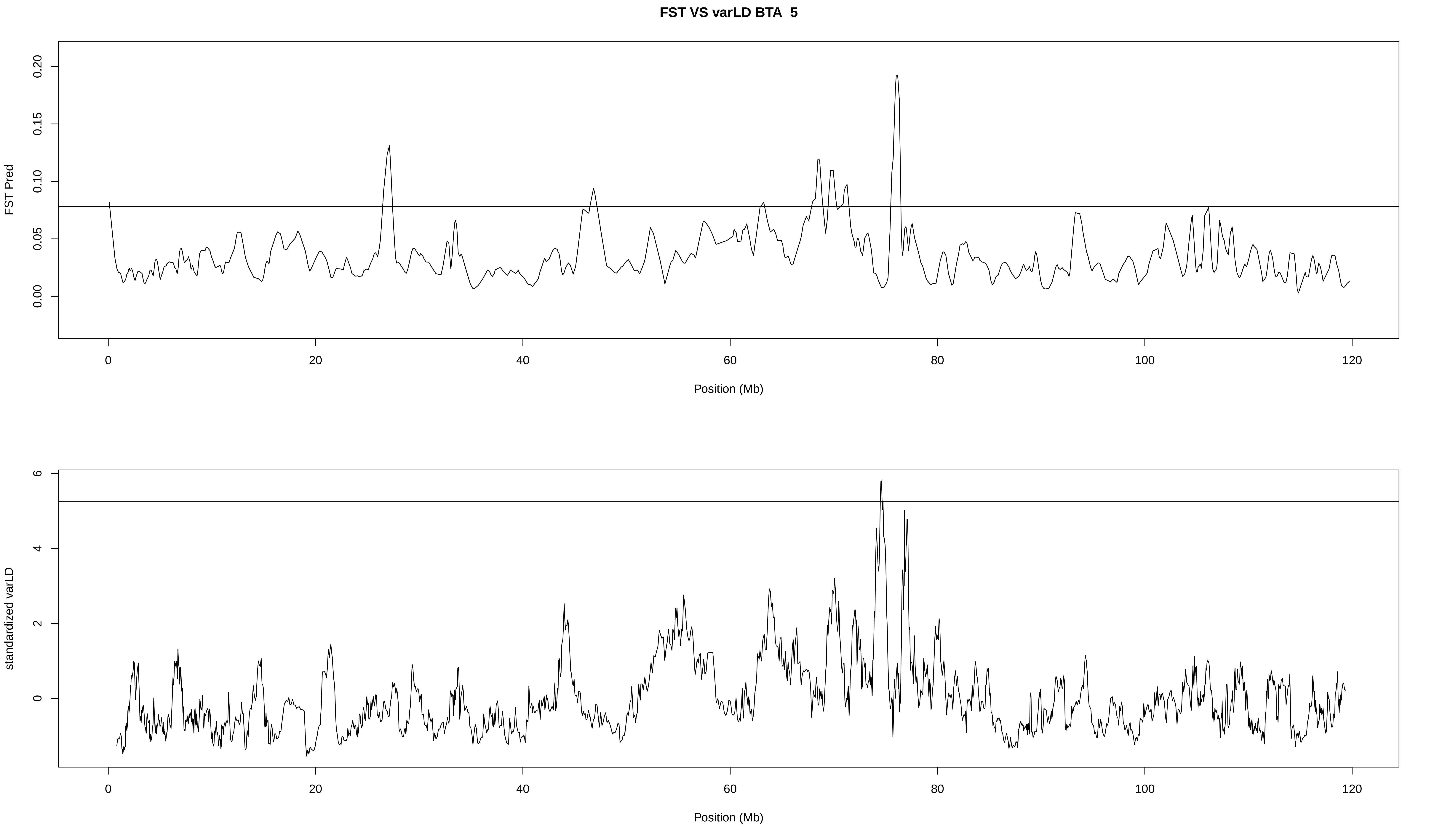
FST VS varLD BTA 5
FST Pred
0.00
0.05
0.10
0.15
0.20
0
20
40
60
80
100
120
Position (Mb)
standardized varLD
0
2
4
6
0
20
40
60
80
100
120
Position (Mb)

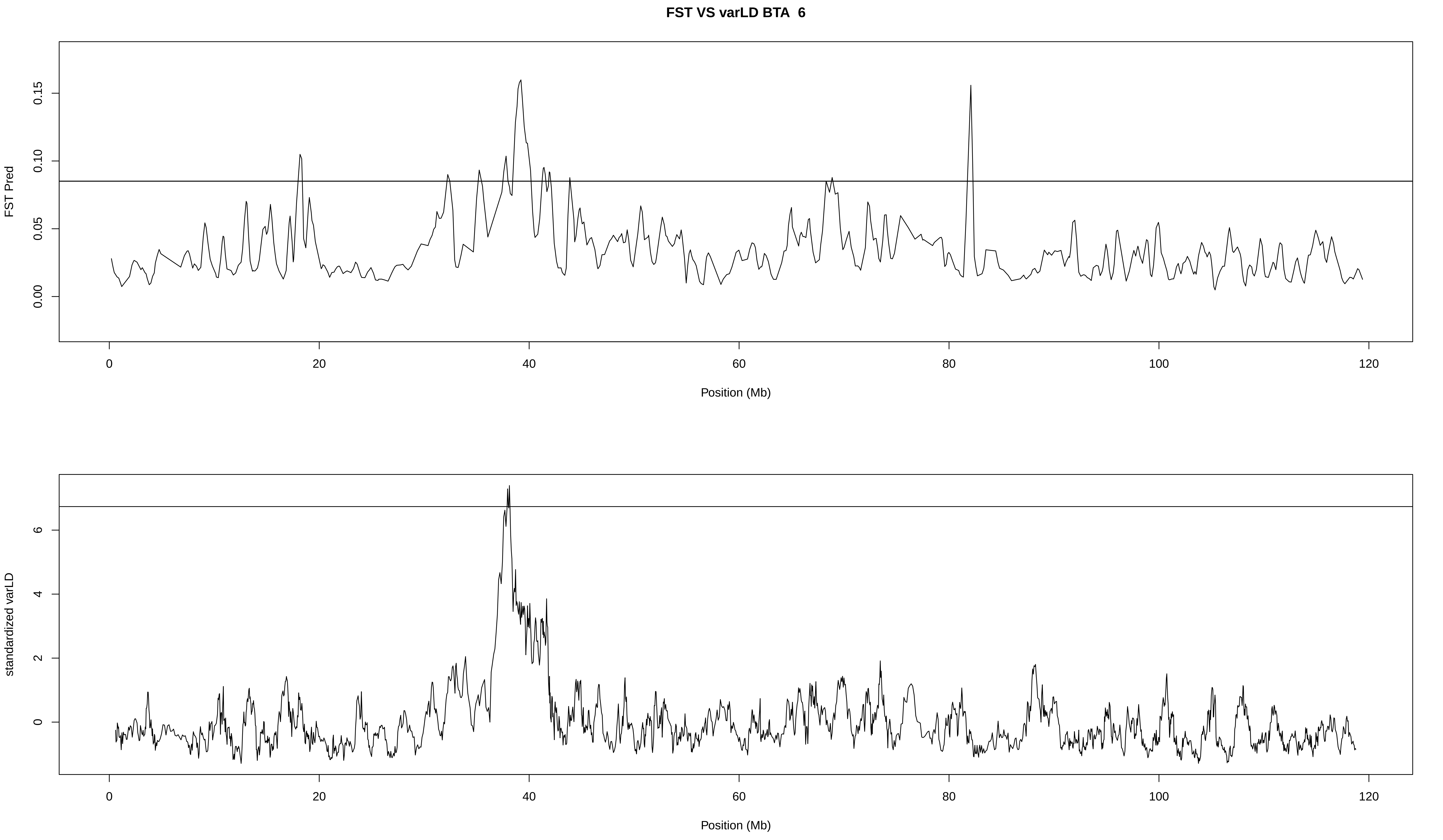
FST VS varLD BTA 6
FST Pred
0.00
0.05
0.10
0.15
0
20
40
60
80
100
120
Position (Mb)
standardized varLD
0
2
4
6
0
20
40
60
80
100
120
Position (Mb)

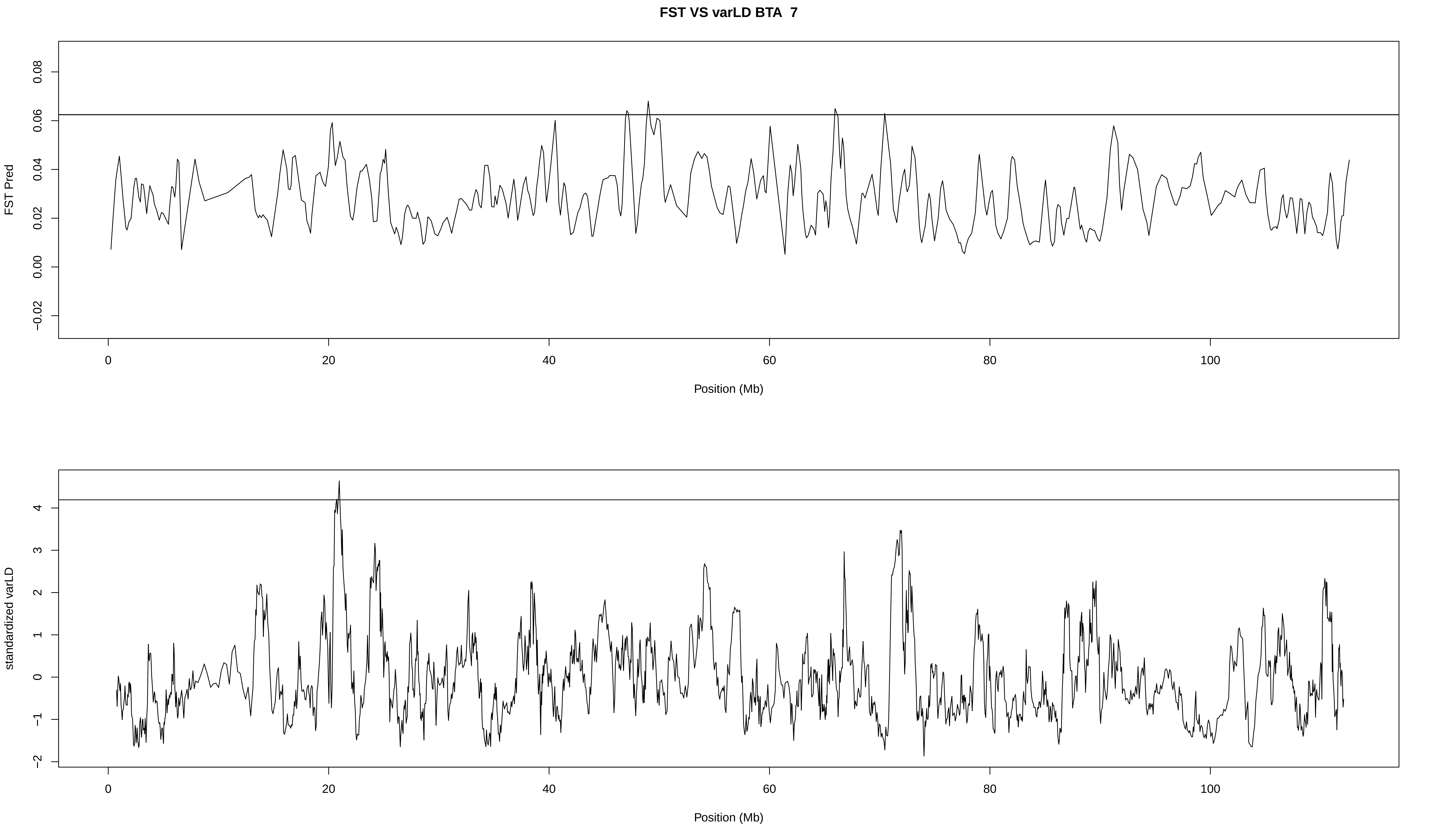
FST VS varLD BTA 7
FST Pred
Position (Mb)
standardized varLD
Position (Mb)

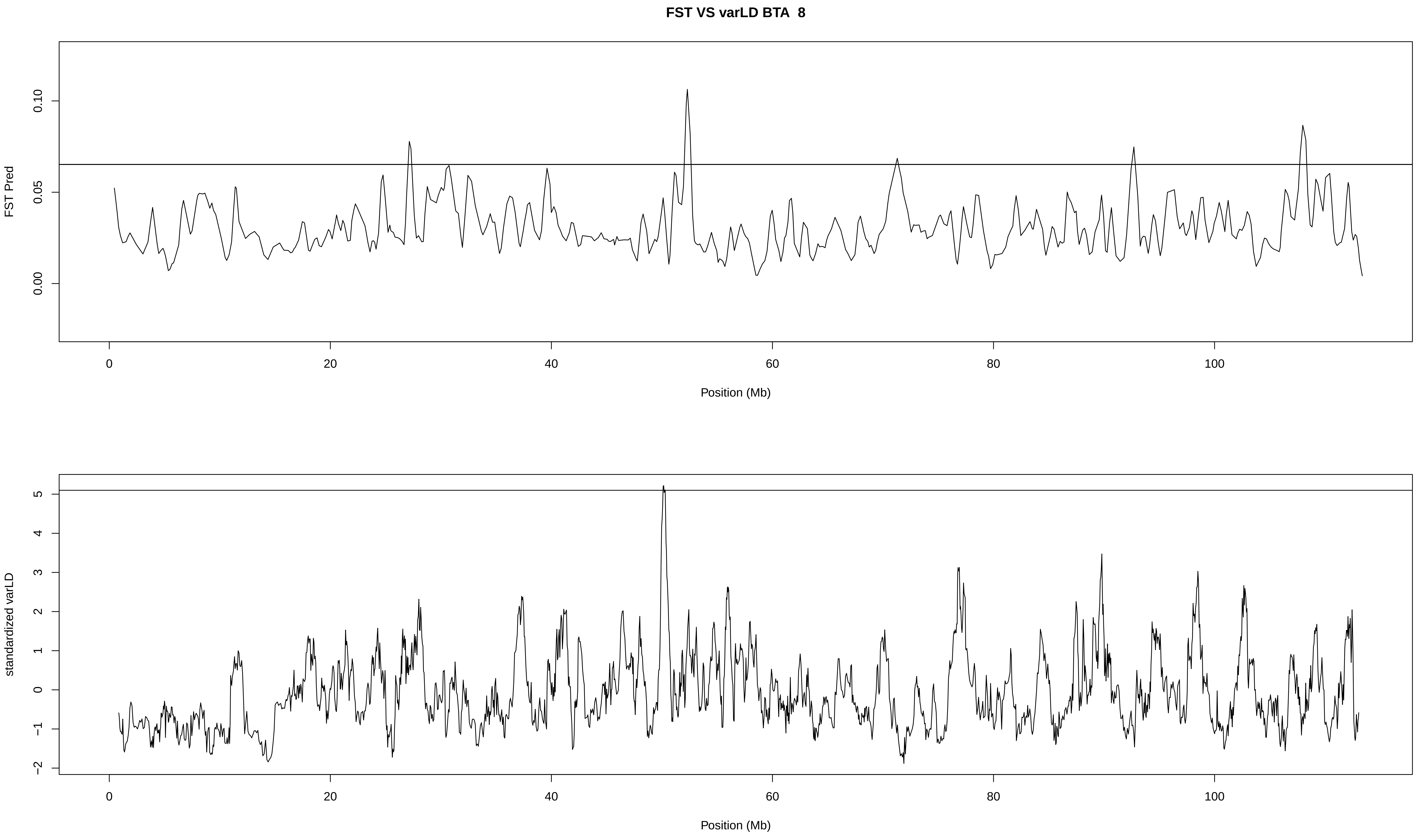

FST VS varLD BTA 8
FST Pred
0.00
0.05
0.10
0
20
40
60
80
100
Position (Mb)
standardized varLD
−2
−1
0
1
2
3
4
5
0
20
40
60
80
100
Position (Mb)

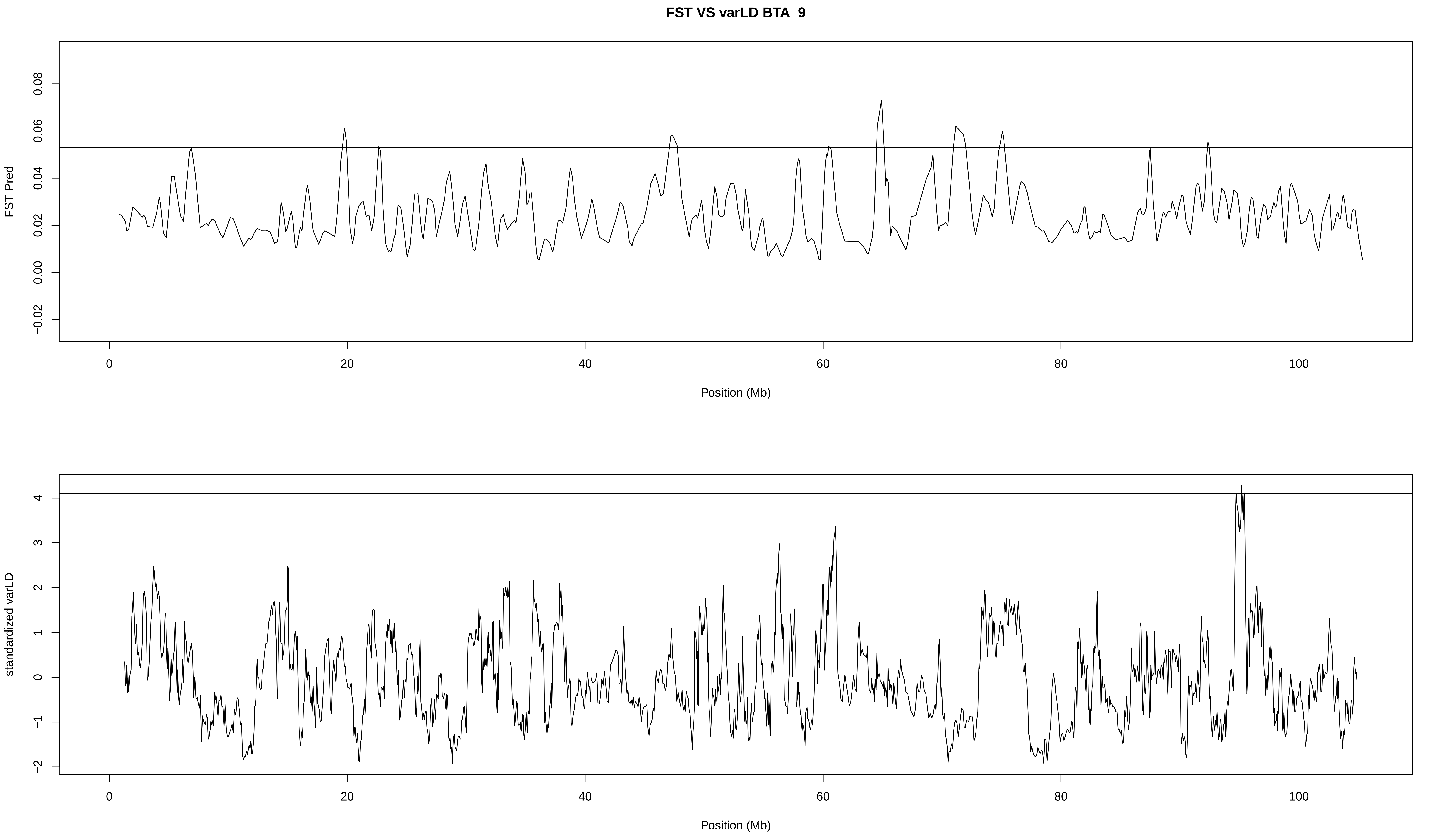
FST VS varLD BTA 9
FST Pred
0.08
0.06
0.04
0.02
0.00
−0.02
0
20
40
60
80
100
Position (Mb)
standardized varLD
4
3
2
1
0
−1
−2
0
20
40
60
80
100
Position (Mb)

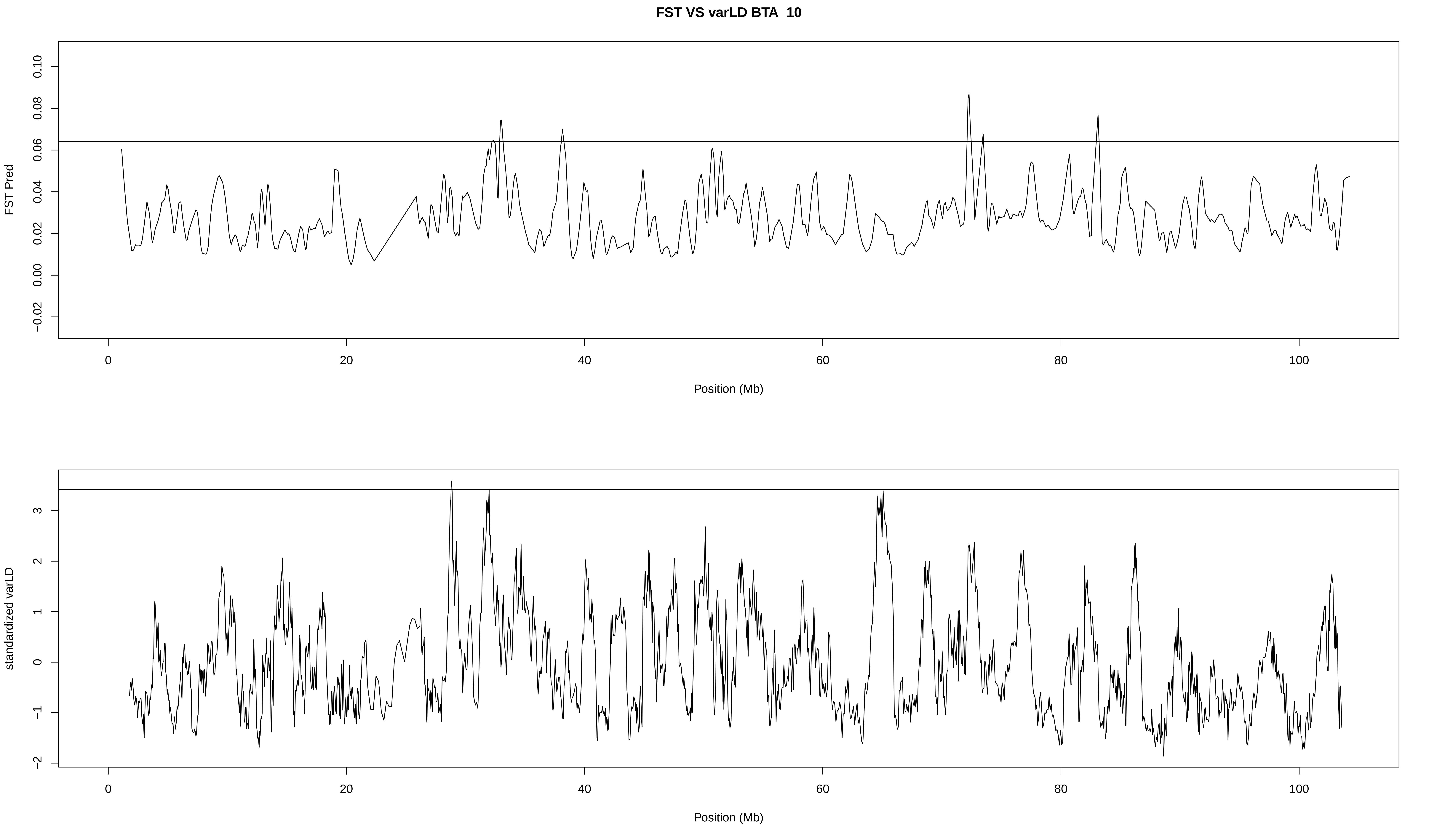
FST VS varLD BTA 10
FST Pred
−0.02
0.00
0.02
0.04
0.06
0.08
0.10
0
20
40
60
80
100
Position (Mb)
standardized varLD
−2
−1
0
1
2
3
0
20
40
60
80
100
Position (Mb)

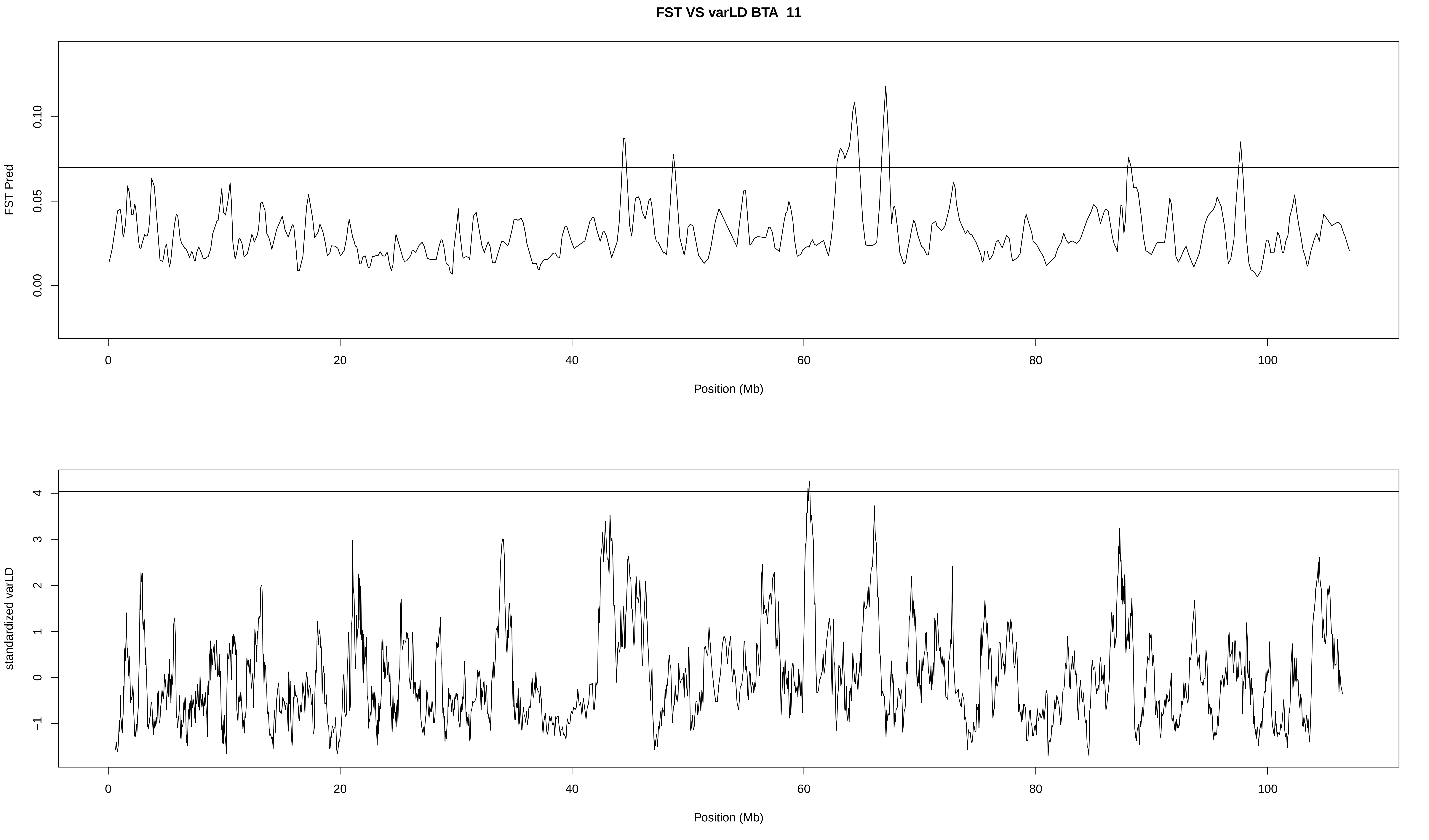
FST VS varLD BTA 11
FST Pred
0.00
0.05
0.10
0
20
40
60
80
100
Position (Mb)
standardized varLD
-1
0
1
2
3
4
0
20
40
60
80
100
Position (Mb)

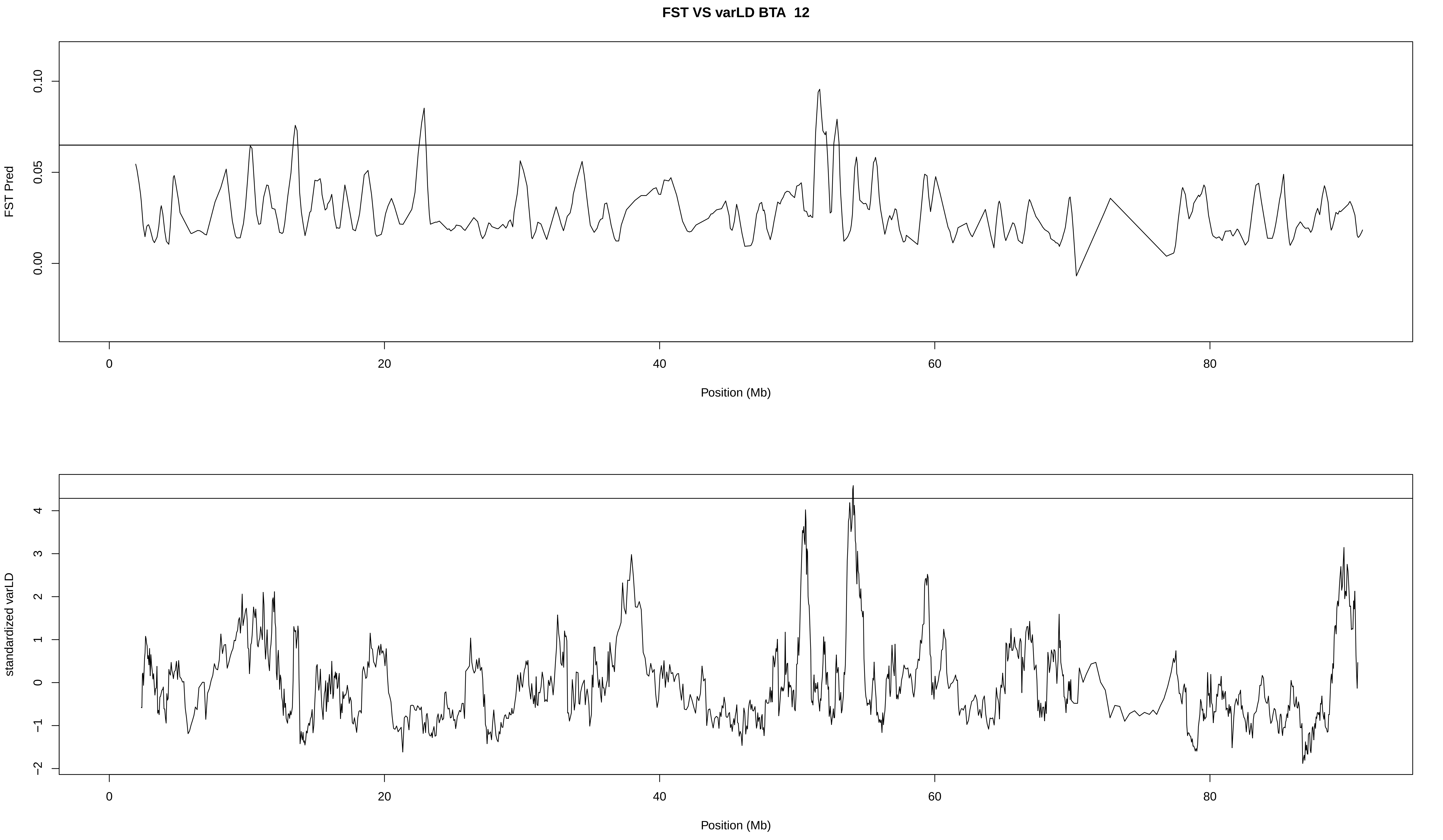

FST VS varLD BTA 12
FST Pred
0.10
0.05
0.00
0
20
40
60
80
Position (Mb)
standardized varLD
4
3
2
1
0
−1
−2
0
20
40
60
80
Position (Mb)

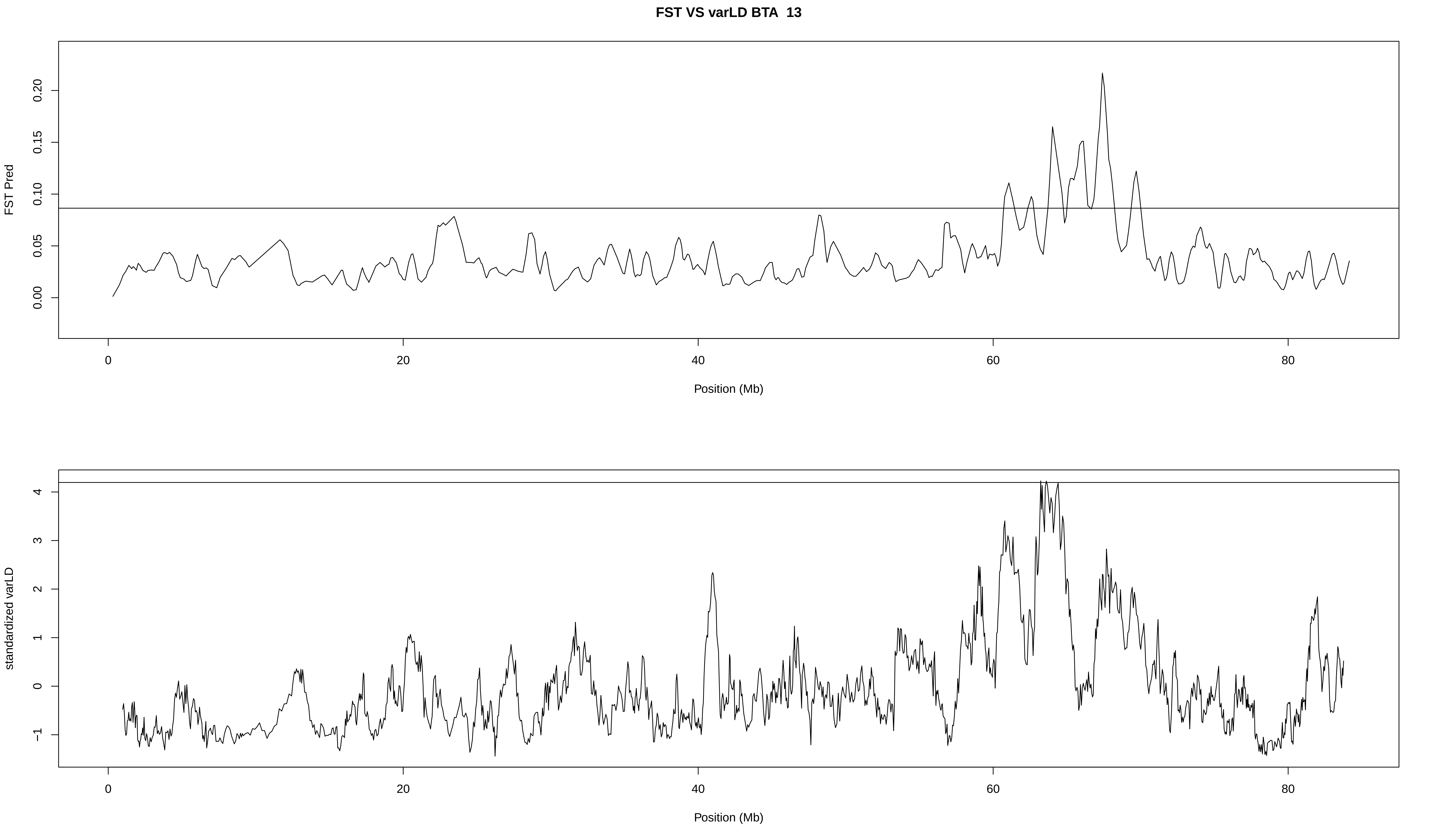
FST VS varLD BTA 13
FST Pred
0.00
0.05
0.10
0.15
0.20
0
20
40
60
80
Position (Mb)
standardized varLD
−1
0
1
2
3
4
0
20
40
60
80
Position (Mb)

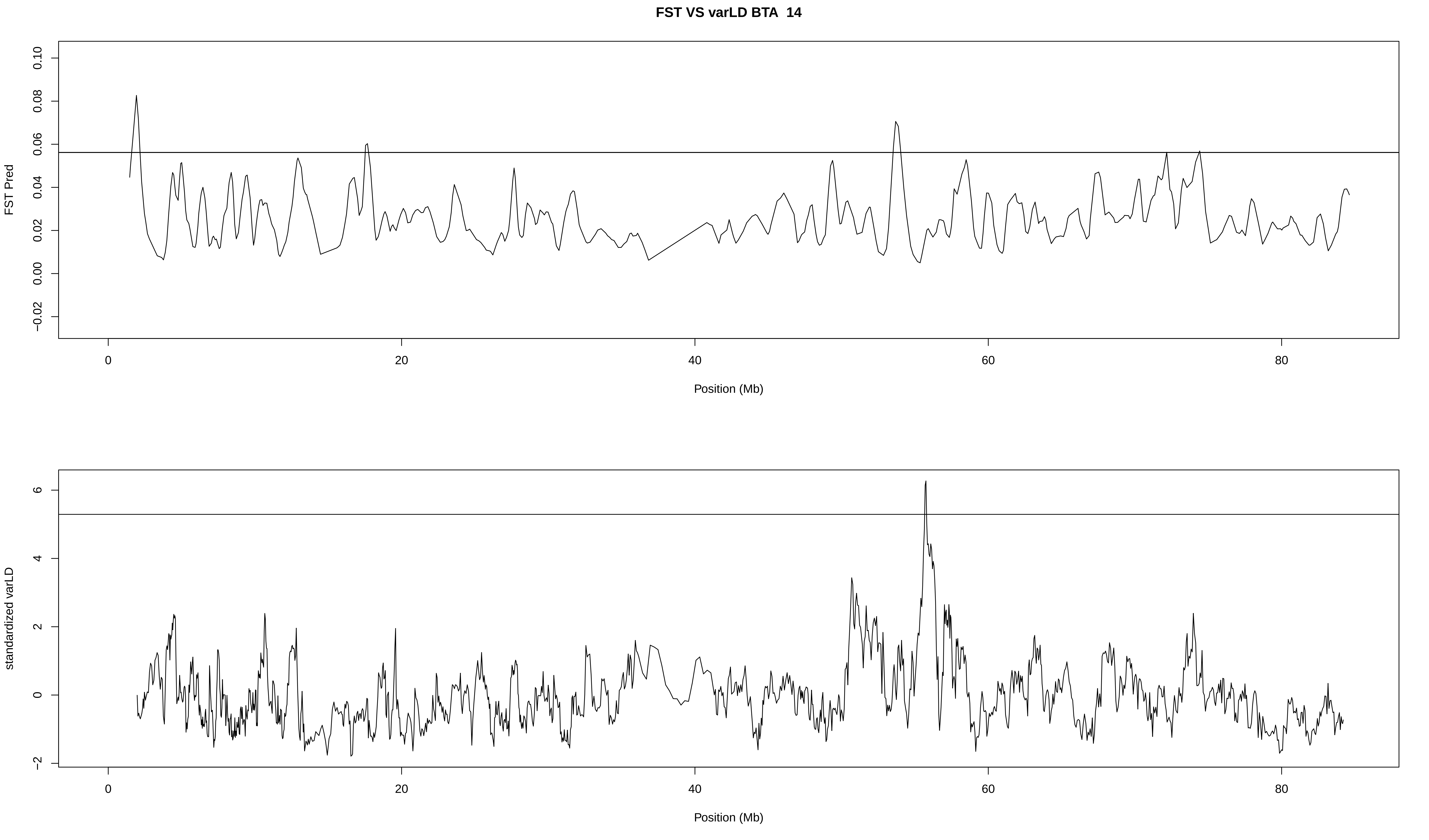

FST VS varLD BTA 14
FST Pred
0.10
0.08
0.06
0.04
0.02
0.00
−0.02
0
20
40
60
80
Position (Mb)
standardized varLD
6
4
2
0
−2
0
20
40
60
80
Position (Mb)

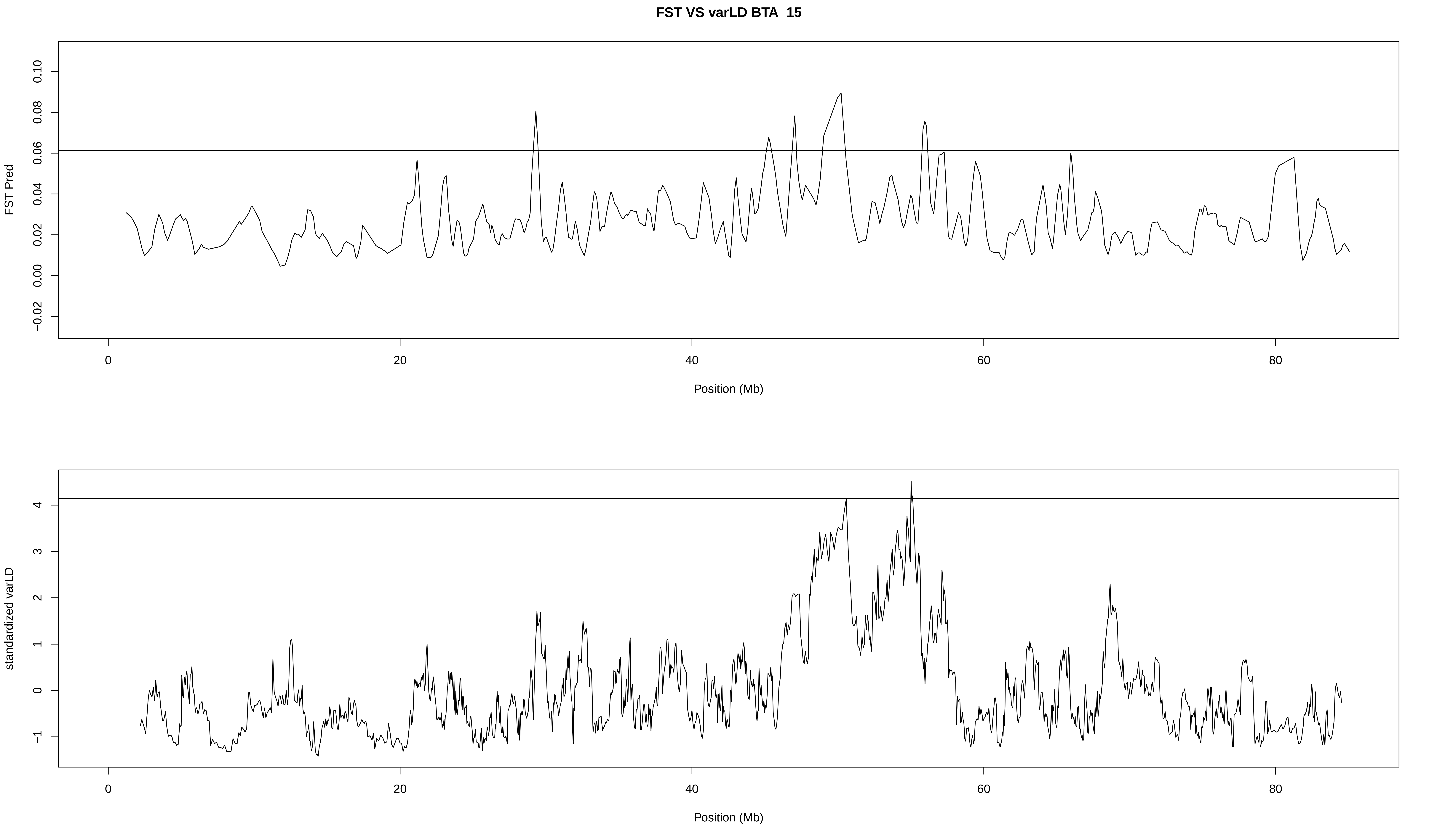

FST VS varLD BTA 15
FST Pred
0.10
0.08
0.06
0.04
0.02
0.00
−0.02
0
20
40
60
80
Position (Mb)
standardized varLD
4
3
2
1
0
−1
0
20
40
60
80
Position (Mb)

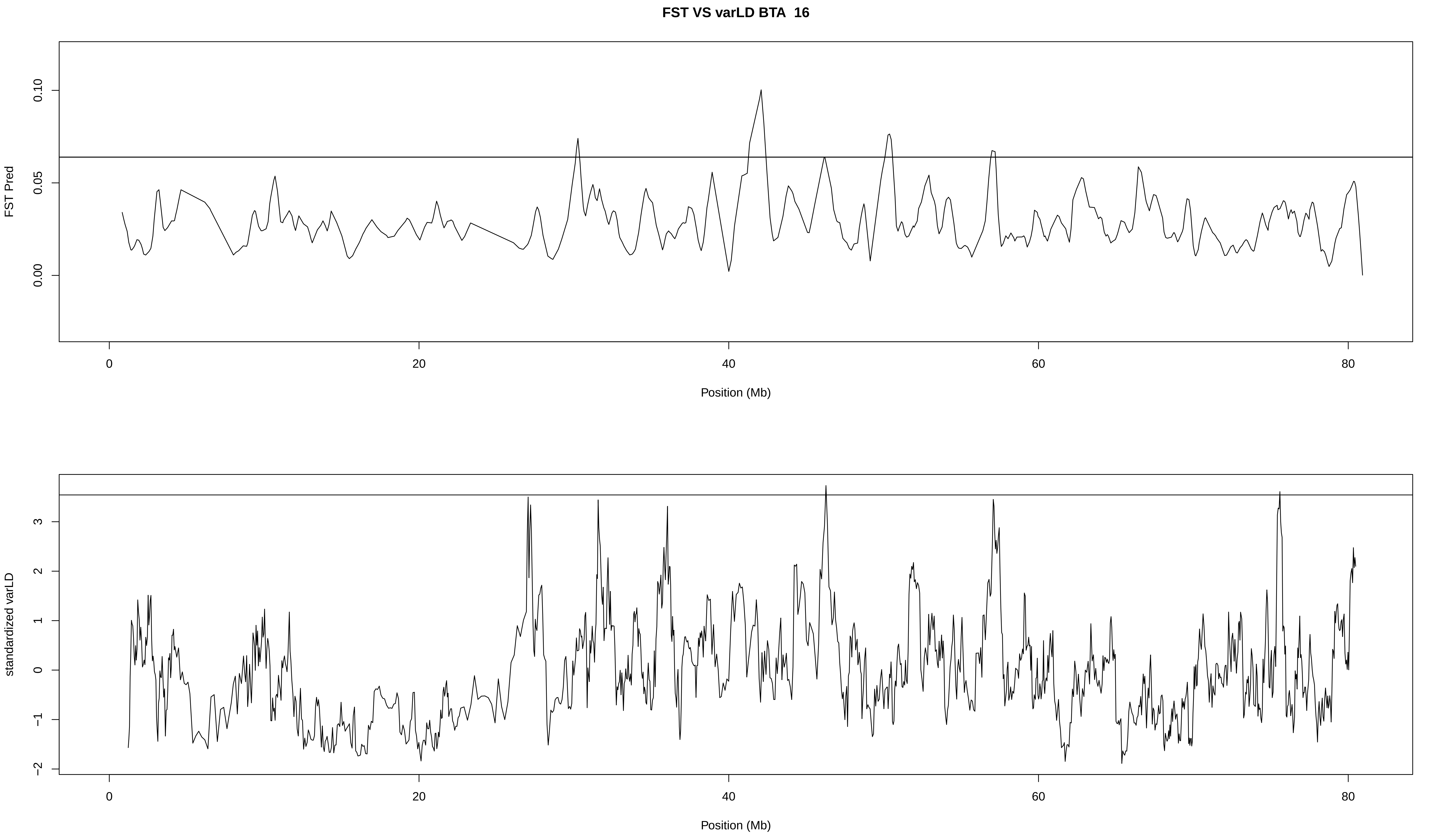

FST VS varLD BTA 16
FST Pred
0.10
0.05
0.00
0
20
40
60
80
Position (Mb)
standardized varLD
3
2
1
0
−1
−2
0
20
40
60
80
Position (Mb)

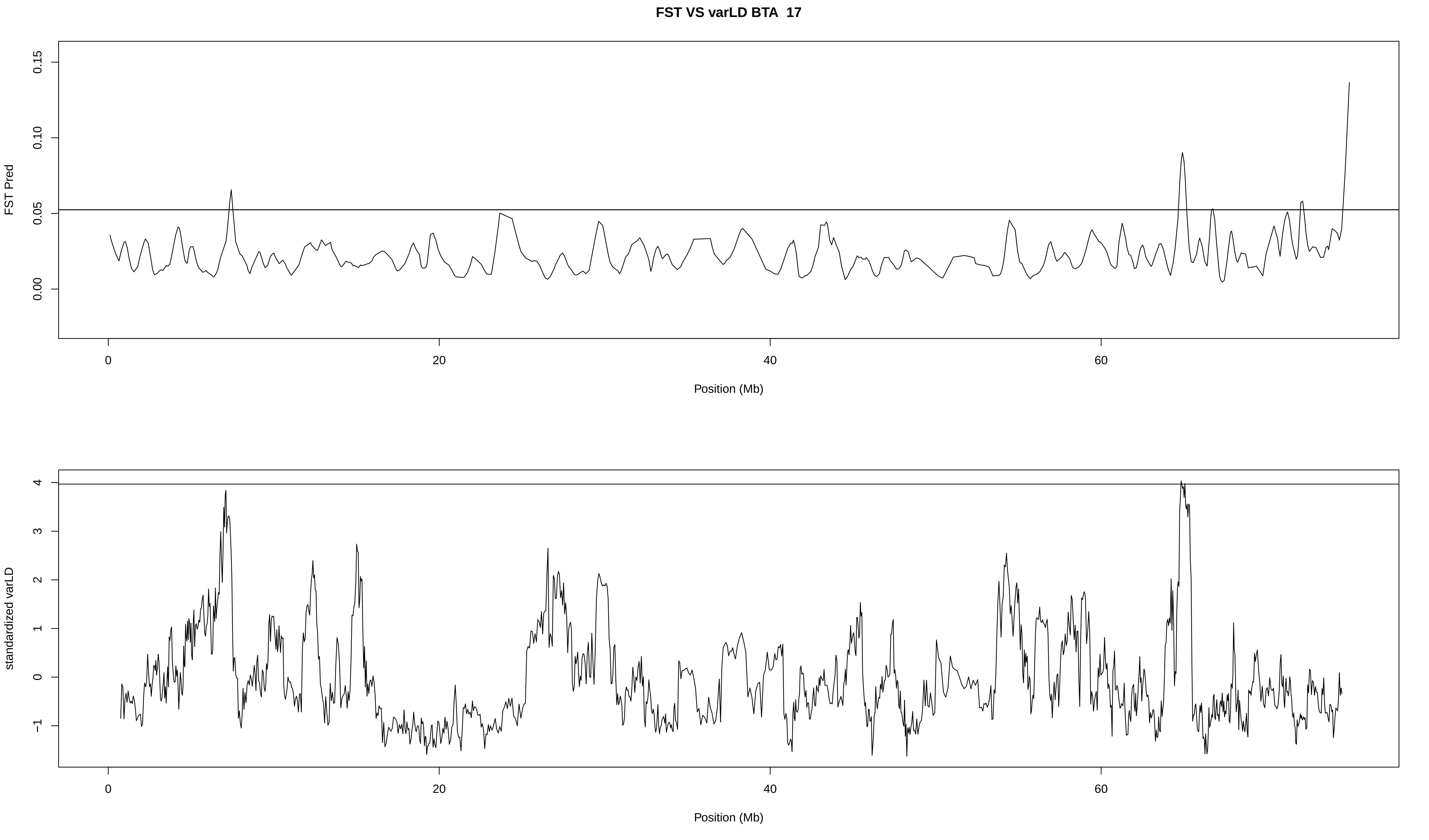
FST VS varLD BTA 17
FST Pred
0.15
0.10
0.05
0.00
0
20
40
60
Position (Mb)
standardized varLD
4
3
2
1
0
−1
0
20
40
60
Position (Mb)

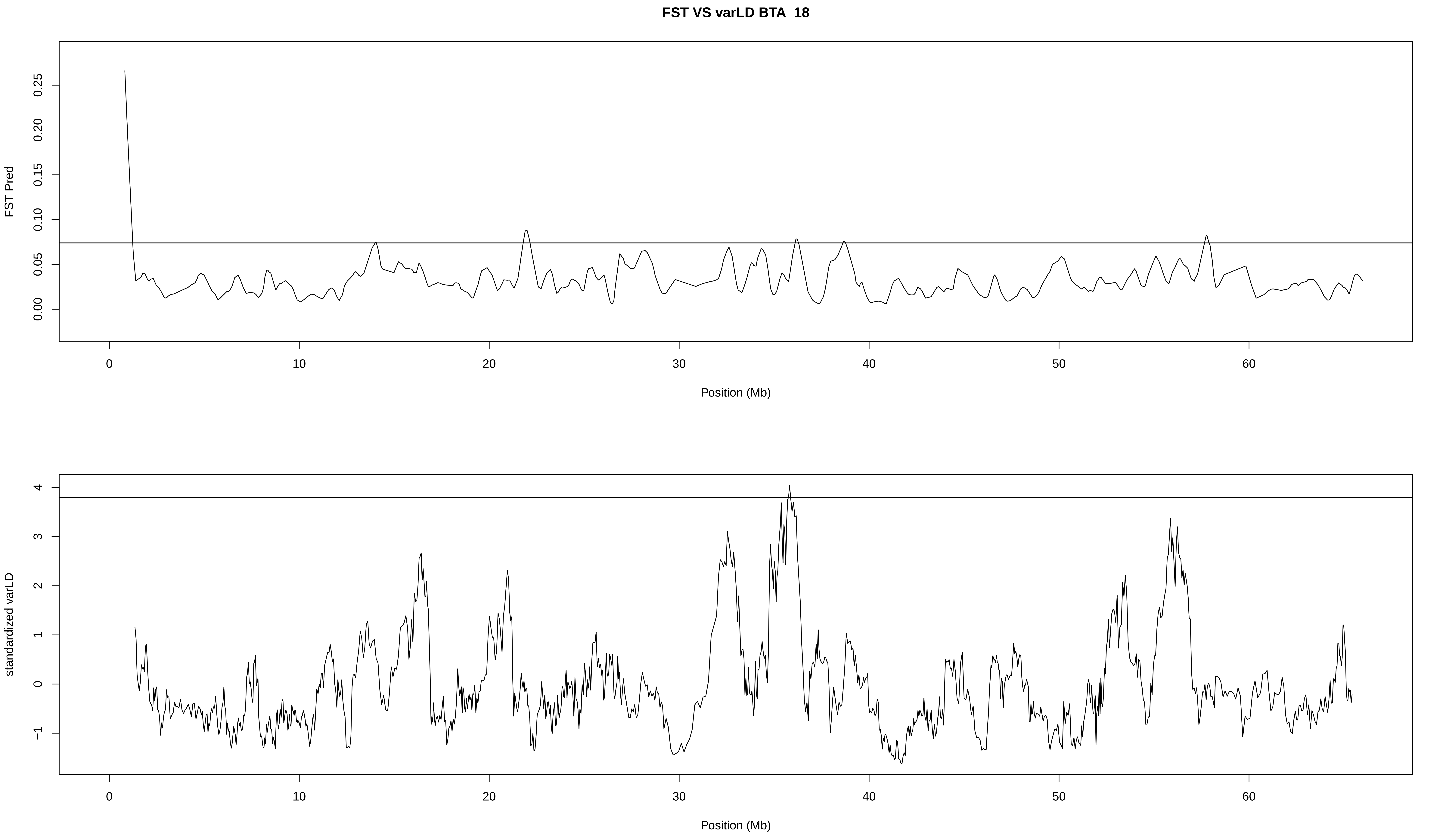
FST VS varLD BTA 18
FST Pred
0.00
0.05
0.10
0.15
0.20
0.25
0
10
20
30
40
50
60
Position (Mb)
standardized varLD
−1
0
1
2
3
4
0
10
20
30
40
50
60
Position (Mb)

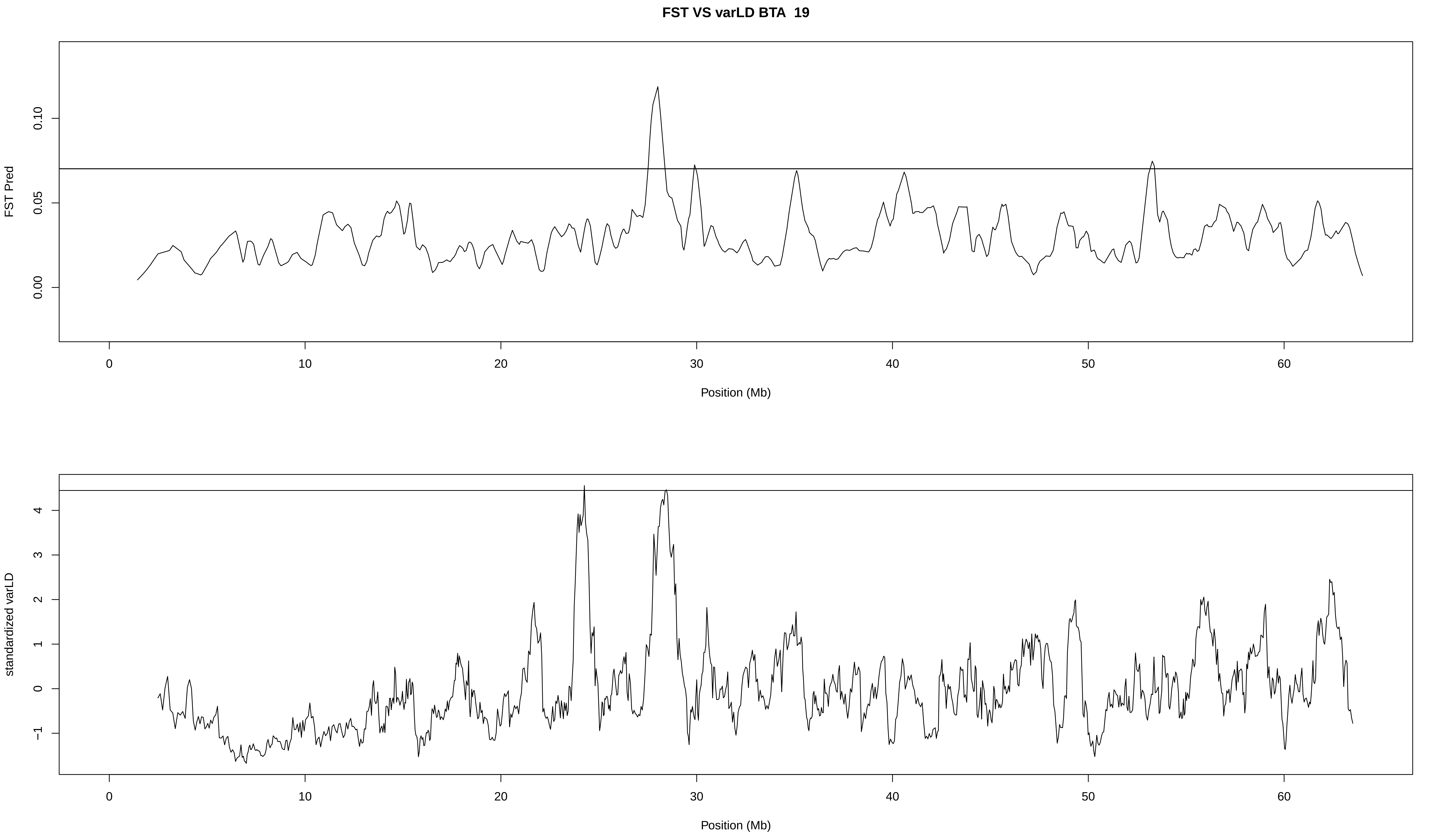
FST VS varLD BTA 19
FST Pred
0.00
0.05
0.10
Position (Mb)
0
10
20
30
40
50
60
standardized varLD
−1
0
1
2
3
4
Position (Mb)

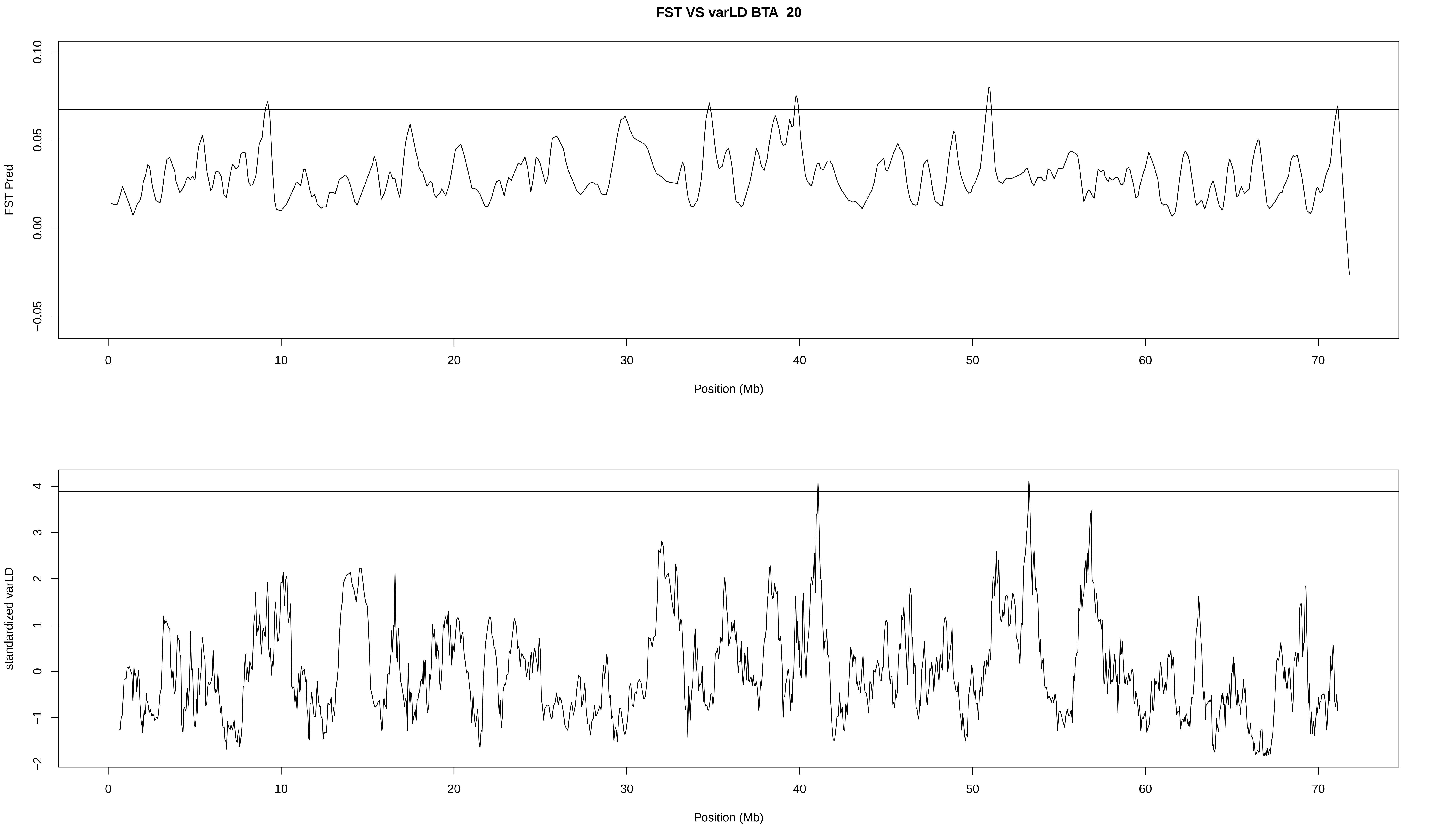
FST VS varLD BTA 20
FST Pred
0.10
0.05
0.00
−0.05
0
10
20
30
40
50
60
70
Position (Mb)
standardized varLD
4
3
2
1
0
−1
−2
0
10
20
30
40
50
60
70
Position (Mb)

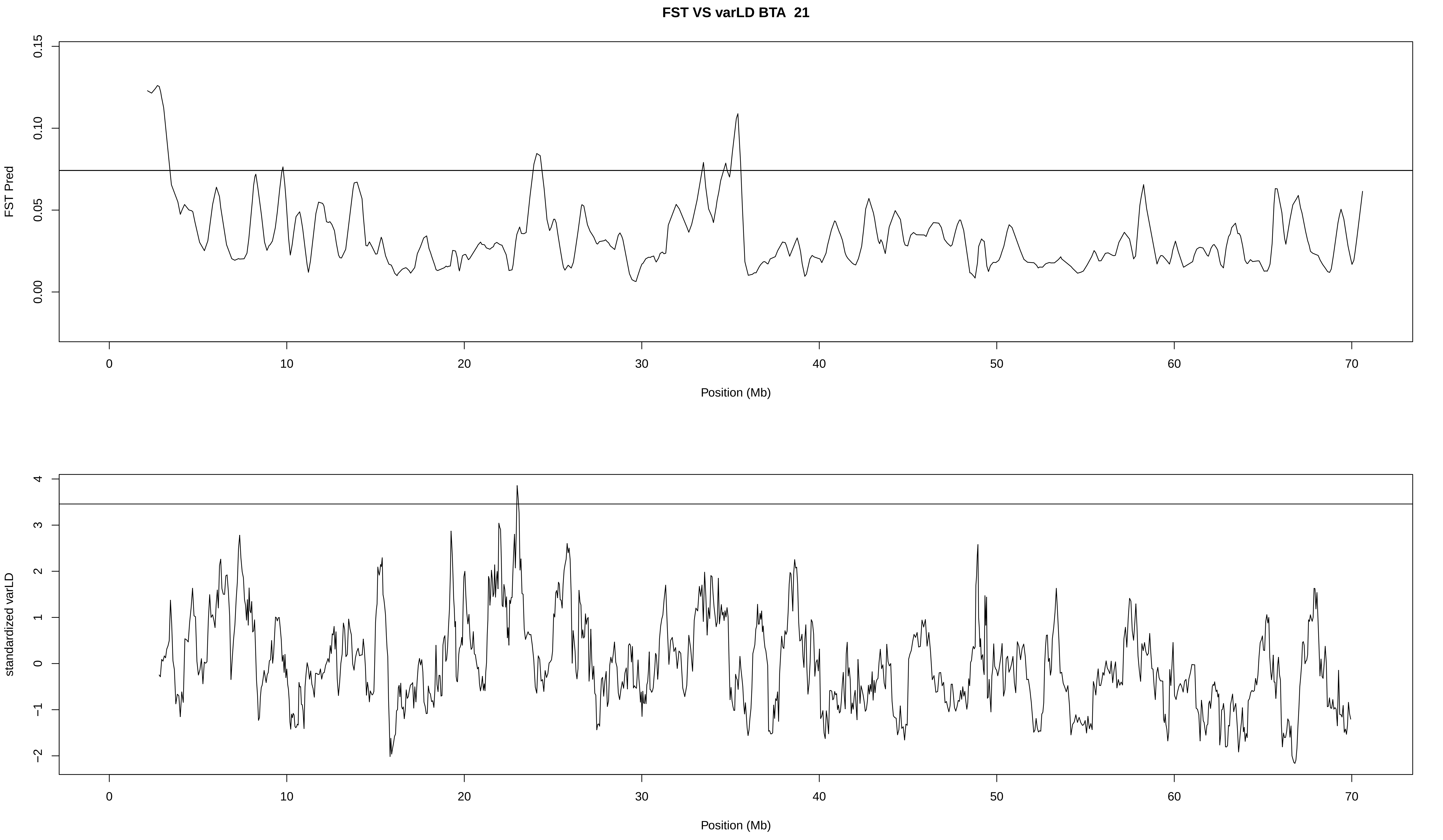

FST VS varLD BTA 21
FST Pred
0.15
0.10
0.05
0.00
Position (Mb)
0 10 20 30 40 50 60 70
standardized varLD
4 3 2 1 0 −1 −2
Position (Mb)
0 10 20 30 40 50 60 70

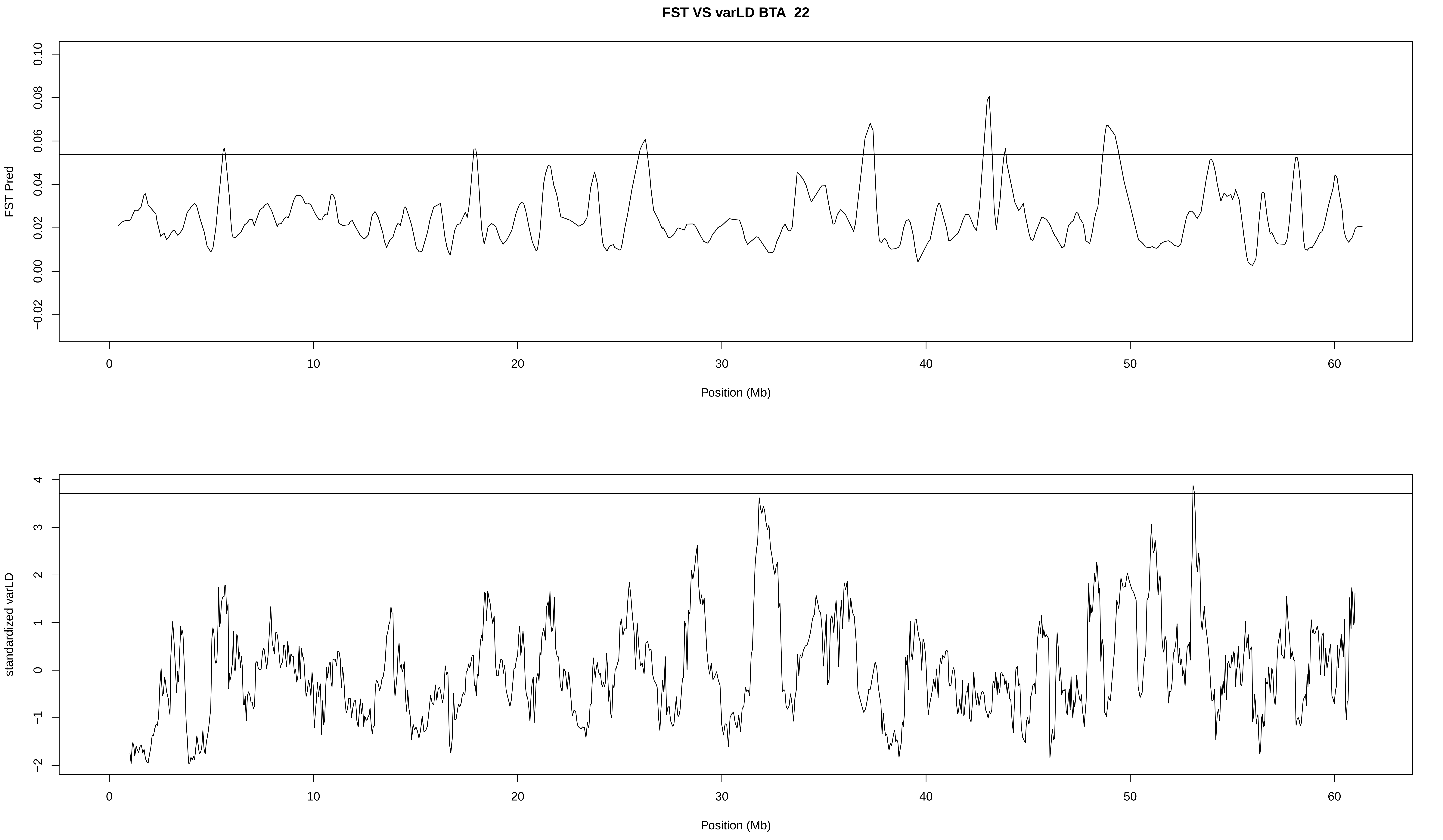
FST VS varLD BTA 22
FST Pred
0.10
0.08
0.06
0.04
0.02
0.00
−0.02
0
10
20
30
40
50
60
Position (Mb)
standardized varLD
4
3
2
1
0
−1
−2
0
10
20
30
40
50
60
Position (Mb)

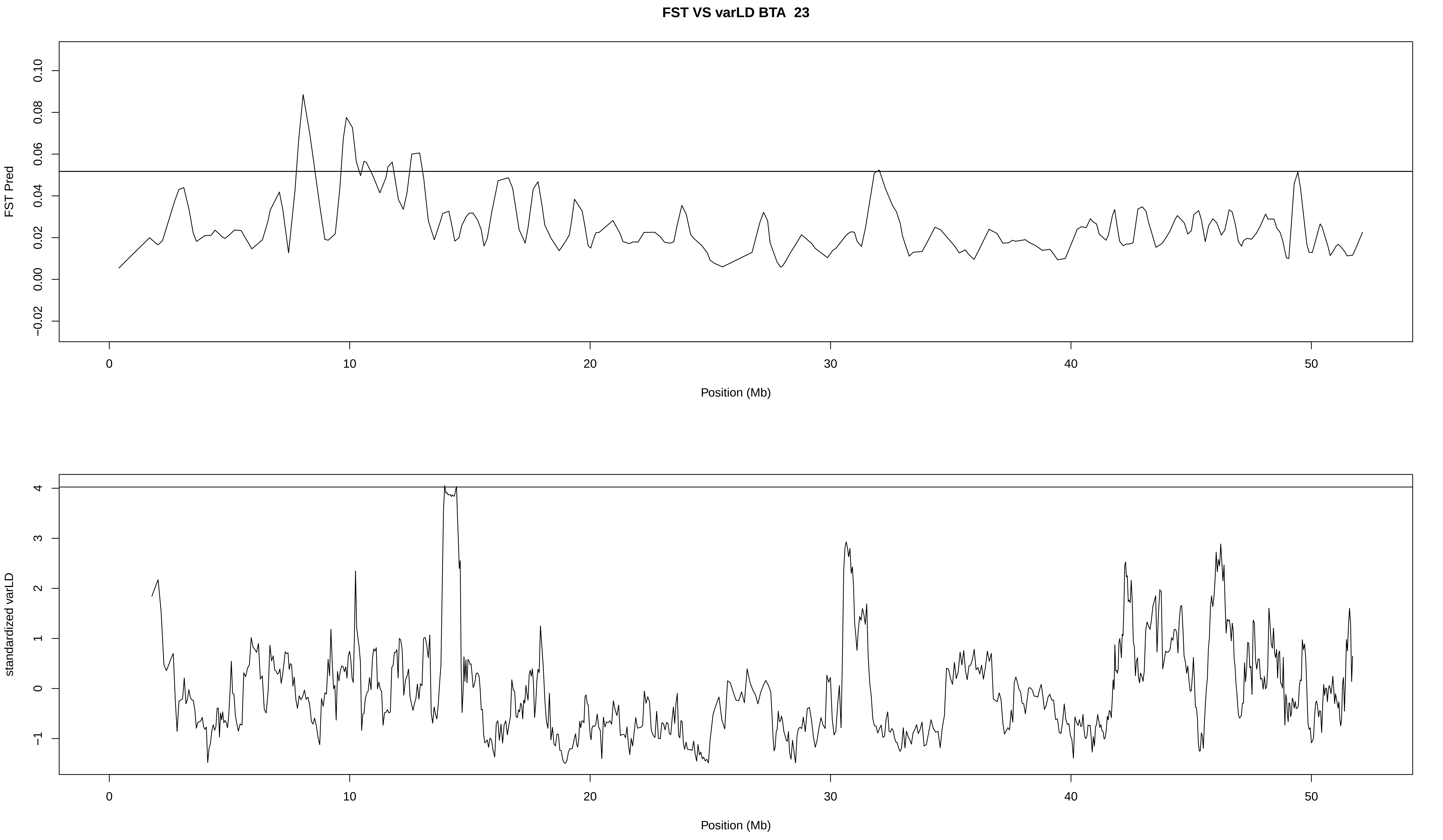

FST VS varLD BTA 23
FST Pred
0.10
0.08
0.06
0.04
0.02
0.00
−0.02
0
10
20
30
40
50
Position (Mb)
standardized varLD
4
3
2
1
0
−1
0
10
20
30
40
50
Position (Mb)

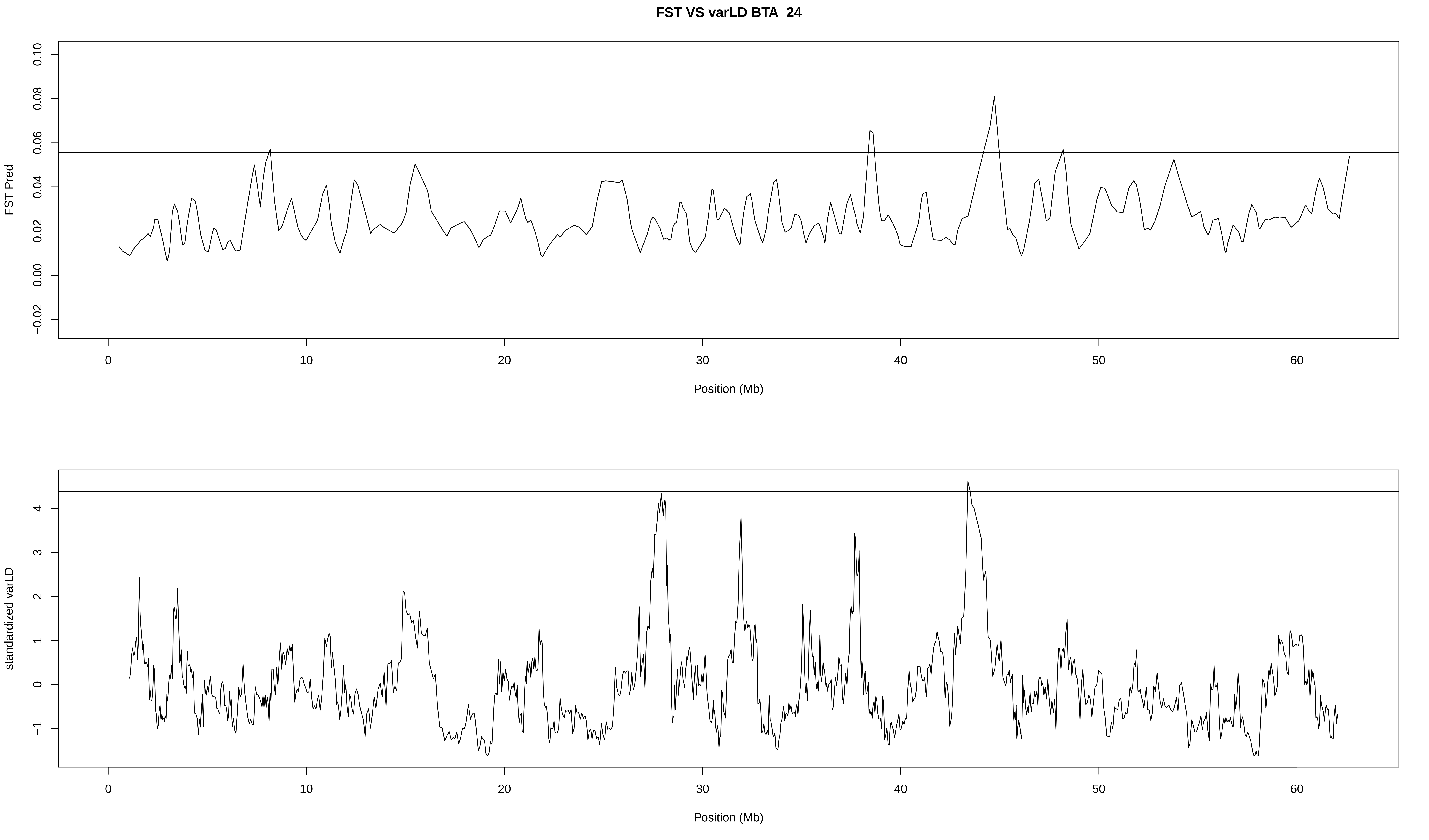
FST VS varLD BTA 24
FST Pred
0.10
0.08
0.06
0.04
0.02
0.00
−0.02
0
10
20
30
40
50
60
Position (Mb)
standardized varLD
4
3
2
1
0
−1
0
10
20
30
40
50
60
Position (Mb)

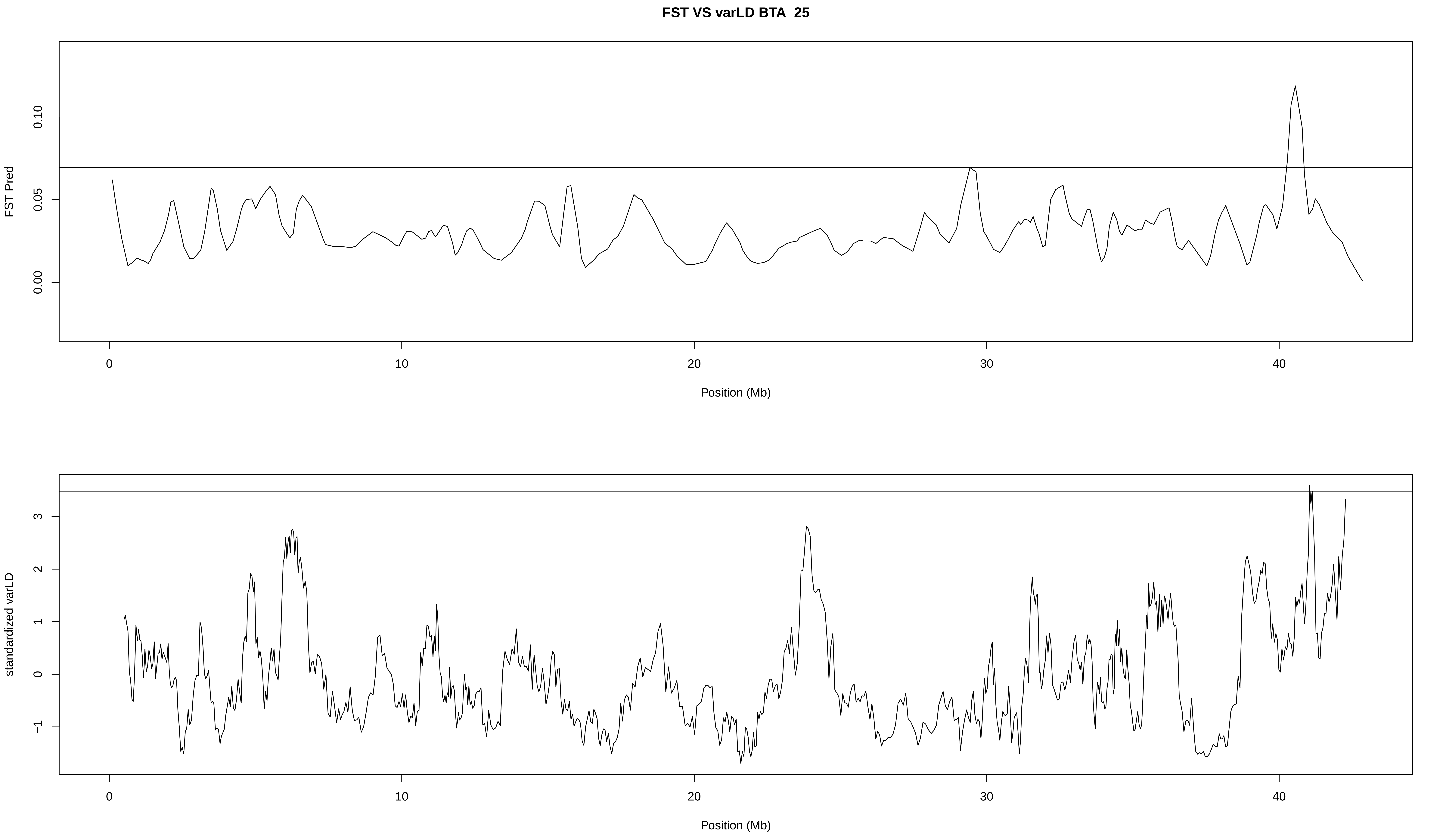
FST VS varLD BTA 25
FST Pred
0.00
0.05
0.10
0
10
20
30
40
Position (Mb)
standardized varLD
−1
0
1
2
3
0
10
20
30
40
Position (Mb)

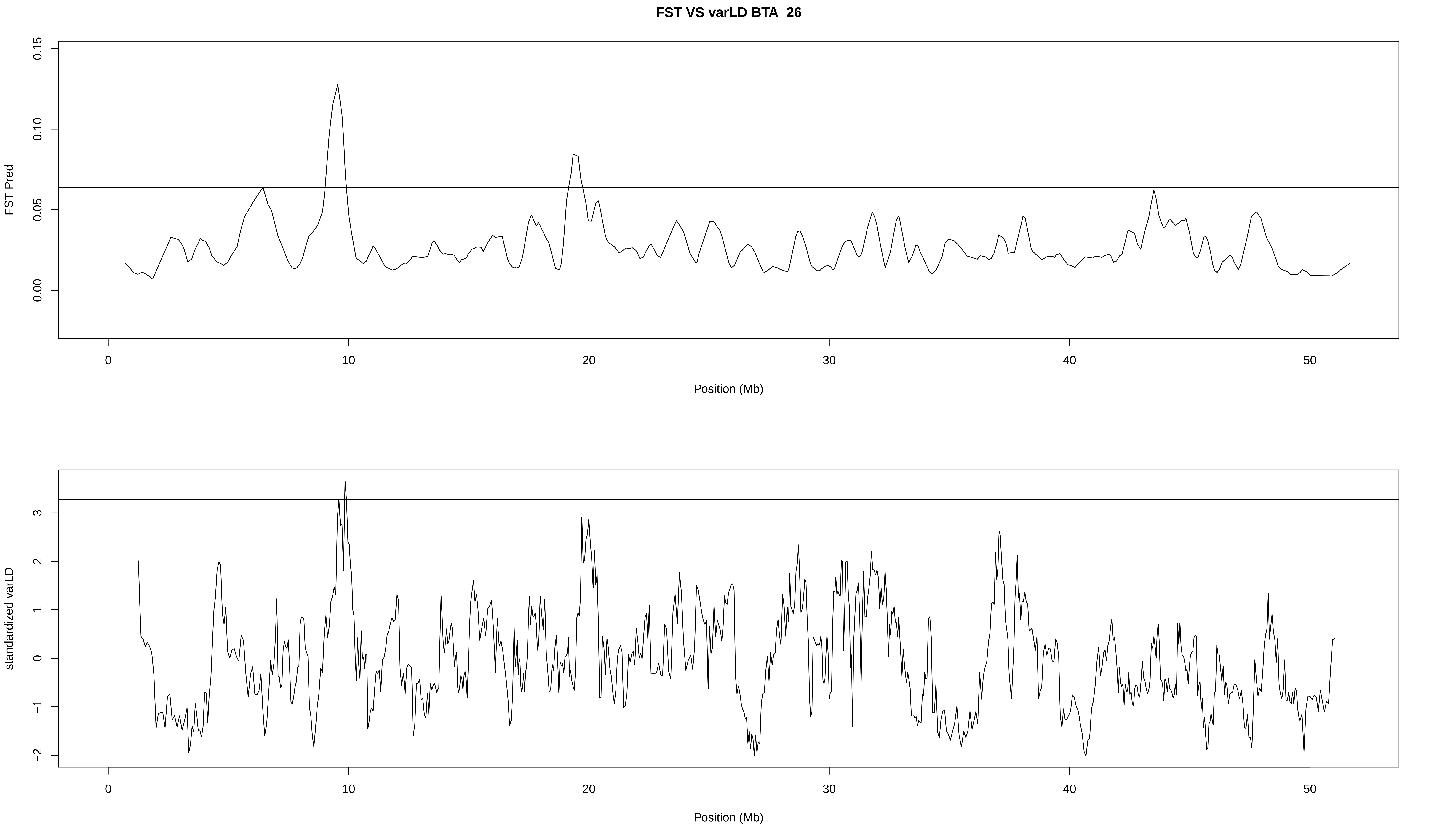
FST VS varLD BTA 26
FST Pred
0.00
0.05
0.10
0.15
0
10
20
30
40
50
Position (Mb)
standardized varLD
−2
−1
0
1
2
3
0
10
20
30
40
50
Position (Mb)

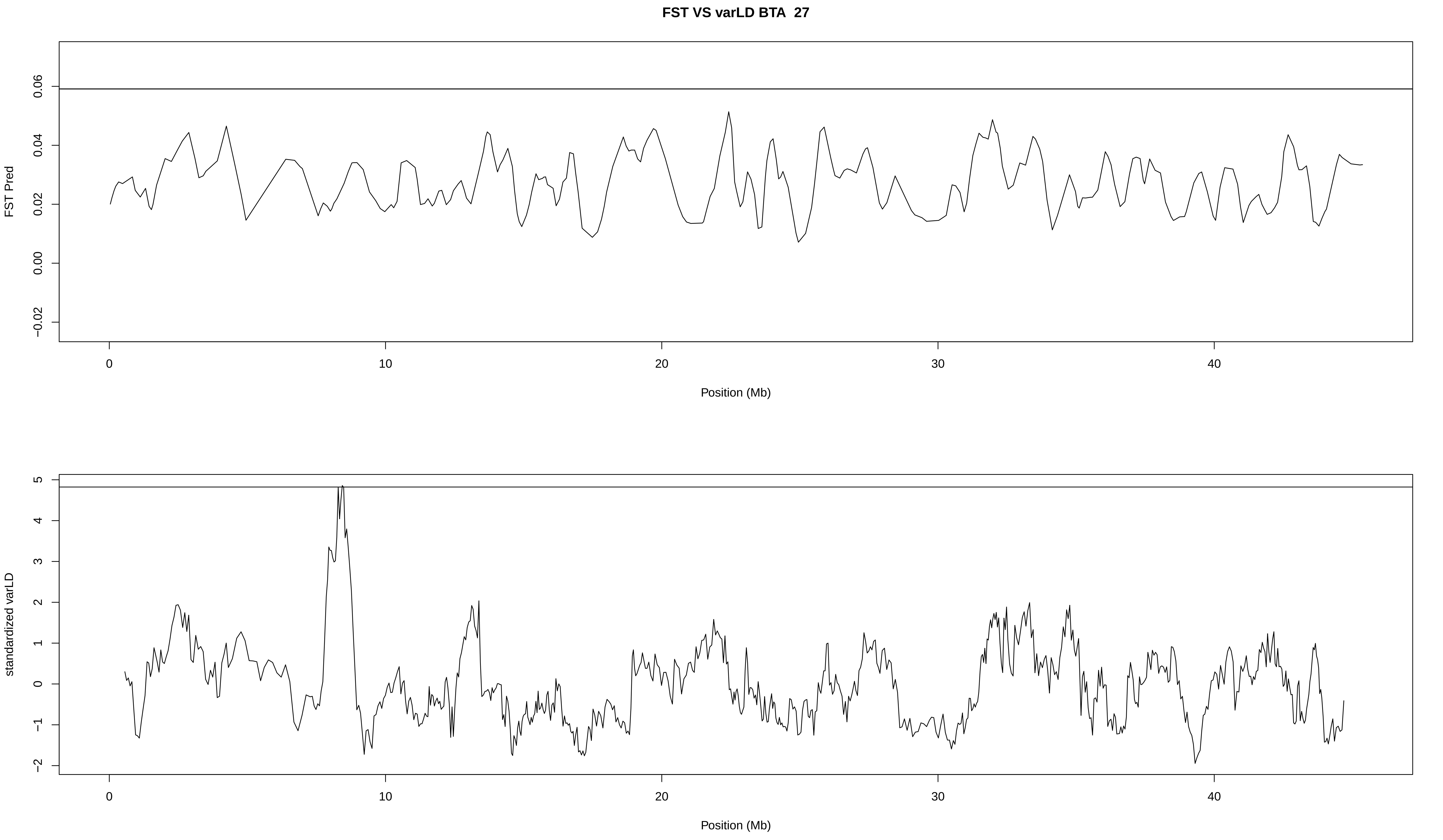

FST VS varLD BTA 27
FST Pred
0.06
0.04
0.02
0.00
−0.02
0
10
20
30
40
Position (Mb)
standardized varLD
5
4
3
2
1
0
−1
−2
0
10
20
30
40
Position (Mb)

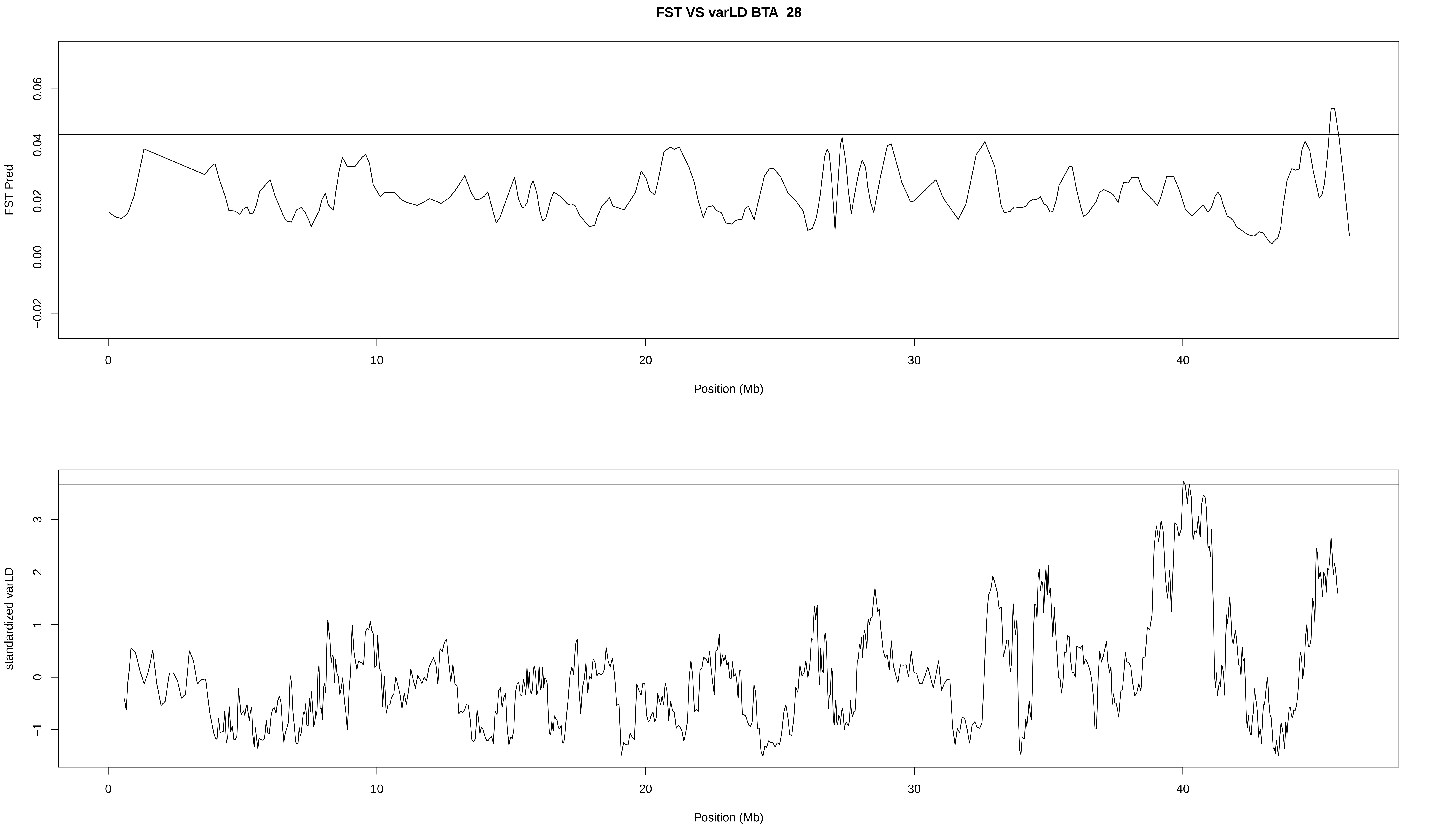
FST VS varLD BTA 28
FST Pred
-0.02
0.00
0.02
0.04
0.06
0
10
20
30
40
Position (Mb)
standardized varLD
-1
0
1
2
3
0
10
20
30
40
Position (Mb)

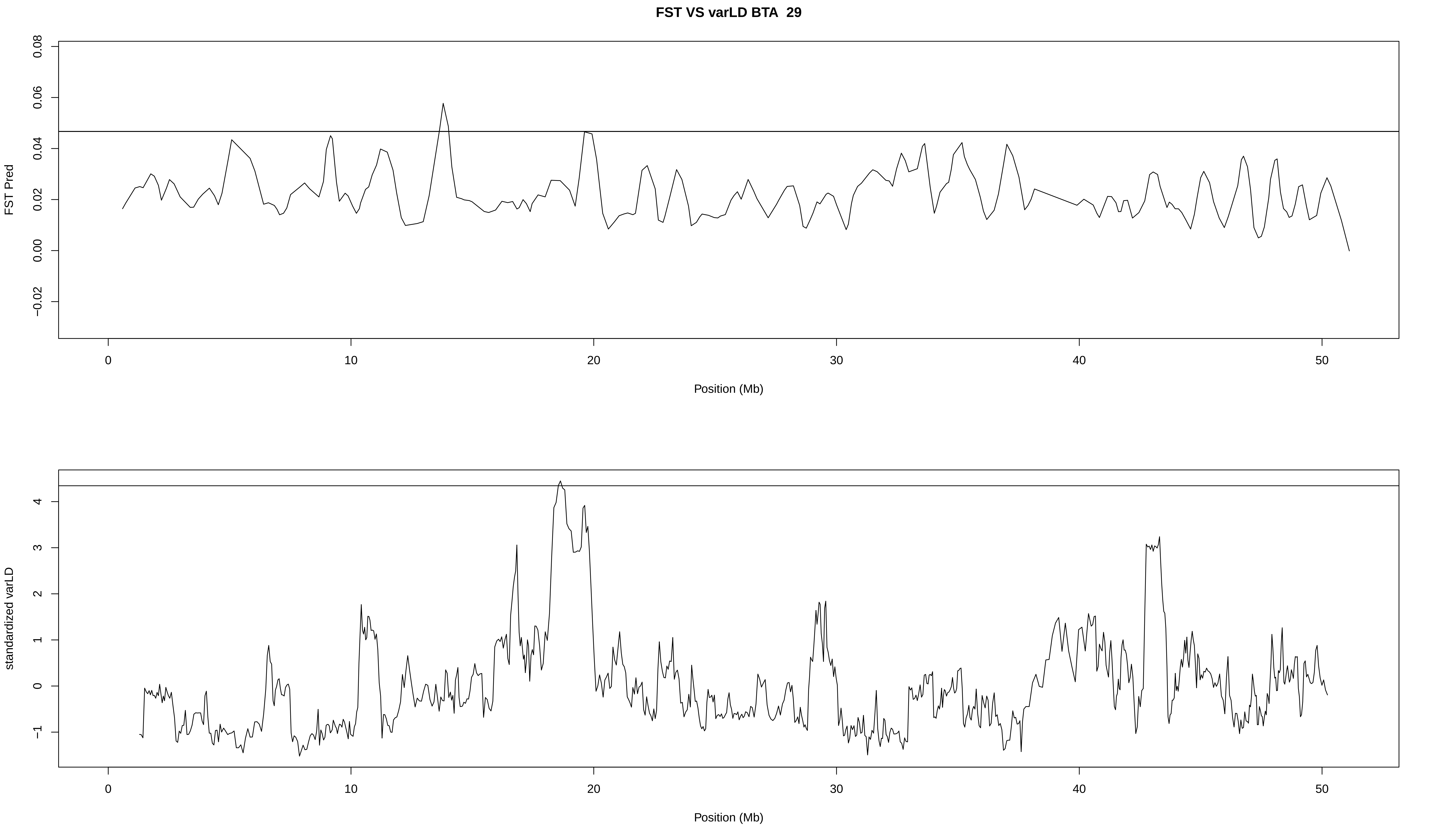
FST VS varLD BTA 29
FST Pred
Position (Mb)
standardized varLD
Position (Mb)
